# Phylogenetic Insights into H7Nx Influenza Viruses: Uncovering Reassortment Patterns and Geographic Variability

**DOI:** 10.3390/v16111656

**Published:** 2024-10-23

**Authors:** Sofya G. Feoktistova, Alexandra O. Ivanova, Egor P. Degtyarev, Daria I. Smirnova, Pavel Yu. Volchkov, Andrei A. Deviatkin

**Affiliations:** 1 Federal Research Center for Innovator and Emerging Biomedical and Pharmaceutical Technologies, 125315 Moscow, Russiavpwwww@gmail.com (P.Y.V.); 2 Shemyakin-Ovchinnikov Institute of Bioorganic Chemistry, RAS (IBCh RAS), 117997 Moscow, Russia; 3 Center for Personalized Medicine, The MCSC Named After A.S. Loginov, 111123 Moscow, Russia

**Keywords:** H7Nx, influenza A viruses, IAVs, H7N2, H7N3, H7N7, H7N9, phylogeny, reassortment

## Abstract

Influenza A viruses (IAVs), which belong to the Orthomyxoviridae family, are RNA viruses characterized by a segmented genome that allows them to evolve and adapt rapidly. These viruses are mainly transmitted by wild waterfowl. In this study, we investigated the evolutionary processes of H7Nx (H7N1, H7N2, H7N3, H7N4, H7N5, H7N6, H7N7, H7N8, H7N9) viruses, which pose a significant pandemic risk due to the known cases of human infection and their potential for rapid genetic evolution and reassortment. The complete genome sequences of H7Nx influenza viruses (*n* = 3239) were compared between each other to investigate their phylogenetic relationships and reassortment patterns. For the selected viruses, phylogenetic trees were constructed for eight genome segments (PB2, PB1, PA, HA, NP, NA, M, NS) to assess the genetic diversity and geographic distribution of these viruses. Distinct phylogenetic clades with remarkable geographic patterns were found for the different segments. While the viruses were consistently grouped by subtype based on the NA segment sequences, the phylogeny of the other segment sequences, with the exception of the NS segment, showed distinct grouping patterns based on geographic origin rather than formal subtype assignment. Reassortment events leading to complex phylogenetic relationships were frequently observed. In addition, multiple cases of previously undescribed reassortments between subtypes were detected, emphasizing the fluidity of H7Nx virus populations. These results indicate a high degree of genetic diversity and reassortment within H7Nx influenza viruses. In other words, H7Nx viruses exist as constantly changing combinations of gene pools rather than stable genetic lineages.

## 1. Introduction

Influenza A viruses (IAVs) are RNA viruses that belong to the *Orthomyxoviridae* family and are characterized by an RNA genome with eight segments. These segments code from 10 to 12 functional viral proteins, depending on the strain [1]. The segmented nature of their genome in combination with an error-prone polymerase facilitates rapid evolution and adaptation to new host species [2]. This ability can lead to the emergence of new strains with pandemic potential. Genetic reassortment, which occurs when a host is coinfected by multiple viruses, further enhances this adaptability by producing offspring with new gene composition [3].

IAVs are primarily found in wild waterfowl and shorebirds, which serve as natural hosts, but they have also been detected in several other avian species and mammalian hosts, including humans. Influenza viruses are classified into subtypes based on the antigenic properties of their surface glycoproteins, hemagglutinin (HA) and neuraminidase (NA). Currently, 16 HA and 9 NA subtypes have been identified in waterfowl [3], and 2 additional subtypes have been discovered in bats [4,5]. In addition, a new strain of IAV was recently collected from *Aythya ferina* in Kazakhstan, whose HA has been tentatively classified as the 19th subtype of HA [6].

In wild birds, IAVs typically cause asymptomatic infections [7]. However, when introduced into new hosts such as terrestrial poultry, they can cause severe disease with high mortality [3]. Therefore, avian influenza viruses (AIVs) are divided into highly pathogenic (HP) and low pathogenic (LP) strains. High-pathogenicity avian influenza (HPAI), especially those of subtypes H5 and H7, have shown high lethality in chickens and other poultry. In contrast, low-pathogenicity avian influenza (LPAI) strains usually cause mild disease limited to the intestinal and respiratory tracts [3,8]. Human infections with HPAI H5N1 have a mortality rate of approximately 60% worldwide [9].

The first documented case of the H7 influenza virus in humans occurred in the USA in 1959 [10]. In the following years, isolated cases were observed in the USA, Australia, and the United Kingdom [11,12,13]. These incidents emphasized the zoonotic potential of this virus and raised concerns about its ability to cause infections in humans.

The first significant event occurred in 2003 with the H7N7 subtype during a large outbreak in poultry in the Netherlands. This outbreak resulted in more than 80 confirmed human cases, mainly with conjunctivitis, and one death [14]. The most concerning emergence of an H7 influenza virus in recent history occurred in 2013 with the H7N9 subtype in China. This virus caused severe respiratory illness in humans and was associated with a high mortality rate [15]. By early 2021, there had been five major waves of H7N9 infections, resulting in more than 1500 laboratory-confirmed cases of human infection and numerous deaths. The H7N9 virus had genetic markers that suggested it could adapt to humans, raising fears of a possible pandemic [15].

These historical events demonstrate the recurring threat of H7 influenza viruses. Each outbreak has provided valuable insights into the transmission dynamics, pathogenicity, and host interactions of the virus. Many cases of new H7Nx (H7N1, H7N2, H7N3, H7N4, H7N5, H7N6, H7N7, H7N8, H7N9) genotypes have been described in the literature. For example, a case of human infection with H7N9 was reported from China in 2014, which is thought to have originated from poultry and reassorted with other H9 or H7 viruses before transmission to humans [16], and a multiple-H7N4 reassortment was isolated and characterized in China in 2018 [17]. The regular emergence of H7 viruses with pandemic potential emphasizes the need for continuous epidemiological surveillance and the importance of studying and understanding the evolutionary processes of new influenza virus variants.

The aim of this study was to provide a comprehensive analysis of the evolutionary dynamics and reassortment patterns of H7Nx influenza viruses (including subtypes H7N1 to H7N9), with a focus on elucidating their potential for genetic diversity, geographic spread, and pandemic risk.

## 2. Materials and Methods

### 2.1. Preparation of Nucleotide Sequence Alignments

All available H7Nx subtype virus genome sequences were downloaded from GenBank (*n* = 23,368) and GISAID (*n* = 36,295) on 30 April 2024. All segment sequences with complete protein-coding regions were concatenated for each virus using a Viral Segment Concatenator [18]. A total of 3239 artificially concatenated genomic sequences of H7Nx IAVs with complete protein-coding regions of eight segments were generated (strains are specified in Supplementary Table S1). The CD-HIT software (version 4.8.1) [19] was used to remove all sequences that differed by less than 1% of nucleotides. The remaining nucleotide sequences were then aligned using the MAFFT software (version 7) [20,21].

### 2.2. Reassortment Analysis

Most reassortment identification methods are based on searching for phylogenetic discrepancies between phylogenetic trees for different segments and comparing pairwise genetic distances between the selected segments of the viral genome [22,23,24,25]. To our knowledge, however, such approaches are labor-intensive and hardly scalable.

At the same time, recombination analysis methods (Recombination Detection Program (RDP5) [26], Genetic Algorithm Recombination Detection (GARD), pairwise distance correspondence plot (PDCP), and pairwise distance divergence matrices (PDDMs) [27]) can process almost any number of sequences simultaneously. Such approaches are not suitable for the simultaneous analysis of multiple segments and are therefore not used for the search for reassortment events. However, in the case of sequential concatenation of all segments, recombination analysis methods can be used to detect traces of reassortment events.

To analyze the frequency and systematic nature of reassortment events in H7Nx viruses, we used a method allowing us to comprehensively identify reassortment events. In the first step of the analysis, all viral segments were sequentially concatenated using the Viral Segment Concatenator [18]. Subsequently, a PDDM was created for each subtype to identify the segments showing the highest pairwise phylogenetic incongruence. PDCP was constructed for the segments of the corresponding H7Nx subtypes selected by PDDM. The viruses that showed pronounced signs of reassortment were selected for a further analysis with PDCP. In this way, datasets of viruses with traces of reassortment were formed for each H7Nx subtype. In addition, similarity plots were generated for selected sequences using Simplot 3.5.1 [28]. The segments of the virus selected as a reference for the similarity plot were compared with the GenBank database using the BLASTn algorithm to complement the datasets with the viruses of the other subtype with the highest degree of identity. RDP5 was implemented in these datasets to validate the results of the reassortment event.

### 2.3. Phylogenetic Analysis

To reveal the cases of reassortment between H7Nx IAVs, the datasets for each subtype were merged and used for phylogenetic reconstruction. Maximum likelihood (ML) phylogenetic inference was performed using IQ-TREE [29]. The best-fitting model was automatically selected using the ModelFinder [30] implemented in the IQ-TREE package (v.1.6.1) according to the Bayesian information criterion. The ultrafast bootstrap (BB) approximation (1000 replicates) was chosen to evaluate the statistical robustness of the internal branching order in the phylogeny [29]. Clades with support greater than 95% were considered reliable.

## 3. Results

A total of 3239 H7Nx virus genomes with all segment sequences with complete protein-coding regions were retrieved from GenBank and GISAID databases (strains are specified in Supplementary Table S1). Traces of reassortment are hardly detectable in nearly identical viruses that differ in their complete genome sequence by less than 1% of the nucleotides. After removal of nearly identical sequences (differing by less than 1% of nucleotides) using the CD-HIT algorithm, a total of 822 viral genome sequences remained (Table 1).

**Figure 1:**
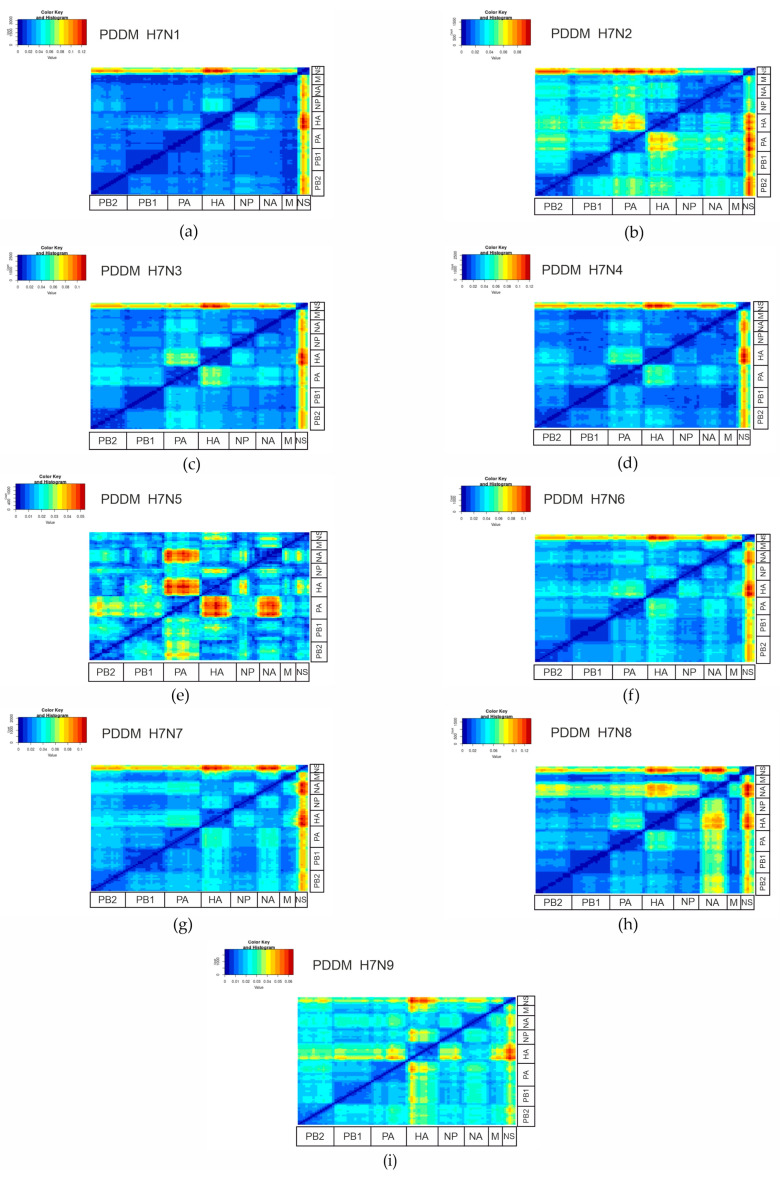
Pairwise distance divergence matrices (PDDMs) for H7Nx influenza viruses, namely H7N1 (**a**), H7N2 (**b**), H7N3 (**c**), H7N4 (**d**), H7N5 (**e**), H7N6 (**f**), H7N7 (**g**), H7N8 (**h**), and H7N9 (**i**) (window = 500 nucleotides, step = 150 nucleotides). The color gradient scale shows the root mean square error (RMSE) values in PDCP as a figure.

The root mean square error (RMSE) of the pairwise distances between two genomic regions relative to the regression line quantifies the degree of phylogenetic discordance between these genomic segments. To analyze this systematically, a pairwise distance deviation matrix (PDDM) was created for all potential pairs of genomic regions using a sliding window of 500 nucleotides and a step of 150 nucleotides. This approach enables the efficient detection of reassortment events in viral genomes by dividing the sequences into smaller segments to compare pairwise genetic distances. The rationale for these parameters lies in the trade-off between sensitivity and precision. A smaller window increases the likelihood that a reassortment will be detected, but also increases the probability of false positive findings. This is because shorter windows encompass less genetic information, which can exaggerate minor variations, leading to false reassortment signals. In contrast, larger windows reduce the number of detected events, but ensure that the detected events are more reliable as they cover a larger portion of the genome. Essentially, larger windows smooth out the random variations and focus on significant genomic changes. The step size of 150 nucleotides was chosen to ensure a balance between resolution and computational efficiency. With smaller step sizes, the number of overlapping windows increases, which improves the resolution but can also increase the computing costs. Larger step sizes reduce the overlap, which in turn reduces the resolution and can lead to certain smaller events being overlooked (results with other step and window values used in PDDM can be found in Supplementary Figures S1–S9). An increased RMSE value, shown in red in Figure 1, indicates increased phylogenetic incongruence between the corresponding genomic regions, while low phylogenetic incongruence, shown in blue, indicates less frequent reassortment. As shown in Figure 1, reassortment between the most divergent viruses in the NS segment was detected for all serotypes except H7N5. According to these results (Figure 1), the polymerase genes (PB2, PB1) were the least prone to reassortment, while HA and NA showed a pattern of segment exchange.

For all serotypes with more than five concatenated genomic sequences in the dataset (Table 1; H7N1, H7N2, H7N3, H7N4, H7N6, H7N7, H7N8, H7N9), PDCPs were constructed for HA and NS segments to show the difference in pairwise evolutionary distances between these genomic regions (Supplementary Figure S10). The deviation from the main trend of the dots on the PDCP plot may be an indicator of a different evolutionary history of the genomic regions analyzed. For example, A/northern shoveler/California/LDC351/2015 (H7N4) and A/northern pintail/Alaska/471/2014 (H7N4) shared 96.0% of nucleotides in the HA segment and 71.6% in the NS segment. These drastic changes in the identity of the different segment sequences can most plausibly be explained by the presumed reassortment event.

**Figure 2:**
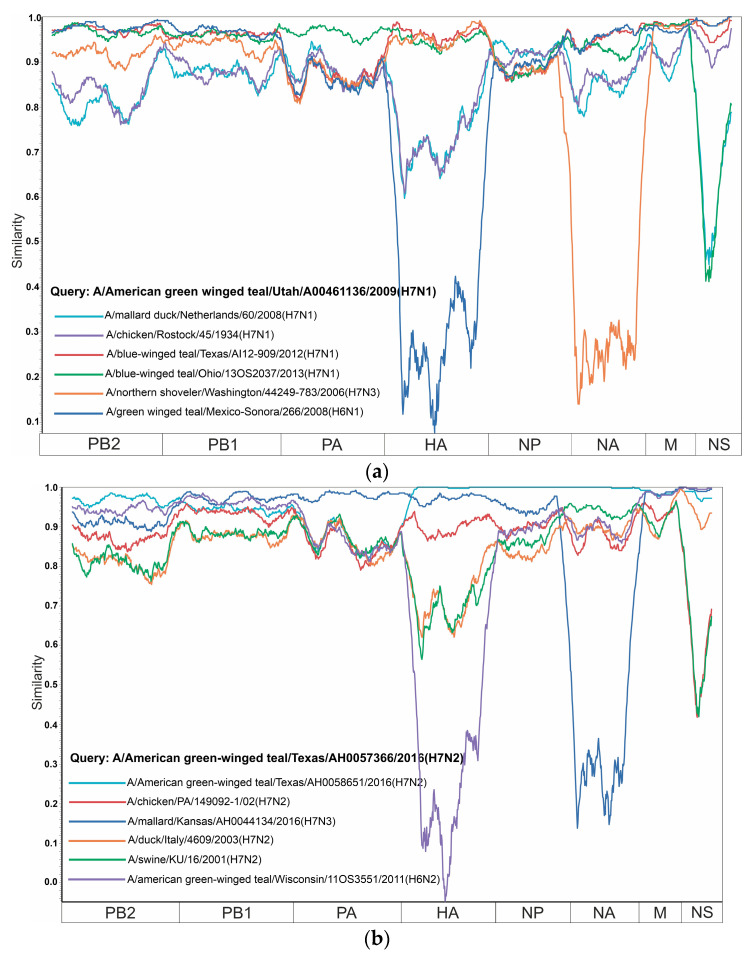

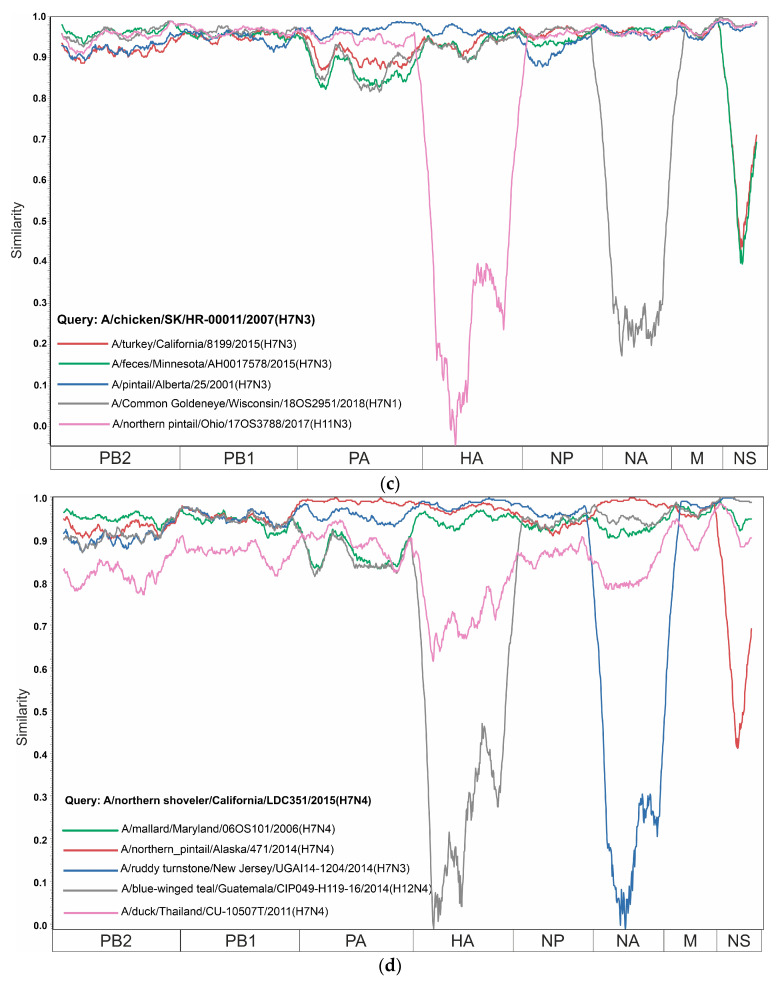

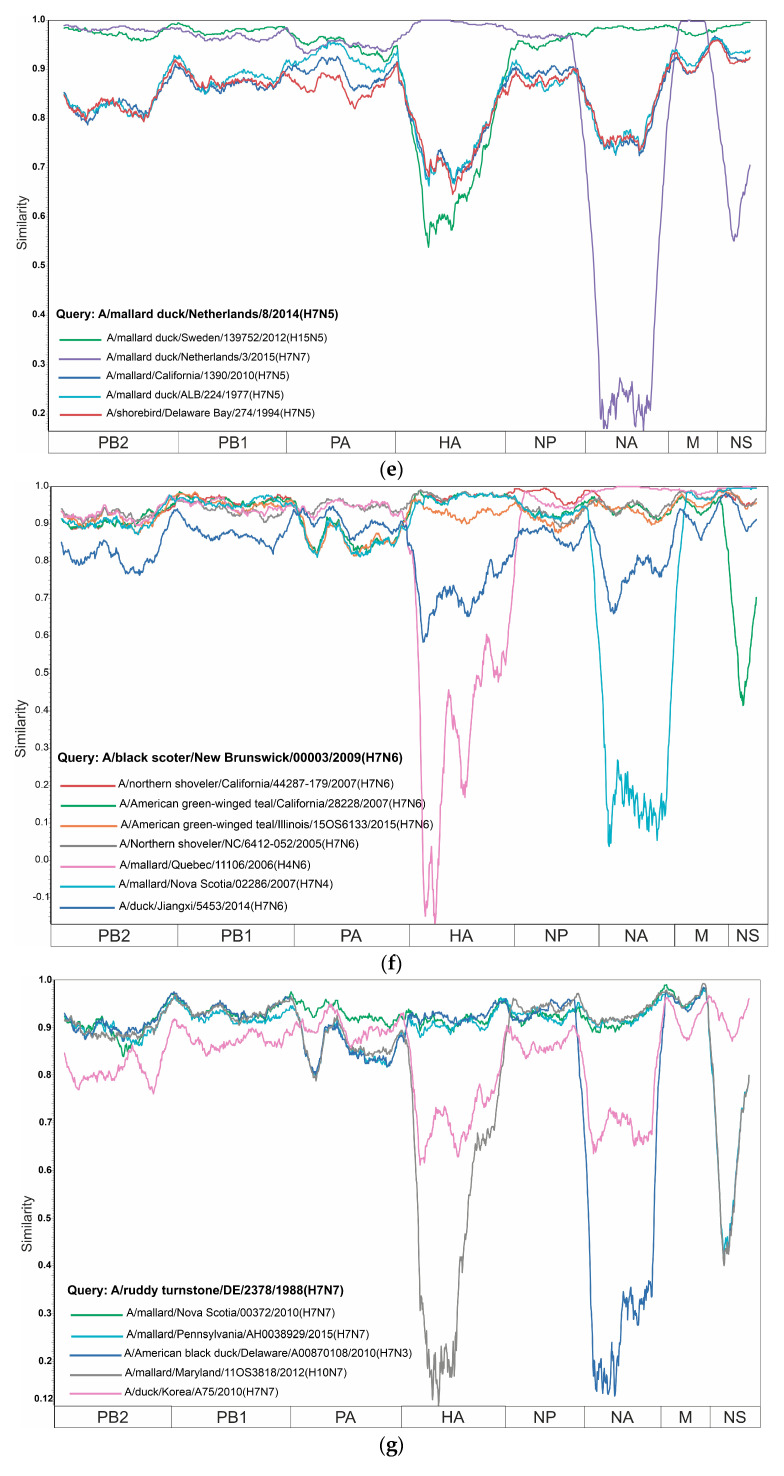

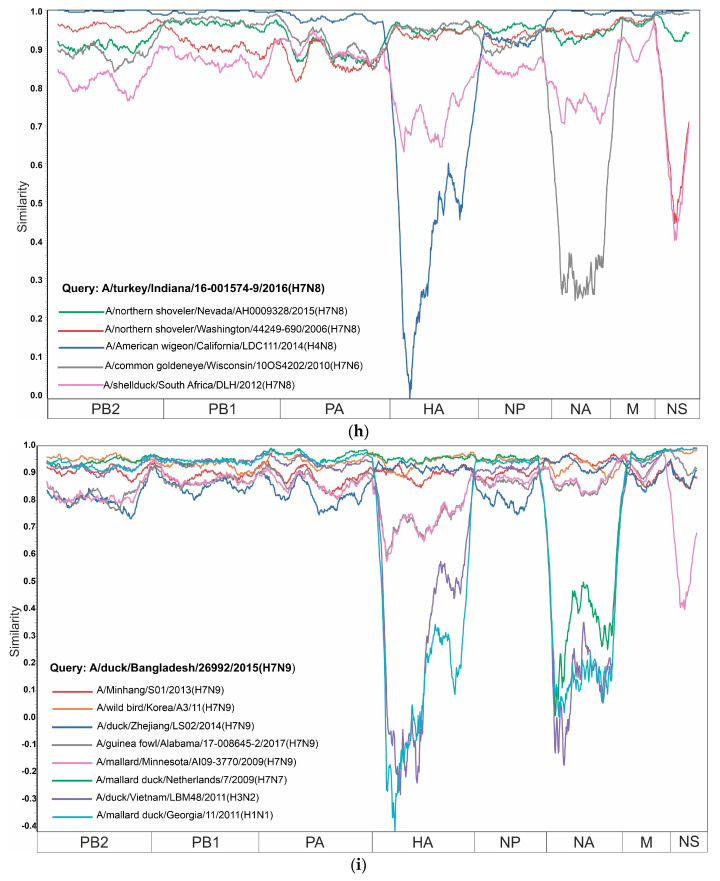
Similarity plot analyses (window = 400, step = 10) for H7Nx influenza viruses, namely H7N1 (**a**), H7N2 (**b**), H7N3 (**c**), H7N4 (**d**), H7N5 (**e**), H7N6 (**f**), H7N7 (**g**), H7N8 (**h**), and H7N9 (**i**). The *x*-axis shows the segments, and the *y*-axis shows the percent similarity between the query sequence and other chosen viruses.

According to the analysis of the PDCP, a set of viruses was selected for each subtype to create the similarity plots (Figure 2). For H7N1, A/American green-winged teal/Utah/A00461136/2009 (H7N1) was selected as the query sequence; for H7N2, A/American green-winged teal/Texas/AH0057366/2016 (H7N2) was selected; for H7N3, A/chicken/SK/HR-00011/2007 (H7N3) was selected; for H7N4, A/northern-shoveler/California/LDC351/2015 (H7N4) was selected; for H7N5, A/mallard duck/Netherlands/8/2014 (H7N5) was selected; for H7N6, A/black scoter/New Brunswick/00003/2009 (H7N6) was selected; for H7N7, A/ruddy turnstone/DE/2378/1988 (H7N7) was selected; for H7N8, A/turkey/Indiana/16-001574-9/2016 (H7N8) was selected; and for H7N9, A/duck/Bangladesh/26992/2015 (H7N9) was selected. Concatenated sequences of two or three viruses differing in the HA or NA subtype from the analyzed subtype were added to each similarity plot to investigate the possibility of intersubtype reassortment. For the selection of additional viruses, a search based on the BLASTn algorithm was performed on the NS segment of the query sequences and the samples with the highest degree of identity were selected for the analysis. As a result, highly pronounced reassortment events were detected within all subtypes presented. In addition, an RDP5 analysis was performed on these datasets (results are presented in Supplementary File S1). These results were consistent with the results obtained with PDCP and PDDM.

For example, the HA segment of virus A/blue-winged-teal/Ohio/13OS2037/2013 (H7N1) has 94.8% identical nucleotides with virus A/American-green-winged-teal/Utah/A00461136/2009 (H7N1), while the NS segments of these viruses share 69.0% of identical nucleotides. At the same time, the NS segment of virus A/blue-winged teal/Ohio/13OS2037/2013 (H7N1) has 96.2% identical nucleotides with the NS segment of virus A/green-winged teal/Mexico-Sonora/266/2008 (H6N1) (Figure 2a). The most plausible explanation for this situation is the reassortment that took place in the ancestors of these viruses.

Most of the internal gene segments showed the similarity between strain A/American green-winged teal/Texas/AH0057366/2016 (H7N2) and other influenza H7N2 strains (Figure 2b). Although strain A/swine/KU/16/2001 (H7N2) is similar in most segments, it showed a drastic decrease in similarity in the HA segment to as low as 74.1% of identical nucleotides. The same pattern is observed for strain A/duck/Italy/4609/2003 (H7N2), whose HA segment differs from that of query strain A/American green-winged teal/Texas/AH0057366/2016 (H7N2) by more than 24.8% of nucleotides. The NS segment for strains A/American green-winged teal/Texas/AH0058651/2016 (H7N2), A/mallard/Kansas/AH0044134/2016 (H7N3), A/duck/Italy/4609/2003 (H7N2), and A/American green-winged teal/Wisconsin/11OS3551/2011 (H6N2) showed an almost identical match to the query strain, although strains A/chicken/PA/149092-1/02 (H7N2) and A/swine/KU/16/2001 (H7N2) had only 69.8% of identical nucleotides.

The analysis of the similarity plot revealed a high degree of similarity between the PB2, PB1, PA, NP, and M gene segments for query strain A/chicken/SK/HR-00011/2007 (H7N3) compared to several other influenza strains (Figure 2c). However, the NS segment shows a significant decrease in similarity, especially when compared to strains A/turkey/California/8/99/2015 (H7N3) and A/feces/Minnesota/AH017578/2015 (H7N3), where similarity was significantly reduced to 66.4% of identical nucleotides, while for strains A/northern pintail/Ohio/17OS3788/2017 (H11N3) and A/Common Goldeneye/Wisconsin/18OS2951/2018 (H7N1), the similarity remains relatively high, generally above 98.5% of identical nucleotides.

Similarly, in the plots in Figure 2d–i, similarities were found for most of the internal gene segments with the query strains and significant differences (30–40%) for the HA segment within one subtype (H7). In addition, all these plots are characterized by a decrease in identical nucleotides for the NS segment between the query strains and some other strains. The differences between the query strains and other samples in the HA segment may have a geographical explanation, while the difference in the NS segment is not related to the location of sample isolation. The high degree of identity between the internal gene segments of query strains and strains with other subtypes observed in all similarity plots in this study could be a consequence of reassortment between the subtypes.

**Figure 3:**
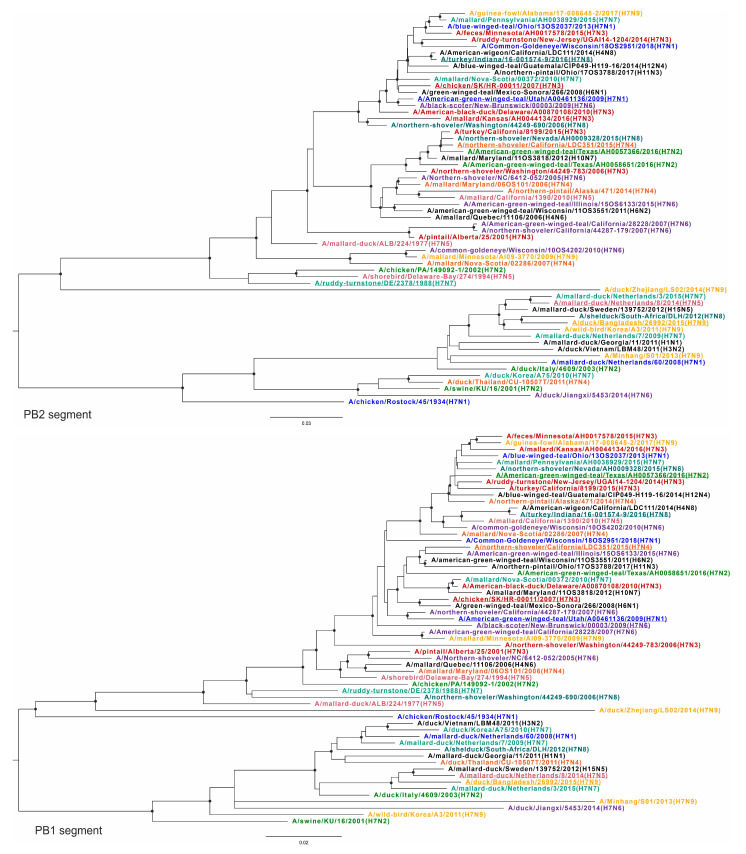
Phylogenetic trees for PB2 and PB1 segments. Each H7Nx subtype is color-coded; other subtypes are indicated in black. Black circles indicate nodes that were supported by ultrafast bootstrap approximation values above 95%.

**Figure 4:**
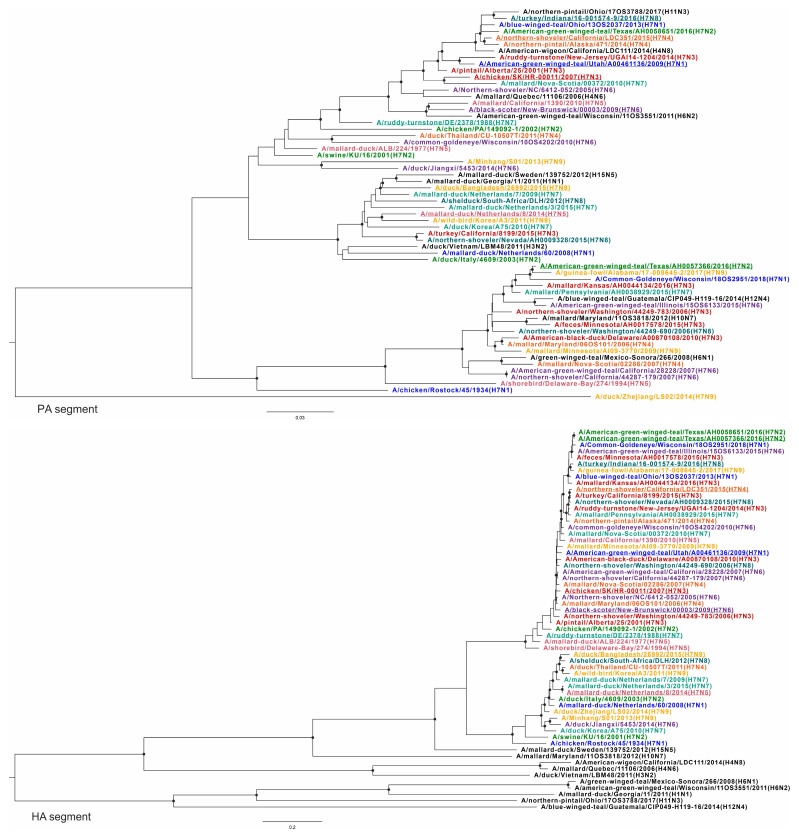
Phylogenetic trees for PA and HA segments. Each H7Nx subtype is color-coded; other subtypes are indicated in black. Black circles indicate nodes that were supported by ultrafast bootstrap approximation values above 95%.

**Figure 5:**
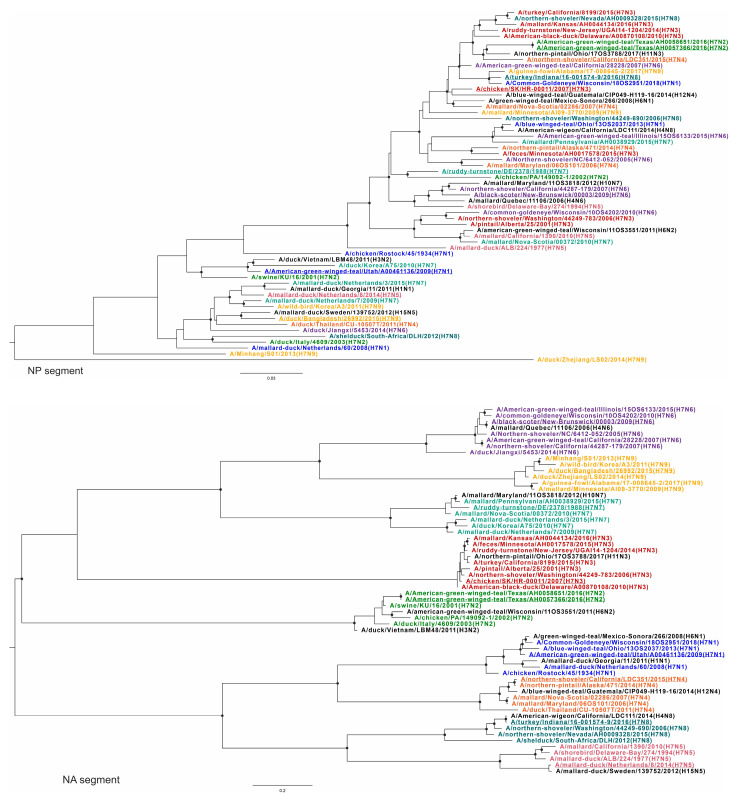
Phylogenetic trees for NP and NA segments. Each H7Nx subtype is color-coded; other subtypes are indicated in black. Black circles indicate nodes that were supported by ultrafast bootstrap approximation values above 95%.

**Figure 6:**
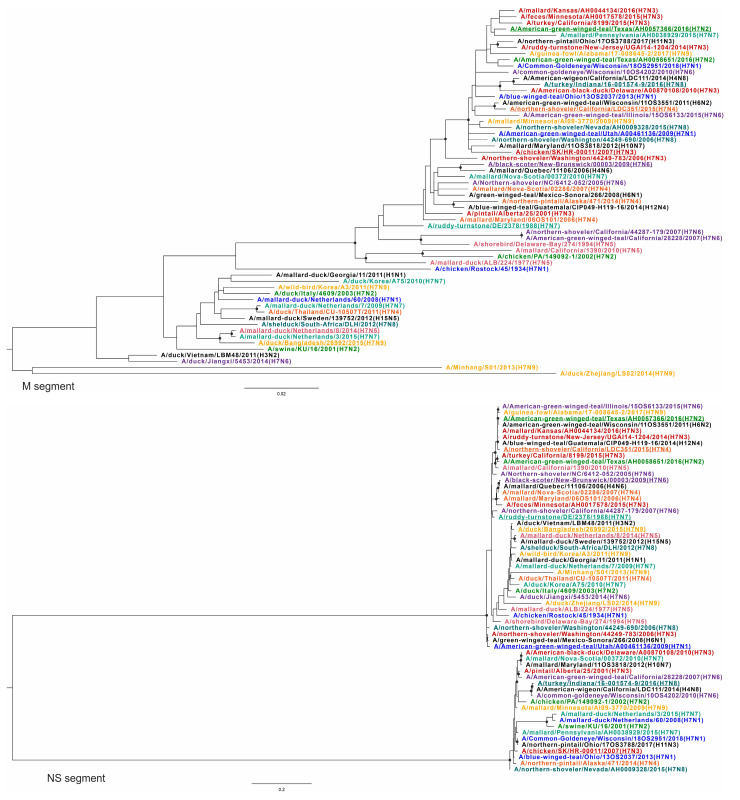
Phylogenetic trees for M and NS segments. Each H7Nx subtype is color-coded; other subtypes are indicated in black. Black circles indicate nodes that were supported by ultrafast bootstrap approximation values above 95%.

To confirm the above results demonstrating extensive reassortment within and between subtypes of H7Nx viruses, phylogenetic trees were constructed for all virus variants and used to generate similarity plots. To visualize changes in the topology of the phylogenetic trees based on different segments, each subtype was assigned a specific color. The query sequences (according to the similarity analysis) for each group are underlined (Figure 3, Figure 4, Figure 5 and Figure 6) (Supplementary Figures S11–S18, phylogenetic trees with higher resolution).

Different topologies can be recognized in the phylogenetic trees based on different segments. All viruses with the same neuraminidase subtype were grouped into distinct clades (Figure 5, tree NA). At the same time, such grouping was not consistent for other segments (Figure 3, Figure 4, Figure 5 and Figure 6). For example, A/mallard-duck/Netherlands/8/2014 (H7N5) was reliably closer to a representative of another subtype, A/mallard-duck/Netherlands/3/2015 (H7N7) than to A/shorebird/Delaware-Bay/274/1994 (H7N5), based on HA segment sequences (Figure 4, HA tree). On the one hand, this can be explained by the fact that the HA segments of the two viruses collected in the Netherlands in 2014 and 2015 originate from a single recent ancestor. On the other hand, their NA segments definitely have a different origin (Figure 5, NA tree). To summarize, the phylogenetic trees for all segments except NA show that the viruses are not grouped according to their affiliation to a formal subtype. Furthermore, it should be noted that for most phylogenetic trees, the viruses were grouped according to the geographic region where the biomaterial was collected, but not according to their formal subtype assignment.

According to the PB2 phylogenetic tree, all H7Nx viruses could be tentatively divided into two large, geographically contiguous clades consisting of viruses collected in North America and viruses collected in Eurasia and Africa. It should be noted that only one virus collected in Africa was included in the dataset—A/shelduck/South-Africa/DLH/2012 (H7N8). In addition, the virus collected in China—A/duck/Zhejiang/LS02/2014 (H7N9)—had an origin independent of these two clades. The phylogenetic trees based on the sequences of PB1, HA, NP, and M segments show a similar pattern of virus grouping. For the PA segment, viruses collected in Eurasia and Africa formed a clade with one group of viruses collected in North America, while the other group of viruses collected in North America was an outgroup. It should be noted that in several cases, exceptions to this division were found based on geographic characteristics.

The phylogenetic tree for the NP segment retains the division into clades and subclades based on geographic features, except for virus A/American green winged teal/Utah/A00461136/2009 (H7N1) collected in the USA, which is phylogenetically closest to the NP segments of viruses collected in East Asia. In addition, according to the phylogenetic tree for the PA segment, the closely related A/turkey/California/8199/2015 (H7N3) and A/northern-shoveler/Nevada/AH0009328/2016 (H7N8) were grouped with viruses collected in Eurasia, but not with viruses collected in North America, as observed in the phylogenetic trees for other segments (PB2, PB1, HA, NP, M).

It should be noted that the phylogenetic relationship was also different for different sequences of viruses collected in one geographic region. For example, the NP segments of A/American-green-winged-teal/Texas/AH0058651/2016 (H7N2) and A/American-green-winged-teal/Texas/AH0057366/2016 (H7N2) are in the same subclade. They are phylogenetically closest to A/northern-pintail/Ohio/17OS3788/2017 (H11N3) and A/northern-shoveler/California/LDC351/2015 (H7N4). At the same time, these viruses formed different clades according to the phylogenetic tree based on PB1 segments. A/American-green-winged-teal/Texas/AH0058651/2016 (H7N2) was phylogenetically the most closely related to A/northern-pintail/Ohio/17OS3788/2017 (H11N3), while A/American-green-winged-teal/Texas/AH0057366/2016/2016 (H7N2) was the most closely related to A/northern-shoveler/Nevada/AH0009328/2015 (H7N8) and A/mallard/Pennsylvania/AH0038929/2015 (H7N7).

As mentioned above, the viruses in the phylogenetic trees of NA segment sequences were grouped into clades consisting of viruses of the same subtype. At the same time, a phylogenetic tree based on NS segment sequences was drastically distinguishable from other phylogenetic trees (Figure 6, NS tree). All viruses were categorized into two clades regardless of their subtype or geographic affiliation. It should be noted that the viruses in these clades varied considerably from each other. For example, the NS segment sequences of A/ruddy turnstone/DE/2378/1988 (H7N7) and A/mallard/Nova Scotia/00372/2010 (H7N7) differed in 243 of 868 nucleotides (28.0%). Since the differences between the other segment sequences of these viruses are not as pronounced, reassortment is the most plausible explanation for this observation.

## 4. Discussion

Research into the evolution of influenza viruses is crucial for understanding the mechanisms of their spread, adaptation to new hosts, and development of pathogenic variants [31]. The rapid rate at which influenza viruses evolve due to the high mutation rate and reassortment of genes [32] requires continuous monitoring and detailed analyses of genetic changes. In the current study, we focused on the detection and investigation of reassortment patterns of H7Nx (H7N1, H7N2, H7N3, H7N4, H7N5, H7N6, H7N7, H7N8, H7N9) influenza viruses, as these subtypes have a high pandemic potential [33].

Significant differences (up to 30% non-identical nucleotides) between serotype 7 hemagglutinins (H7) collected in North America and Eurasia were found even within a single subtype (similarity plots in Figure 2). Combined with the fact that genes encoding hemagglutinins are frequently exchanged between viruses of different subtypes, this means that the efficacy of vaccines developed against H7Nx viruses [33] should be estimated with caution [34].

The H7 sequences were grouped into clades on the phylogenetic tree for the HA segment according to their geographic origin. Viruses with different HA and NA subtypes have internal genes with a high degree of identity, in some cases even higher than viruses with the same HA and NA variants. From the other side, the sequences of the segments encoding internal proteins (PB2, PB2, PA, NP, M), except for the NS segment, were clustered mainly according to the geographical proximity of the locations where the samples were collected. This observation suggests that geographical proximity facilitates reassortment events, leading to a complex evolutionary landscape that could differ by gene segment. It should be noted that the sequences of genes encoding internal proteins of representatives of the same subtype were often more similar to the sequences of viruses of different subtypes than to the sequences of viruses of the same subtype. For example, the PB2 segment sequences of A/duck/Korea/A75/2010 (H7N7) and A/mallard/Nova Scotia/00372/2010 (H7N7) differed in 364 of 2271 nucleotides (16.0%), while the PB2 segment sequences of A/Mallard/Nova Scotia/00372/2010 (H7N7) and A/green teal/Mexico-Sonora/266/2008 (H6N1) differed in 49 of 2316 nucleotides (2.1%). The most plausible explanation for these observations is constant and frequent reassortment events within and between subtypes. In addition, the different segments might have an independent evolutionary history. Considering the active reassortment between different subtypes of influenza viruses, classifying these subtypes based only on the differences between two segments seems to be to some extent an oversimplification of the picture of complex phylogenetic relationships. This concept challenges the traditional view of stable lineages and points to a more fluid viral population structure.

It was previously shown that changes in the proteins encoded in the segments PB2 [35], PB1 [36], PA [37], NP [38], M [39], and NS [40] can predetermine the pathogenicity of IAV. Reassortment may therefore lead to new combinations of segment variants that may enhance the pathogenic properties of a new virus. It should be noted that the NP, PA, and M segments of the A/duck/Zhejiang/LS02/2014 (H7N9) virus form outgroups in the corresponding tree (Figure 4, Figure 5 and Figure 6). They are phylogenetically the most distant from all other viruses in the dataset. Interestingly, according to the BLASTn search in the GenBank database, the sequences of the NP, PA, and M segments of this virus were closely related to the representatives of the other subtypes. For example, the NP segment of A/duck/Zhejiang/LS02/2014 (H7N9) has 1426 of 1497 nucleotides (95.3%) with the NP segment of A/New York/206/2005 (H3N2), the M segment has 910 of 985 nucleotides (92.4%) with the M segment of A/duck/Jiangxi/35/2011 (H5N1), and the PA segment has 2112 of 2151 nucleotides (98.2%) with the PA segment of A/New York/463/2005 (H3N2). Thus, A/duck/Zhejiang/LS02/2014 (H7N9) is a combination of segments attributable to different IAV subtypes, at least some of which have high pandemic potential [41]. The addition of these segments to the actively reassorting pool of the H7Nx gene could increase the chance of the emergence of novel viruses with high pathogenicity and the ability to overcome the barrier of host adaptation [42]. To the best of our knowledge, there is currently no information on the phenotypic characteristics of A/duck/Zhejiang/LS02/2014 (H7N9). The phenotypic characteristics of the reassorted virus A/duck/Zhejiang/LS02/2014 (H7N9) were assessed by analyzing specific amino acid substitutions that are known to increase pathogenic potential. As a result, the following substitutions were discovered in the protein sequences of A/duck/Zhejiang/LS02/2014 (H7N9), which are associated with pathogenicity. For instance, the I106M substitution in the NS1 protein increases virulence in mice by enhancing CPSF30 binding [43]. Similarly, substitutions S37A and N383D in the PA protein boost polymerase activity in human cells [44], while K526R and E627K in the PB2 protein enhance viral replication in mammalian cells [45,46]. Despite these pathogenic markers, the HA cleavage site sequence, PEVPKGR/GLF, is typical for LPAIV. However, it is important to note that no experimental data are available to confirm the phenotypic effects of all these substitutions together, leaving their combined impact on virulence speculative at this stage.

The difference between HPAI and LPAI lies primarily in their pathogenicity, a phenotypic characteristic that determines the severity of the disease caused by the virus [47,48,49,50,51]. The cleavage site of HA, which is processed by cellular proteases, can vary and largely determines the pathogenicity of IAVs. For example, Kim et al. [52] showed that an influenza virus that has 1 of the 13 cleavage site variants associated with low pathogenicity in its HA is an LPAI virus. If at the same time 1 of the 29 cleavage sites associated with high pathogenicity is found in the HA, it is an HPAI virus. According to this information, 41 viruses from our dataset could be assigned as HPAI, 2915 viruses could be assigned as LPAI, and 283 viruses did not contain any of the cleavage sites listed in the table of Kim et al.’s study [52]. In addition, other segments of the influenza genome, such as those encoding polymerase proteins or the non-structural protein (NS), may also contain determinants of pathogenicity, as suggested by several studies [53,54,55]. Therefore, predictions made in silico should be taken with caution.

Pools of IAV genes, which are divided into segments, can form new gene combinations under the condition of the coinfection of an organism by different strains. This phenomenon is essentially referred to as reassortment. In this study, we have shown that reassortment within and between subtypes is frequent and systematic in H7Nx viruses (Figure 2, Figure 3, Figure 4, Figure 5 and Figure 6). This situation is likely a consequence of the ecology of H7Nx—its natural reservoirs, waterfowl, can form large, dense flocks [56]. In addition, viable IAVs can survive in freshwater [57] for at least months [58]. Not only waterfowl, but also lakes with stagnant water, for example, where these birds spend time, can serve as a natural reservoir of infection. This creates a unique environment for the coexistence of different virus variants, which maintain and increase their genetic diversity through multiple reassortments. This suggests that the H7Nx viruses do not survive in the natural host as a series of stable viral clones. In some cases, reassortment leads to the crossing of the interspecies barrier or the emergence of new highly pathogenic variants, such as H7N4, which was isolated in humans in China in 2020 [17], or the widely studied pandemic influenza H1N1 [59].

Based on amino acid [60] and nucleotide homology [61], the NS segments of all IAVs can be categorized into two different gene pools: allele A and allele B. These alleles formed two clades on the NS phylogenetic tree (Figure 6). Viruses carrying the NS segment belonging to the A allele infect a broader range of hosts than viruses belonging to the B allele [62], and artificial reassortment of the NS segment significantly alters the ability of the virus to replicate in different cell types [40]. At the same time, the reasons for such a phylogenetic division of these two NS lineages are currently unclear [63]. Nevertheless, every H7Nx subtype except H7N5 contained viruses with both alleles. The high topological incompatibility between the phylogenetic tree for the NS segment and the phylogenetic trees for the other seven segments can apparently only be explained by active reassortment processes in the evolutionary history of H7Nx viruses. Furthermore, the lack of geographic isolation between H7Nx viruses collected in North America and Eurasia, according to the phylogeny of the NS segment, suggests that intercontinental exchange of these viruses is systematic.

It is noteworthy that the number of full-length H7N5 genomes is only 1% of the total number of full-length H7Nx genomes (Table 1). According to Table 1, the rarest serotype among all H7Nx is H7N5, which has been detected in Bangladesh, Cambodia, the USA, Canada, the Netherlands, Belgium, Iceland, and Australia. It should be noted that the absence of H7N5 sequences of viruses collected in countries between these regions (e.g., China, Japan, Korea, Russia, Germany) despite an existing influenza virus surveillance system [64,65,66,67,68] does not indicate the absence of H7N5 in these countries, but rather a fragmentary knowledge of the actual diversity and spread of this pathogen.

The IAV dataset used in this study (Supplementary Table S1, *n* = 3239) showed a pronounced bias in the collection of samples. A significant proportion of viruses were collected from humans (*n* = 800), chickens (*n* = 846), and turkeys (*n* = 105), reflecting a strong focus on hosts with direct relevance to human health and agriculture. Ducks, one of the natural reservoirs for IAV, were reported as hosts for 965 viruses, but other important species were significantly underrepresented. Despite the well-documented role of waterfowl and other wild birds (except ducks) in the transmission ecology of IAV, this group was severely underrepresented (*n* = 235). Environmental samples, which are crucial for understanding viral persistence and transmission dynamics outside the host organisms, also accounted for only 233 IAVs. Only isolated cases of infection in mammals such as a pika, cat, horse, seal, and pig were detected, suggesting sporadic transmission from birds to mammalian hosts. This biased sampling focus on humans and domestic animals limits our ability to fully understand the diversity of IAV and the ecological factors driving its evolution and spread. By overlooking wild bird populations and environmental reservoirs, important insights into the behavior of the virus in non-human hosts are missed.

The global surveillance system for avian influenza viruses consists of a network of separate national systems operating at different intensities. The establishment of robust genetic surveillance programs in areas with known migratory waterfowl populations would increase the virus detection rate. Promoting international collaboration and data sharing would facilitate standardized protocols for sampling and sequencing and enable comprehensive analyses of IAV outbreaks (e.g., H7N5). Finally, targeted surveillance of specific habitats, such as wetlands frequented by waterfowl, would focus surveillance efforts where the virus is most likely to occur. These measures could close knowledge gaps about the spread of H7N5 and other IAV subtypes and improve the overall understanding of the ecology of this pathogen. In addition to efficient surveillance to control the spread of infection, it is of great importance to gain insights into the evolution and pathogenesis of the virus. Recent advances in virus modeling have produced sophisticated tools that can significantly improve the understanding of viral evolution and transmission dynamics. In particular, studies of influenza and other viral pathogens have emphasized the use of computational analyses. Comprehensive reviews of viral modeling approaches [69] and influenza-specific models [70], for example, provide valuable insights into methods for predicting outbreaks and understanding transmission patterns.

It should be noted that the enormous diversity of IAVs means that, despite the active development of universal influenza vaccines [71,72], there are currently no ready-made preparations. For this reason, there are inevitable concerns that in the case of the emergence of a new influenza virus that can be transmitted from person to person [73], there will initially be no effective means of preventing this infection. Expanding genomic surveillance to capture a broader range of H7Nx variants and understanding the mechanisms driving reassortment between virus subtypes and their impact on viral pathogenicity will be critical to improving vaccination strategies and pandemic preparedness.

## 5. Conclusions

In the present study, we analyzed all available complete genomes of H7Nx viruses. Based on the methodology used, previously undescribed reassortment events were identified in all H7Nx subtypes. In addition, examples of reassortment between subtypes were uncovered—internal segments of individual H7Nx viruses were closer to other subtypes than to H7Nx viruses of the same subtype. These cases of reassortment and the limited number of some subtypes in the databases emphasize the importance of continuous surveillance of influenza viruses and the use of whole-genome sequencing to better understand their evolution and distribution patterns.

## Figures and Tables

**Table 1:** IAV subtypes used in the analysis.

Subtype	Before Filtration by Identity	After Filtration by Identity (Sequences with More Than 99.0% of Identical Nucleotides Were Omitted)
H7N1	123	57
H7N2	316	68
H7N3	691	278
H7N4	37	22
H7N5	7	4
H7N6	50	28
H7N7	364	144
H7N8	36	20
H7N9	1615	201
**Total**	**3239**	**822**

## Data Availability

Restrictions apply to the availability of these data. Data were obtained from GISAID and are available at https://gisaid.org/ (accessed on 10 September 2024) with the permission of GISAID. Names of the H7Nx virus strains analyzed in the study (*n* = 3239) are specified in Supplementary Table S1.

## Supplementary Materials

The following supporting information can be downloaded at: https://www.mdpi.com/article/10.3390/v16111656/s1. Names of the H7Nx virus strains analyzed in the study (*n* = 3239) are specified in Table S1. Figures S1–S9: Pairwise distance divergence matrices (PDDMs) for H7Nx influenza viruses; Supplementary Figure S10: Pairwise nucleotide distance comparison plots (PDCPs) showing phylogenetic incongruence between selected genetic regions of H7Nx influenza viruses; Figures S11–S18: Phylogenetic trees with higher resolution; File S1: RDP5 result files for H7Nx influenza viruses.
