# Supplementary material for: Phylogenetic Insights into H7Nx Influenza Viruses: Uncovering Reassortment Patterns and Geographic Variability
Source: Viruses. 2024 Oct 23;16(11):1656. doi: 10.3390/v16111656 (PMC11598867; doi:10.3390/v16111656)
Supplement: Supplementary file 1 [file viruses-16-01656-s001.zip › Supplementary Figure S10.pdf]

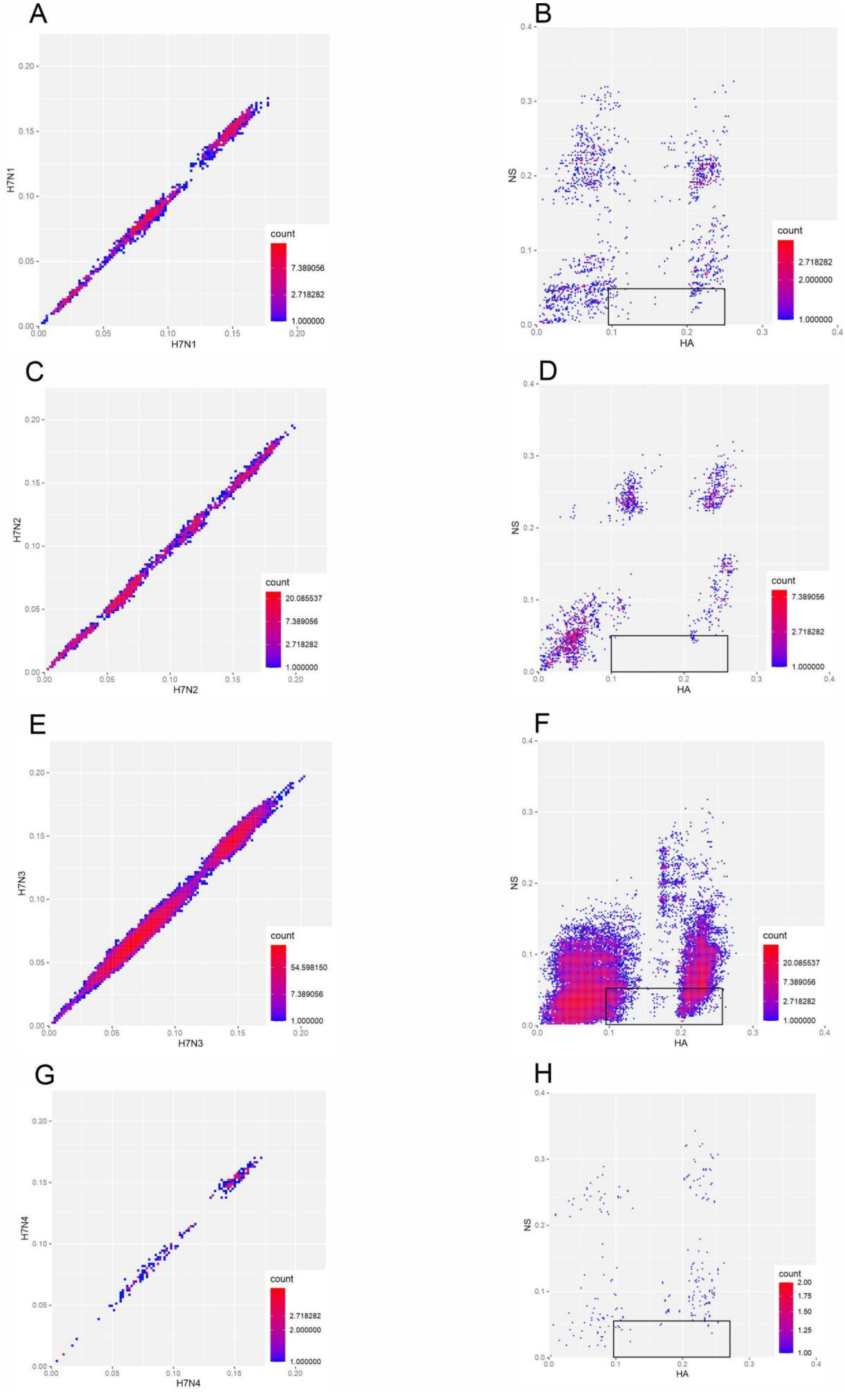
A
H7N1
H7N1
count
7.389056
2.718282
1.000000
B
NS
HA
count
2.718282
2.000000
1.000000
C
H7N2
H7N2
count
20.085537
7.389056
2.718282
1.000000
D
NS
HA
count
7.389056
2.718282
1.000000
E
H7N3
H7N3
count
54.598150
7.389056
1.000000
F
NS
HA
count
20.085537
7.389056
2.718282
1.000000
G
H7N4
H7N4
count
2.718282
2.000000
1.000000
H
NS
HA
count
2.00
1.75
1.50
1.25
1.00

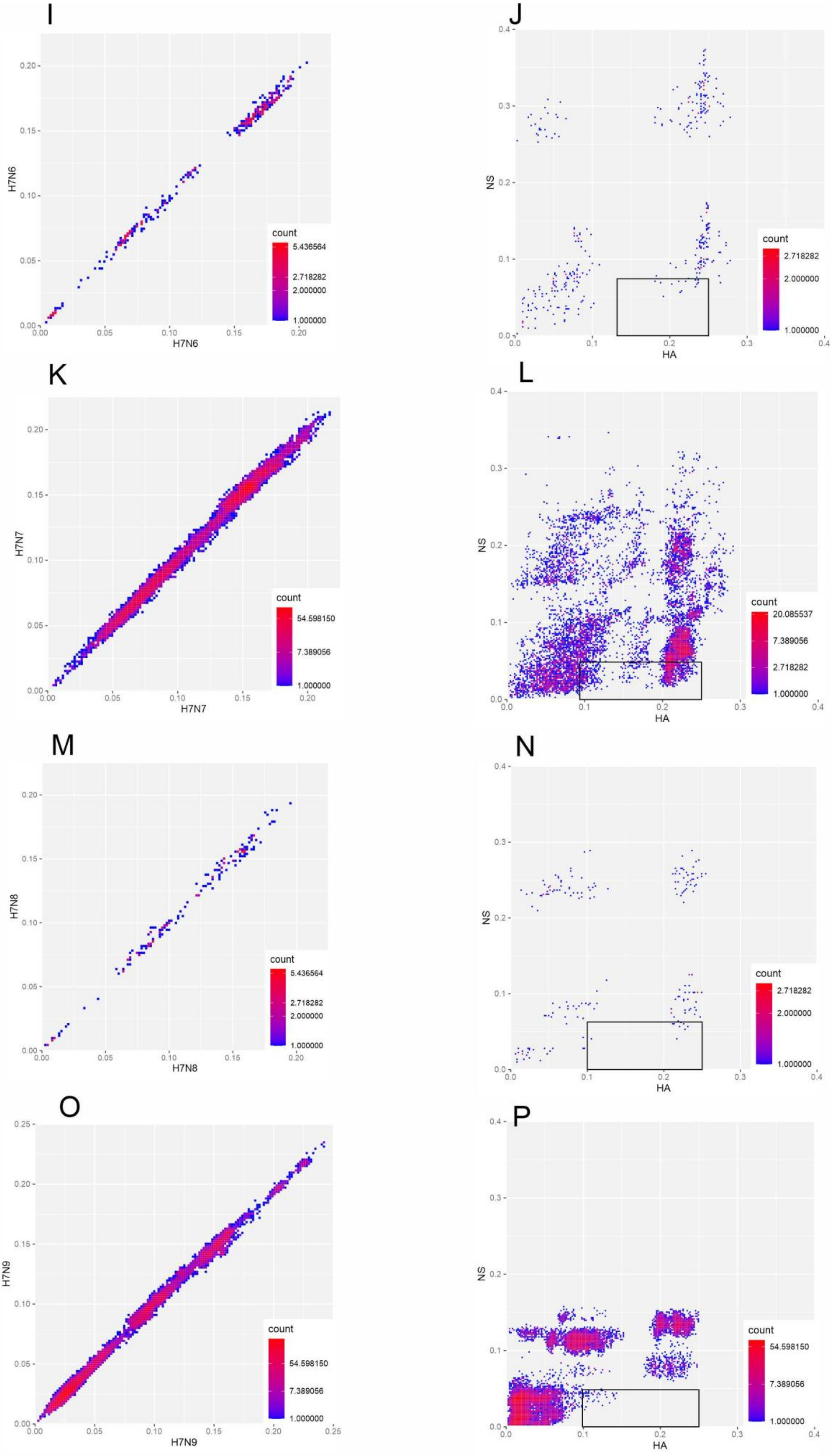

I
H7N6
count
5.436564
2.718282
2.000000
1.000000
J
NS
HA
count
2.718282
2.000000
1.000000
K
H7N7
count
54.598150
7.389056
1.000000
L
NS
HA
count
20.085537
7.389056
2.718282
1.000000
M
H7N8
count
5.436564
2.718282
2.000000
1.000000
N
NS
HA
count
2.718282
2.000000
1.000000
O
H7N9
count
54.598150
7.389056
1.000000
P
NS
HA
count
54.598150
7.389056
1.000000

Figure S10. Pairwise nucleotide distance comparison plots (PDCPs) showing phylogenetic incongruence between selected genetic regions of H7Nx influenza viruses. Each dot represents a pair of raw nucleotide distances between two sequences in two genomic regions (axis labeling). The count value indicates the number of virus pairs per bin by a color gradient. The region of subsequent analysis was highlighted with the black boxes. (**A**) PDCP constructed for the concatenated full genomic sequences of H7N1 viruses for which pairwise distances were calculated for even and odd sites (negative control); (**B**) PDCP constructed for NS and HA segments of H7N1 influenza viruses; (**C**) PDCP constructed for the concatenated full genomic sequences of H7N2 viruses for which pairwise distances were calculated for even and odd sites (negative control); (**D**) PDCP constructed for NS and HA segments of H7N2 influenza viruses; (**E**) PDCP constructed for the concatenated full genomic sequences of H7N3 viruses for which pairwise distances were calculated for even and odd sites (negative control); (**F**) PDCP constructed for NS and HA segments of H7N3 influenza viruses; (**G**) PDCP constructed for the concatenated full genomic sequences of H7N4 viruses for which pairwise distances were calculated for even and odd sites (negative control); (**H**) PDCP constructed for NS and HA segments of H7N4 influenza viruses; (**I**) PDCP constructed for the concatenated full genomic sequences of H7N6 viruses for which pairwise distances were calculated for even and odd sites (negative control); (**J**) PDCP constructed for NS and HA segments of H7N6 influenza viruses; (**K**) PDCP constructed for the concatenated full genomic sequences of H7N7 viruses for which pairwise distances were calculated for even and odd sites (negative control); (**L**) PDCP constructed for NS and HA segments of H7N7 influenza viruses; (**M**) PDCP constructed for the concatenated full genomic sequences of H7N8 viruses for which pairwise distances were calculated for even and odd sites (negative control); (**N**) PDCP constructed for NS and HA segments of H7N8 influenza viruses; (**O**) PDCP constructed for the concatenated full genomic sequences of H7N9 viruses for which pairwise distances were calculated for even and odd sites (negative control); (**P**) PDCP constructed for NS and HA segments of H7N9 influenza viruses.
