# Supplementary material for: Phylogenetic Insights into H7Nx Influenza Viruses: Uncovering Reassortment Patterns and Geographic Variability
Source: Viruses. 2024 Oct 23;16(11):1656. doi: 10.3390/v16111656 (PMC11598867; doi:10.3390/v16111656)
Supplement: Supplementary file 1 [file viruses-16-01656-s001.zip › Supplementary Figures S11-S18.pdf]

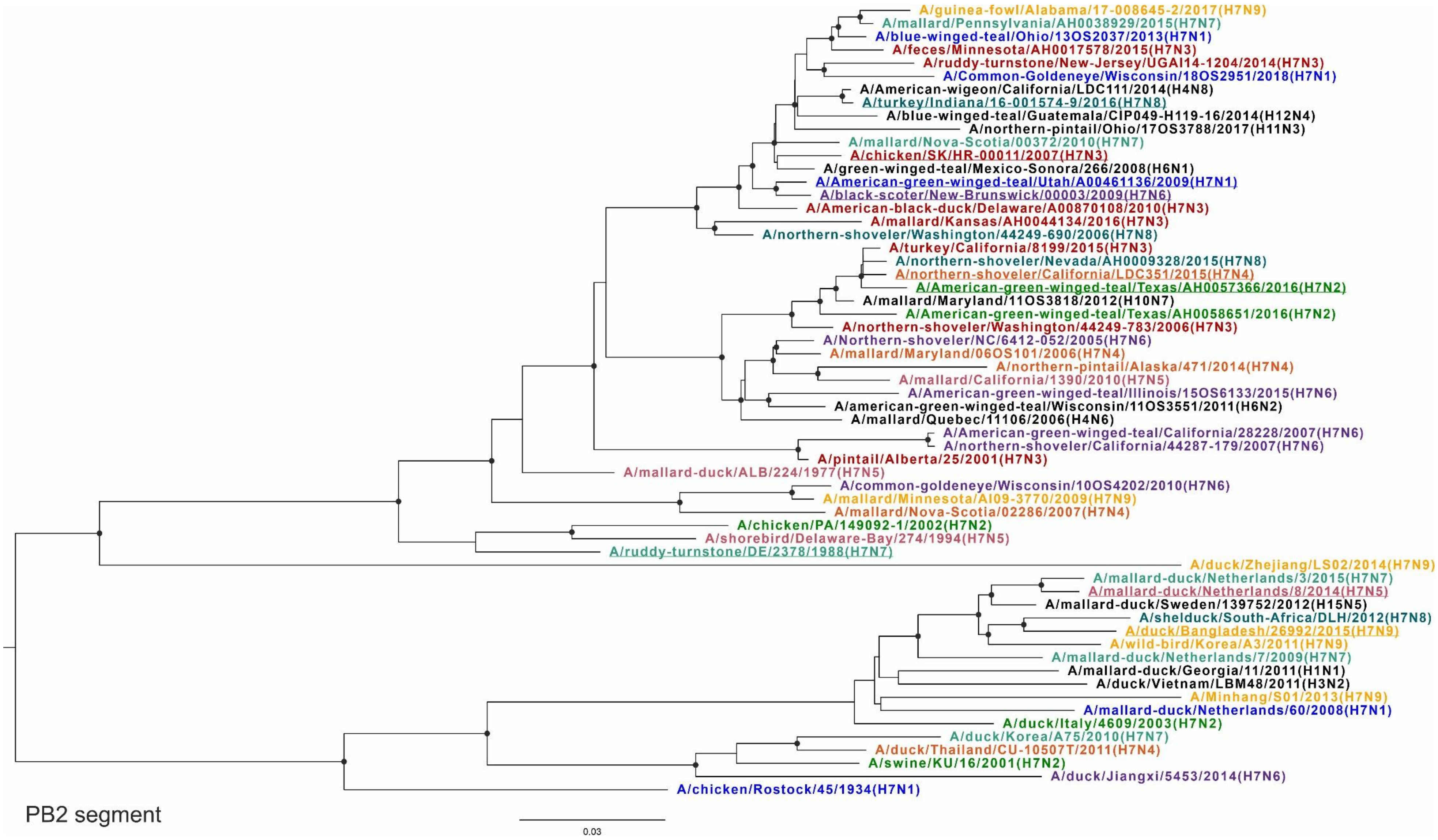

Supplementary Figure S11. Phylogenetic tree for PB2 segment. Each H7Nx subtype was color coded, other subtypes were indicated by black. Black circles indicate nodes that were supported by ultrafast bootstrap approximation values above 95%.

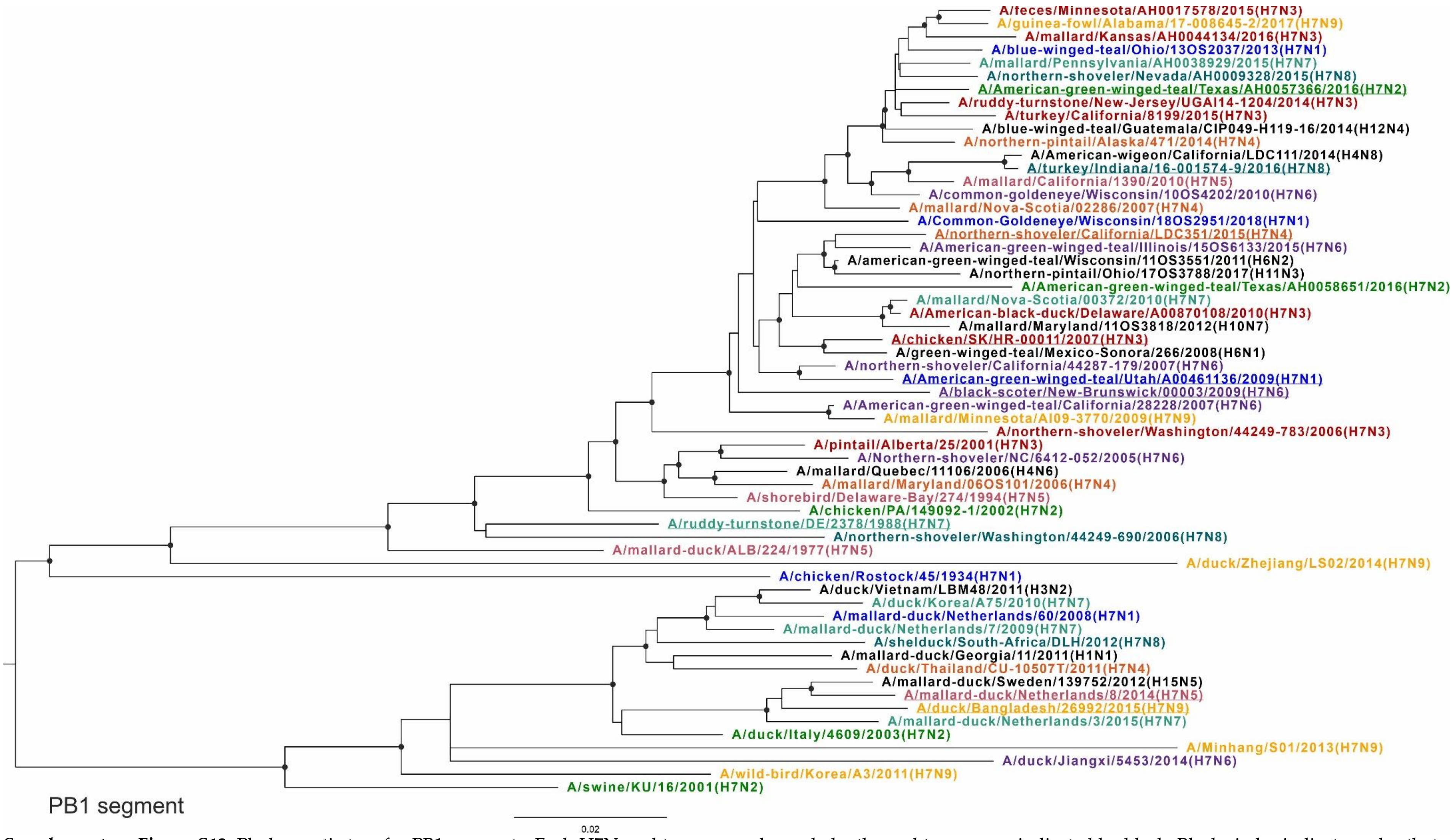

Supplementary Figure S12. Phylogenetic tree for PB1 segments. Each H7Nx subtype was color coded, other subtypes were indicated by black. Black circles indicate nodes that were supported by ultrafast bootstrap approximation values above 95%.

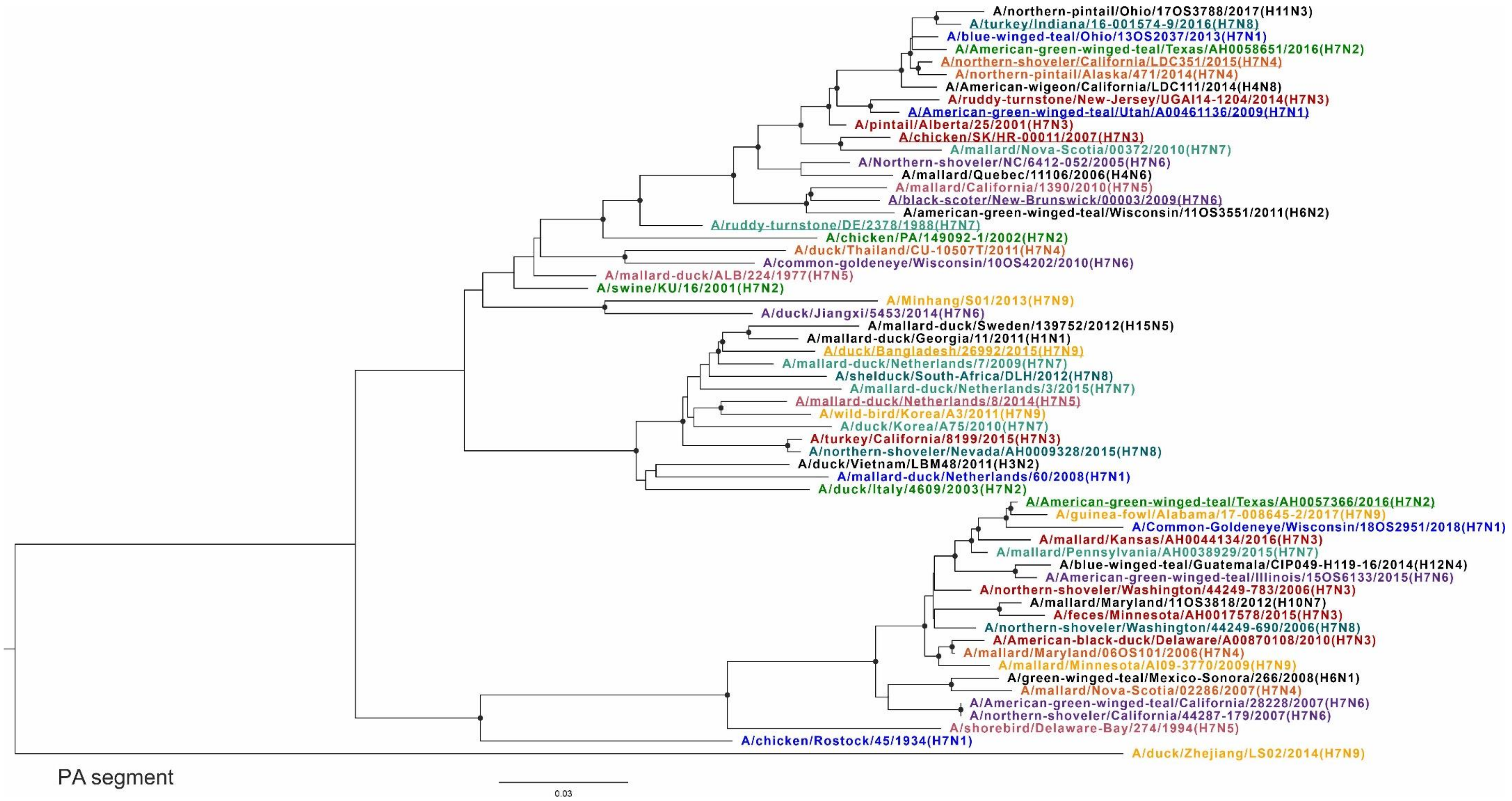

Supplementary Figure S13. Phylogenetic tree PA segments. Each H7Nx subtype was color coded, other subtypes were indicated by black. Black circles indicate nodes that were supported by ultrafast bootstrap approximation values above 95%.

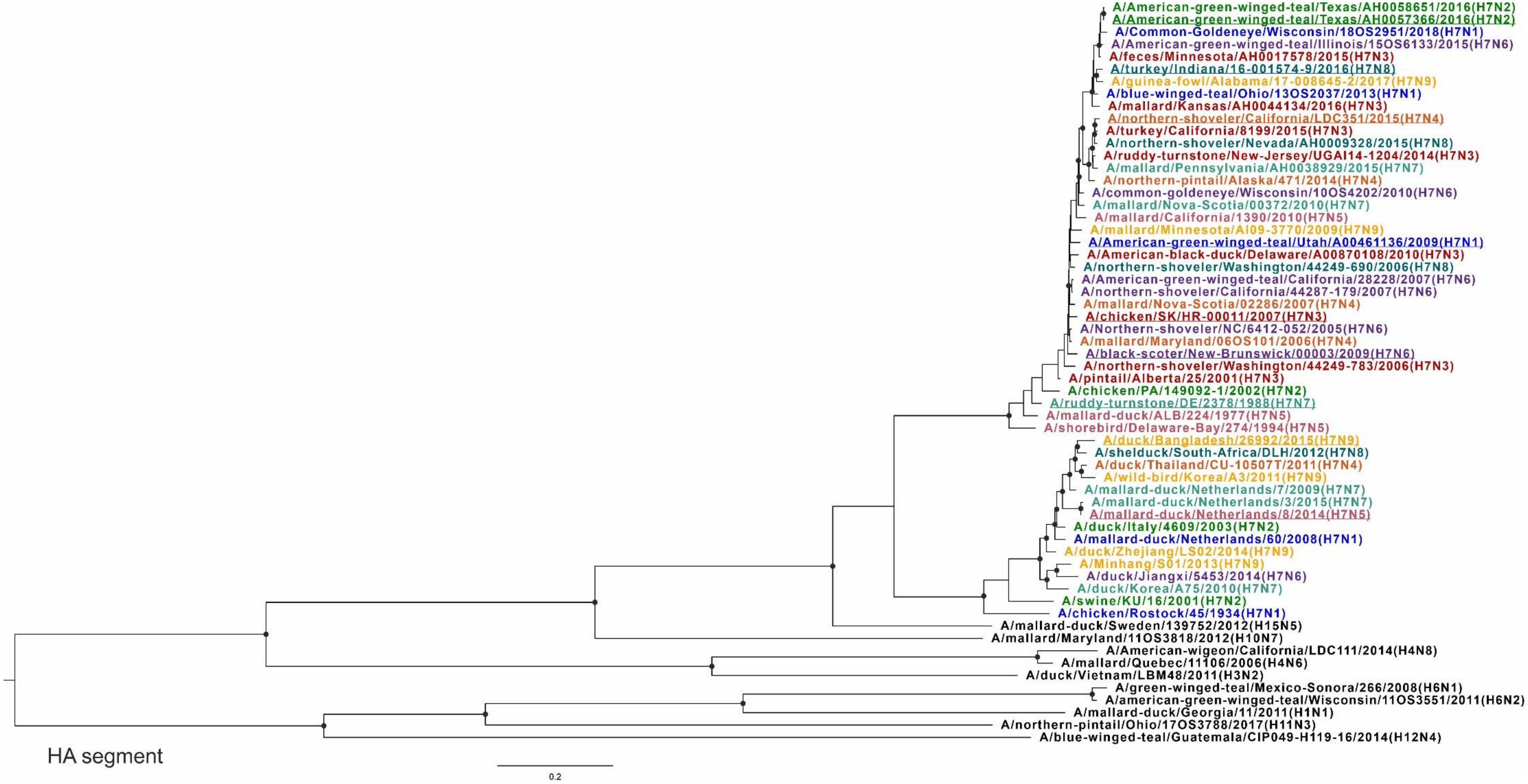

Supplementary Figure S14. Phylogenetic tree HA segments. Each H7Nx subtype was color coded, other subtypes were indicated by black. Black circles indicate nodes that were supported by ultrafast bootstrap approximation values above 95%.

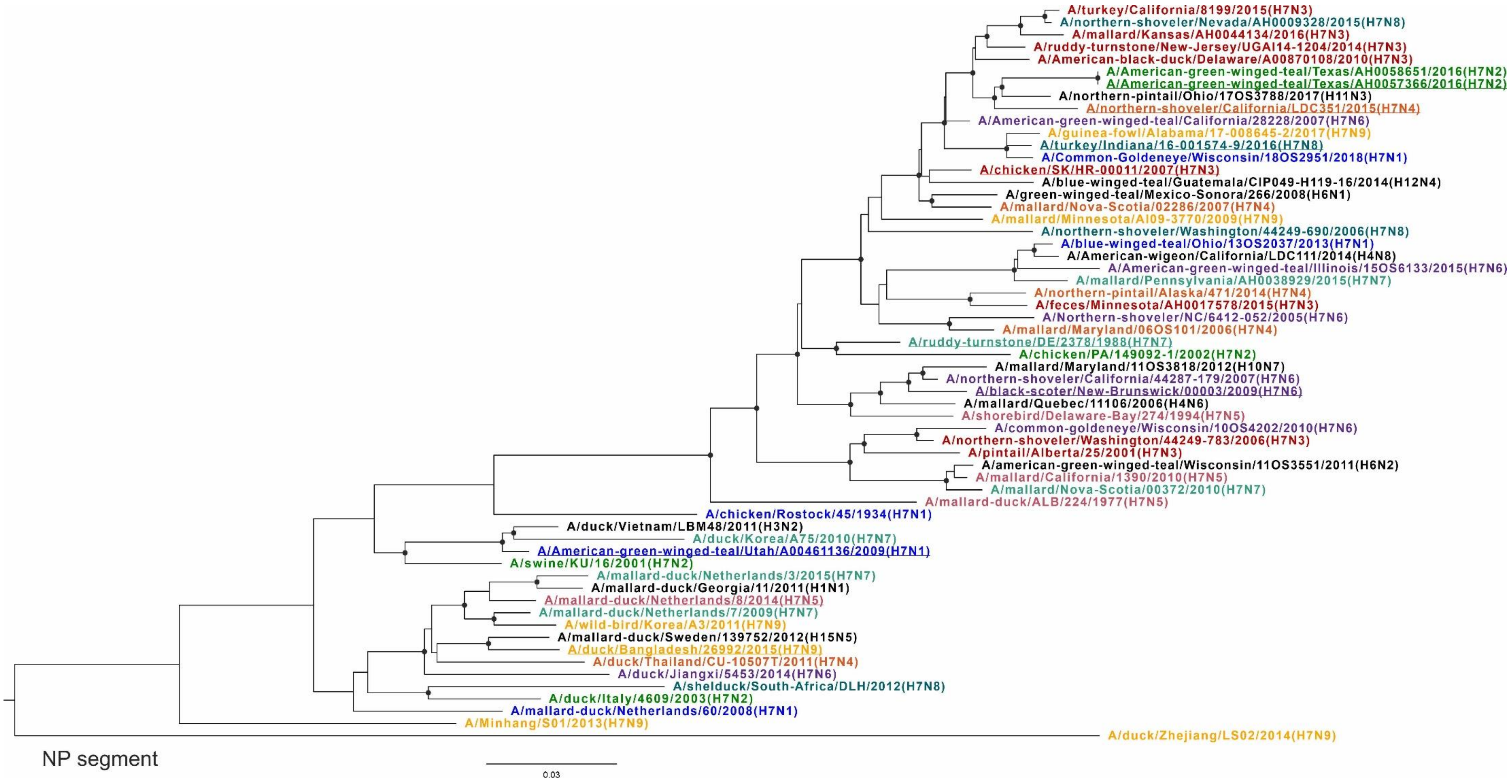

Supplementary Figure S15. Phylogenetic tree NP segments. Each H7Nx subtype was color coded, other subtypes were indicated by black. Black circles indicate nodes that were supported by ultrafast bootstrap approximation values above 95%.

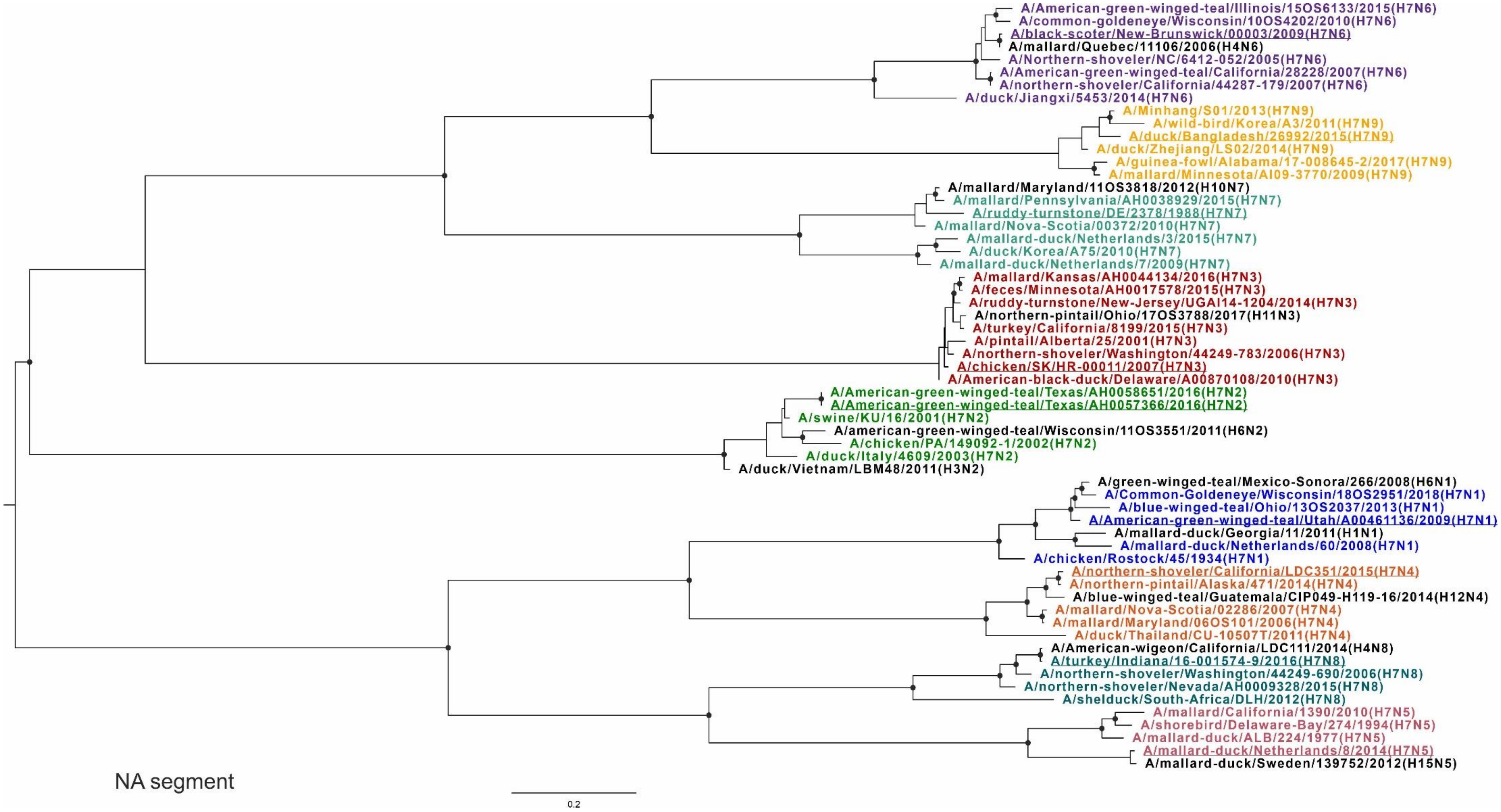

Supplementary Figure S16. Phylogenetic tree for NA segment. Each H7Nx subtype was color coded, other subtypes were indicated by black. Black circles indicate nodes that were supported by ultrafast bootstrap approximation values above 95%.

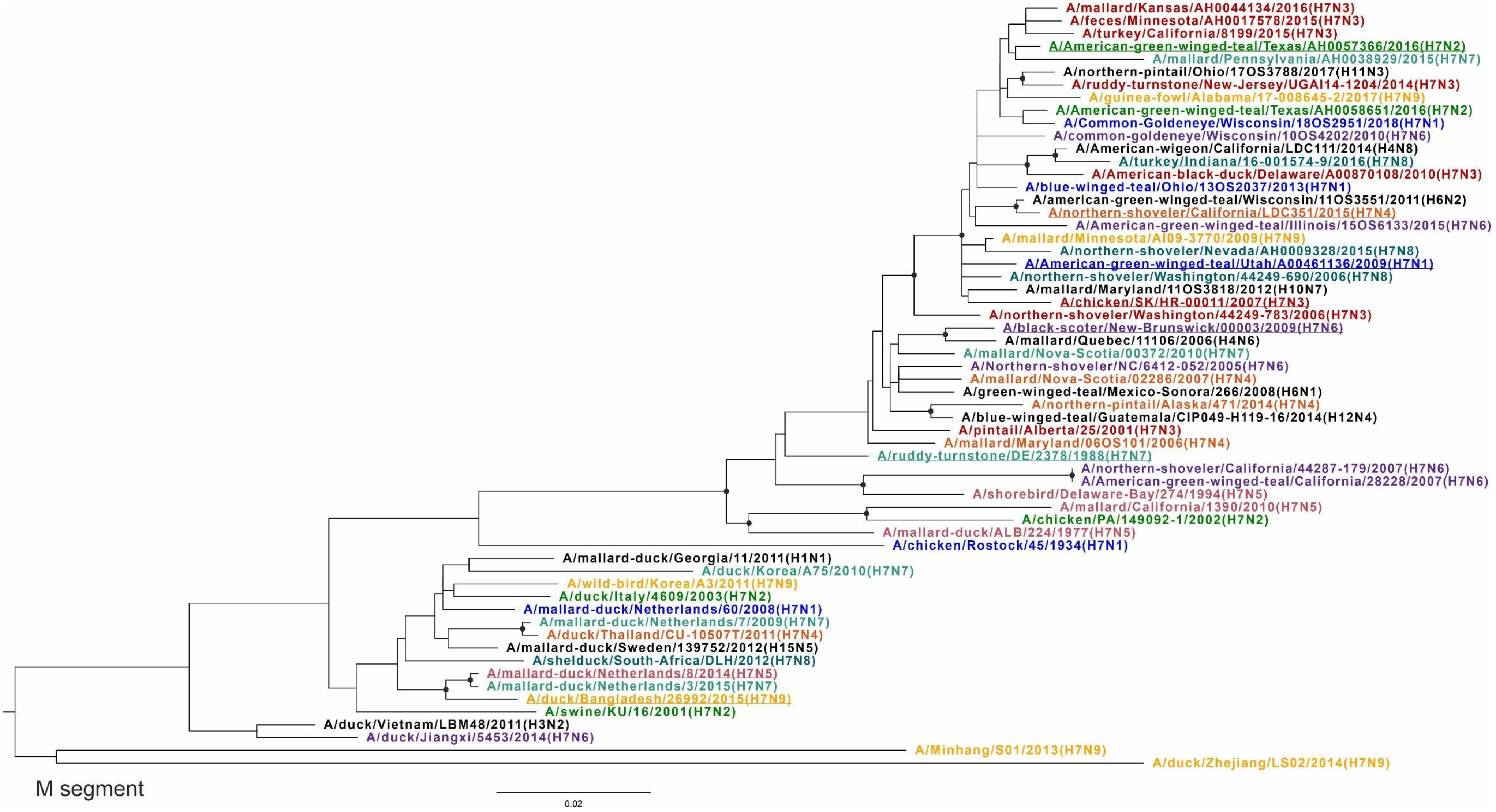

Supplementary Figure S17. Phylogenetic tree for M segments. Each H7Nx subtype was color coded, other subtypes were indicated by black. Black circles indicate nodes that were supported by ultrafast bootstrap approximation values above 95%.

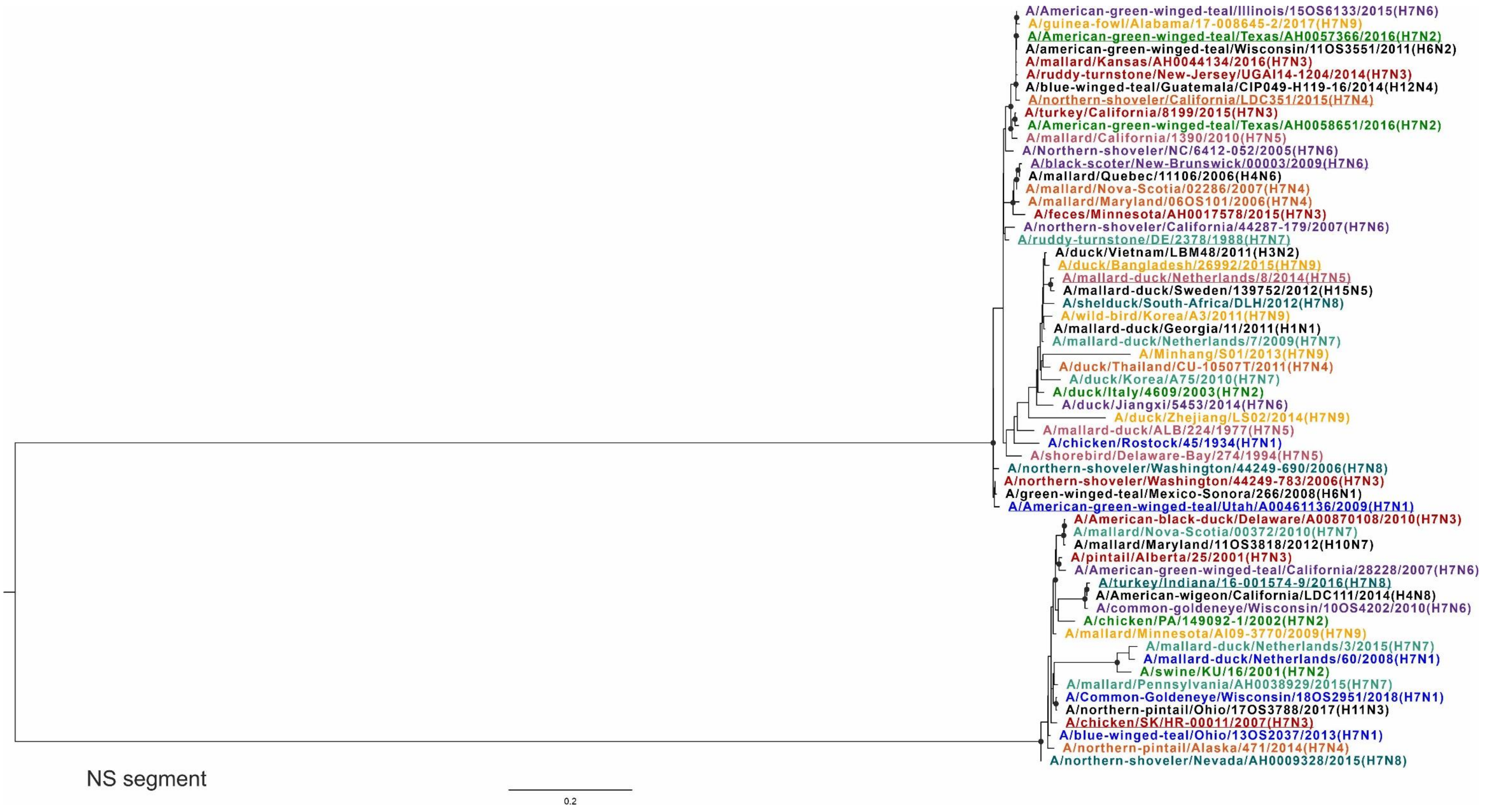

Supplementary Figure S18. Phylogenetic tree for NS segments. Each H7Nx subtype was color coded, other subtypes were indicated by black. Black circles indicate nodes that were supported by ultrafast bootstrap approximation values above 95%.
