# Supplementary material for: Phylogenetic Insights into H7Nx Influenza Viruses: Uncovering Reassortment Patterns and Geographic Variability
Source: Viruses. 2024 Oct 23;16(11):1656. doi: 10.3390/v16111656 (PMC11598867; doi:10.3390/v16111656)
Supplement: Supplementary file 1 [file viruses-16-01656-s001.zip › Supplementary Figures S1-S9.pdf]

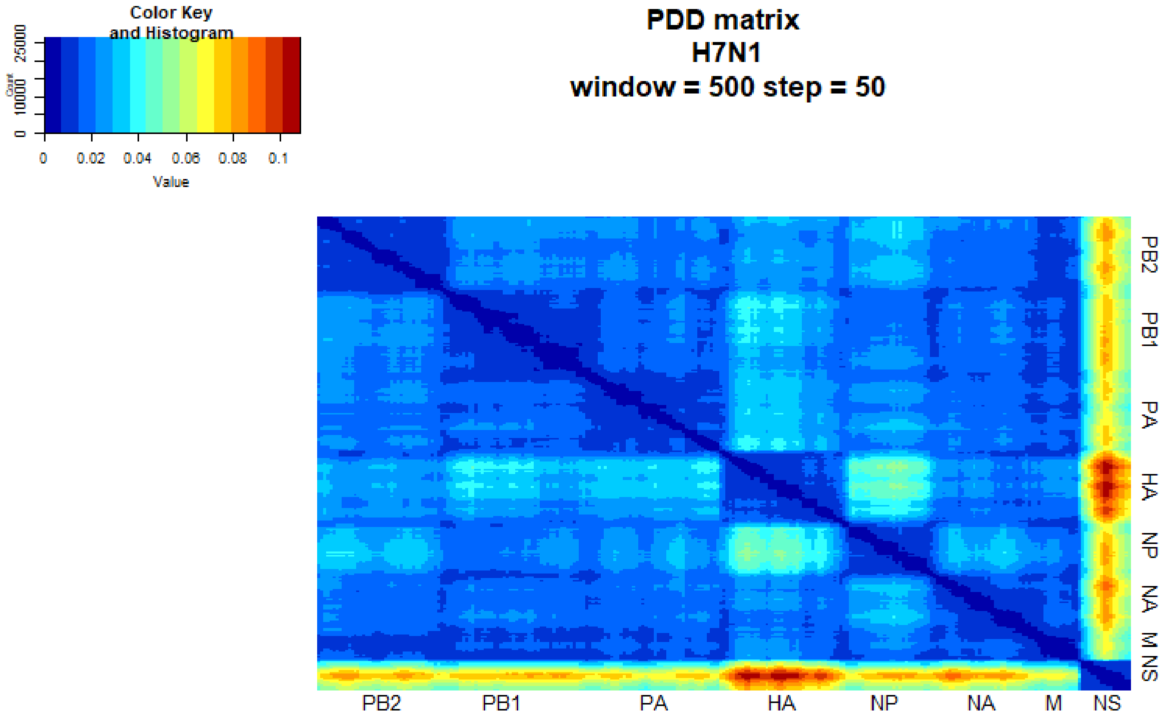
Color Key
and Histogram
Count
0
10000
25000
0
0.02
0.04
0.06
0.08
0.1
Value
PDD matrix
H7N1
window = 500 step = 50
PB2
PB1
PA
HA
NP
NA
M NS
PB2
PB1
PA
HA
NP
NA
M
NS

(a)

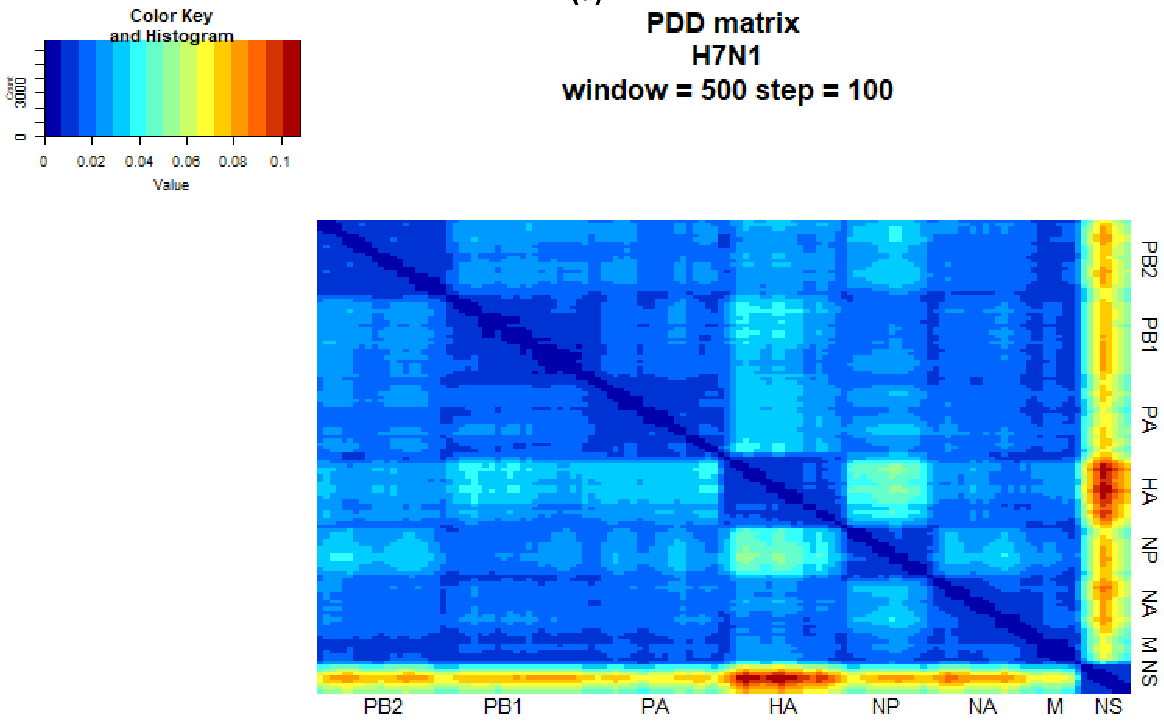
Color Key
and Histogram
Count
0
3000
0
0.02
0.04
0.06
0.08
0.1
Value
PDD matrix
H7N1
window = 500 step = 100
PB2
PB1
PA
HA
NP
NA
M NS
PB2
PB1
PA
HA
NP
NA
M
NS

(b)

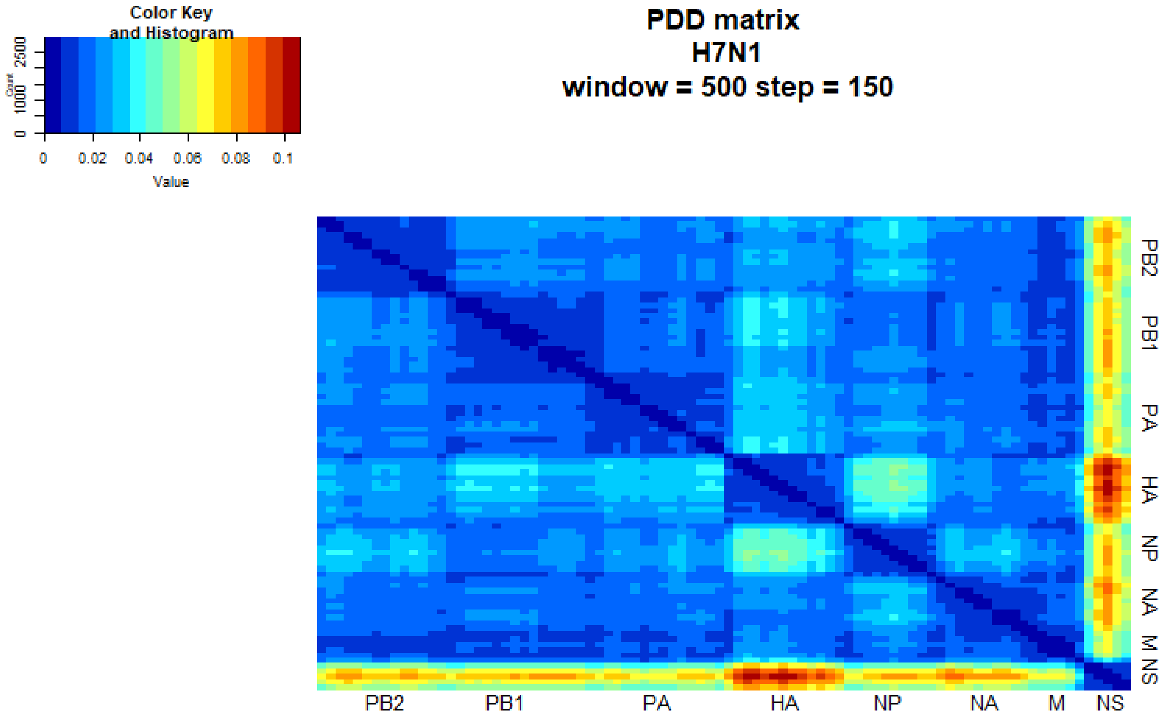

Color Key
and Histogram
Count
0
1000
2500
0
0.02
0.04
0.06
0.08
0.1
Value
PDD matrix
H7N1
window = 500 step = 150
PB2
PB1
PA
HA
NP
NA
M
NS

(c)

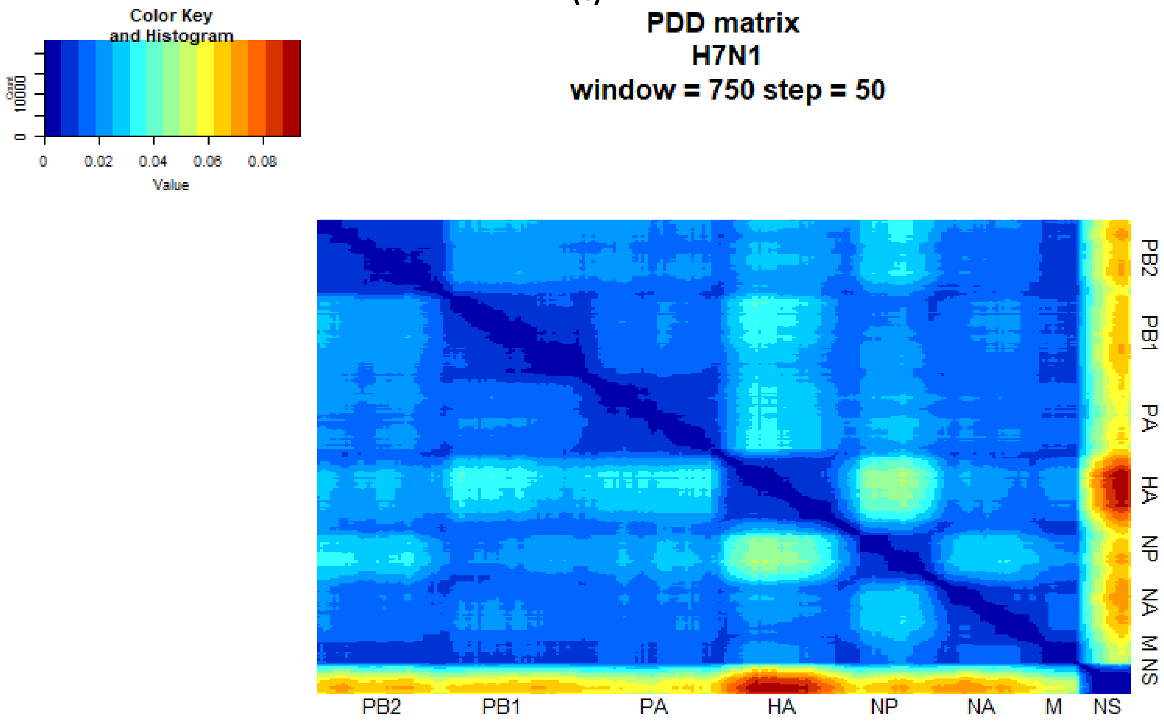

Color Key
and Histogram
Count
0
10000
0
0.02
0.04
0.06
0.08
Value
PDD matrix
H7N1
window = 750 step = 50
PB2
PB1
PA
HA
NP
NA
M
NS

(d)

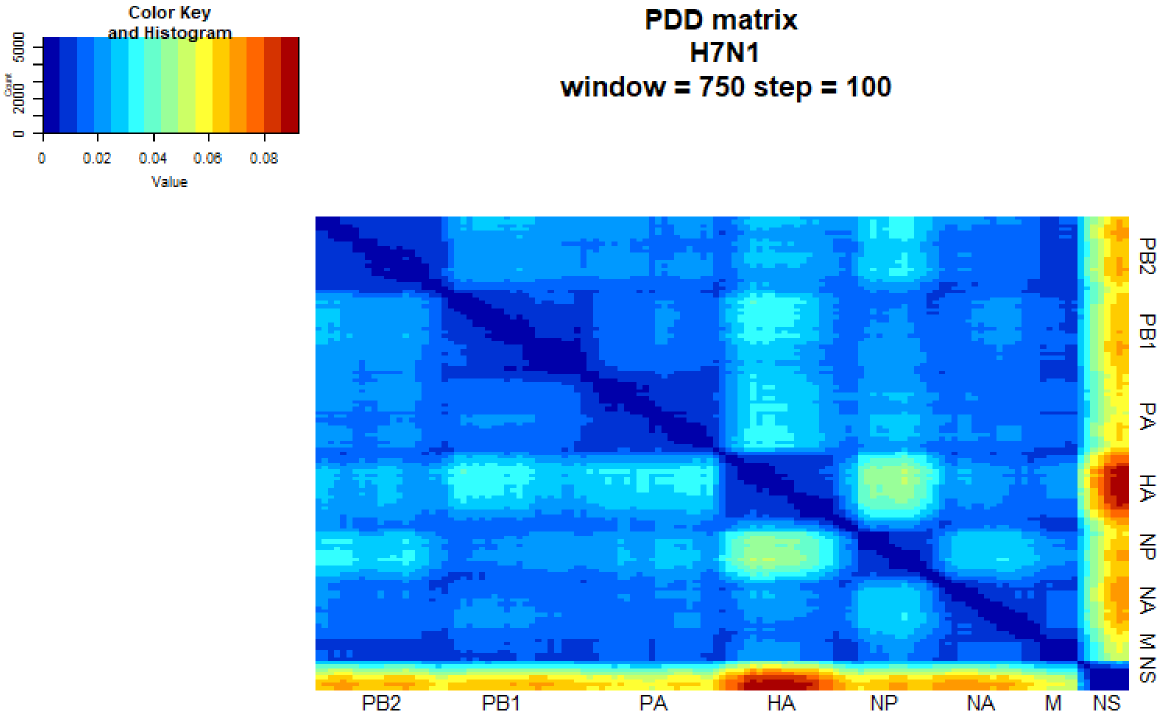
Color Key
and Histogram
Count
0
2000
5000
0
0.02
0.04
0.06
0.08
Value
PDD matrix
H7N1
window = 750 step = 100
PB2
PB1
PA
HA
NP
NA
M
NS
PB2
PB1
PA
HA
NP
NA
M NS

(e)

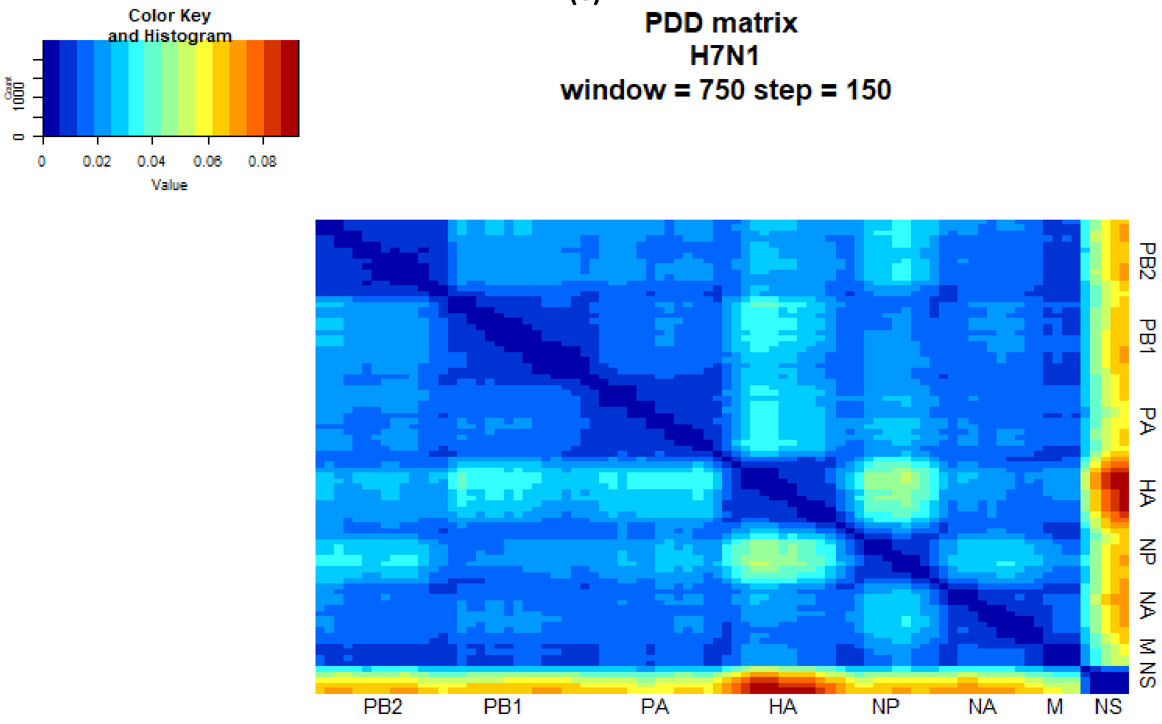
Color Key
and Histogram
Count
0
1000
0
0.02
0.04
0.06
0.08
Value
PDD matrix
H7N1
window = 750 step = 150
PB2
PB1
PA
HA
NP
NA
M
NS
PB2
PB1
PA
HA
NP
NA
M NS

(f)

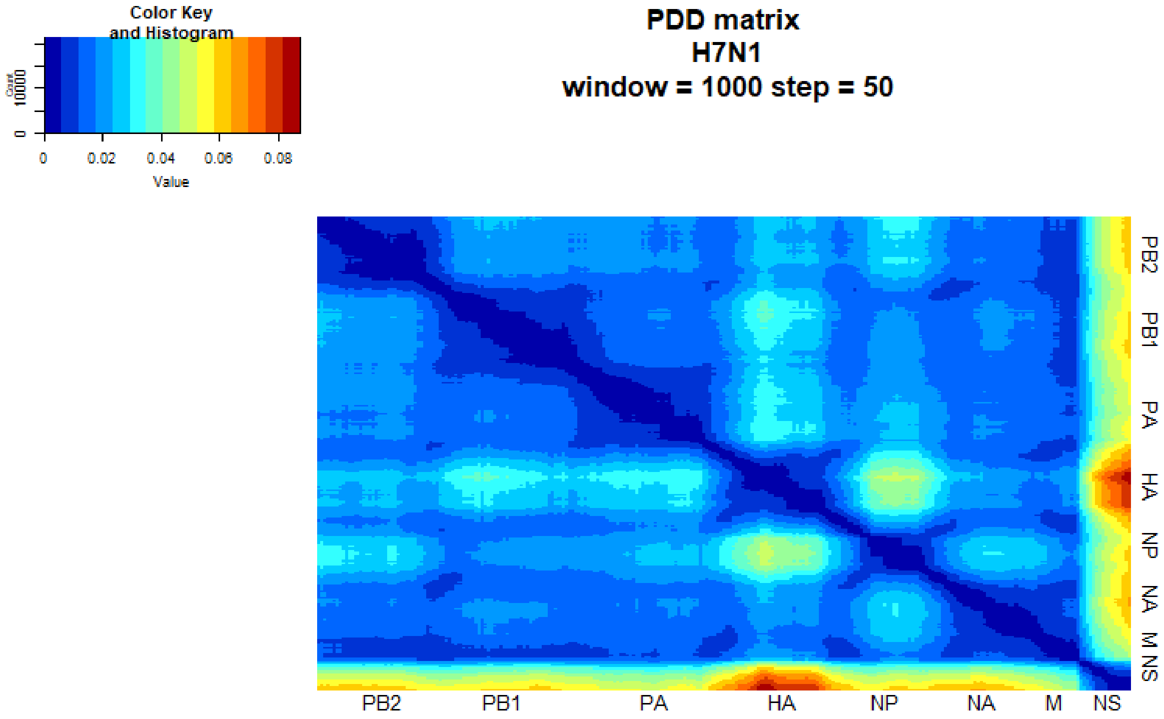
Color Key
and Histogram
Count
0
10000
0
0.02
0.04
0.06
0.08
Value
PDD matrix
H7N1
window = 1000 step = 50
PB2
PB1
PA
HA
NP
NA
M
NS

(g)

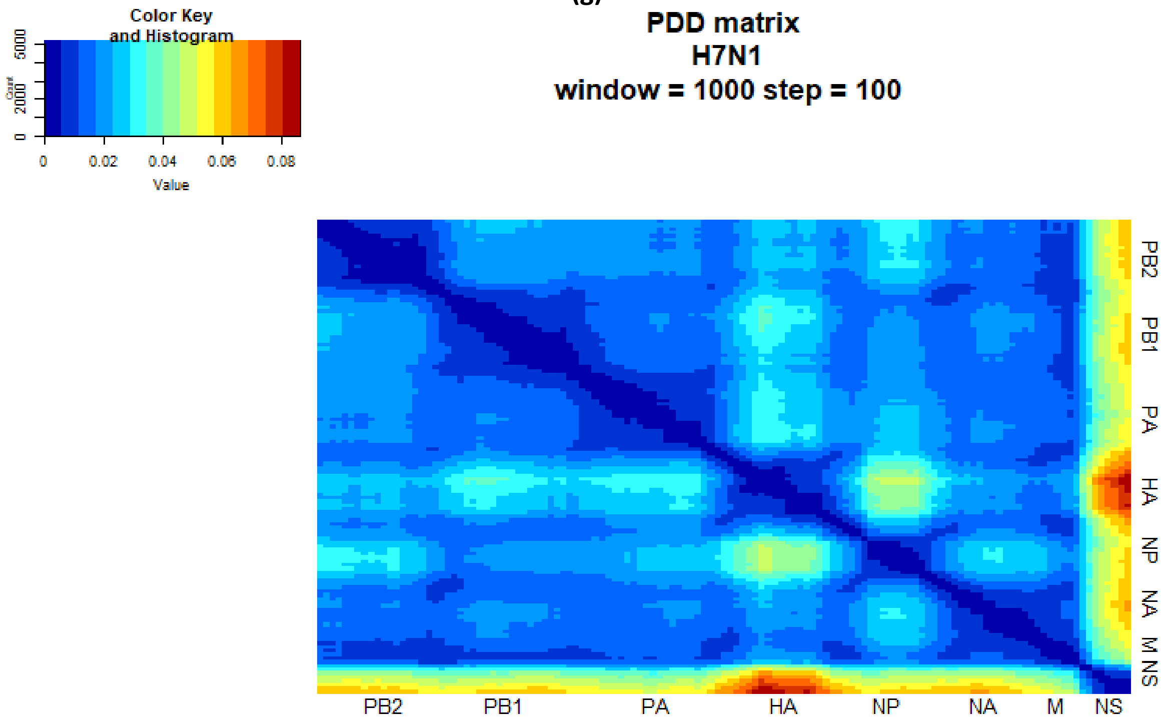
Color Key
and Histogram
Count
0
2000
5000
0
0.02
0.04
0.06
0.08
Value
PDD matrix
H7N1
window = 1000 step = 100
PB2
PB1
PA
HA
NP
NA
M
NS

(h)

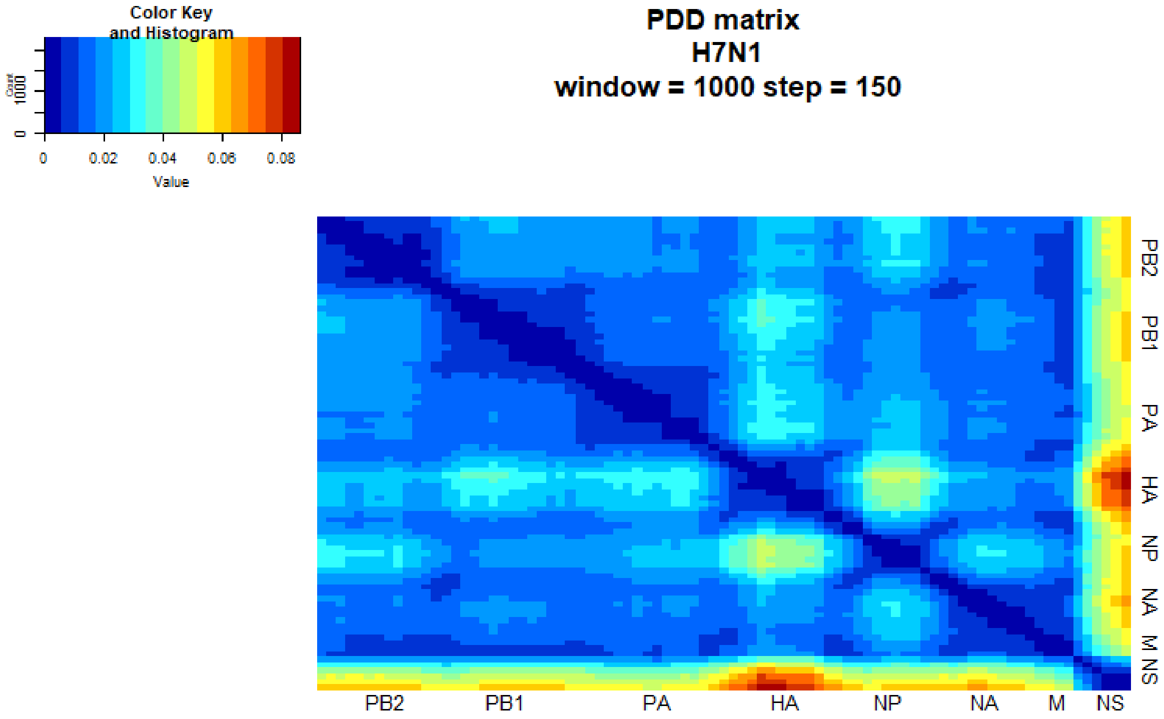

(i)

Supplementary Figure S1. Pairwise distance divergence matrix (PDDM) for H7N1 influenza viruses **(a)** Window = 500 nucleotides, Step = 50 nucleotides **(b)** Window = 500 nucleotides, Step = 100 nucleotides **(c)** Window = 500 nucleotides, Step = 150 nucleotides **(d)** Window = 750 nucleotides, Step = 50 nucleotides **(e)** Window = 750 nucleotides, Step = 100 nucleotides **(f)** Window = 750 nucleotides, Step = 150 nucleotides **(g)** Window = 1000 nucleotides, Step = 50 nucleotides **(h)** Window = 1000 nucleotides, Step = 100 nucleotides **(I)** Window = 1000 nucleotides, Step = 150 nucleotides . The color gradient scale shows the root mean square error (RMSE) values in PDCP is a figure.

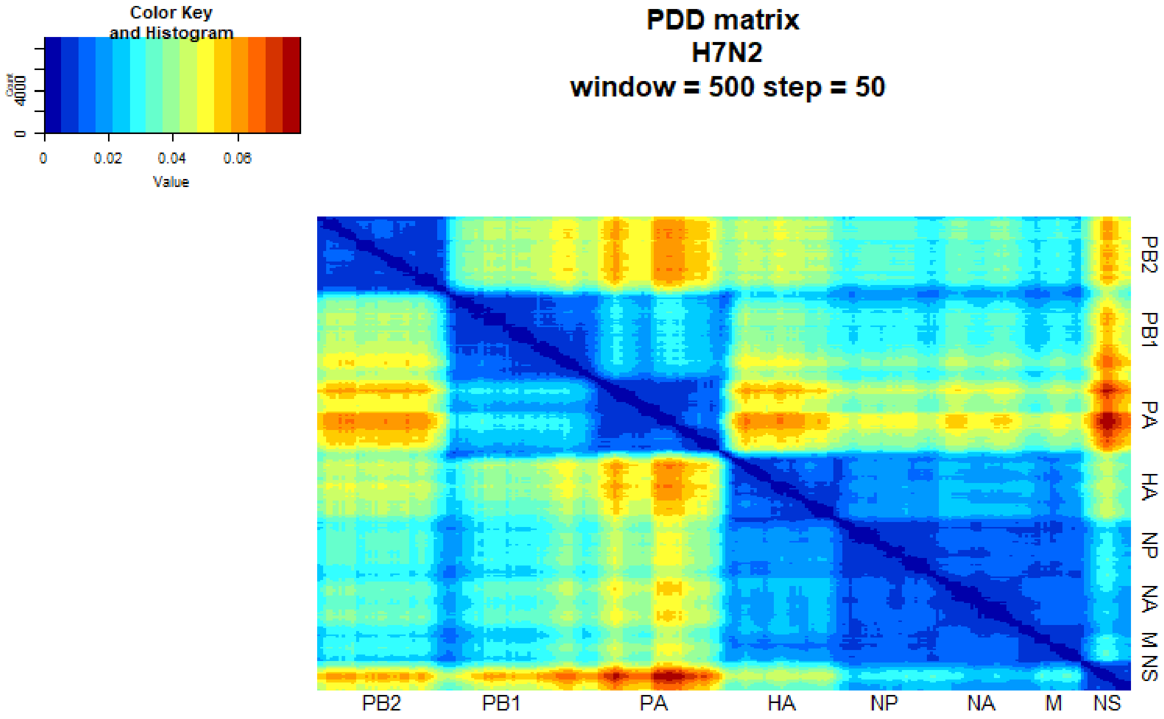
Color Key
and Histogram
Count
0
4000
0
0.02
0.04
0.06
Value
PDD matrix
H7N2
window = 500 step = 50
PB2
PB1
PA
HA
NP
NA
M
NS
PB2
PB1
PA
HA
NP
NA
M NS

(a)

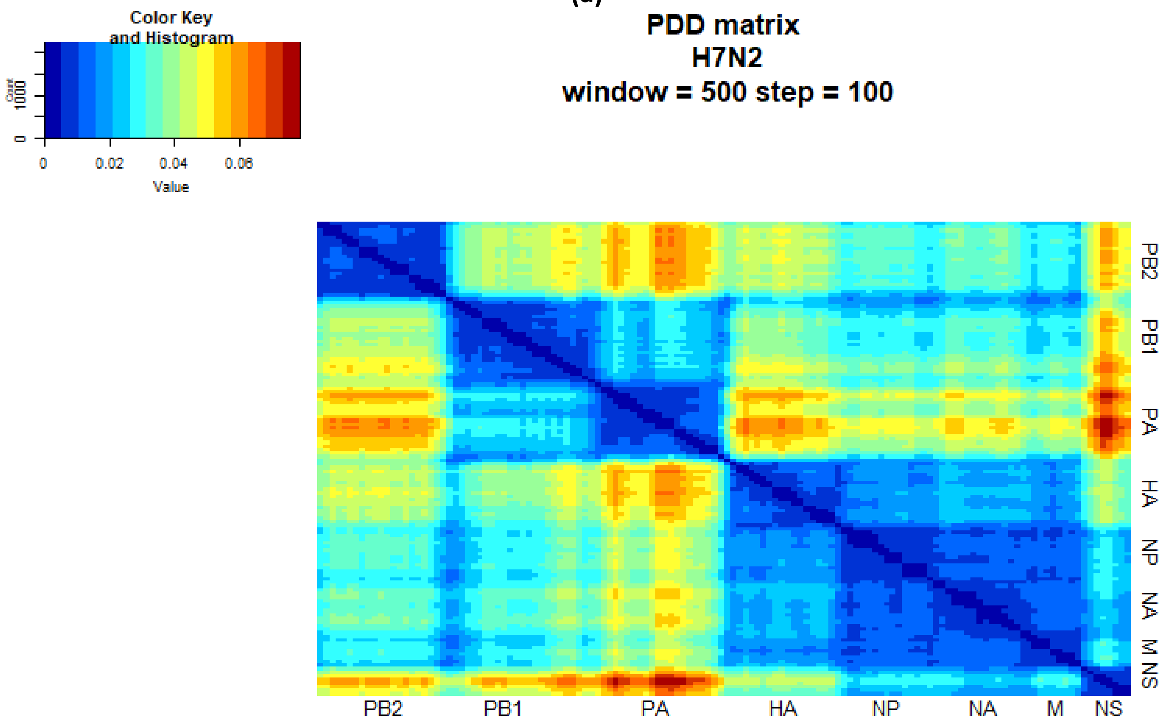
Color Key
and Histogram
Count
0
1000
0
0.02
0.04
0.06
Value
PDD matrix
H7N2
window = 500 step = 100
PB2
PB1
PA
HA
NP
NA
M
NS
PB2
PB1
PA
HA
NP
NA
M NS

(b)

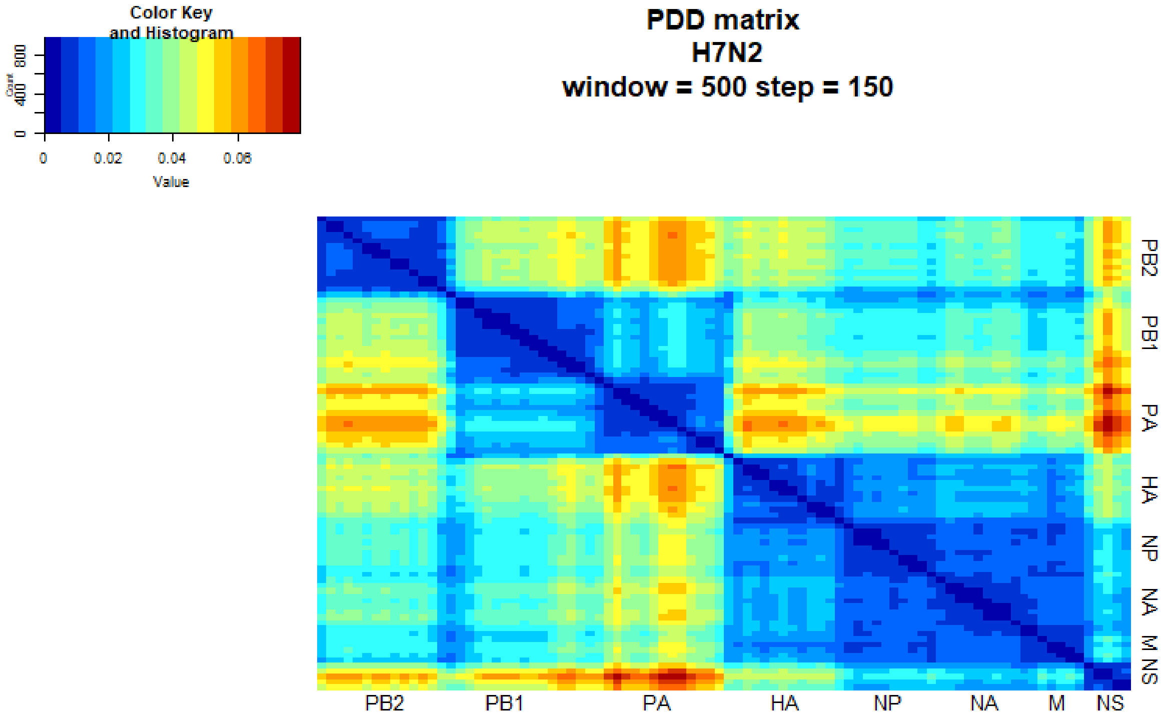
Color Key
and Histogram
Count
0
400
800
0
0.02
0.04
0.06
Value
PDD matrix
H7N2
window = 500 step = 150
PB2
PB1
PA
HA
NP
NA
M
NS

(c)

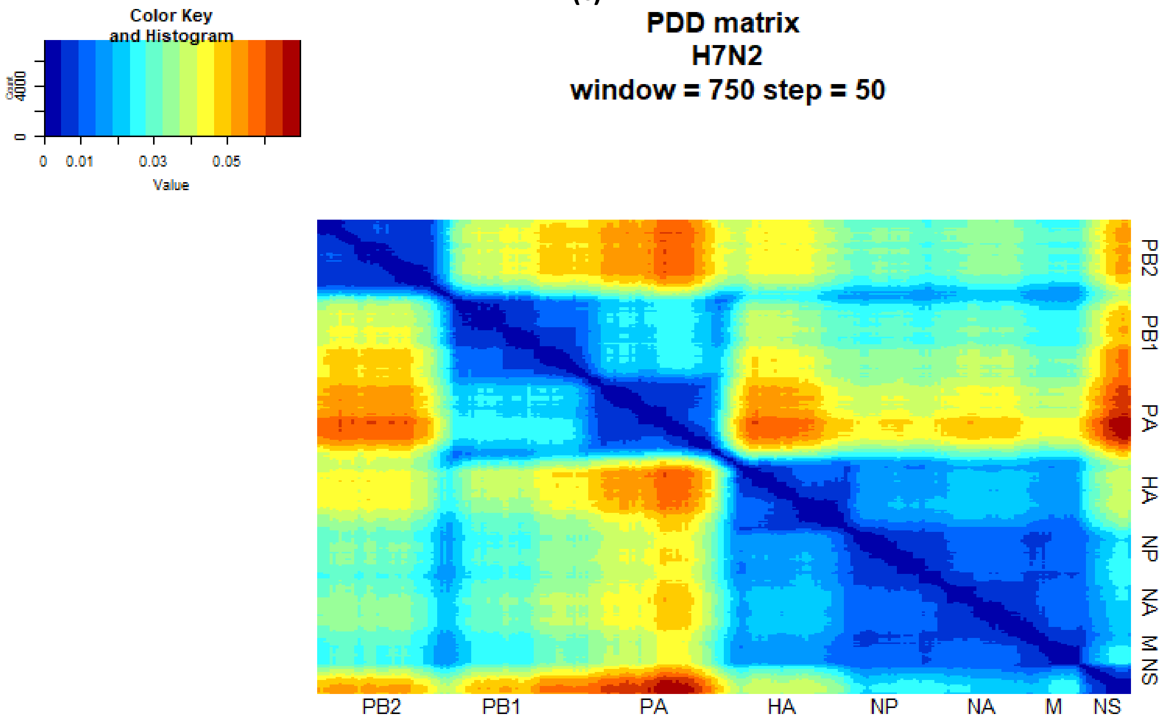
Color Key
and Histogram
Count
0
4000
0
0.01
0.03
0.05
Value
PDD matrix
H7N2
window = 750 step = 50
PB2
PB1
PA
HA
NP
NA
M
NS

(d)

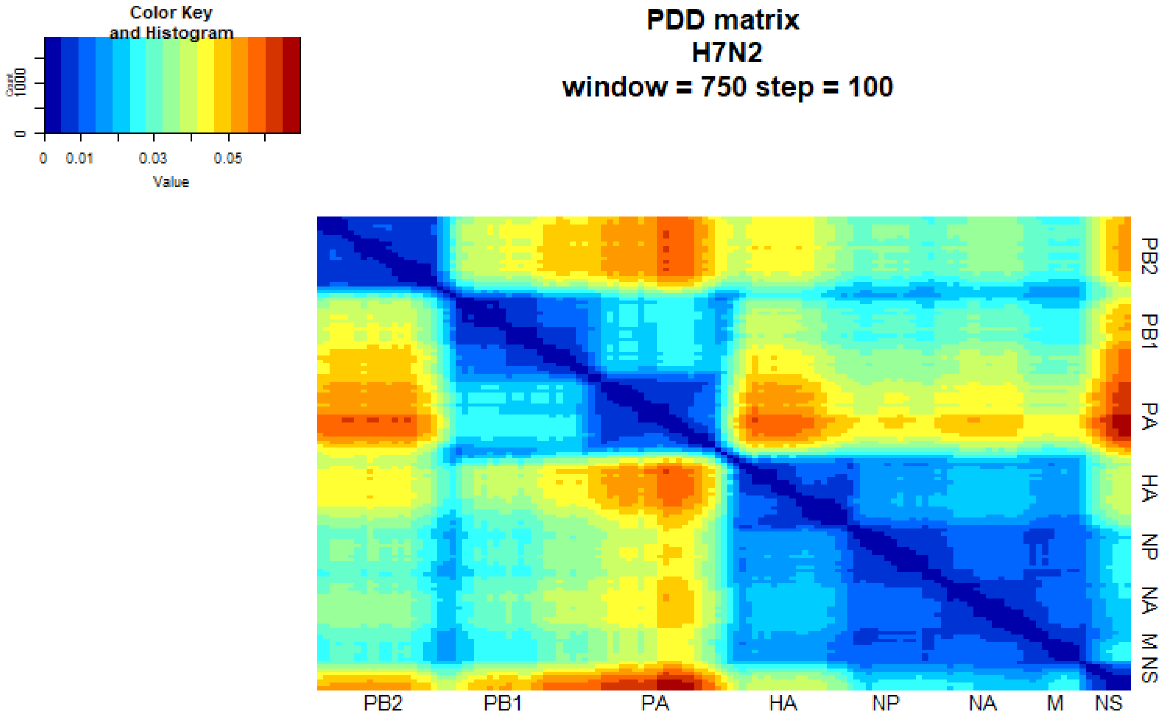
Color Key
and Histogram
Count
0
1000
0.01
0.03
0.05
Value
PDD matrix
H7N2
window = 750 step = 100
PB2
PB1
PA
HA
NP
NA
M
NS

(e)

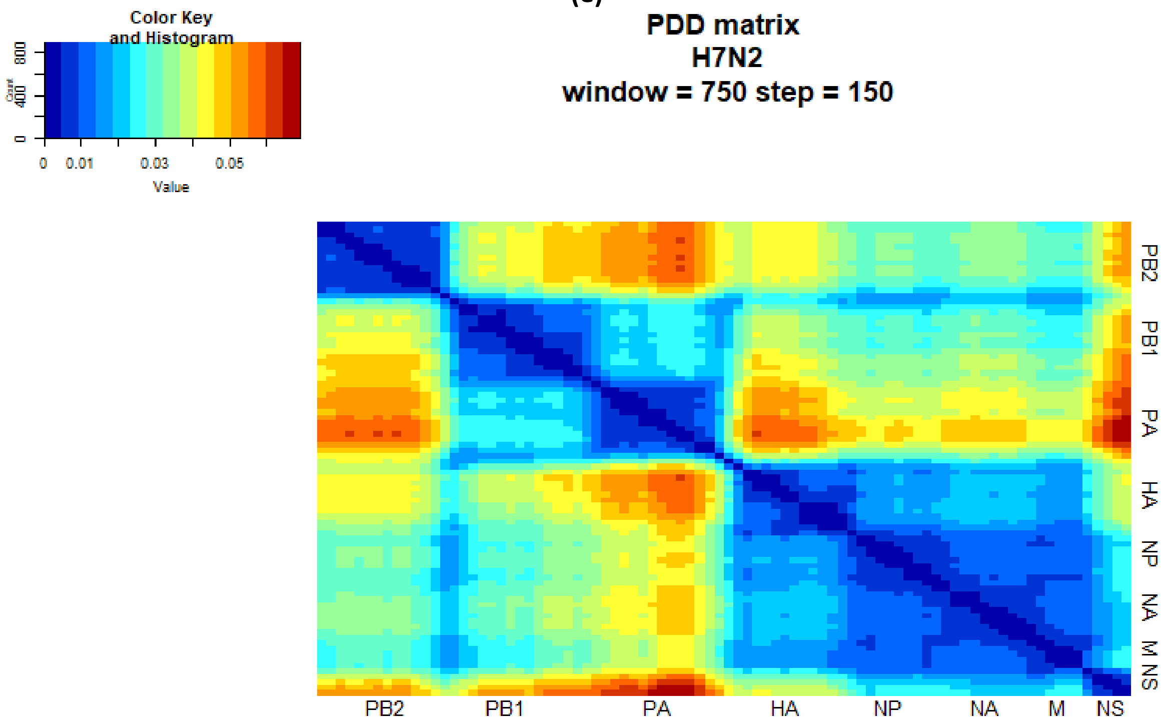
Color Key
and Histogram
Count
0
400
800
0.01
0.03
0.05
Value
PDD matrix
H7N2
window = 750 step = 150
PB2
PB1
PA
HA
NP
NA
M
NS

(f)

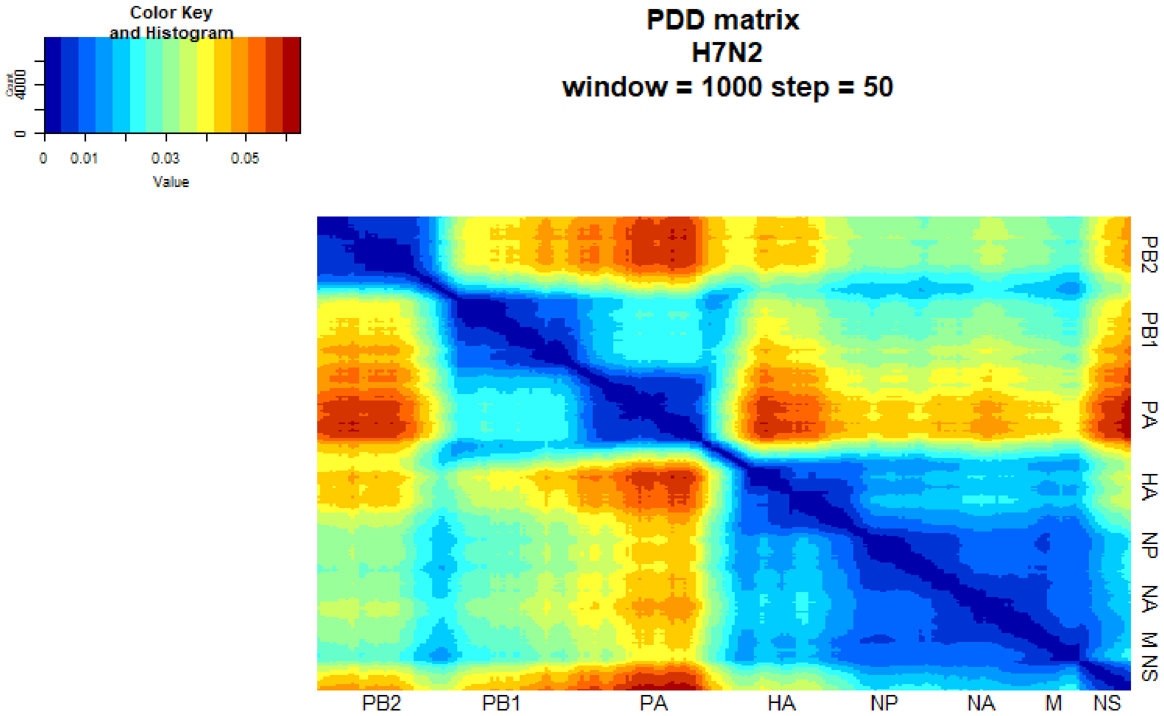
Color Key
and Histogram
Count
0
4000
0
0.01
0.03
0.05
Value
PDD matrix
H7N2
window = 1000 step = 50
PB2
PB1
PA
HA
NP
NA
M
NS
PB2
PB1
PA
HA
NP
NA
M NS

(g)

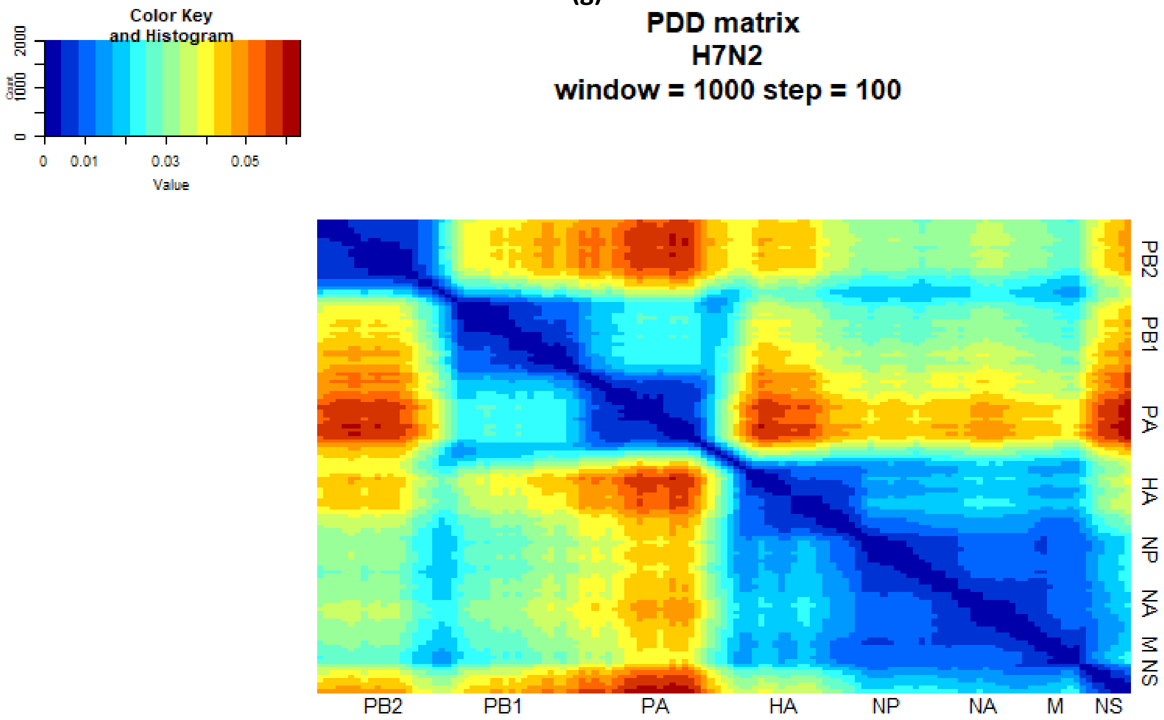
Color Key
and Histogram
Count
0
1000
2000
0
0.01
0.03
0.05
Value
PDD matrix
H7N2
window = 1000 step = 100
PB2
PB1
PA
HA
NP
NA
M
NS
PB2
PB1
PA
HA
NP
NA
M NS

(h)

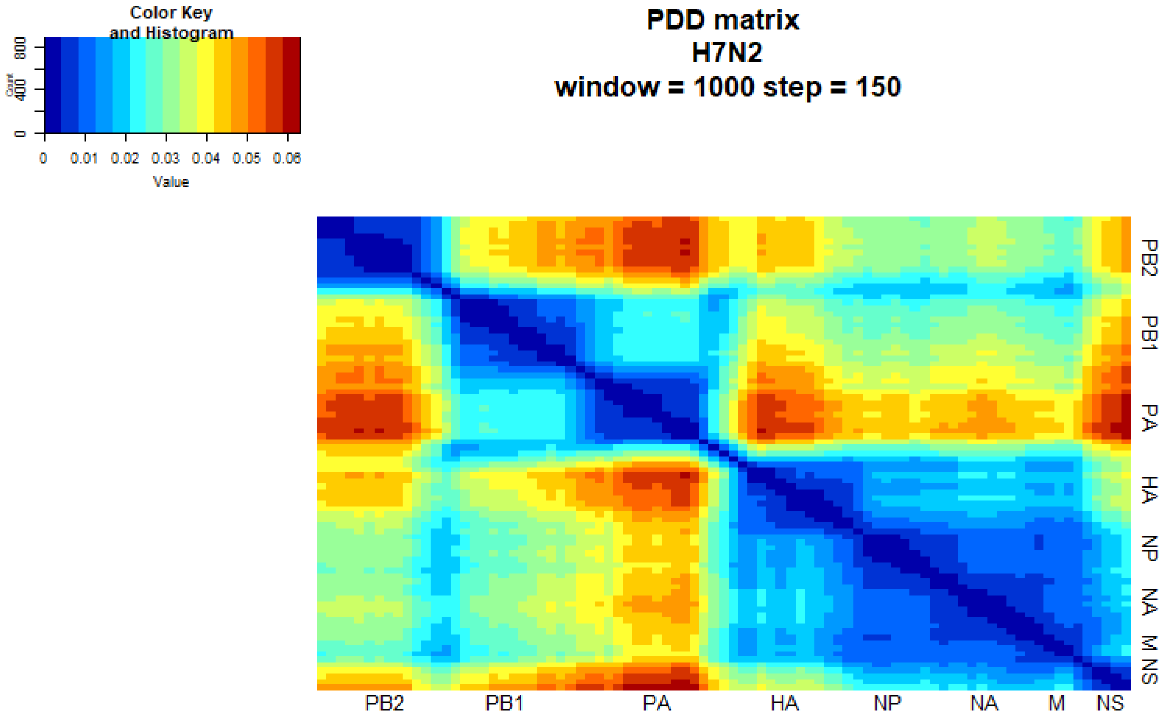

(i)

Supplementary Figure S2. Pairwise distance divergence matrix (PDDM) for H7N2 influenza viruses **(a)** Window = 500 nucleotides, Step = 50 nucleotides **(b)** Window = 500 nucleotides, Step = 100 nucleotides **(c)** Window = 500 nucleotides, Step = 150 nucleotides **(d)** Window = 750 nucleotides, Step = 50 nucleotides **(e)** Window = 750 nucleotides, Step = 100 nucleotides **(f)** Window = 750 nucleotides, Step = 150 nucleotides **(g)** Window = 1000 nucleotides, Step = 50 nucleotides **(h)** Window = 1000 nucleotides, Step = 100 nucleotides **(I)** Window = 1000 nucleotides, Step = 150 nucleotides. The color gradient scale shows the root mean square error (RMSE) values in PDCP is a figure.

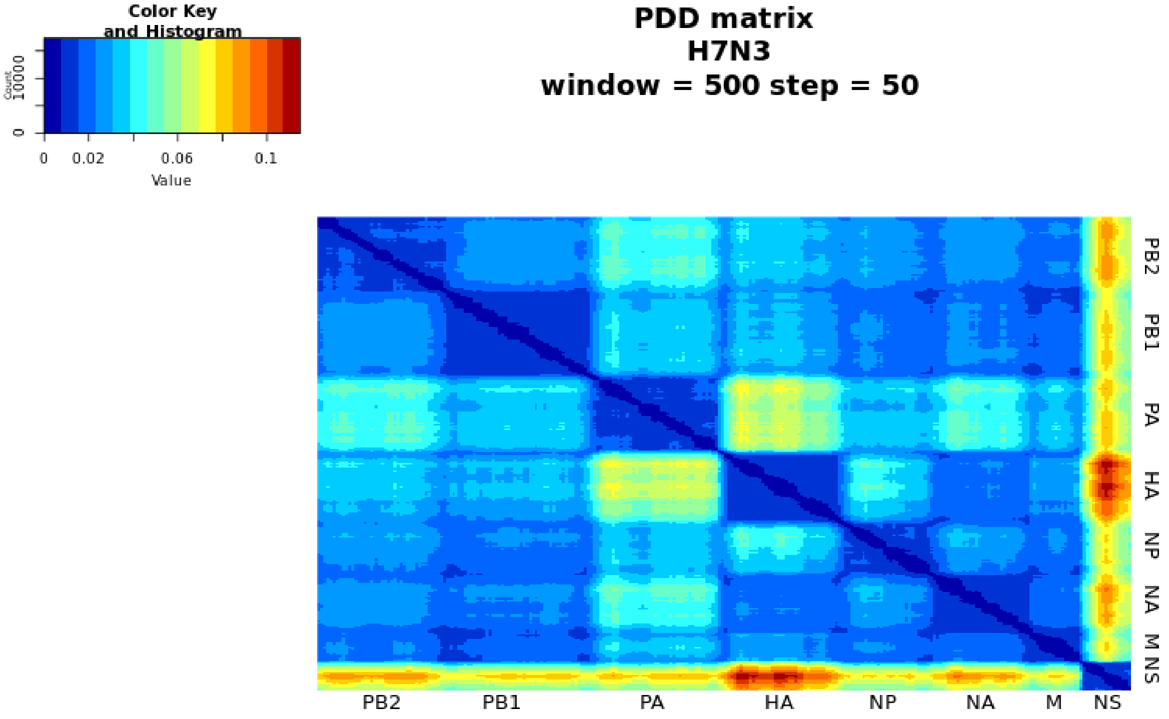
Color Key
and Histogram
Count
0
10000
0
0.02
0.06
0.1
Value
PDD matrix
H7N3
window = 500 step = 50
PB2
PB1
PA
HA
NP
NA
M
NS

(a)

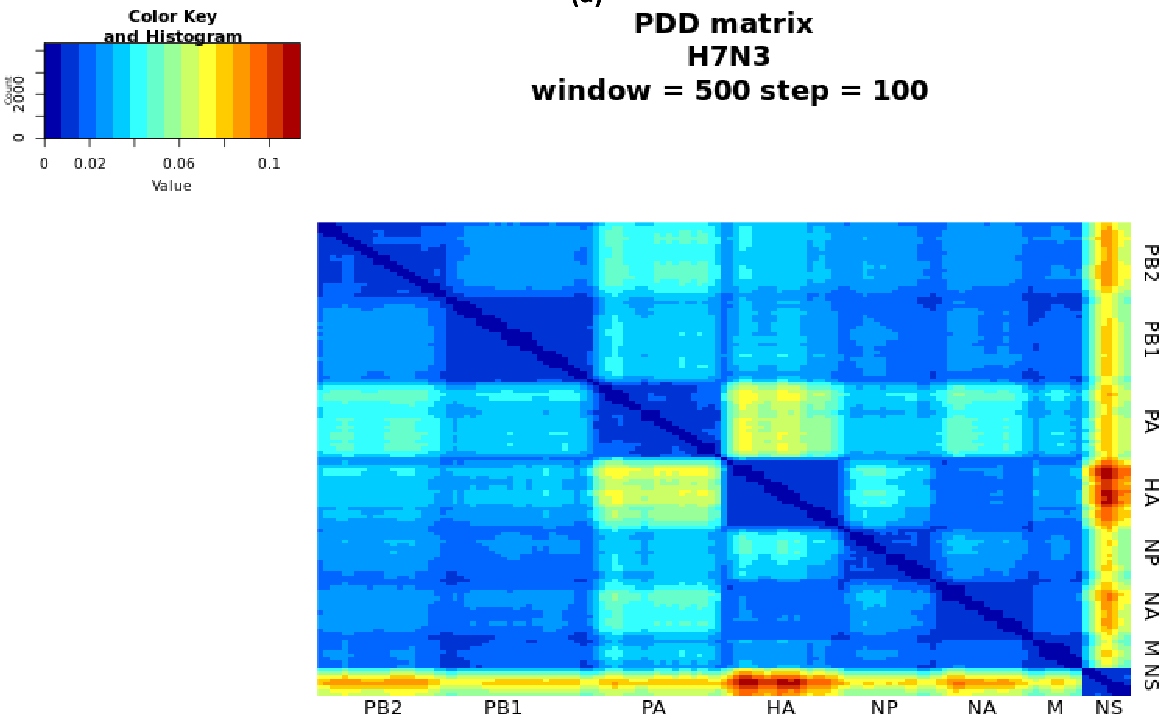
Color Key
and Histogram
Count
0
2000
0
0.02
0.06
0.1
Value
PDD matrix
H7N3
window = 500 step = 100
PB2
PB1
PA
HA
NP
NA
M
NS

(b)

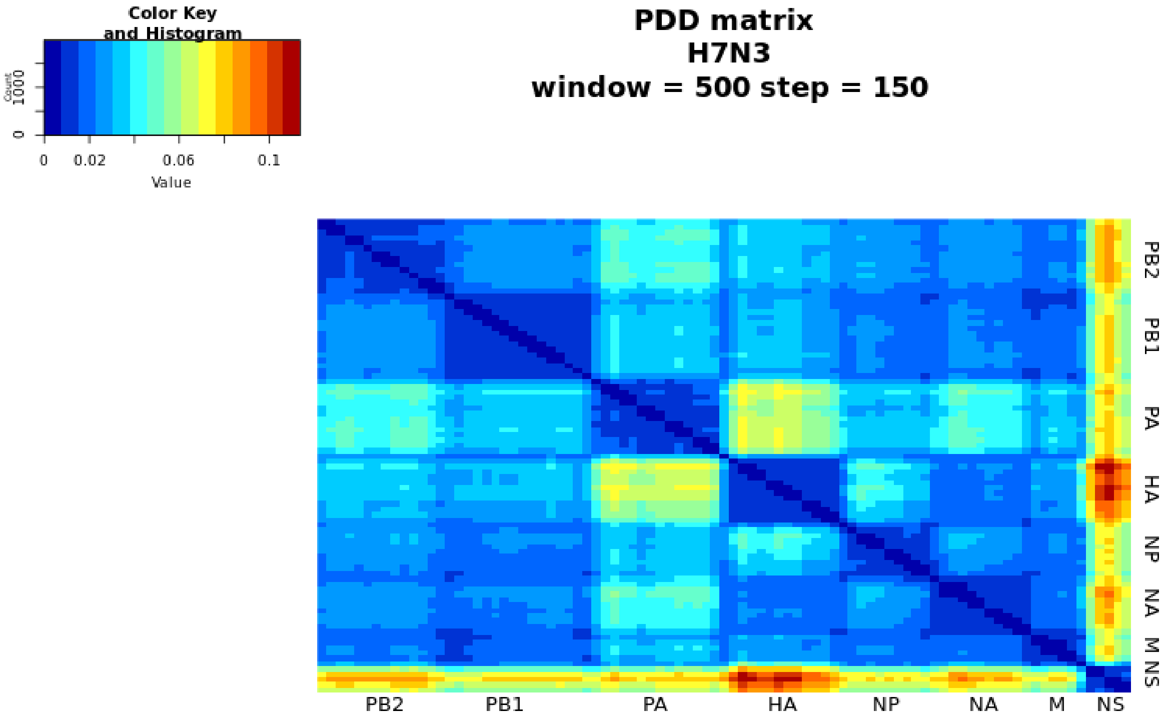
Color Key
and Histogram
Count
1000
0
0
0.02
0.06
0.1
Value
PDD matrix
H7N3
window = 500 step = 150
PB2
PB1
PA
HA
NP
NA
M
NS

(c)

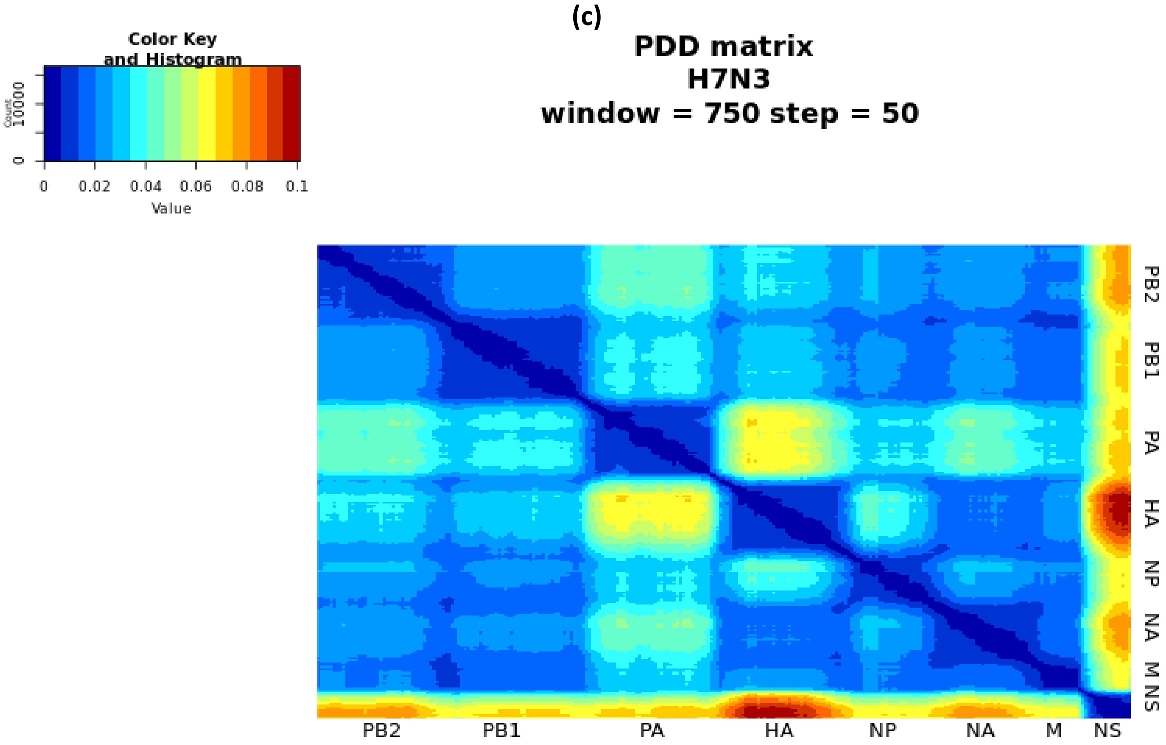
Color Key
and Histogram
Count
10000
0
0
0.02
0.04
0.06
0.08
0.1
Value
PDD matrix
H7N3
window = 750 step = 50
PB2
PB1
PA
HA
NP
NA
M
NS

(d)

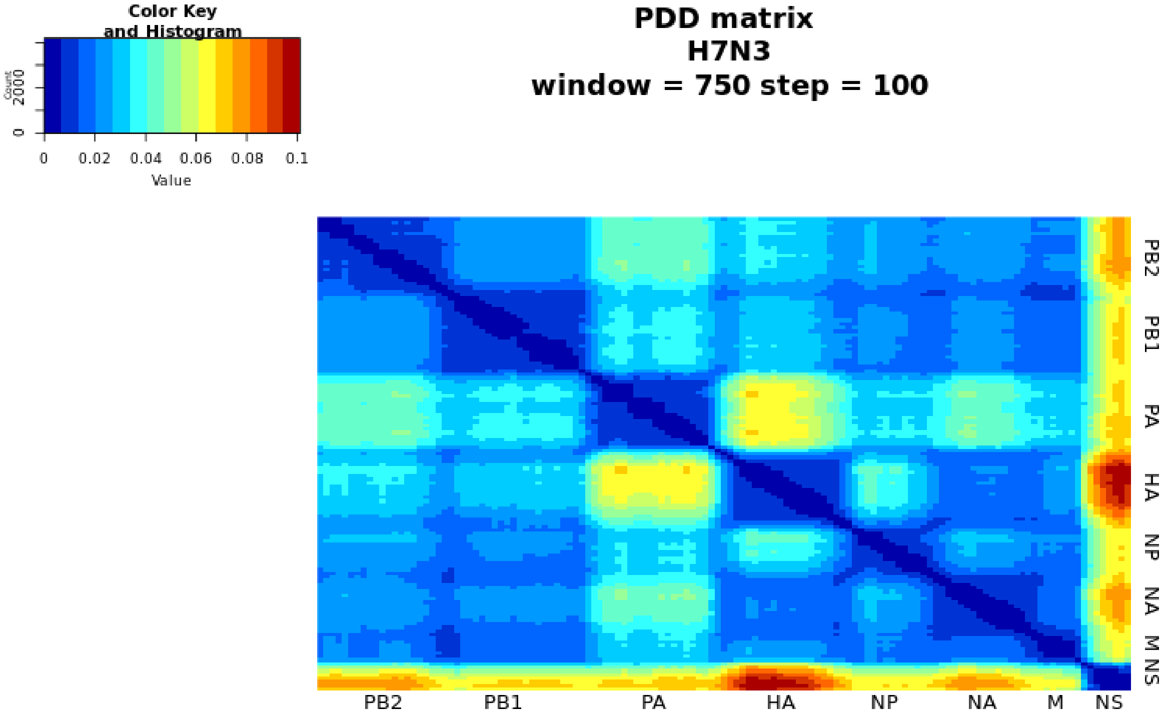
Color Key
and Histogram
Count
2000
0
0
0.02
0.04
0.06
0.08
0.1
Value
PDD matrix
H7N3
window = 750 step = 100
PB2
PB1
PA
HA
NP
NA
M
NS

(e)

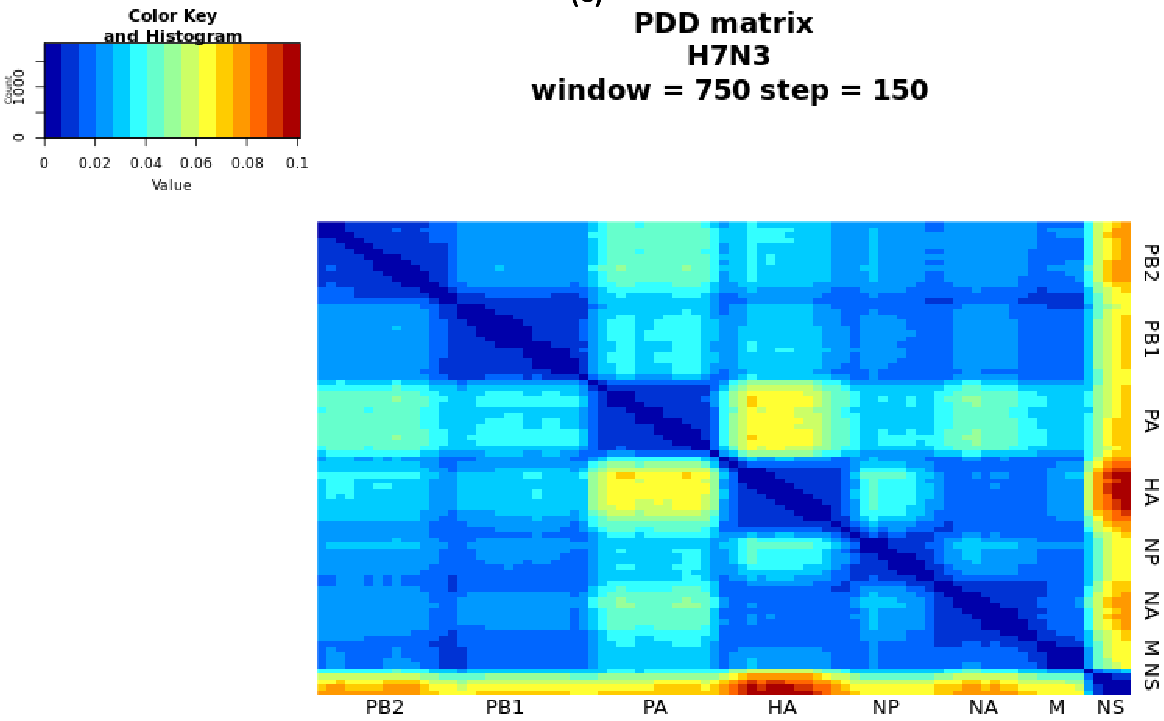
Color Key
and Histogram
Count
1000
0
0
0.02
0.04
0.06
0.08
0.1
Value
PDD matrix
H7N3
window = 750 step = 150
PB2
PB1
PA
HA
NP
NA
M
NS

(f)

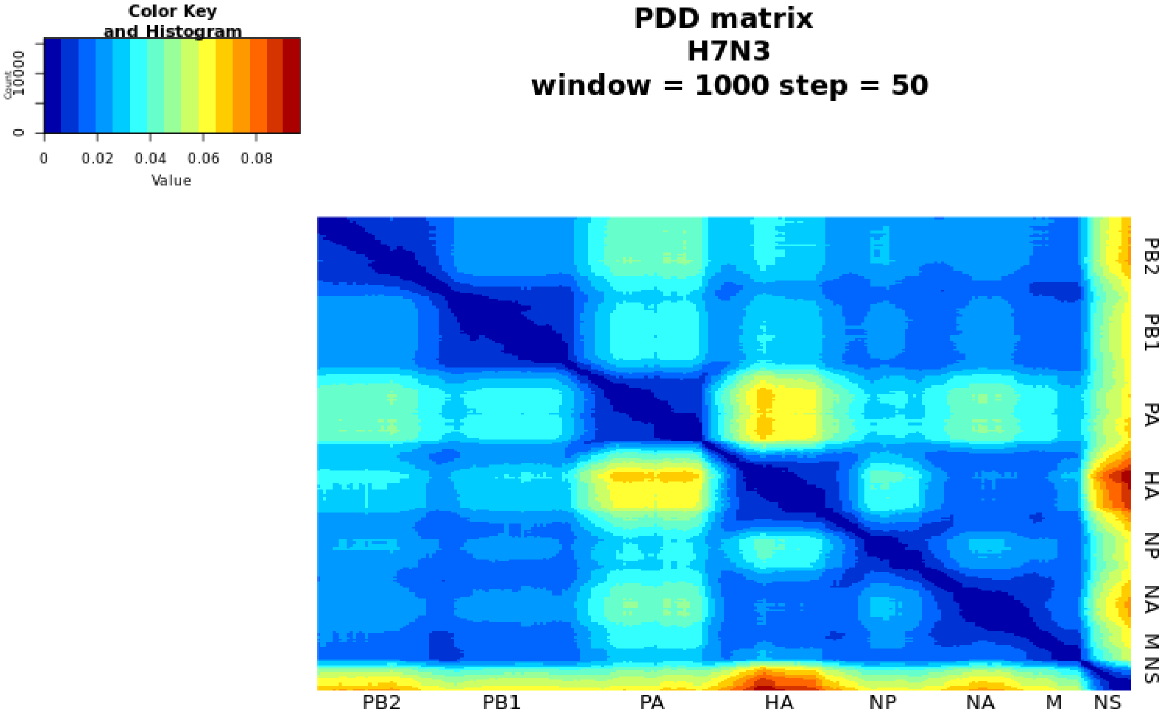
Color Key
and Histogram
Count
10000
0
0
0.02
0.04
0.06
0.08
Value
PDD matrix
H7N3
window = 1000 step = 50
PB2
PB1
PA
HA
NP
NA
M
NS

(g)

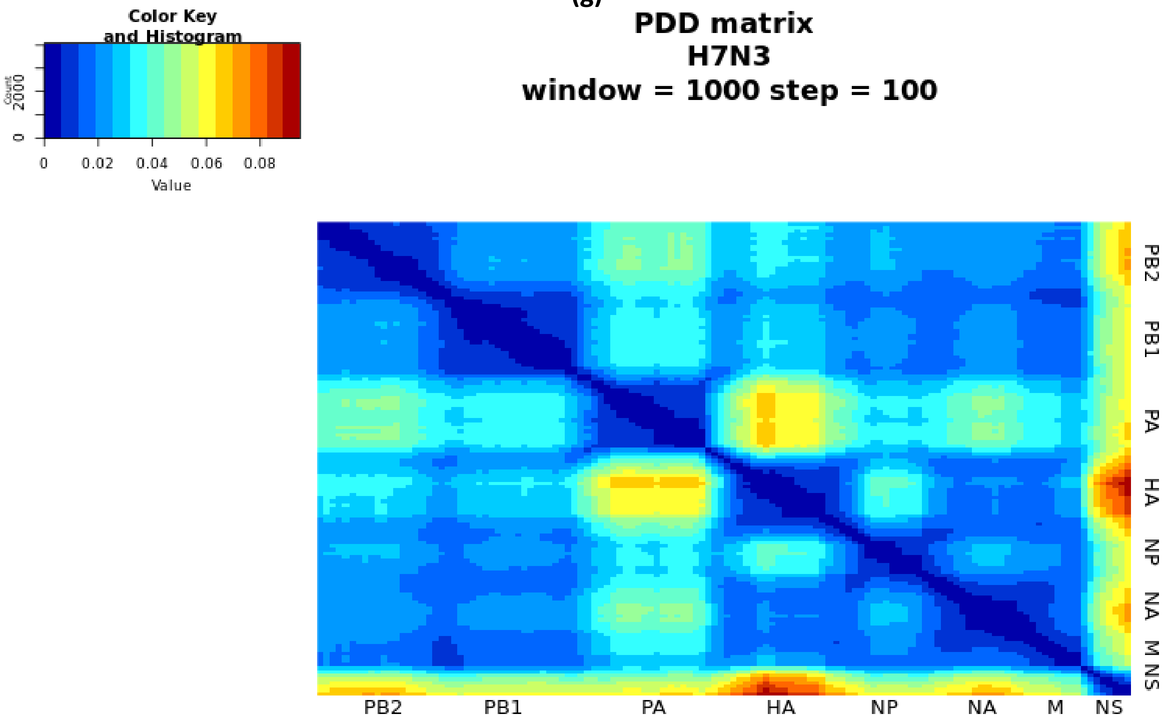
Color Key
and Histogram
Count
2000
0
0
0.02
0.04
0.06
0.08
Value
PDD matrix
H7N3
window = 1000 step = 100
PB2
PB1
PA
HA
NP
NA
M
NS

(h)

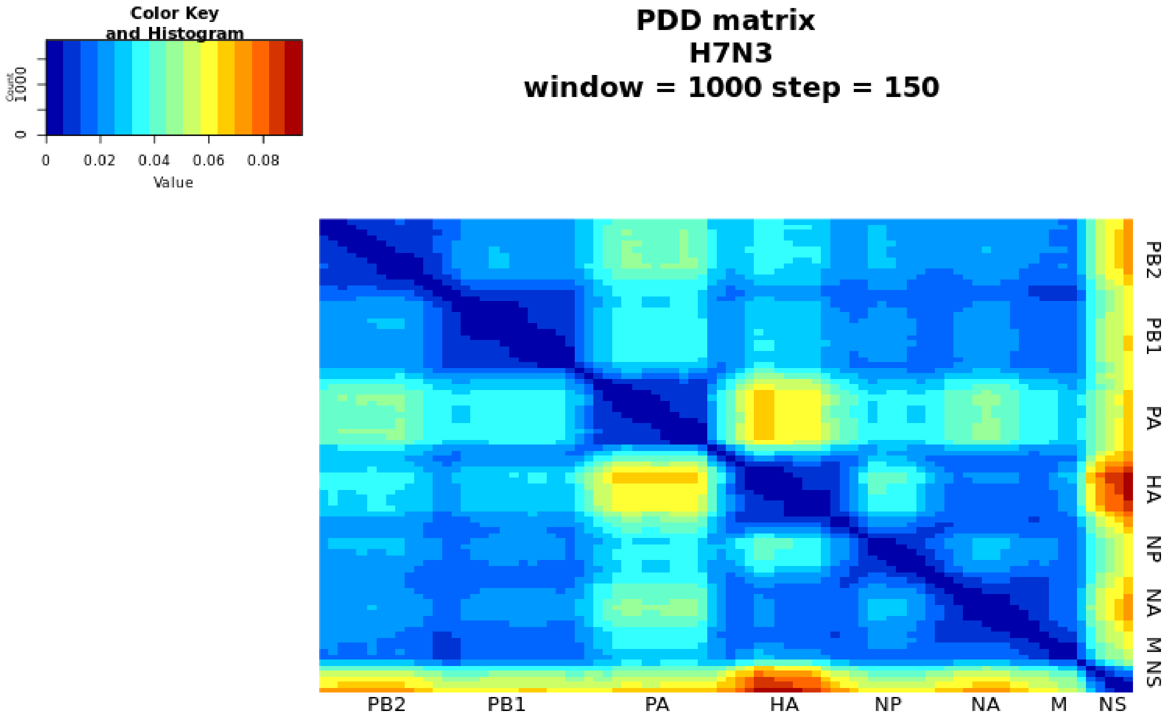

(i)

Supplementary Figure S3. Pairwise distance divergence matrix (PDDM) for H7N3 influenza viruses **(a)** Window = 500 nucleotides, Step = 50 nucleotides **(b)** Window = 500 nucleotides, Step = 100 nucleotides **(c)** Window = 500 nucleotides, Step = 150 nucleotides **(d)** Window = 750 nucleotides, Step = 50 nucleotides **(e)** Window = 750 nucleotides, Step = 100 nucleotides **(f)** Window = 750 nucleotides, Step = 150 nucleotides **(g)** Window = 1000 nucleotides, Step = 50 nucleotides **(h)** Window = 1000 nucleotides, Step = 100 nucleotides **(I)** Window = 1000 nucleotides, Step = 150 nucleotides. The color gradient scale shows the root mean square error (RMSE) values in PDCP is a figure.

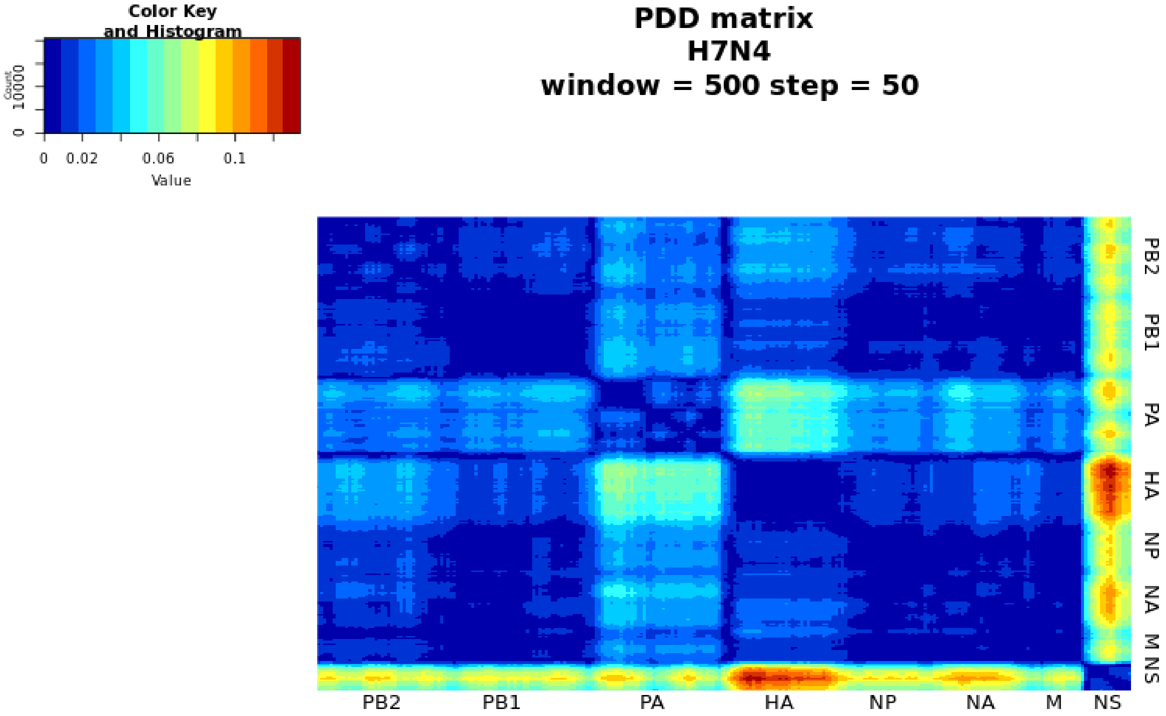
Color Key
and Histogram
Count
0
10000
0
0.02
0.06
0.1
Value
PDD matrix
H7N4
window = 500 step = 50
PB2
PB1
PA
HA
NP
NA
M
NS
PB2
PB1
PA
HA
NP
NA
M
NS

(a)

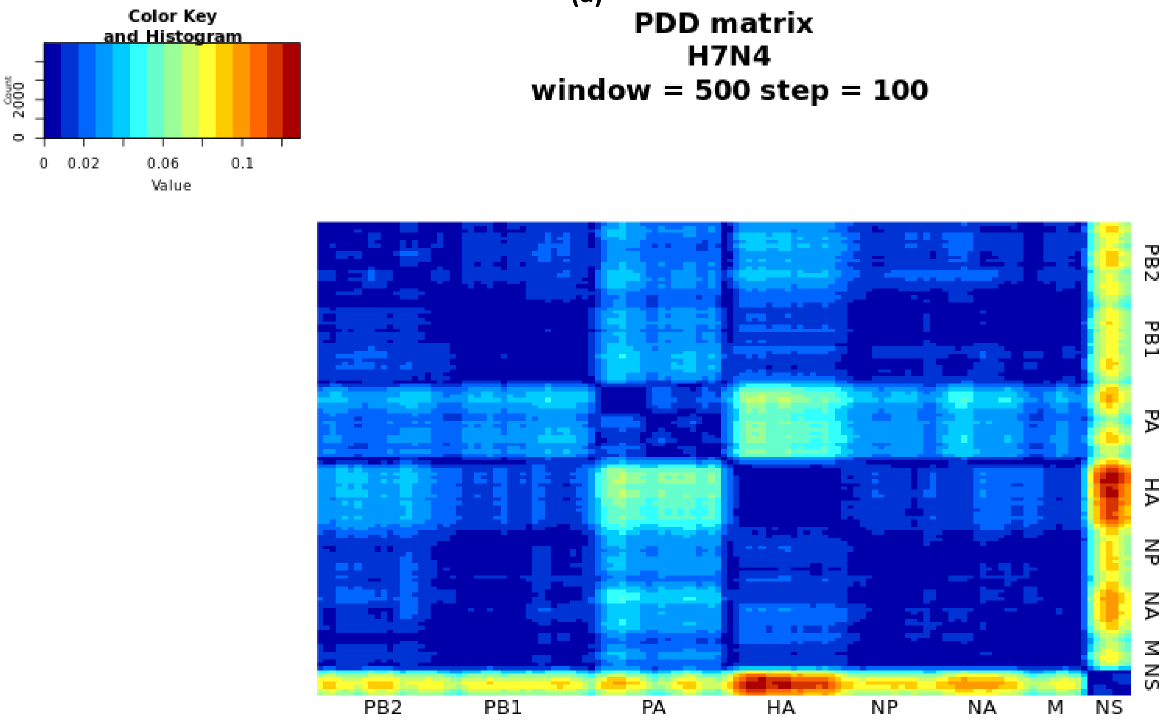
Color Key
and Histogram
Count
0
2000
0
0.02
0.06
0.1
Value
PDD matrix
H7N4
window = 500 step = 100
PB2
PB1
PA
HA
NP
NA
M
NS
PB2
PB1
PA
HA
NP
NA
M
NS

(b)

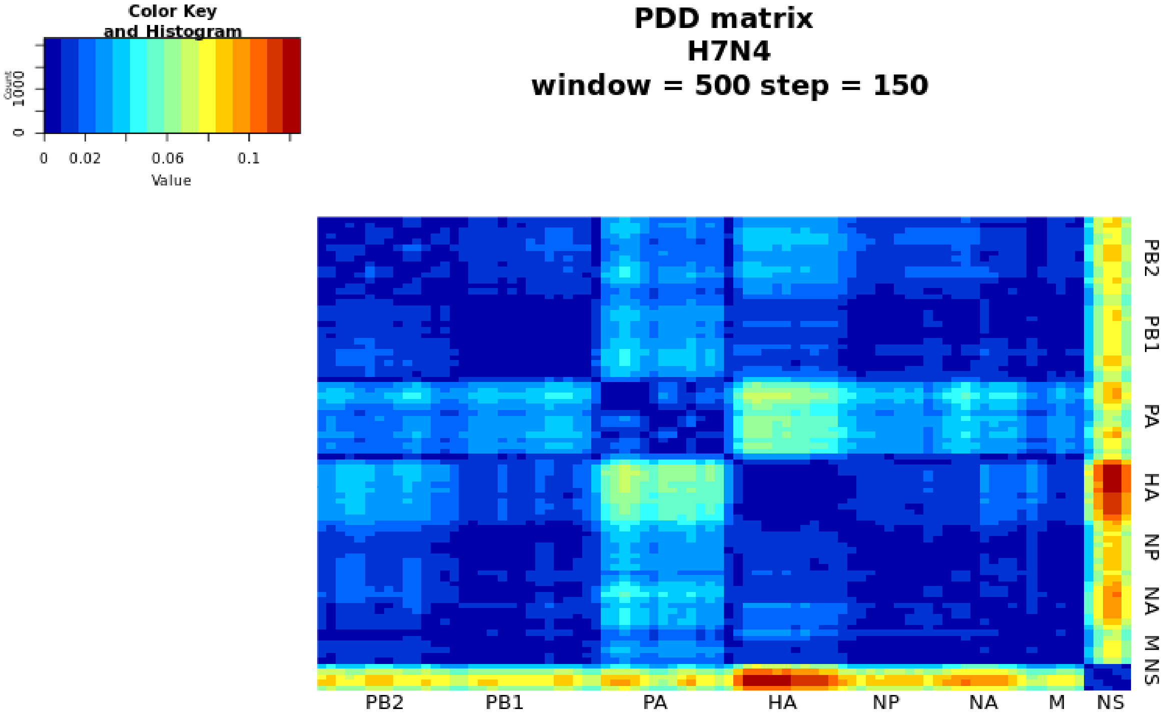
Color Key
and Histogram
Count
0
1000
0
0.02
0.06
0.1
Value
PDD matrix
H7N4
window = 500 step = 150
PB2
PB1
PA
HA
NP
NA
M
NS

(c)

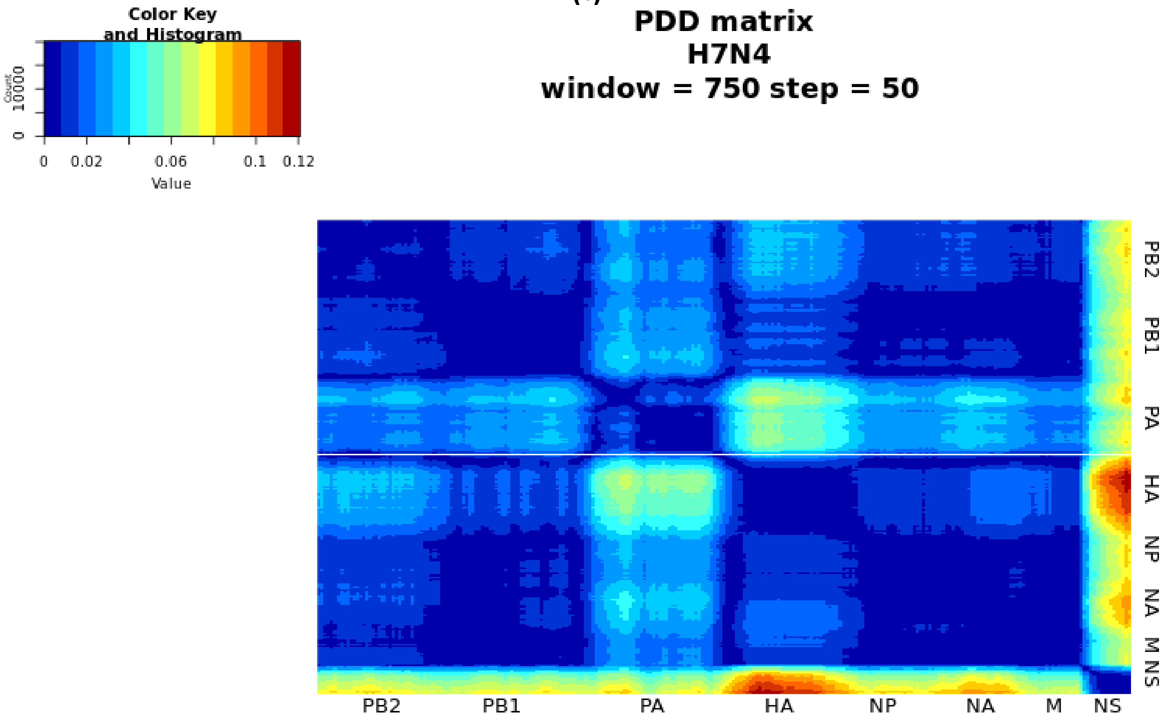
Color Key
and Histogram
Count
0
10000
0
0.02
0.06
0.1
0.12
Value
PDD matrix
H7N4
window = 750 step = 50
PB2
PB1
PA
HA
NP
NA
M
NS

(d)

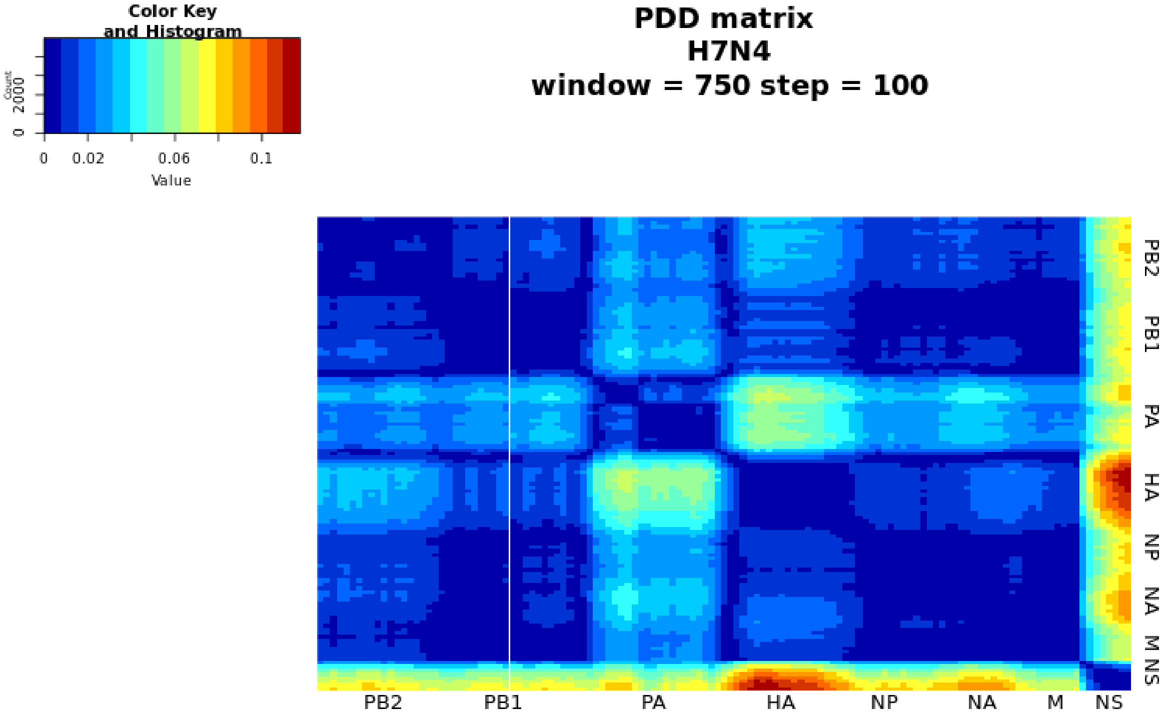
Color Key
and Histogram
Count
0
2000
0
0.02
0.06
0.1
Value
PDD matrix
H7N4
window = 750 step = 100
PB2
PB1
PA
HA
NP
NA
M
NS

(e)

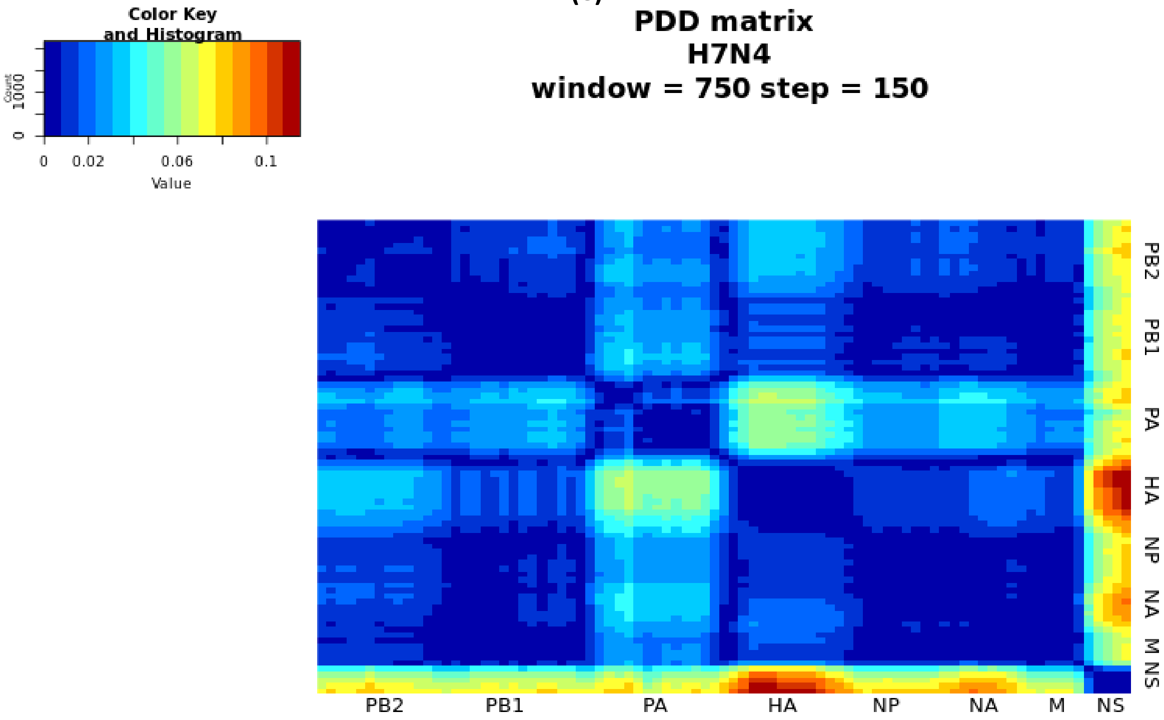
Color Key
and Histogram
Count
0
1000
0
0.02
0.06
0.1
Value
PDD matrix
H7N4
window = 750 step = 150
PB2
PB1
PA
HA
NP
NA
M
NS

(f)

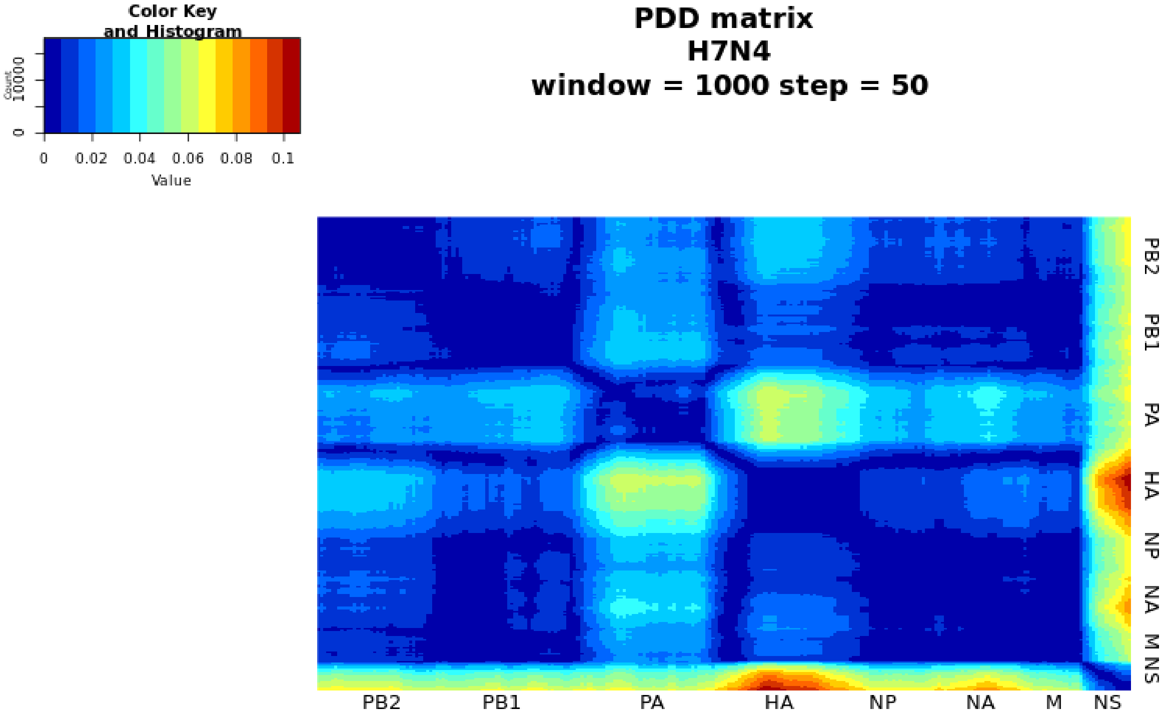
Color Key
and Histogram
Count
10000
0
0
0.02
0.04
0.06
0.08
0.1
Value
PDD matrix
H7N4
window = 1000 step = 50
PB2
PB1
PA
HA
NP
NA
M
NS

(g)

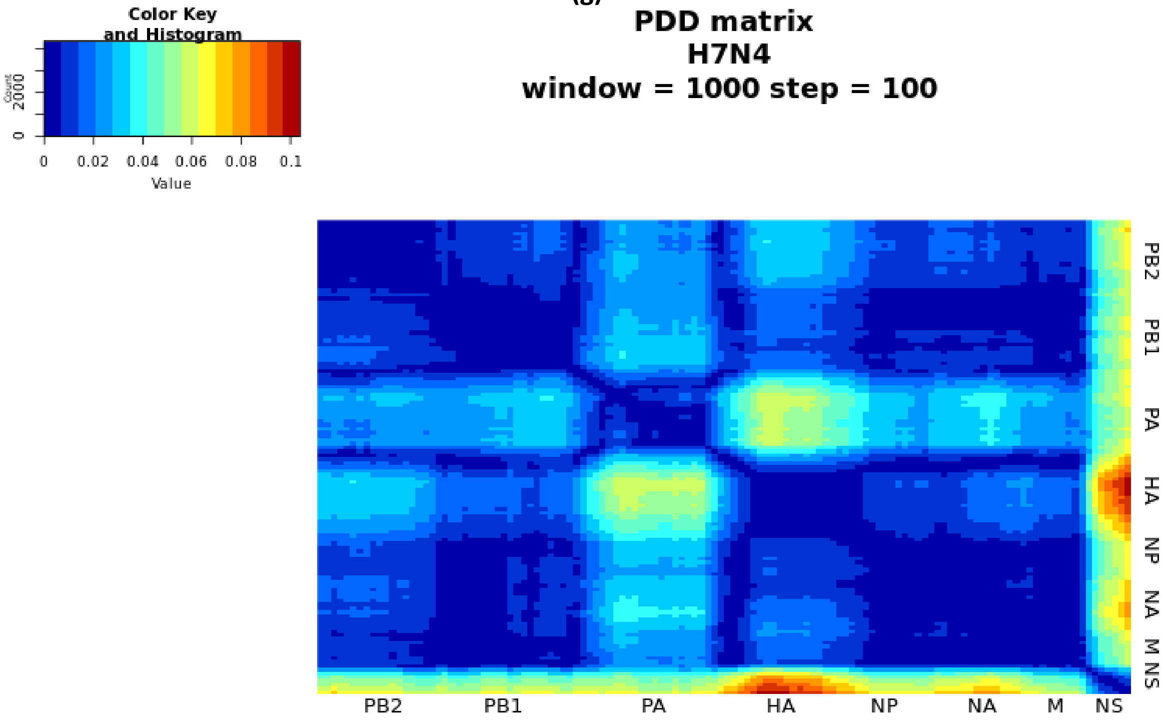
Color Key
and Histogram
Count
2000
0
0
0.02
0.04
0.06
0.08
0.1
Value
PDD matrix
H7N4
window = 1000 step = 100
PB2
PB1
PA
HA
NP
NA
M
NS

(h)

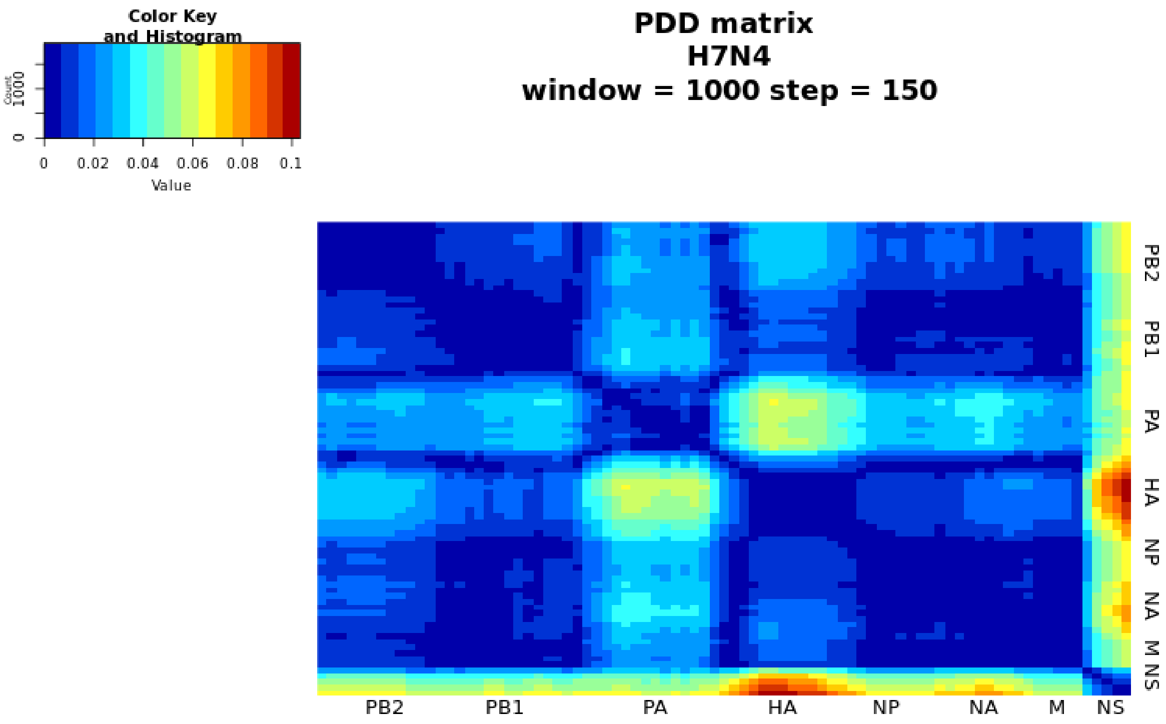

(i)

Supplementary Figure S4. Pairwise distance divergence matrix (PDDM) for H7N4 influenza viruses **(a)** Window = 500 nucleotides, Step = 50 nucleotides **(b)** Window = 500 nucleotides, Step = 100 nucleotides **(c)** Window = 500 nucleotides, Step = 150 nucleotides **(d)** Window = 750 nucleotides, Step = 50 nucleotides **(e)** Window = 750 nucleotides, Step = 100 nucleotides **(f)** Window = 750 nucleotides, Step = 150 nucleotides **(g)** Window = 1000 nucleotides, Step = 50 nucleotides **(h)** Window = 1000 nucleotides, Step = 100 nucleotides **(I)** Window = 1000 nucleotides, Step = 150 nucleotides. The color gradient scale shows the root mean square error (RMSE) values in PDCP is a figure.

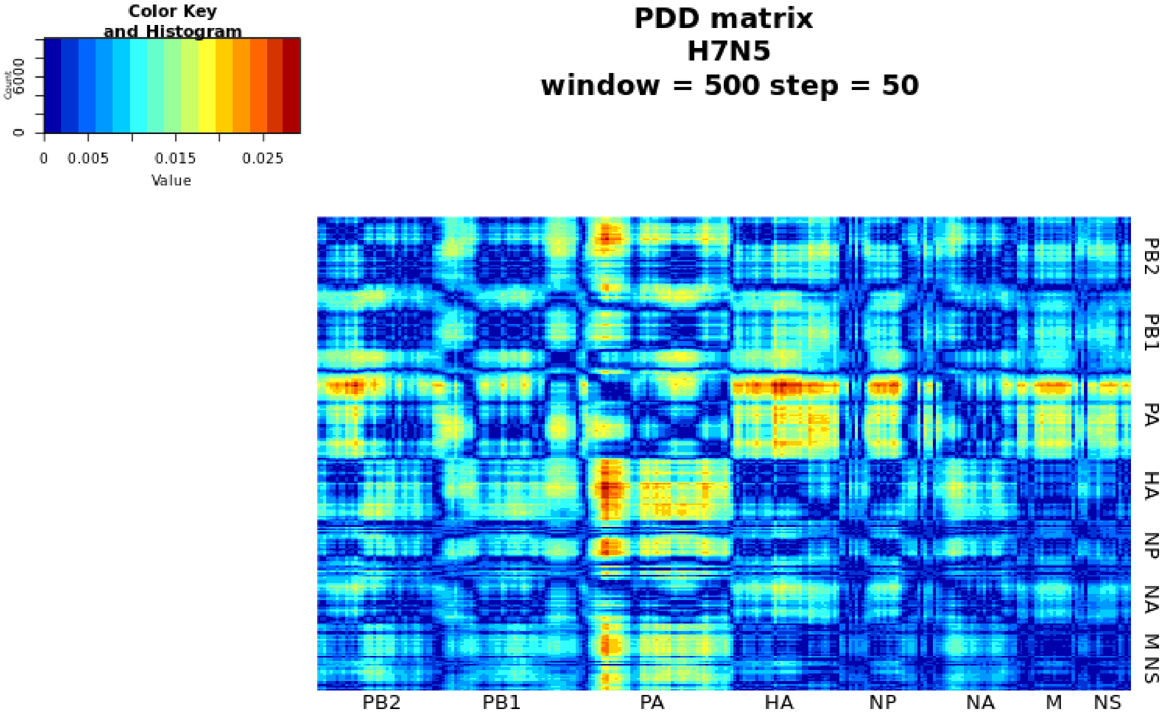
Color Key
and Histogram
Count
6000
0
0
0.005
0.015
0.025
Value
PDD matrix
H7N5
window = 500 step = 50
PB2
PB1
PA
HA
NP
NA
M
NS

(a)

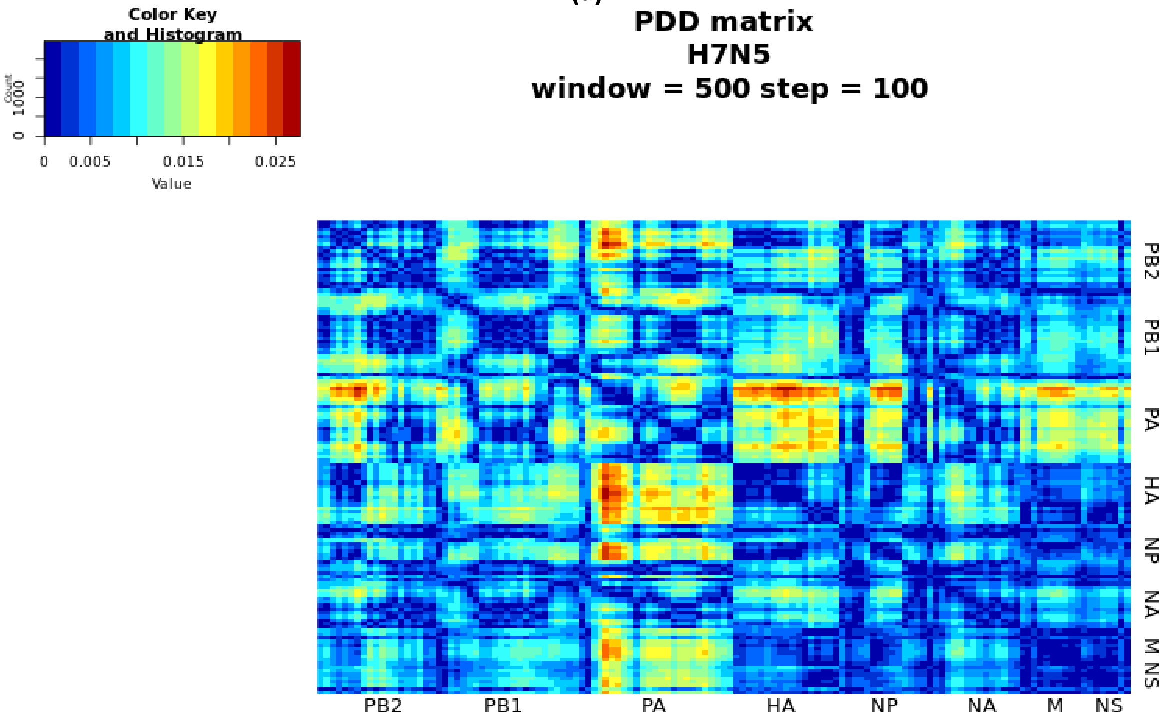
Color Key
and Histogram
Count
1000
0
0
0.005
0.015
0.025
Value
PDD matrix
H7N5
window = 500 step = 100
PB2
PB1
PA
HA
NP
NA
M
NS

(b)

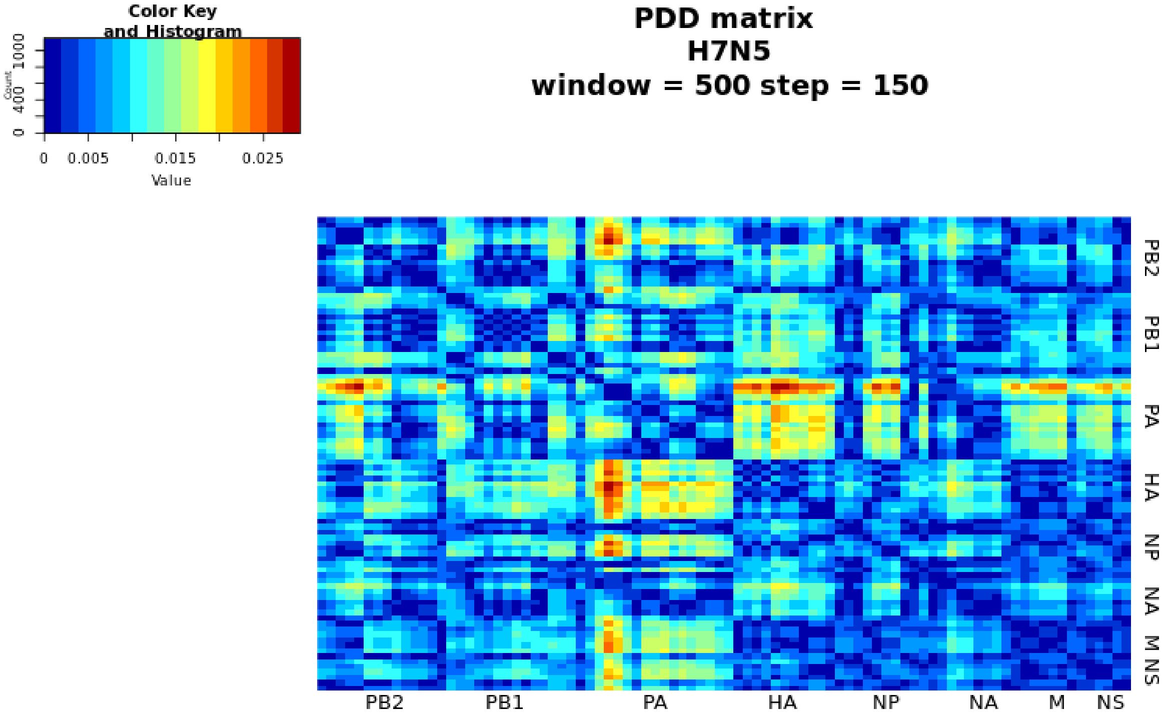
Color Key
and Histogram
Count
0
400
1000
0
0.005
0.015
0.025
Value
PDD matrix
H7N5
window = 500 step = 150
PB2
PB1
PA
HA
NP
NA
M
NS
PB2
PB1
PA
HA
NP
NA
M
NS

(c)

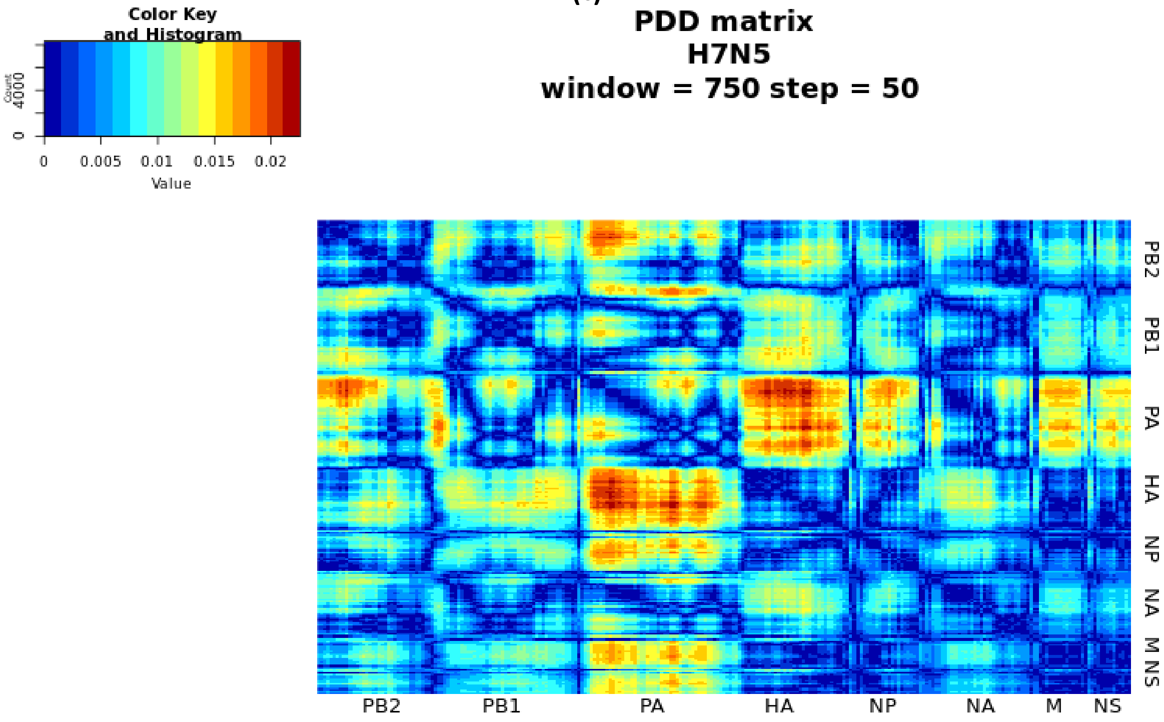
Color Key
and Histogram
Count
0
4000
0
0.005
0.01
0.015
0.02
Value
PDD matrix
H7N5
window = 750 step = 50
PB2
PB1
PA
HA
NP
NA
M
NS
PB2
PB1
PA
HA
NP
NA
M
NS

(d)

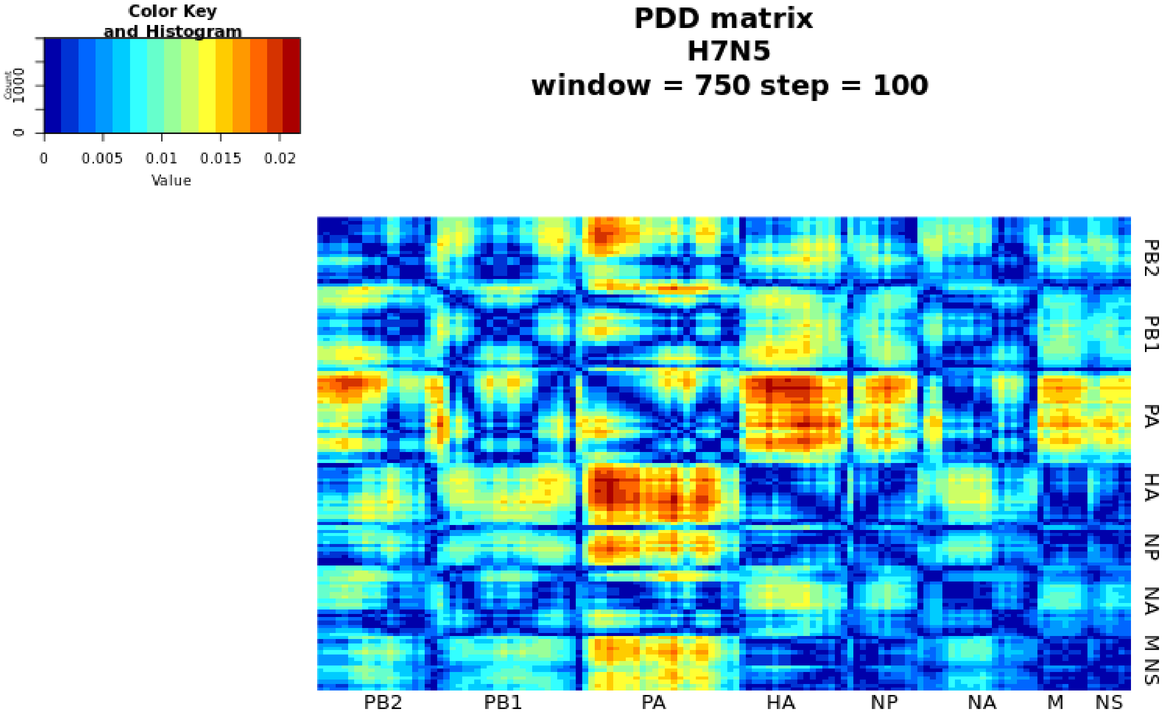
Color Key
and Histogram
Count
0
1000
0
0.005
0.01
0.015
0.02
Value
PDD matrix
H7N5
window = 750 step = 100
PB2
PB1
PA
HA
NP
NA
M
NS

(e)

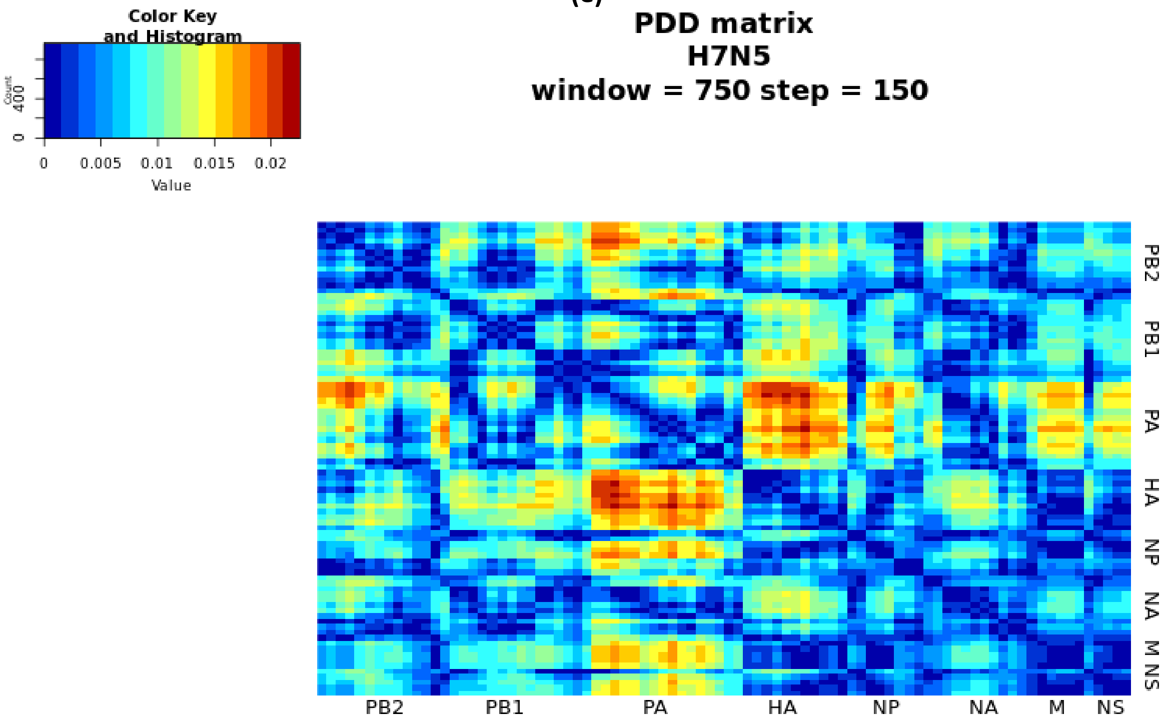
Color Key
and Histogram
Count
0
400
0
0.005
0.01
0.015
0.02
Value
PDD matrix
H7N5
window = 750 step = 150
PB2
PB1
PA
HA
NP
NA
M
NS

(f)

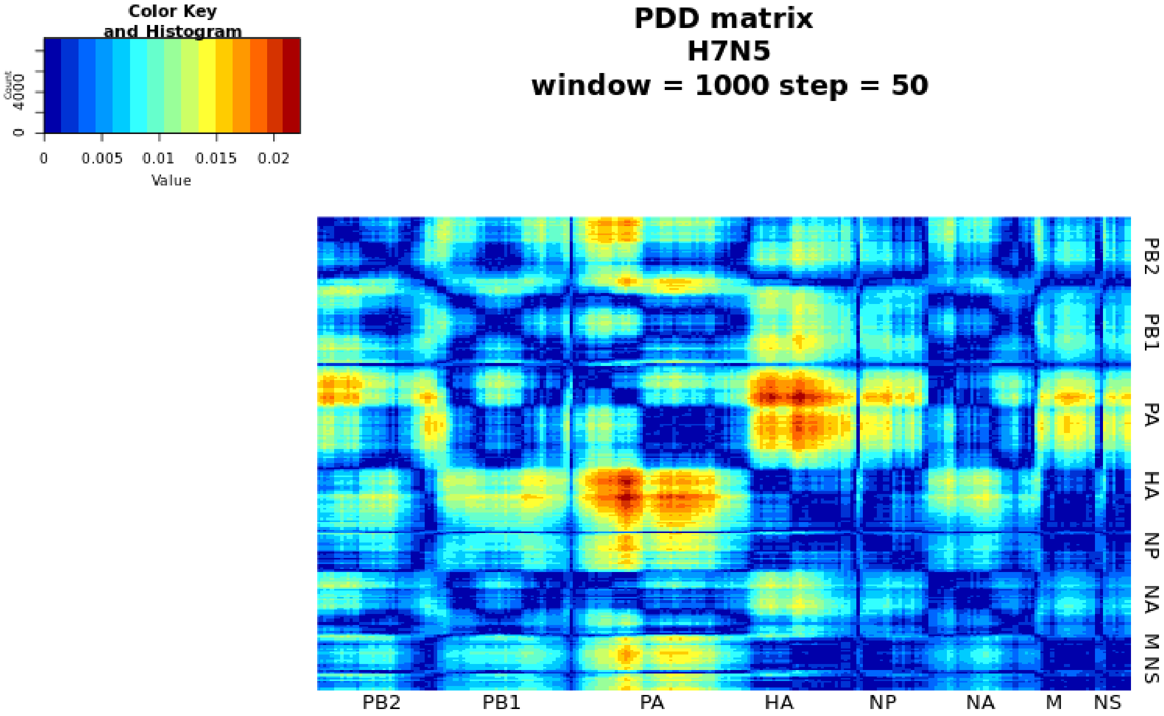
Color Key
and Histogram
Count
0
4000
0
0.005
0.01
0.015
0.02
Value
PDD matrix
H7N5
window = 1000 step = 50
PB2
PB1
PA
HA
NP
NA
M
NS

(g)

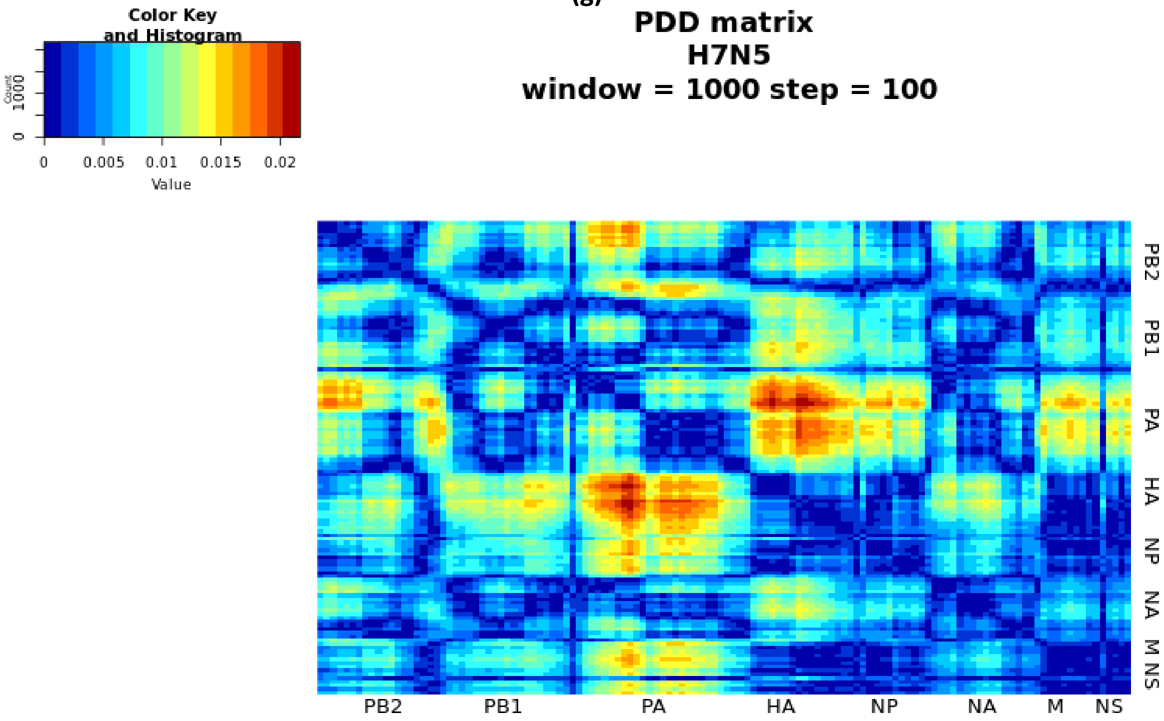
Color Key
and Histogram
Count
0
1000
0
0.005
0.01
0.015
0.02
Value
PDD matrix
H7N5
window = 1000 step = 100
PB2
PB1
PA
HA
NP
NA
M
NS

(h)

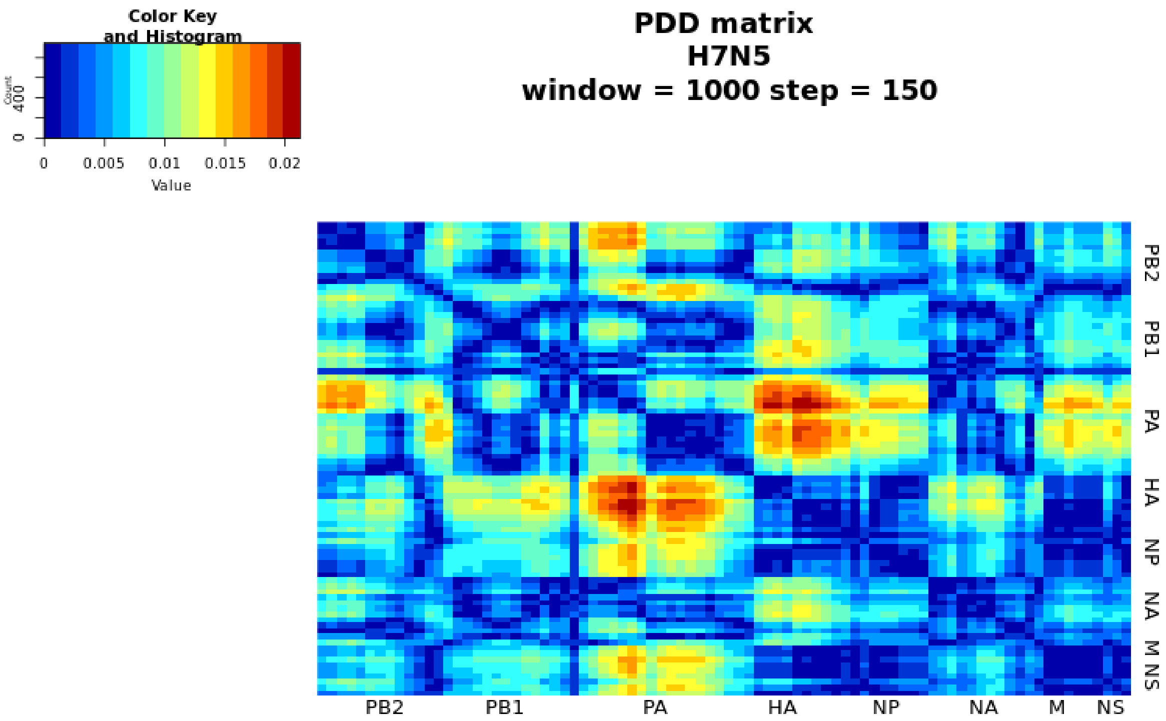

(i)

Supplementary Figure S5. Pairwise distance divergence matrix (PDDM) for H7N5 influenza viruses **(a)** Window = 500 nucleotides, Step = 50 nucleotides **(b)** Window = 500 nucleotides, Step = 100 nucleotides **(c)** Window = 500 nucleotides, Step = 150 nucleotides **(d)** Window = 750 nucleotides, Step = 50 nucleotides **(e)** Window = 750 nucleotides, Step = 100 nucleotides **(f)** Window = 750 nucleotides, Step = 150 nucleotides **(g)** Window = 1000 nucleotides, Step = 50 nucleotides **(h)** Window = 1000 nucleotides, Step = 100 nucleotides **(I)** Window = 1000 nucleotides, Step = 150 nucleotides. The color gradient scale shows the root mean square error (RMSE) values in PDCP is a figure.

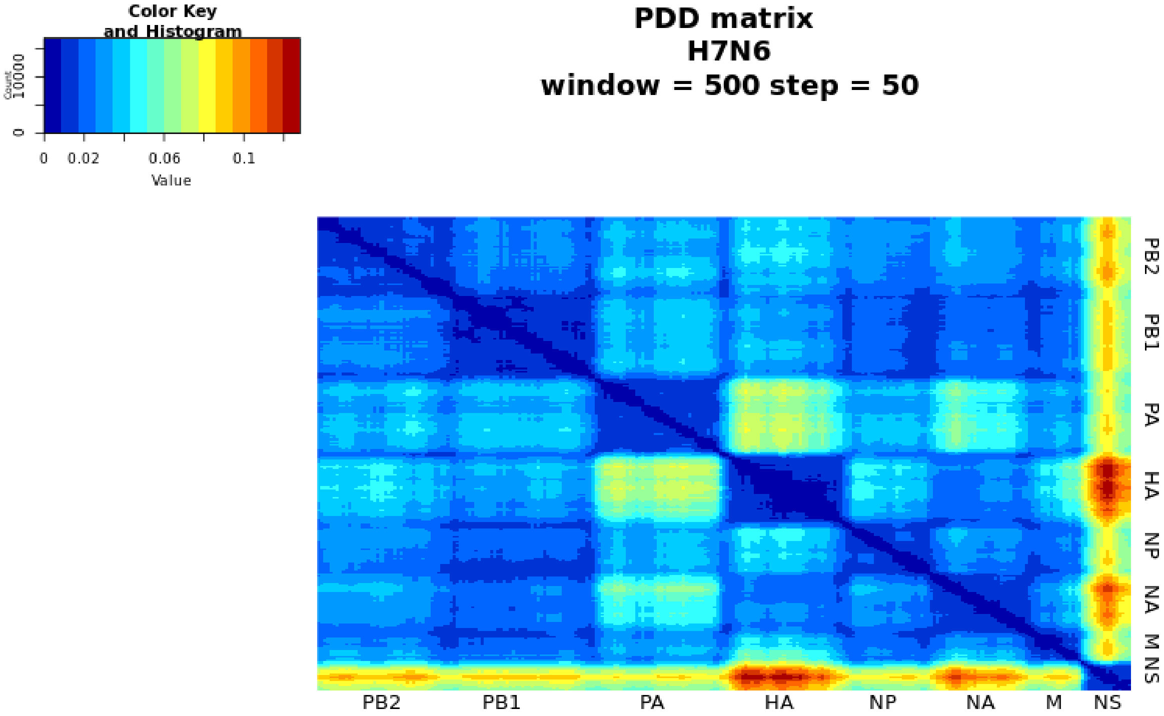

Color Key
and Histogram
Count
10000
0
0
0.02
0.06
0.1
Value
PDD matrix
H7N6
window = 500 step = 50
PB2
PB1
PA
HA
NP
NA
M
NS

(a)

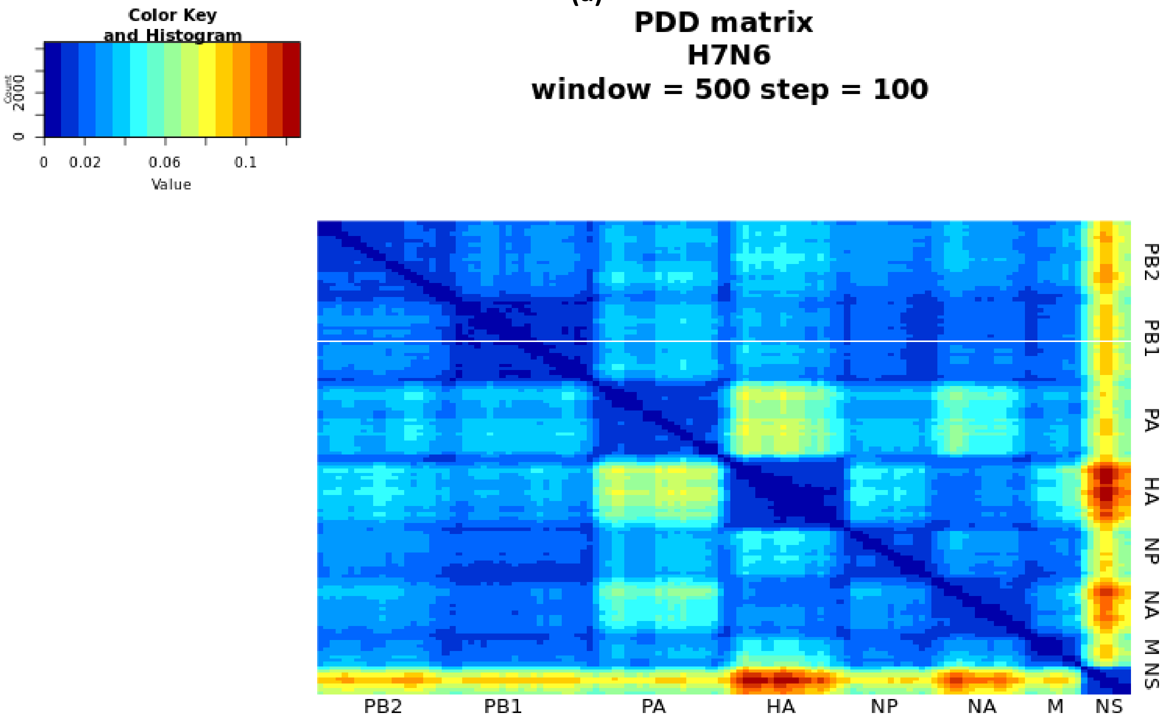

Color Key
and Histogram
Count
2000
0
0
0.02
0.06
0.1
Value
PDD matrix
H7N6
window = 500 step = 100
PB2
PB1
PA
HA
NP
NA
M
NS

(b)

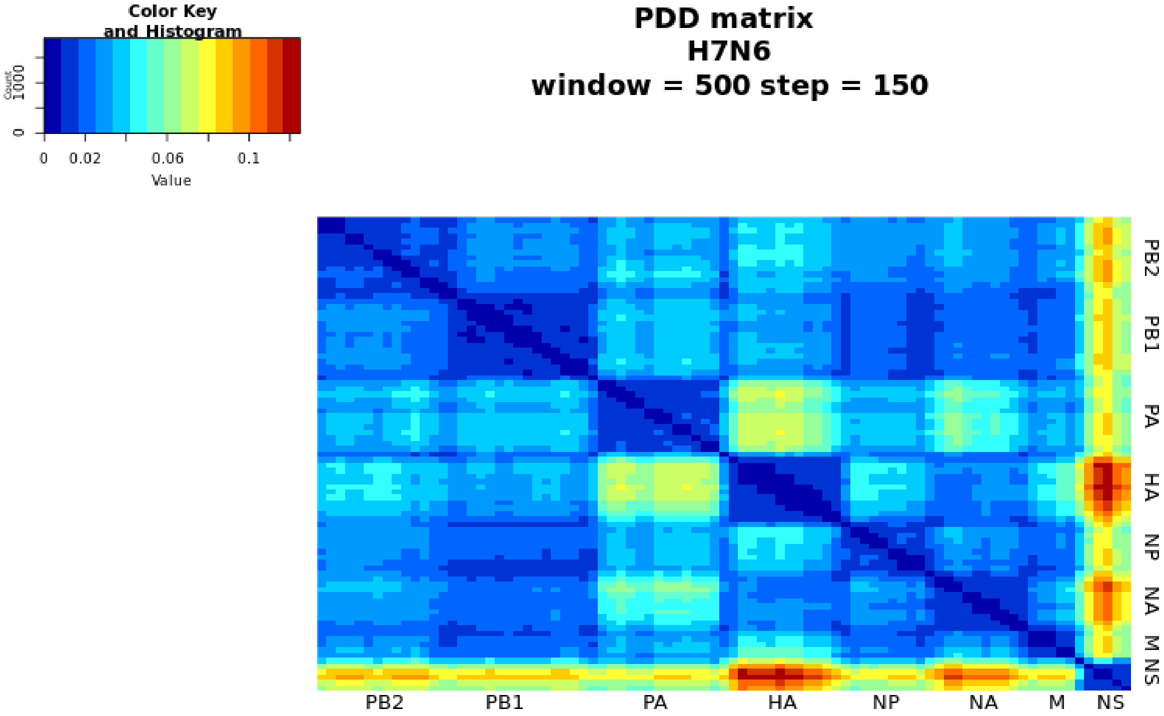
Color Key
and Histogram
Count
0
1000
0
0.02
0.06
0.1
Value
PDD matrix
H7N6
window = 500 step = 150
PB2
PB1
PA
HA
NP
NA
M
NS
PB2
PB1
PA
HA
NP
NA
M
NS

(c)

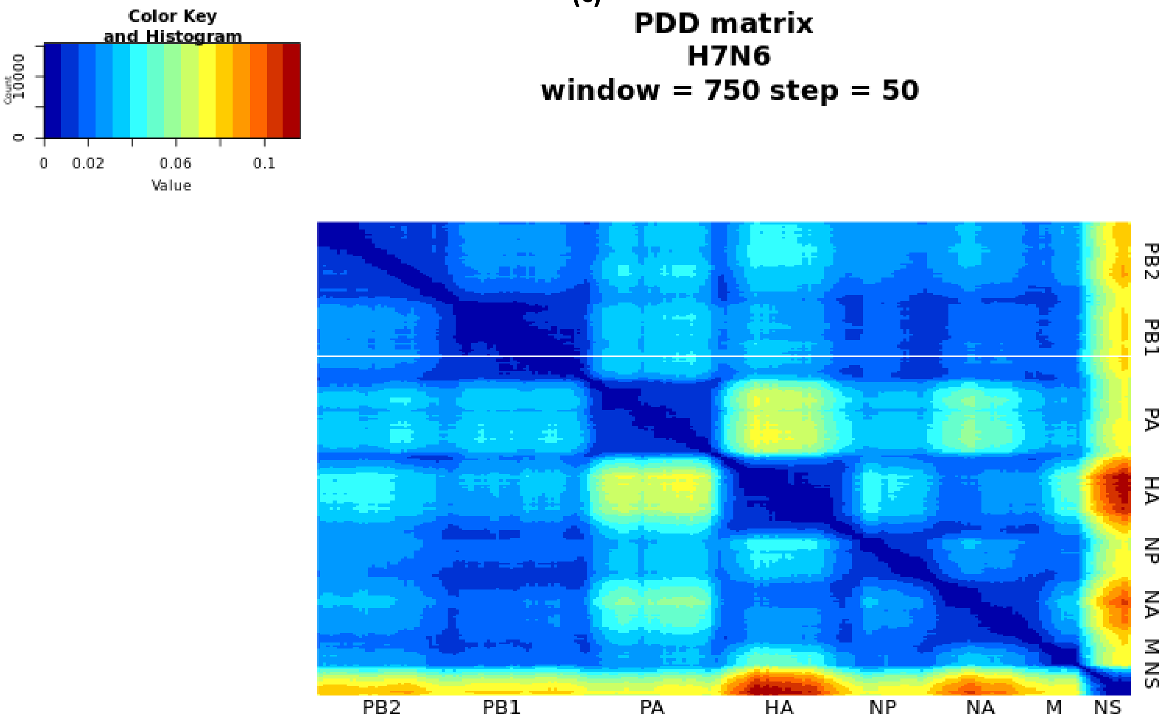
Color Key
and Histogram
Count
0
10000
0
0.02
0.06
0.1
Value
PDD matrix
H7N6
window = 750 step = 50
PB2
PB1
PA
HA
NP
NA
M
NS
PB2
PB1
PA
HA
NP
NA
M
NS

(d)

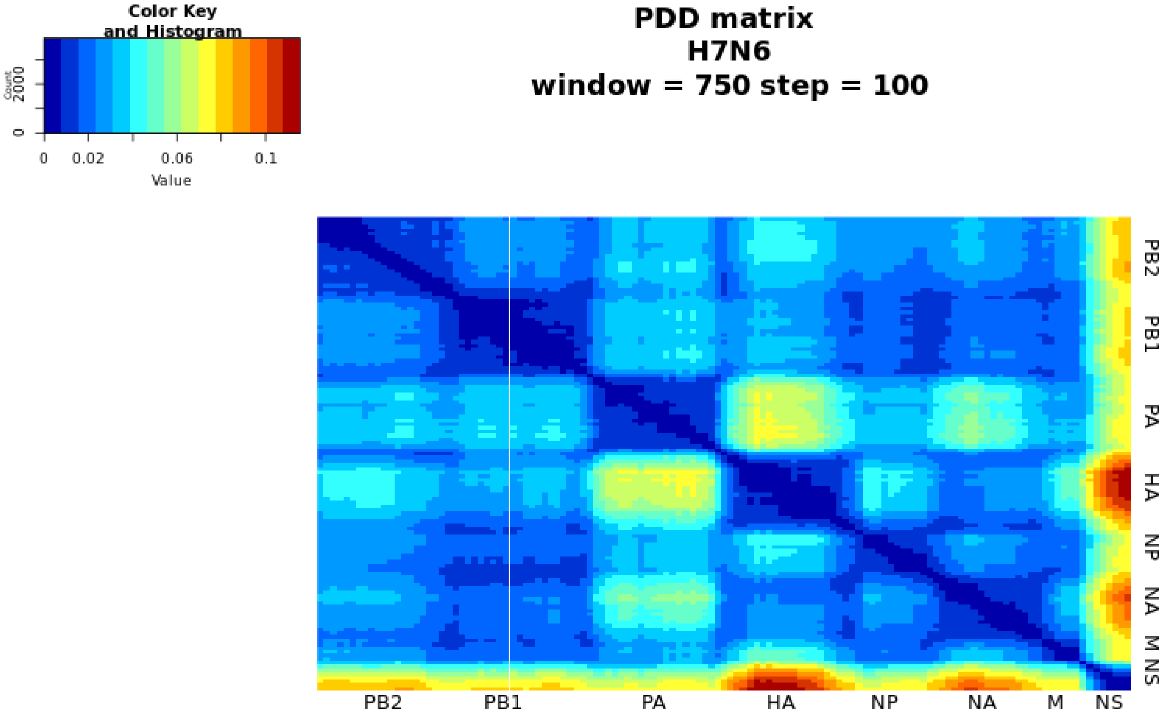
Color Key
and Histogram
Count
2000
0
0
0.02
0.06
0.1
Value
PDD matrix
H7N6
window = 750 step = 100
PB2
PB1
PA
HA
NP
NA
M
NS

(e)

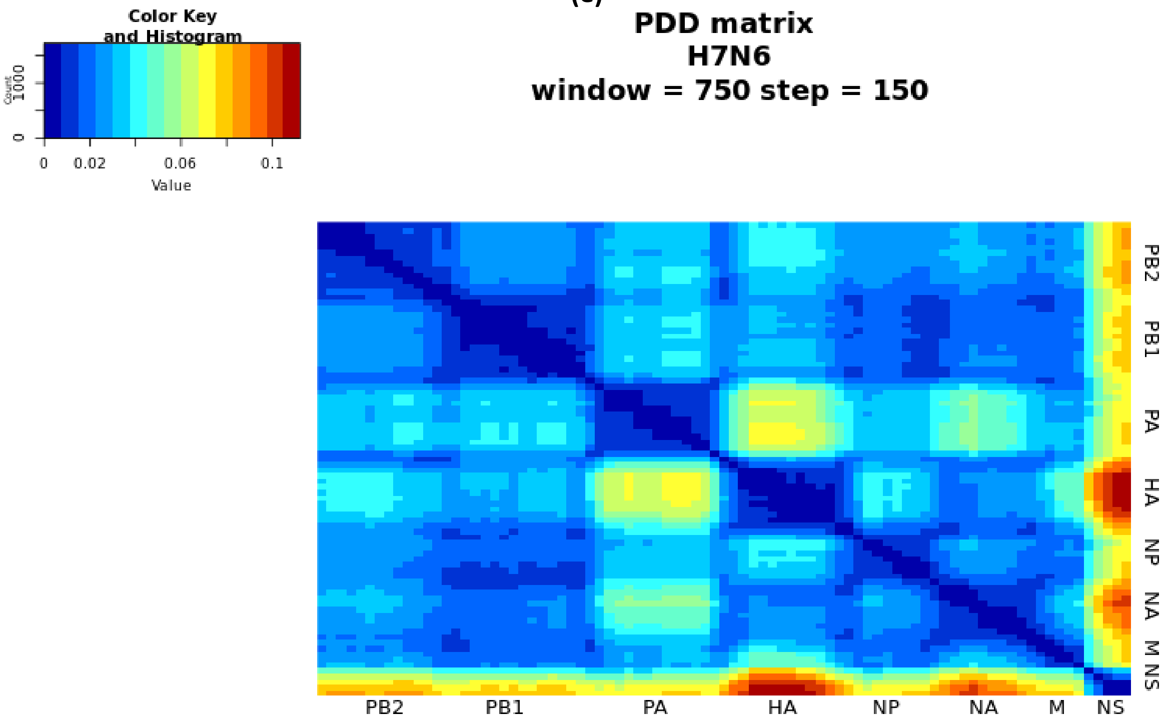
Color Key
and Histogram
Count
1000
0
0
0.02
0.06
0.1
Value
PDD matrix
H7N6
window = 750 step = 150
PB2
PB1
PA
HA
NP
NA
M
NS

(f)

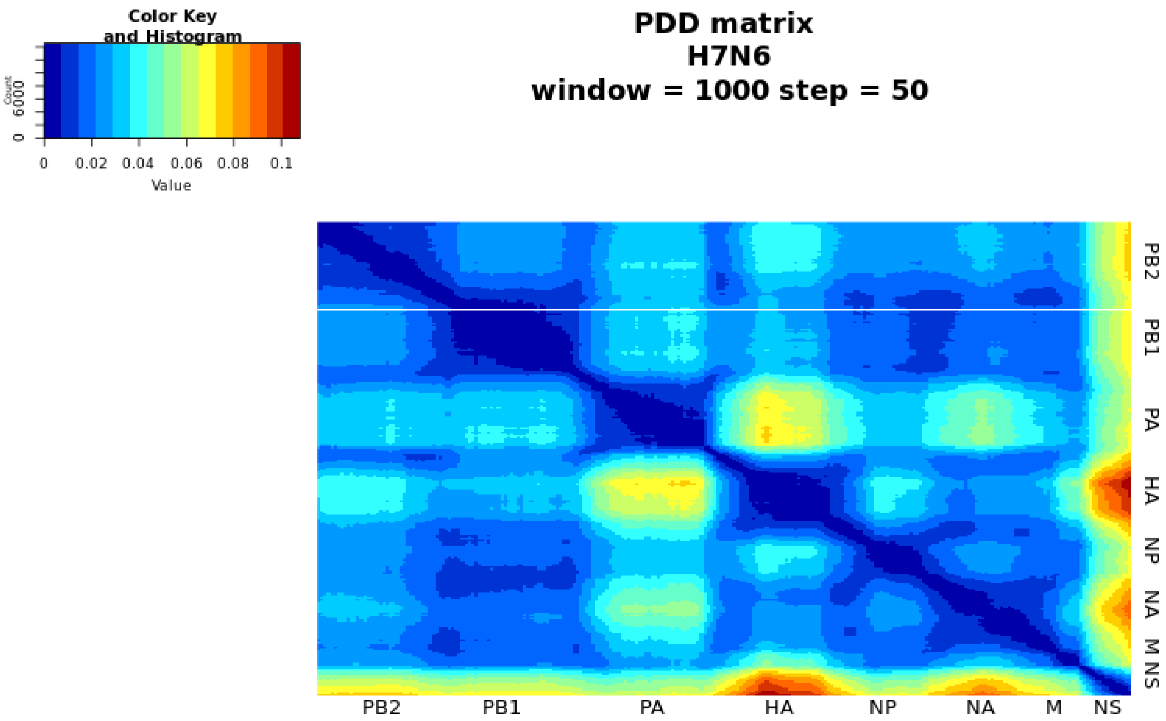
Color Key
and Histogram
Count
0
6000
0
0.02
0.04
0.06
0.08
0.1
Value
PDD matrix
H7N6
window = 1000 step = 50
PB2
PB1
PA
HA
NP
NA
M
NS

(g)

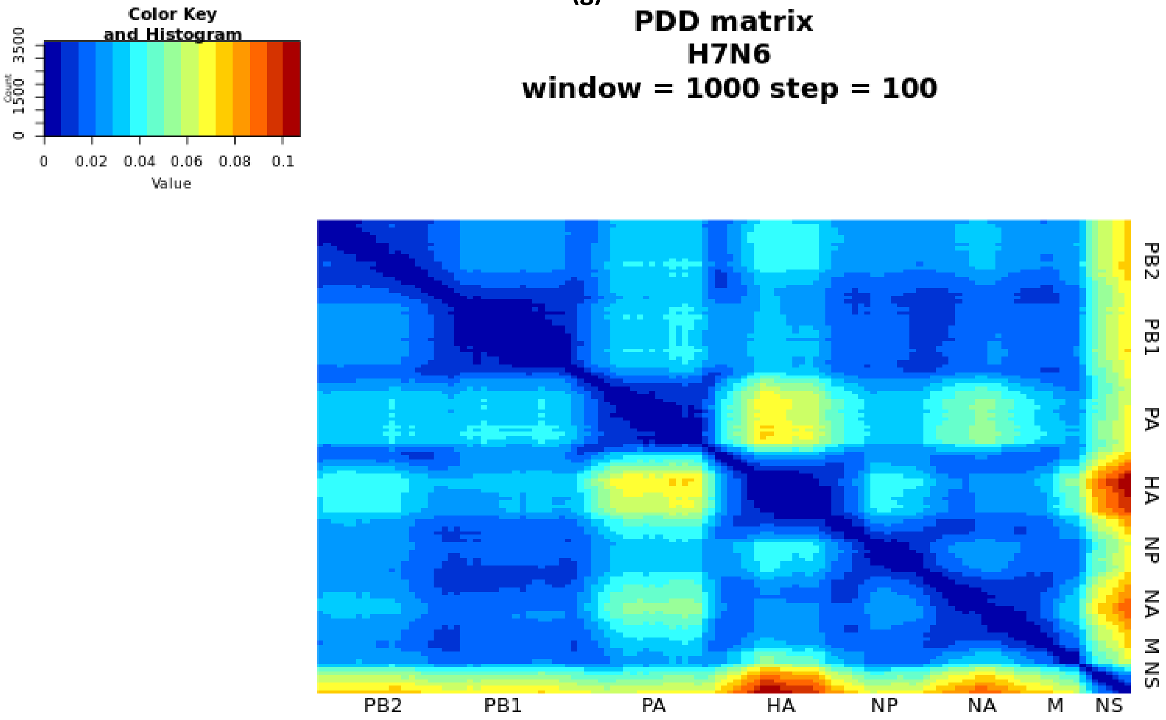
Color Key
and Histogram
Count
0
1500
3500
0
0.02
0.04
0.06
0.08
0.1
Value
PDD matrix
H7N6
window = 1000 step = 100
PB2
PB1
PA
HA
NP
NA
M
NS

(h)

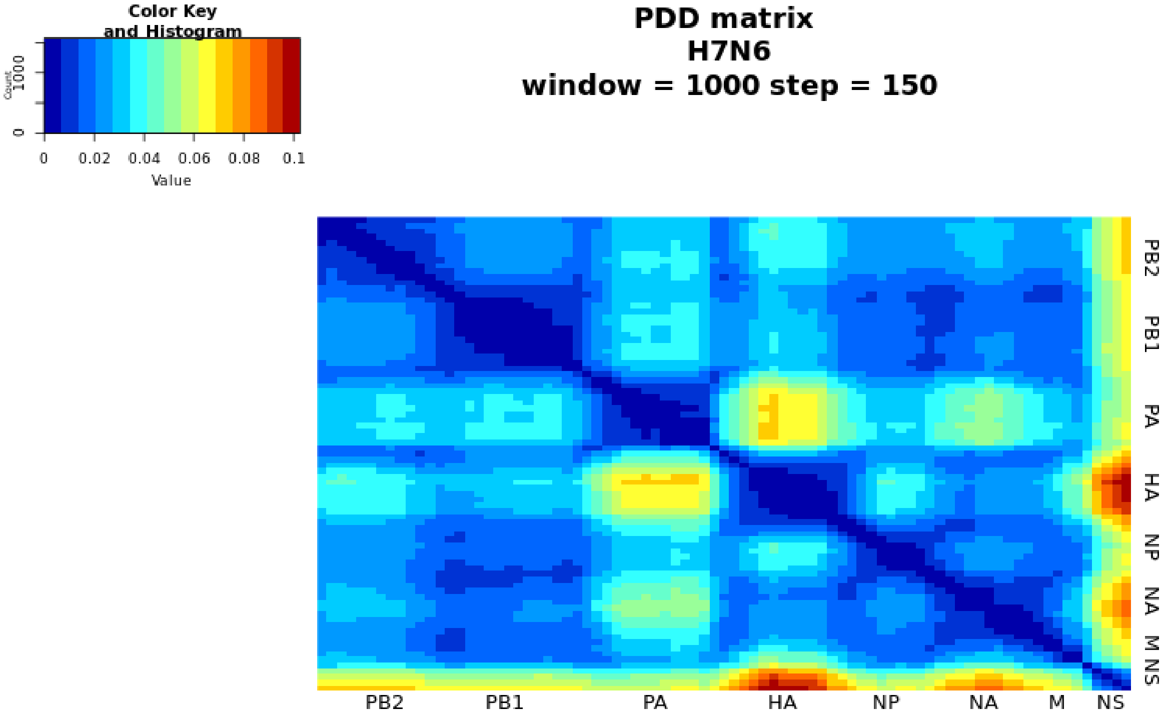

(i)

Supplementary Figure S6. Pairwise distance divergence matrix (PDDM) for H7N6 influenza viruses **(a)** Window = 500 nucleotides, Step = 50 nucleotides **(b)** Window = 500 nucleotides, Step = 100 nucleotides **(c)** Window = 500 nucleotides, Step = 150 nucleotides **(d)** Window = 750 nucleotides, Step = 50 nucleotides **(e)** Window = 750 nucleotides, Step = 100 nucleotides **(f)** Window = 750 nucleotides, Step = 150 nucleotides **(g)** Window = 1000 nucleotides, Step = 50 nucleotides **(h)** Window = 1000 nucleotides, Step = 100 nucleotides **(I)** Window = 1000 nucleotides, Step = 150 nucleotides**.** The color gradient scale shows the root mean square error (RMSE) values in PDCP is a figure.

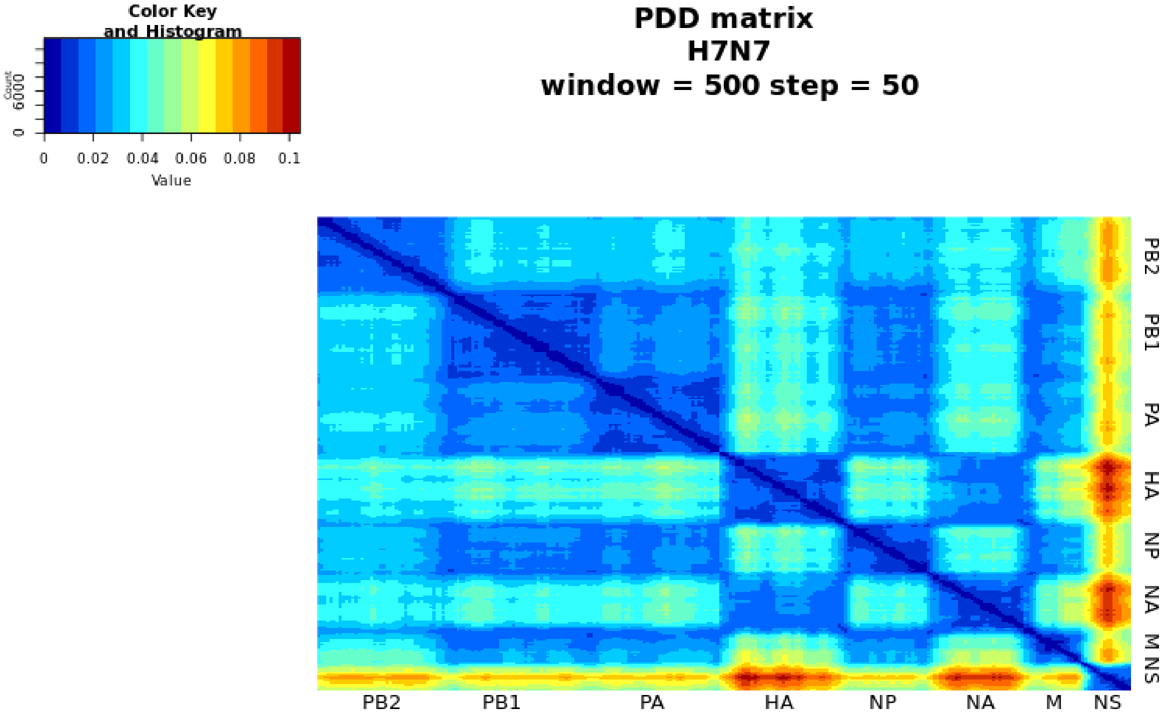
Color Key
and Histogram
Count
0
6000
0
0.02
0.04
0.06
0.08
0.1
Value
PDD matrix
H7N7
window = 500 step = 50
PB2
PB1
PA
HA
NP
NA
M
NS

(a)

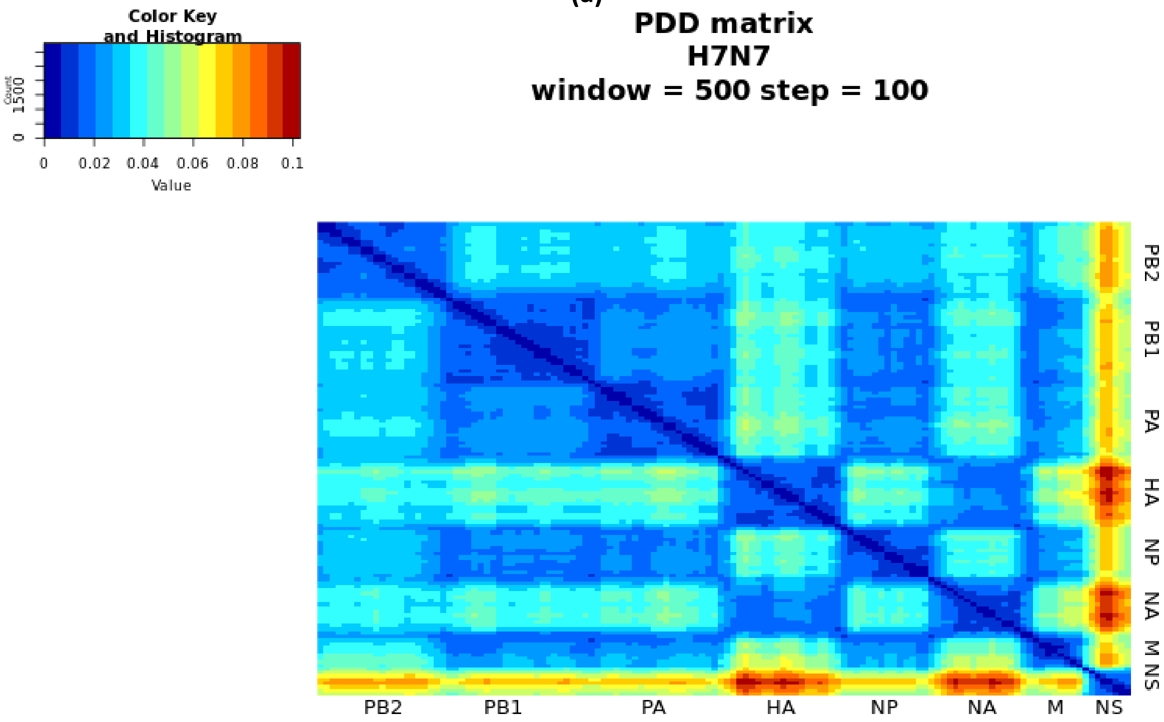
Color Key
and Histogram
Count
0
1500
0
0.02
0.04
0.06
0.08
0.1
Value
PDD matrix
H7N7
window = 500 step = 100
PB2
PB1
PA
HA
NP
NA
M
NS

(b)

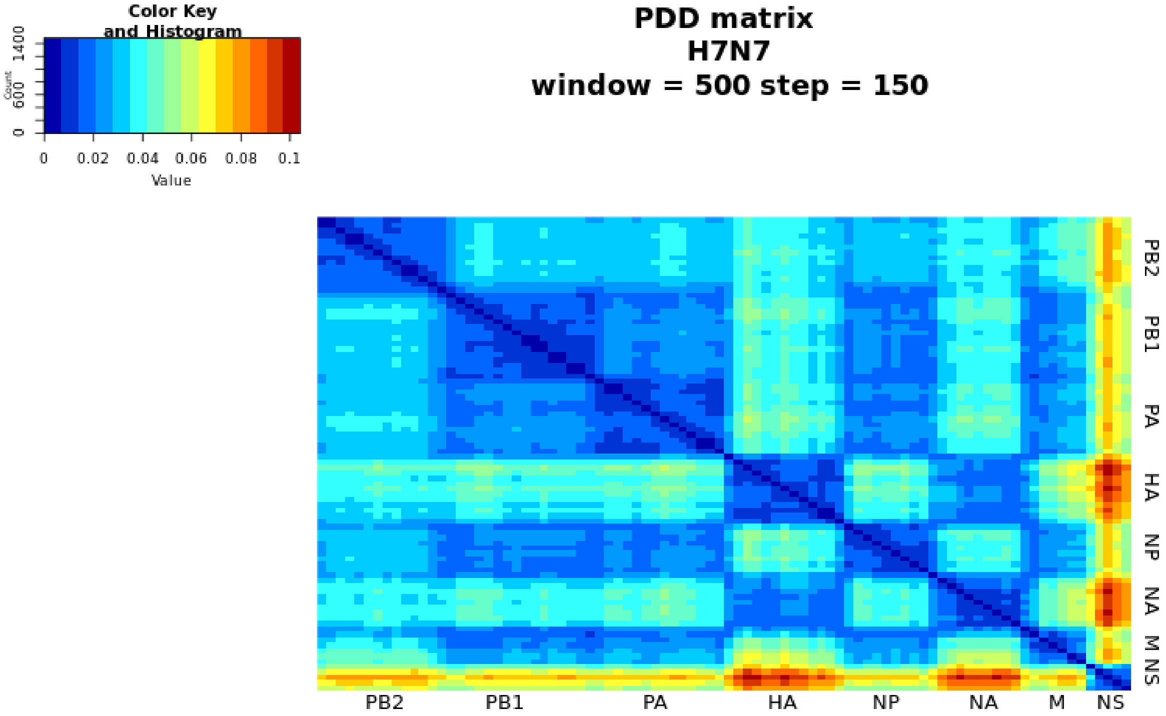
Color Key
and Histogram
Count
0
600
1400
0
0.02
0.04
0.06
0.08
0.1
Value
PDD matrix
H7N7
window = 500 step = 150
PB2
PB1
PA
HA
NP
NA
M
NS

(c)

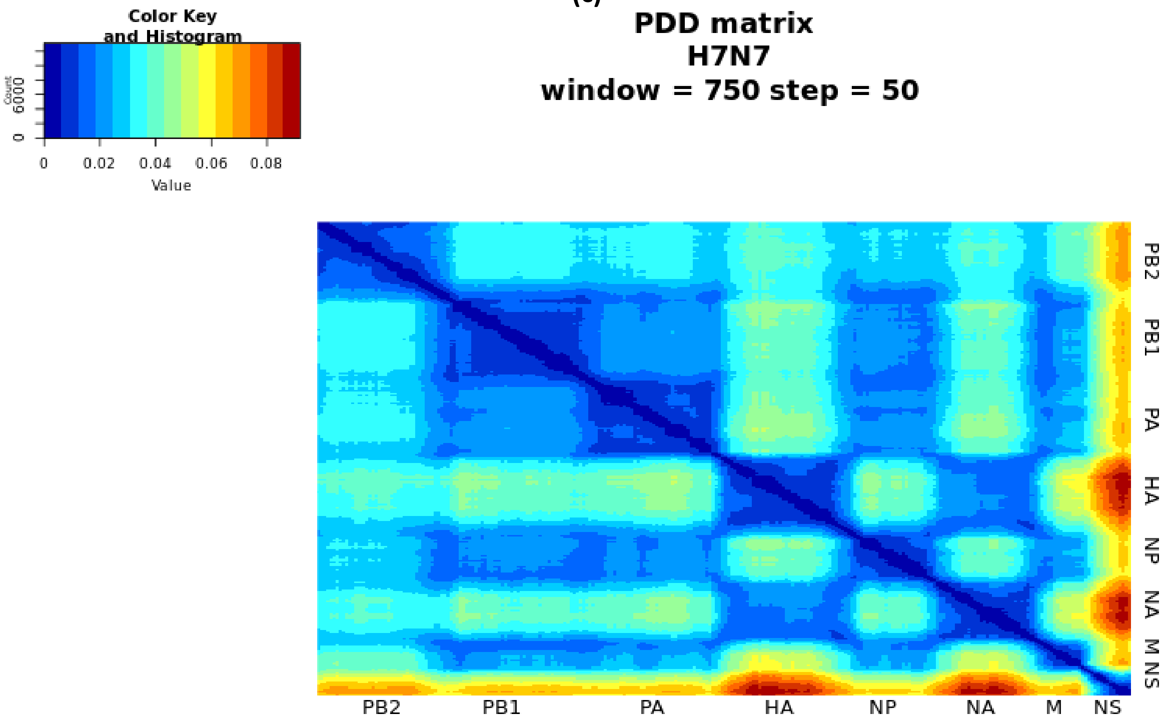
Color Key
and Histogram
Count
0
6000
0
0.02
0.04
0.06
0.08
Value
PDD matrix
H7N7
window = 750 step = 50
PB2
PB1
PA
HA
NP
NA
M
NS

(d)

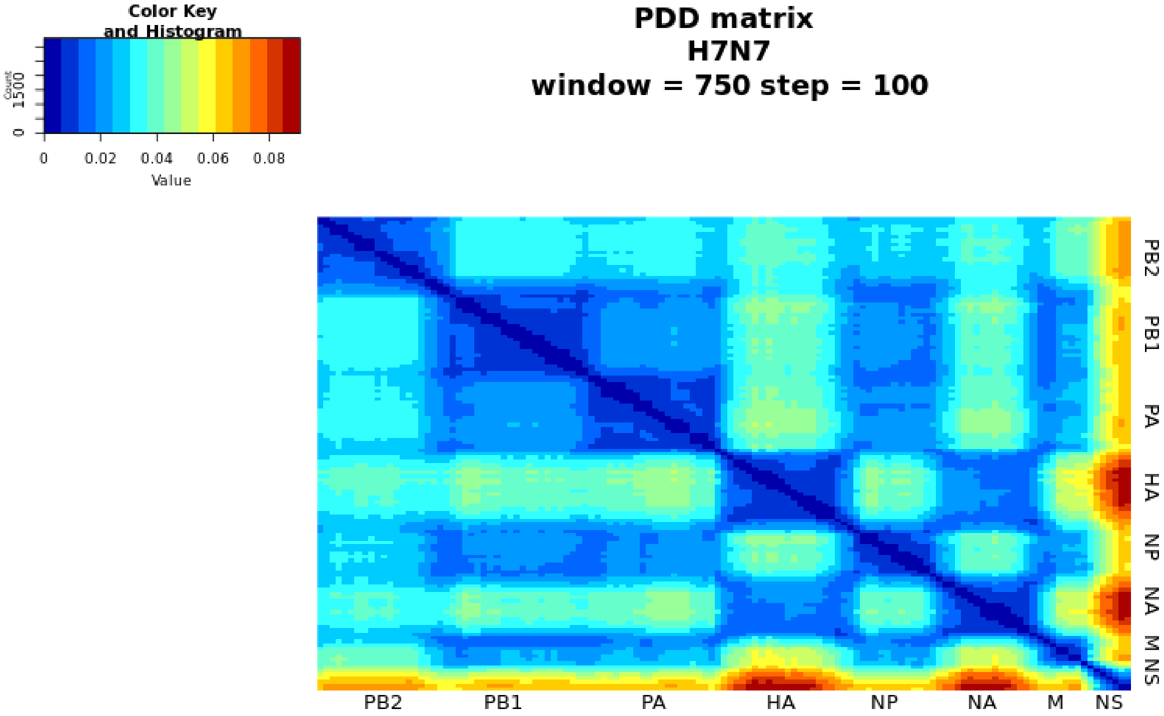
Color Key
and Histogram
Count
0
1500
0
0.02
0.04
0.06
0.08
Value
PDD matrix
H7N7
window = 750 step = 100
PB2
PB1
PA
HA
NP
NA
M
NS
PB2
PB1
PA
HA
NP
NA
M
NS

(e)

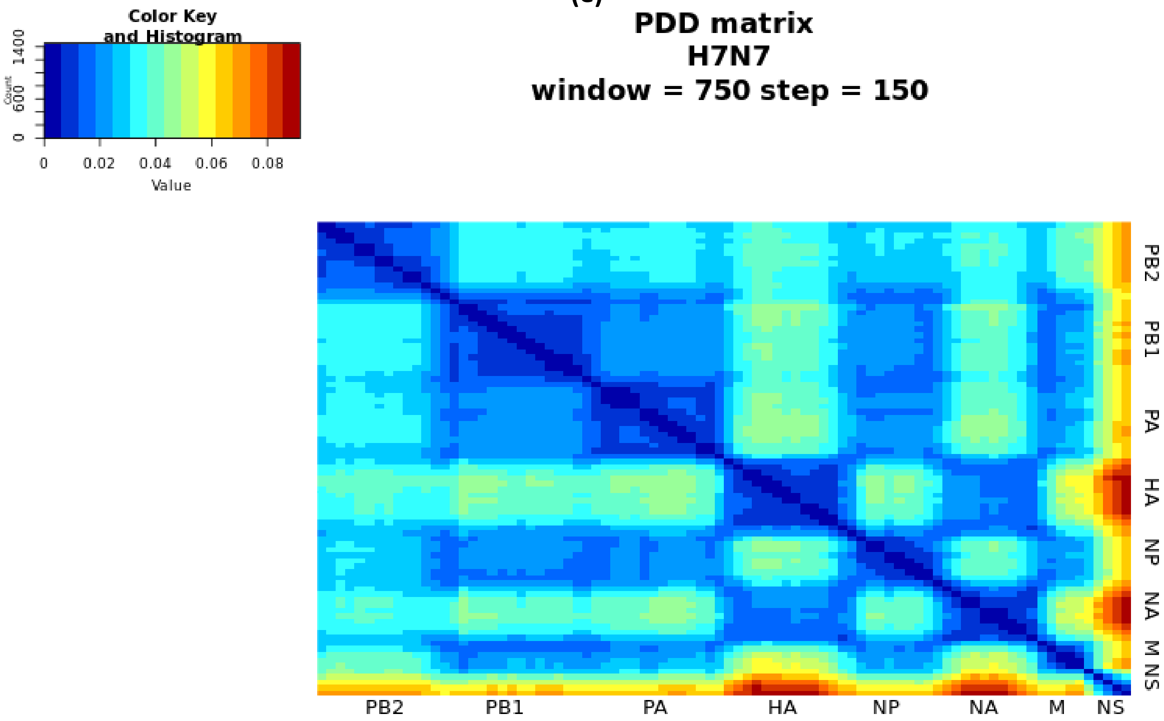
Color Key
and Histogram
Count
0
600
1400
0
0.02
0.04
0.06
0.08
Value
PDD matrix
H7N7
window = 750 step = 150
PB2
PB1
PA
HA
NP
NA
M
NS
PB2
PB1
PA
HA
NP
NA
M
NS

(f)

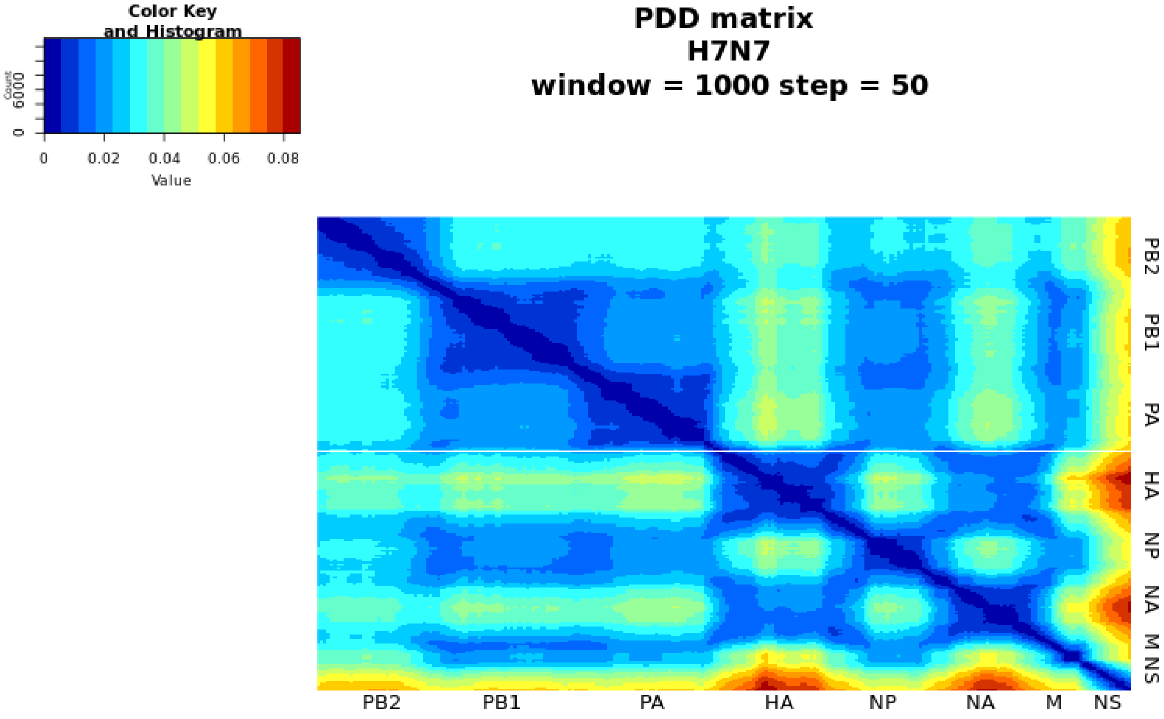
Color Key
and Histogram
Count
0
6000
0
0.02
0.04
0.06
0.08
Value
PDD matrix
H7N7
window = 1000 step = 50
PB2
PB1
PA
HA
NP
NA
M
NS

(g)

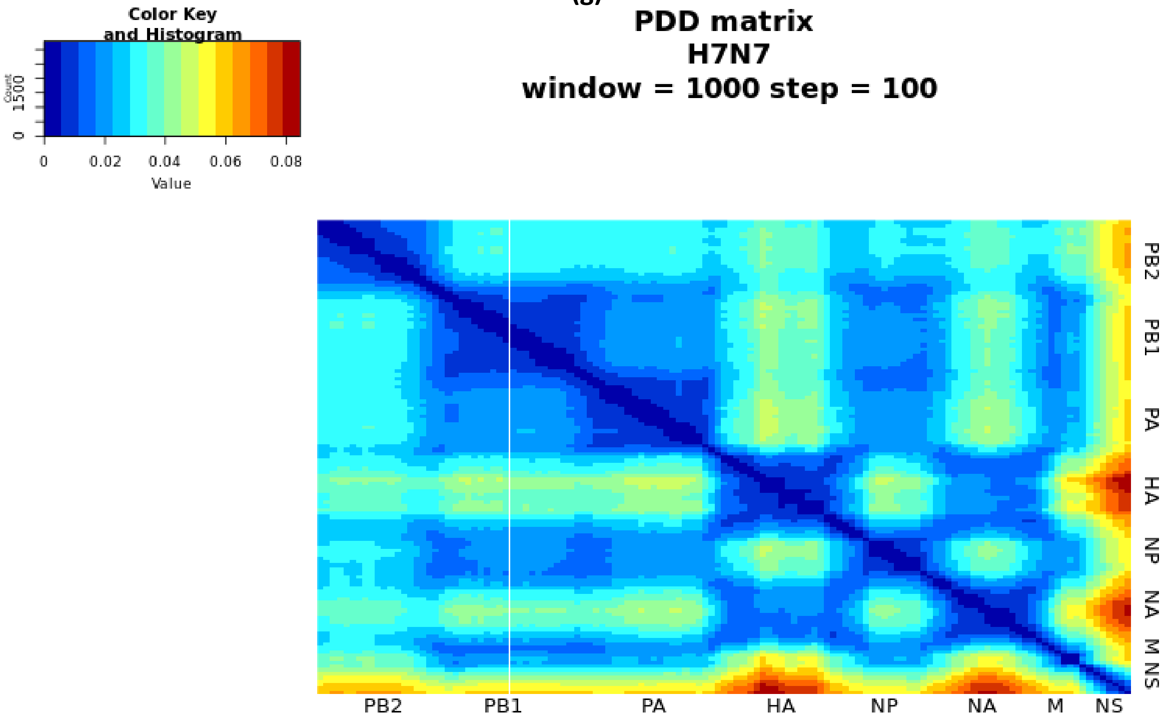
Color Key
and Histogram
Count
0
1500
0
0.02
0.04
0.06
0.08
Value
PDD matrix
H7N7
window = 1000 step = 100
PB2
PB1
PA
HA
NP
NA
M
NS

(h)

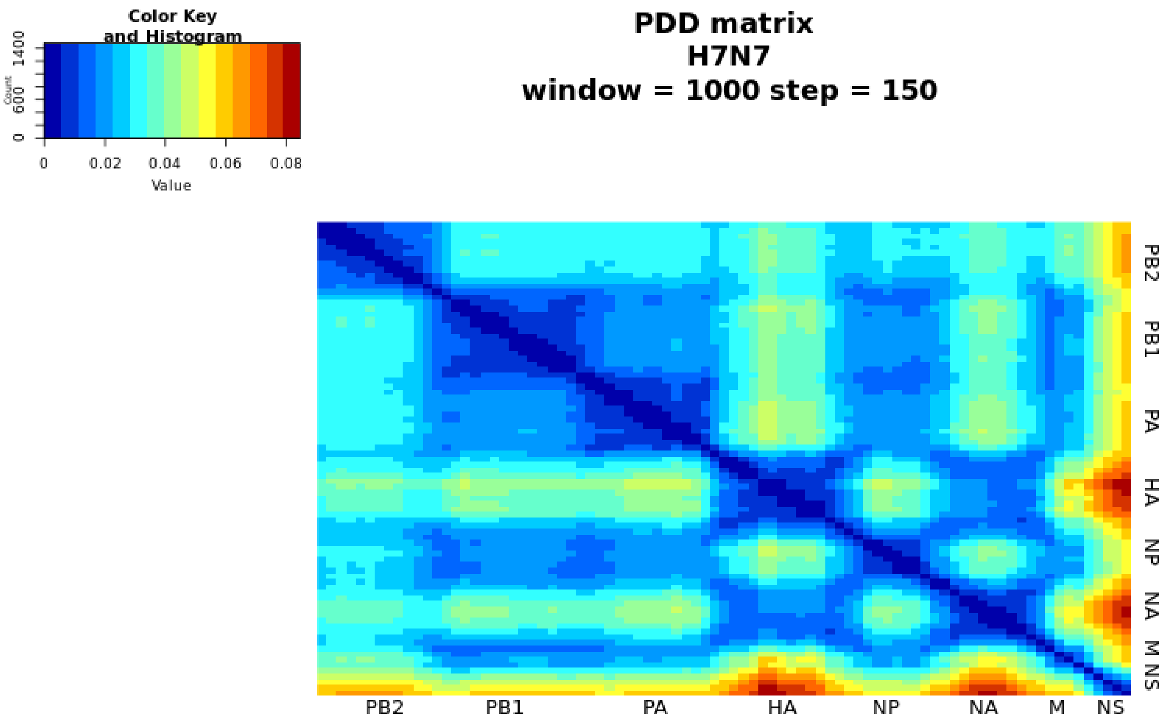

(i)

Supplementary Figure S7. Pairwise distance divergence matrix (PDDM) for H7N7 influenza viruses **(a)** Window = 500 nucleotides, Step = 50 nucleotides **(b)** Window = 500 nucleotides, Step = 100 nucleotides **(c)** Window = 500 nucleotides, Step = 150 nucleotides **(d)** Window = 750 nucleotides, Step = 50 nucleotides **(e)** Window = 750 nucleotides, Step = 100 nucleotides **(f)** Window = 750 nucleotides, Step = 150 nucleotides **(g)** Window = 1000 nucleotides, Step = 50 nucleotides **(h)** Window = 1000 nucleotides, Step = 100 nucleotides **(I)** Window = 1000 nucleotides, Step = 150 nucleotides. The color gradient scale shows the root mean square error (RMSE) values in PDCP is a figure.

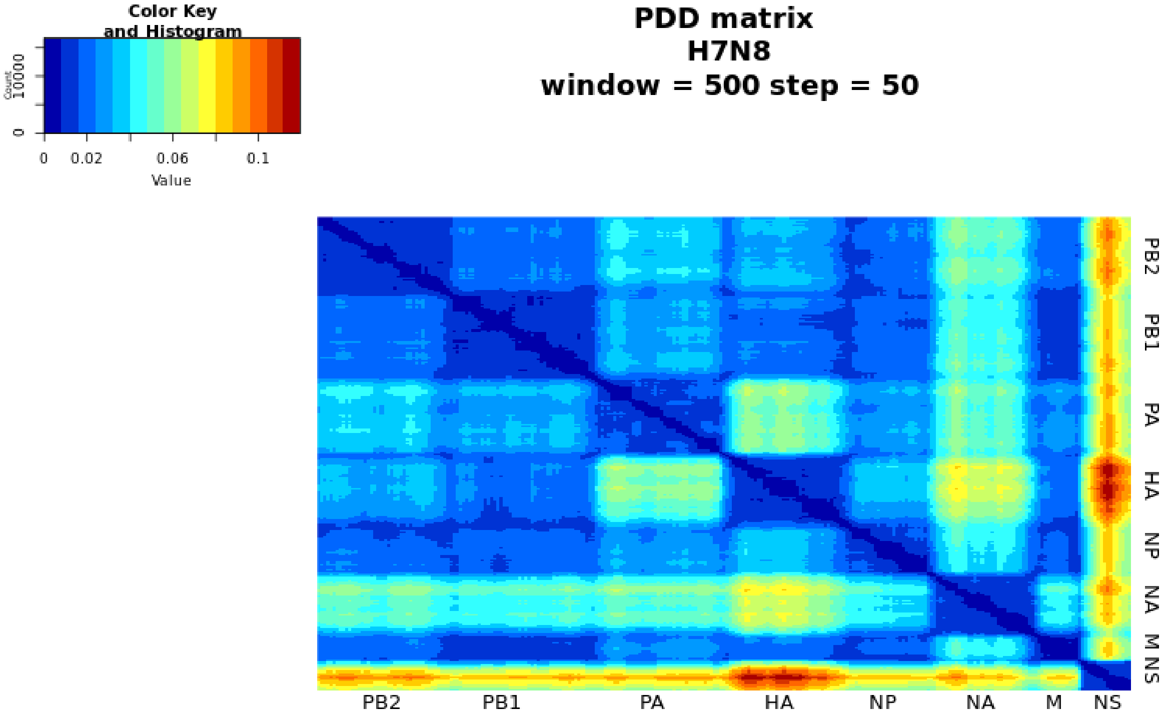
Color Key
and Histogram
Count
10000
0
0
0.02
0.06
0.1
Value
PDD matrix
H7N8
window = 500 step = 50
PB2
PB1
PA
HA
NP
NA
M
NS
PB2
PB1
PA
HA
NP
NA
M
NS

(a)

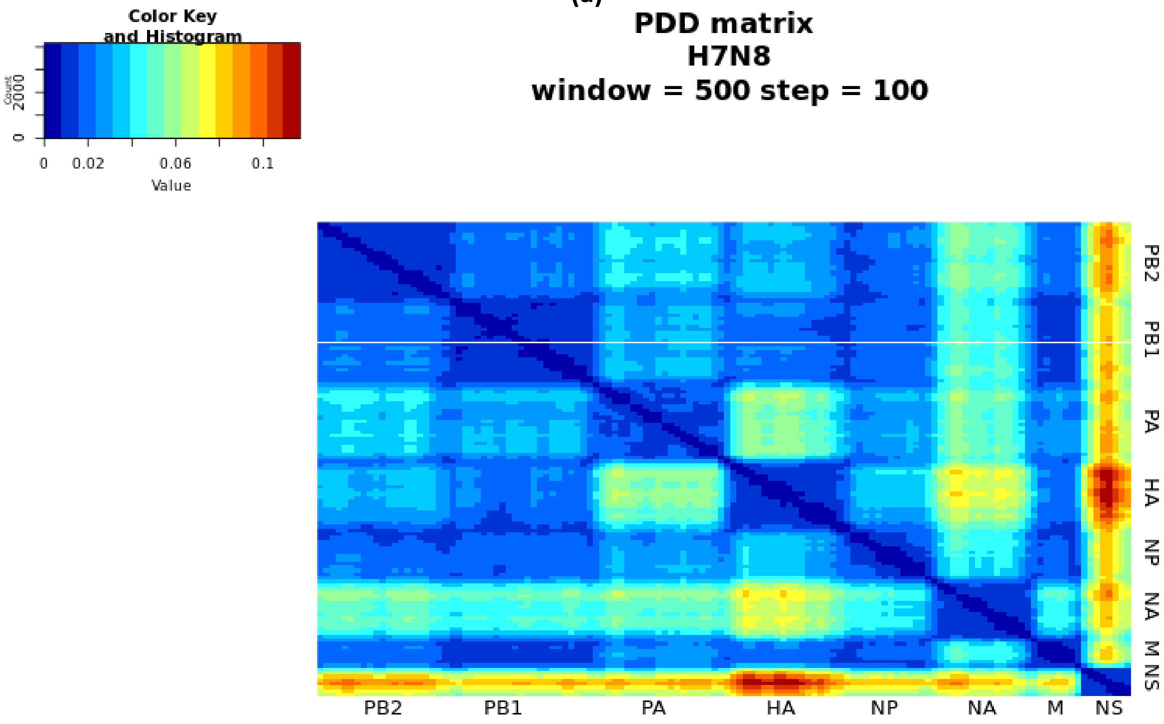
Color Key
and Histogram
Count
2000
0
0
0.02
0.06
0.1
Value
PDD matrix
H7N8
window = 500 step = 100
PB2
PB1
PA
HA
NP
NA
M
NS
PB2
PB1
PA
HA
NP
NA
M
NS

(b)

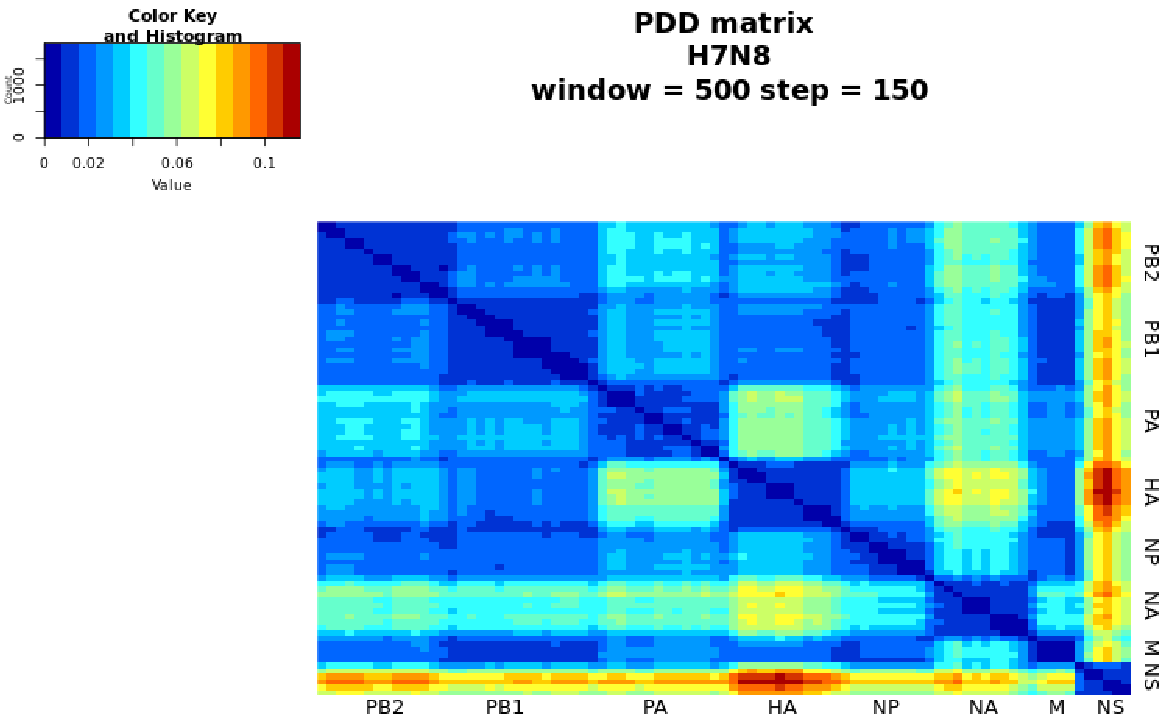
Color Key
and Histogram
Count
1000
0
0
0.02
0.06
0.1
Value
PDD matrix
H7N8
window = 500 step = 150
PB2
PB1
PA
HA
NP
NA
M
NS
PB2
PB1
PA
HA
NP
NA
M
NS

(c)

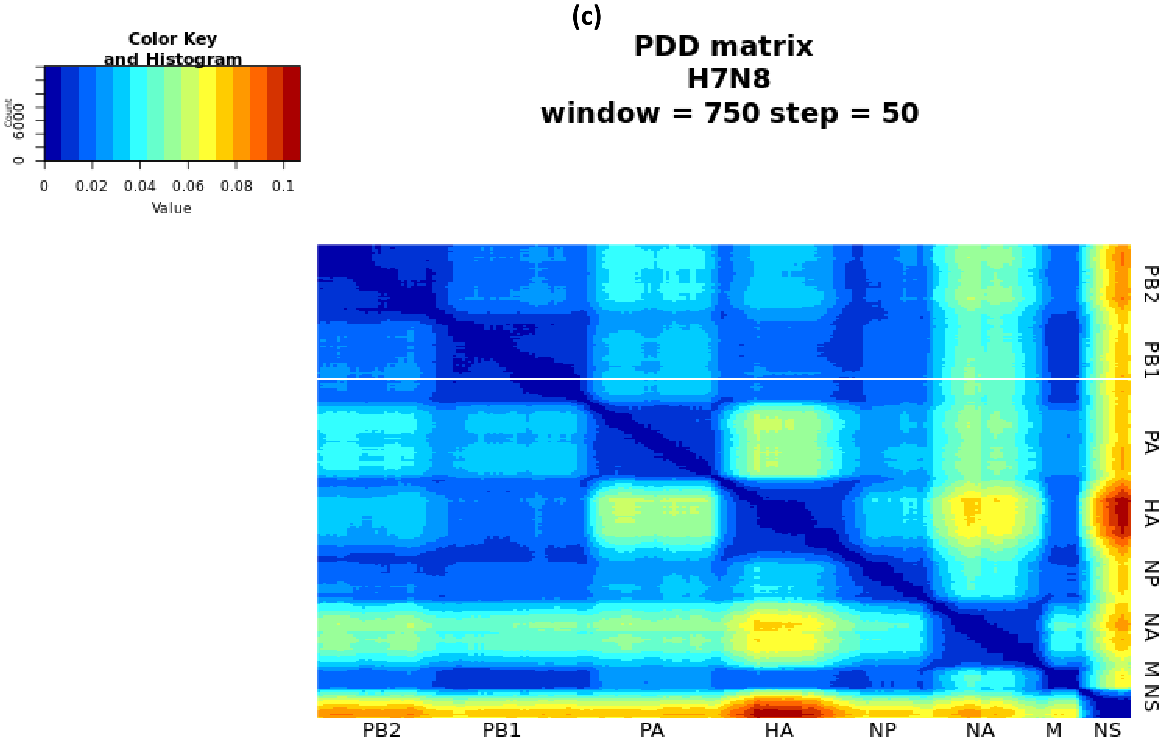
Color Key
and Histogram
Count
6000
0
0
0.02
0.04
0.06
0.08
0.1
Value
PDD matrix
H7N8
window = 750 step = 50
PB2
PB1
PA
HA
NP
NA
M
NS
PB2
PB1
PA
HA
NP
NA
M
NS

(d)

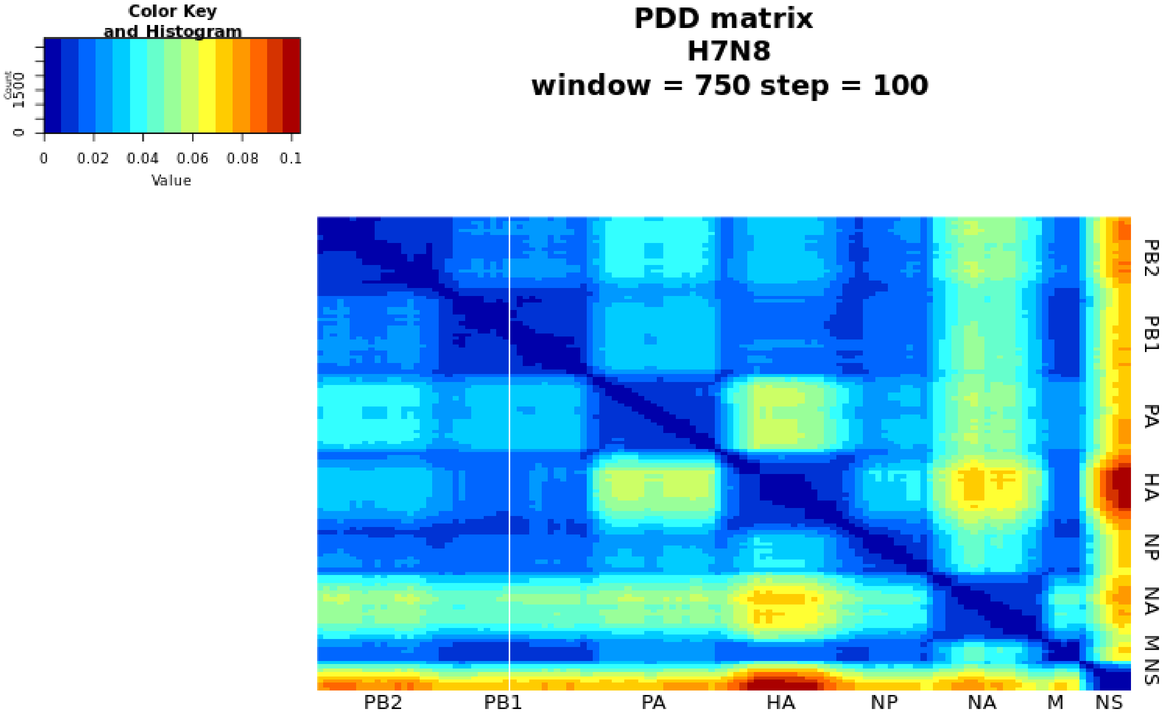
Color Key
and Histogram
Count
0
1500
0
0.02
0.04
0.06
0.08
0.1
Value
PDD matrix
H7N8
window = 750 step = 100
PB2
PB1
PA
HA
NP
NA
M
NS

(e)

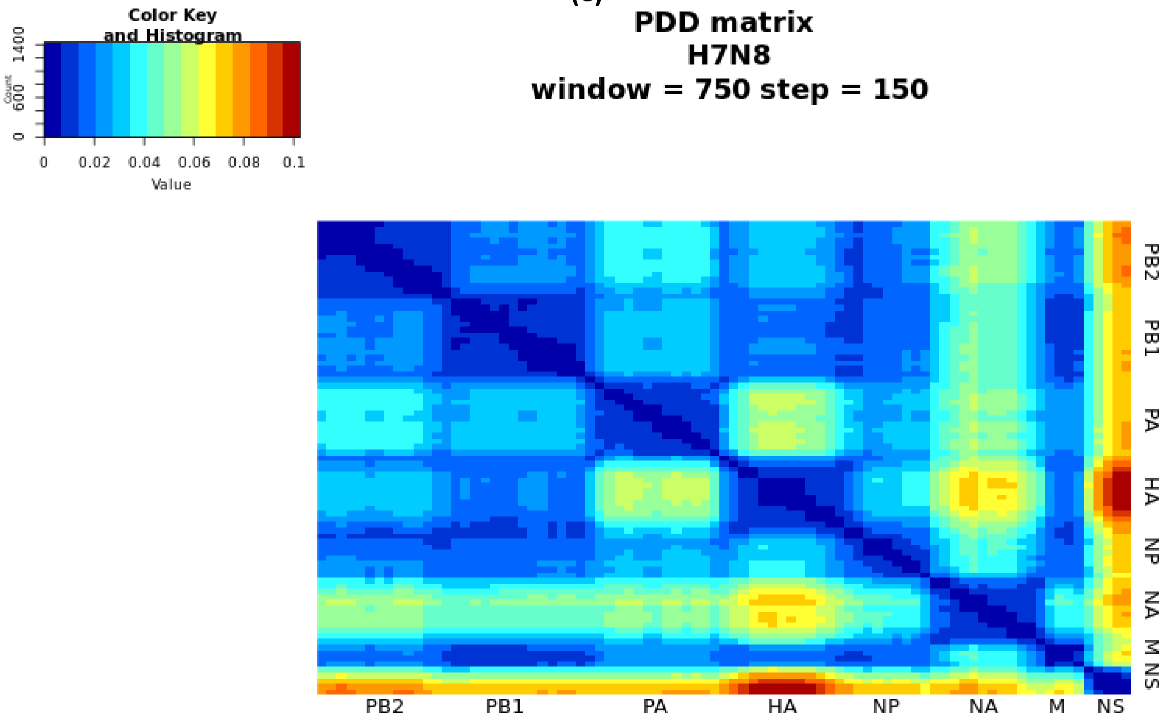
Color Key
and Histogram
Count
0
600
1400
0
0.02
0.04
0.06
0.08
0.1
Value
PDD matrix
H7N8
window = 750 step = 150
PB2
PB1
PA
HA
NP
NA
M
NS

(f)

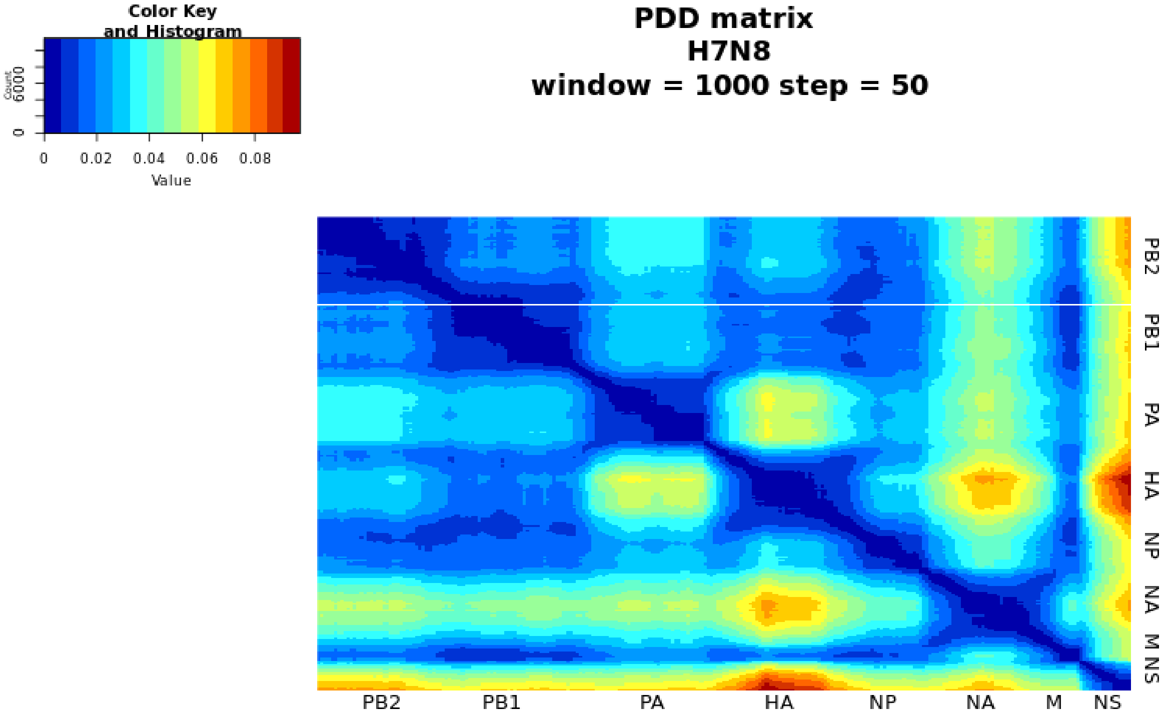

Color Key
and Histogram
Count
6000
0
0
0.02
0.04
0.06
0.08
Value
PDD matrix
H7N8
window = 1000 step = 50
PB2
PB1
PA
HA
NP
NA
M
NS

(g)

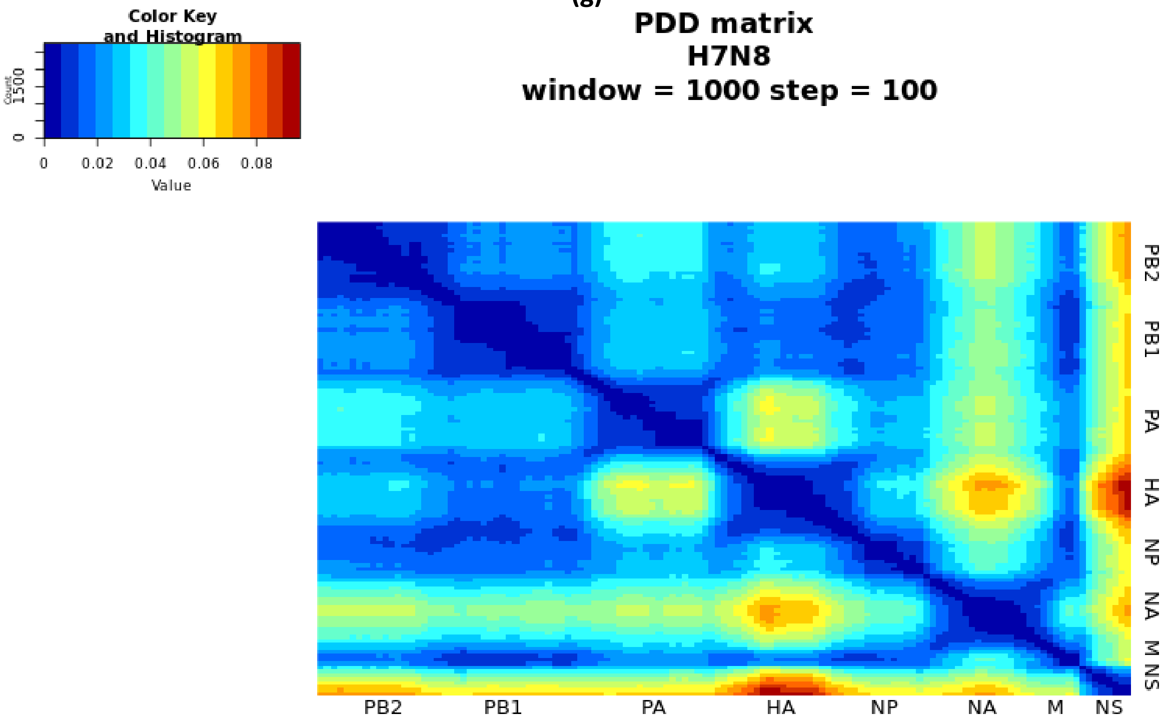

Color Key
and Histogram
Count
1500
0
0
0.02
0.04
0.06
0.08
Value
PDD matrix
H7N8
window = 1000 step = 100
PB2
PB1
PA
HA
NP
NA
M
NS

(h)

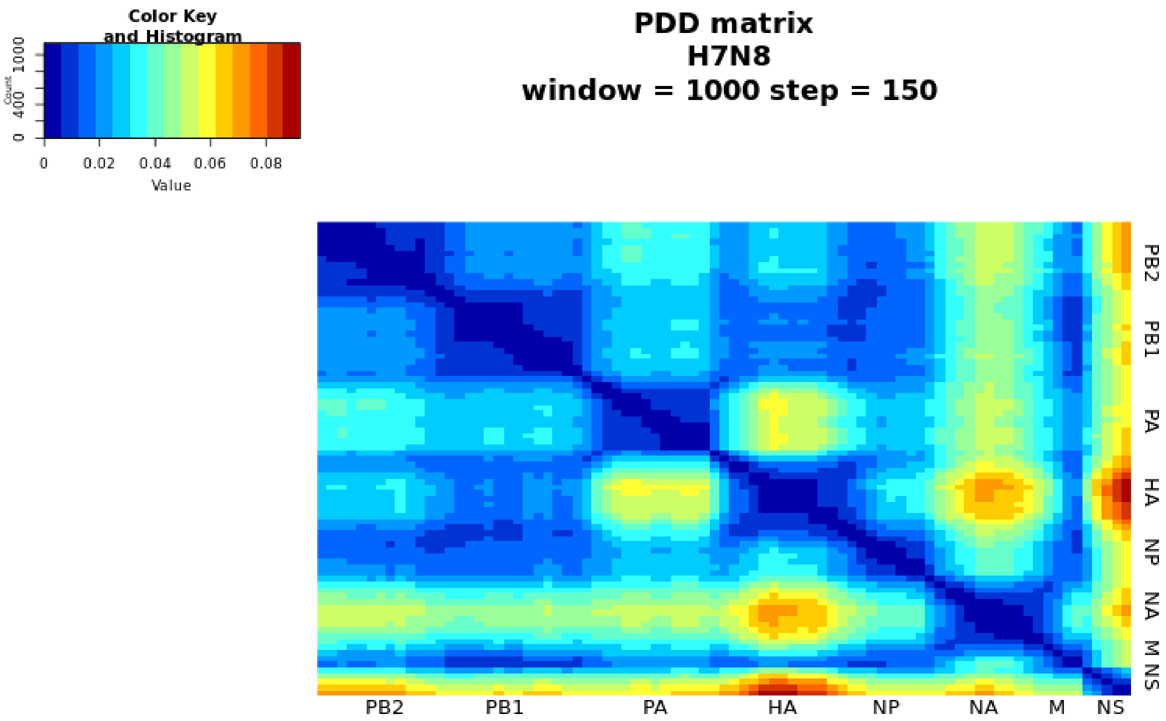

(i)

Supplementary Figure S8. Pairwise distance divergence matrix (PDDM) for H7N8 influenza viruses **(a)** Window = 500 nucleotides, Step = 50 nucleotides **(b)** Window = 500 nucleotides, Step = 100 nucleotides **(c)** Window = 500 nucleotides, Step = 150 nucleotides **(d)** Window = 750 nucleotides, Step = 50 nucleotides **(e)** Window = 750 nucleotides, Step = 100 nucleotides **(f)** Window = 750 nucleotides, Step = 150 nucleotides **(g)** Window = 1000 nucleotides, Step = 50 nucleotides **(h)** Window = 1000 nucleotides, Step = 100 nucleotides **(I)** Window = 1000 nucleotides, Step = 150 nucleotides**.** The color gradient scale shows the root mean square error (RMSE) values in PDCP is a figure.

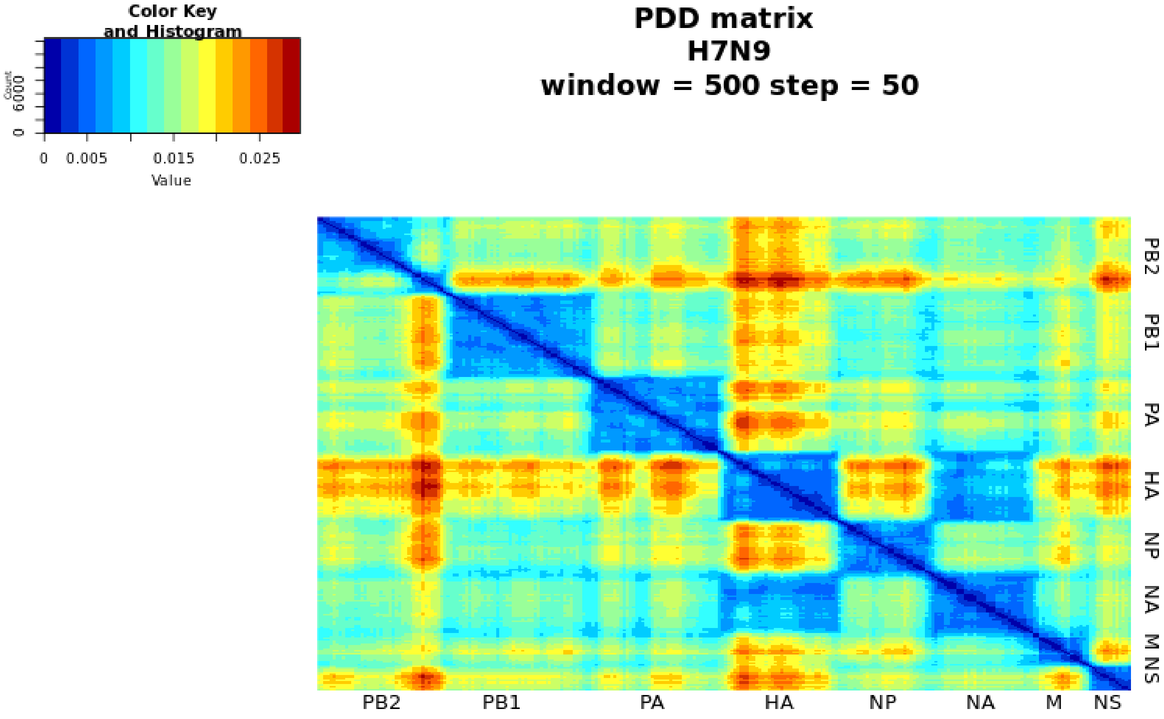

Color Key
and Histogram
Count
0
6000
0
0.005
0.015
0.025
Value
PDD matrix
H7N9
window = 500 step = 50
PB2
PB1
PA
HA
NP
NA
M
NS

(a)

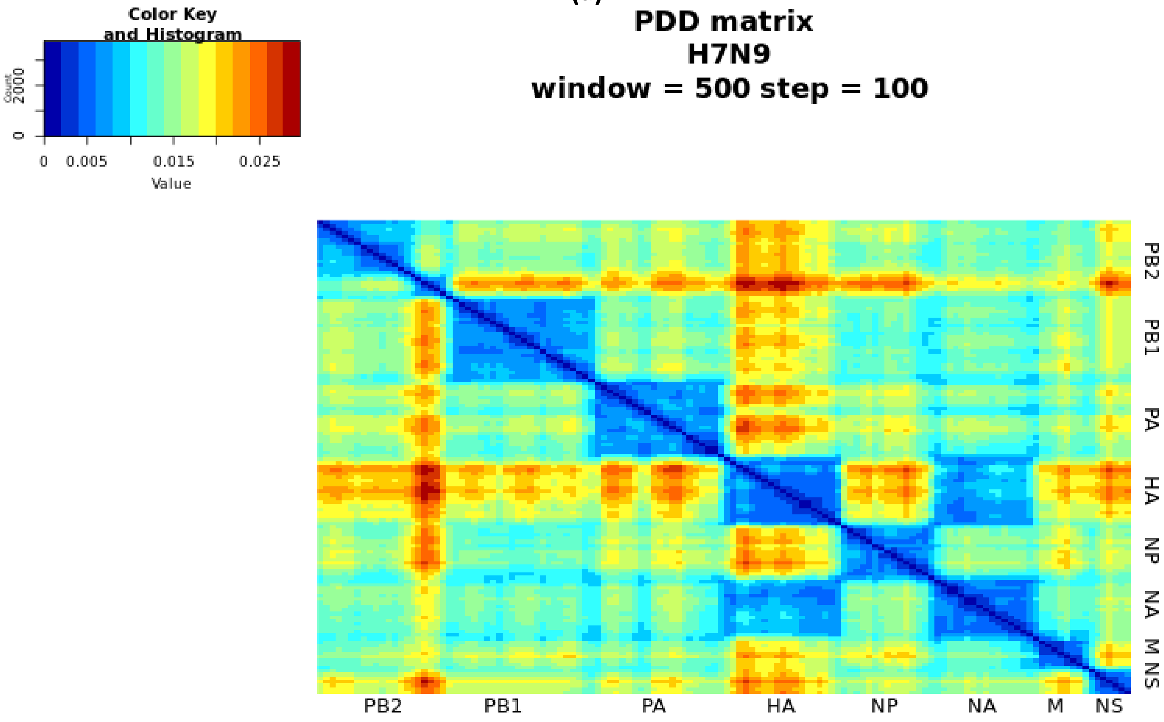

Color Key
and Histogram
Count
0
2000
0
0.005
0.015
0.025
Value
PDD matrix
H7N9
window = 500 step = 100
PB2
PB1
PA
HA
NP
NA
M
NS

(b)

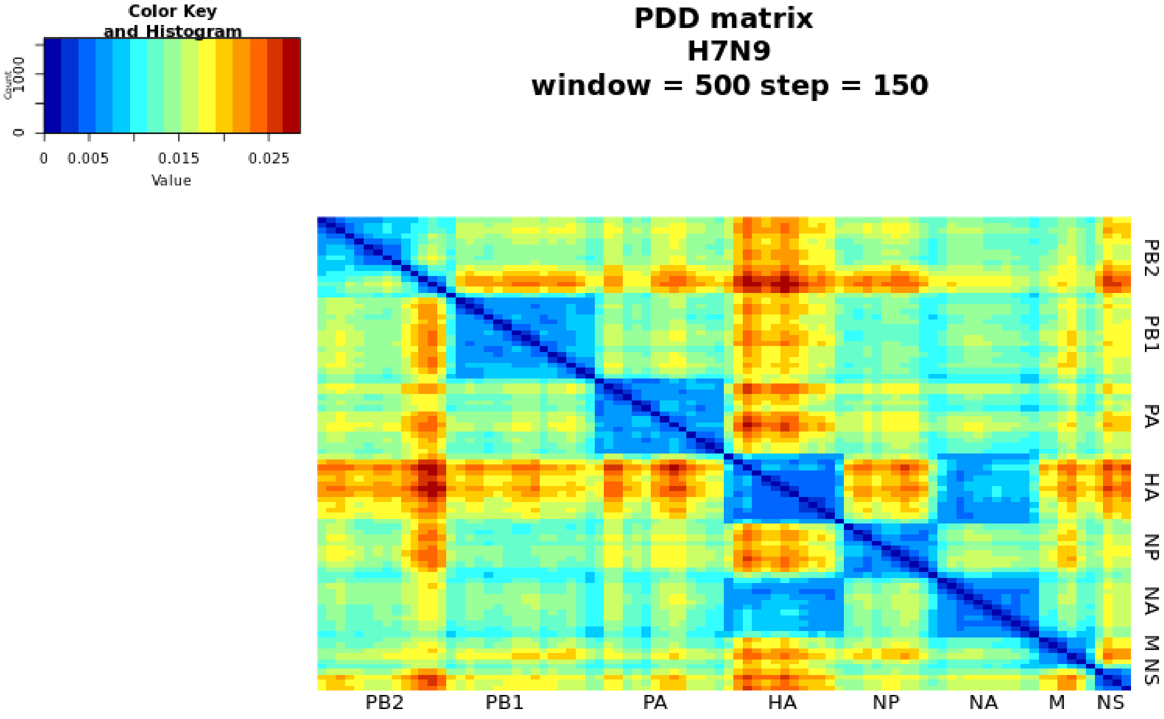
Color Key
and Histogram
Count
1000
0
0
0.005
0.015
0.025
Value
PDD matrix
H7N9
window = 500 step = 150
PB2
PB1
PA
HA
NP
NA
M
NS

(c)

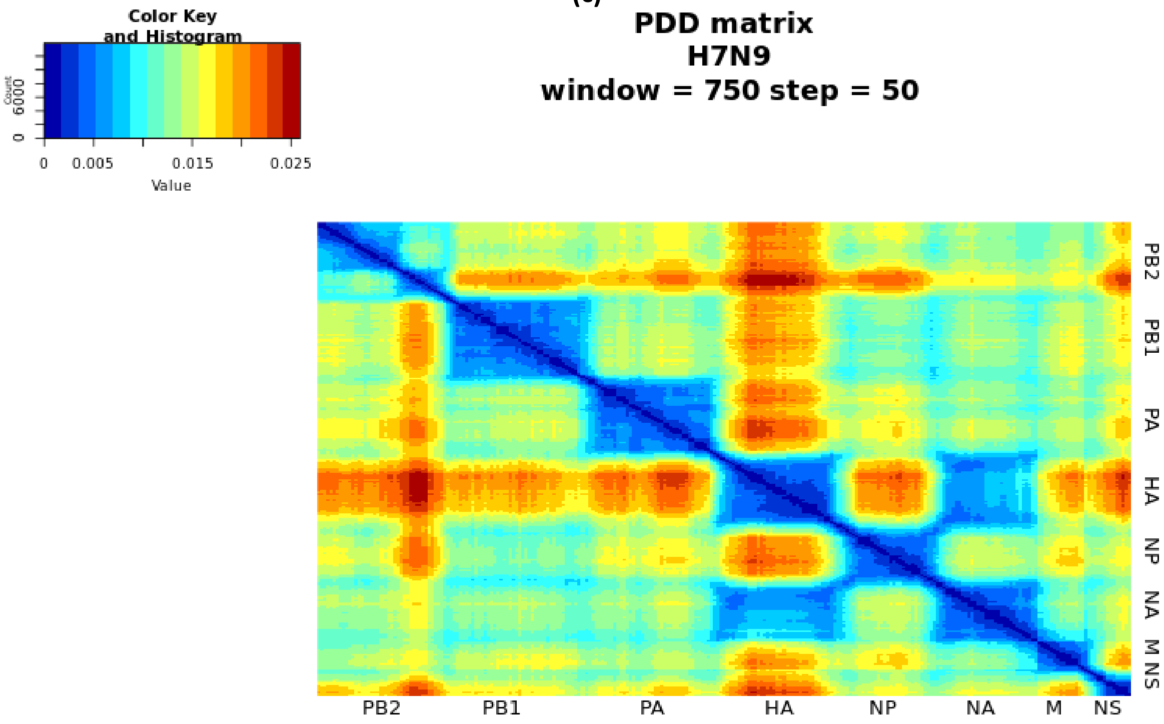
Color Key
and Histogram
Count
6000
0
0
0.005
0.015
0.025
Value
PDD matrix
H7N9
window = 750 step = 50
PB2
PB1
PA
HA
NP
NA
M
NS

(d)

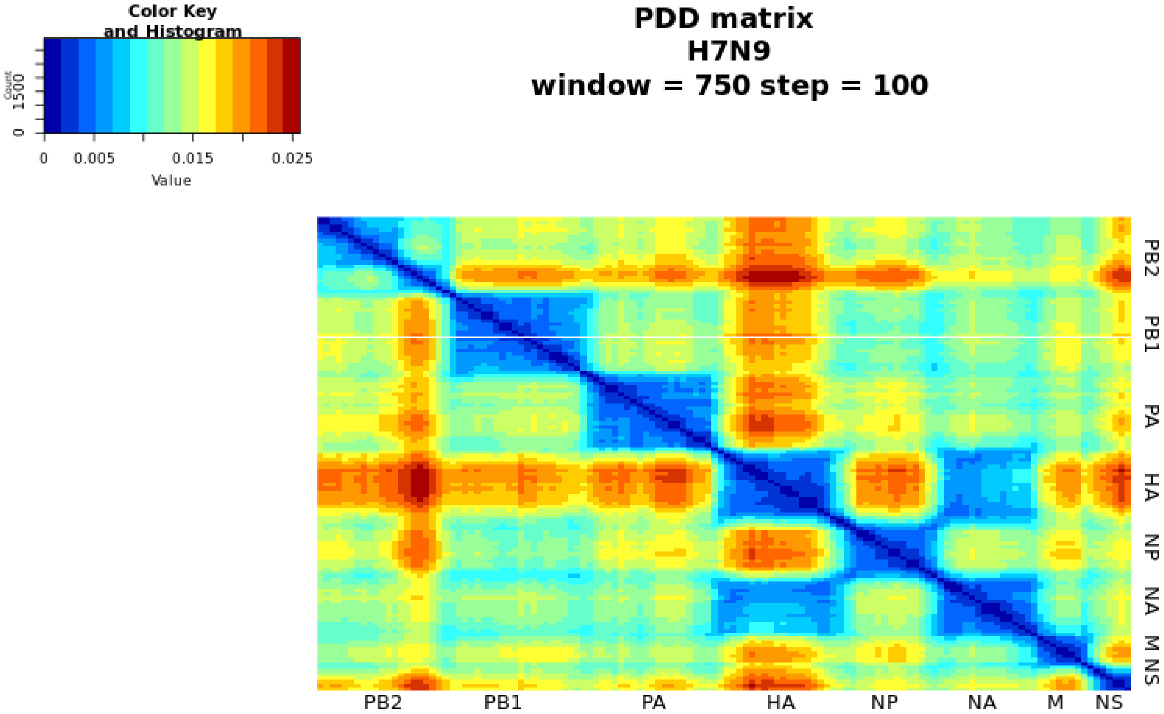
Color Key
and Histogram
Count
0
1500
0
0.005
0.015
0.025
Value
PDD matrix
H7N9
window = 750 step = 100
PB2
PB1
PA
HA
NP
NA
M
NS

(e)

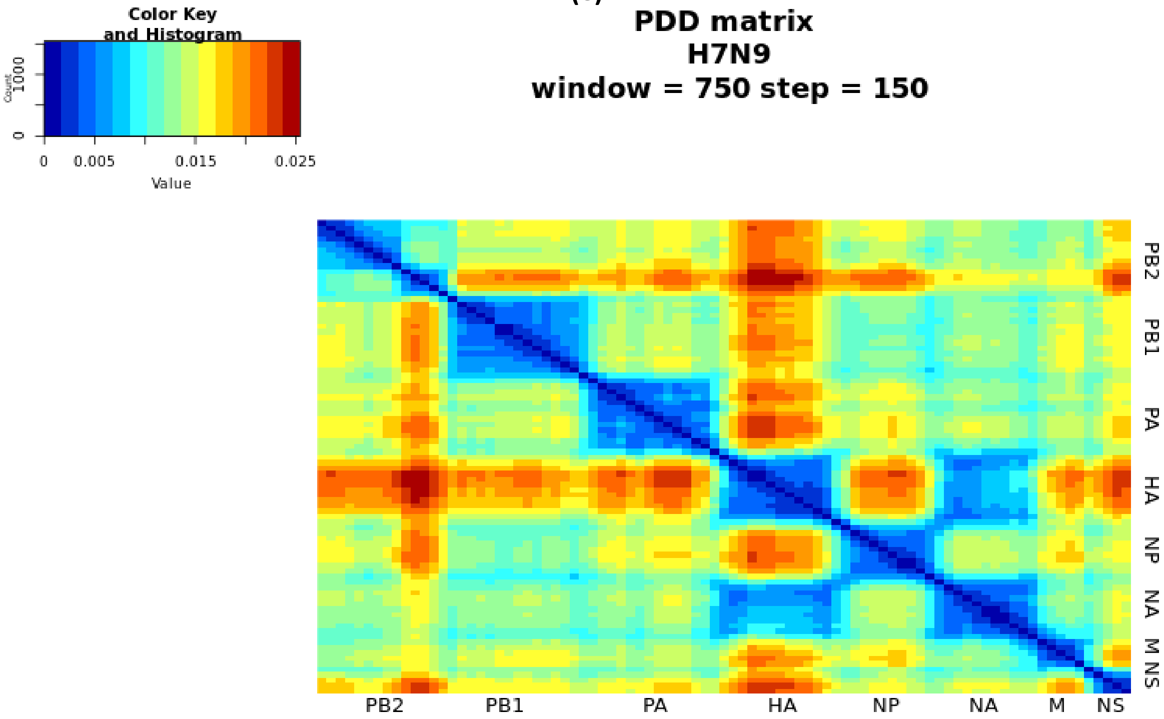
Color Key
and Histogram
Count
0
1000
0
0.005
0.015
0.025
Value
PDD matrix
H7N9
window = 750 step = 150
PB2
PB1
PA
HA
NP
NA
M
NS

(f)

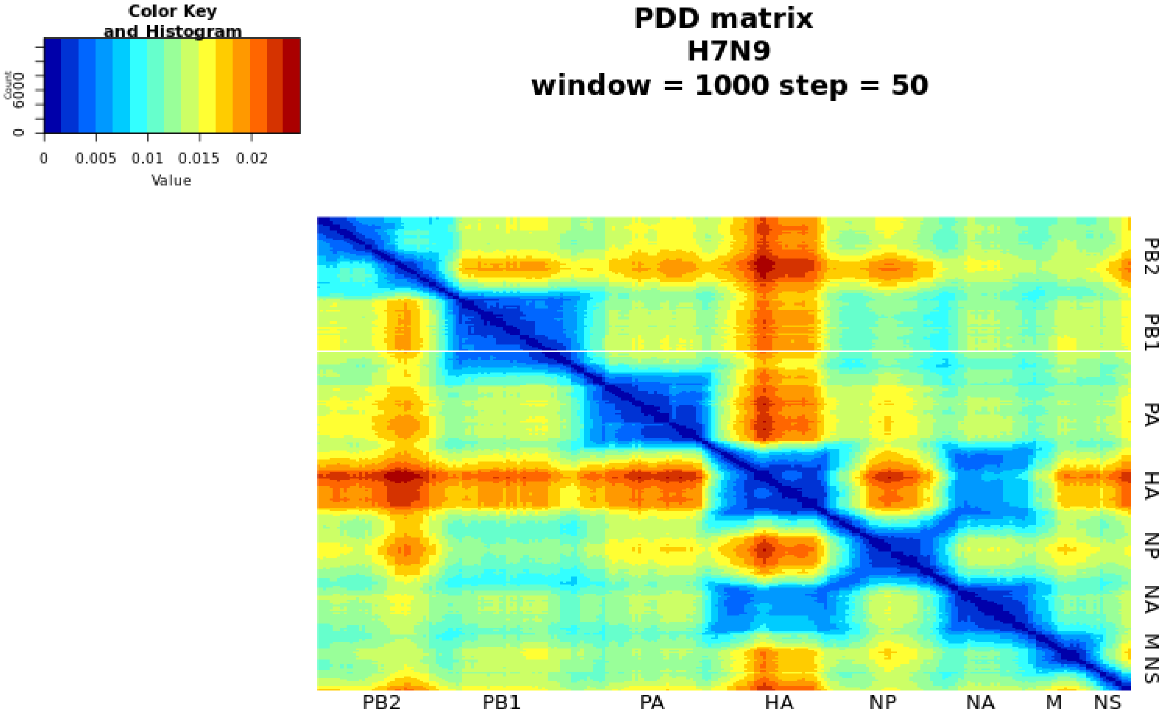
Color Key
and Histogram
Count
0
6000
0
0.005
0.01
0.015
0.02
Value
PDD matrix
H7N9
window = 1000 step = 50
PB2
PB1
PA
HA
NP
NA
M
NS

(g)

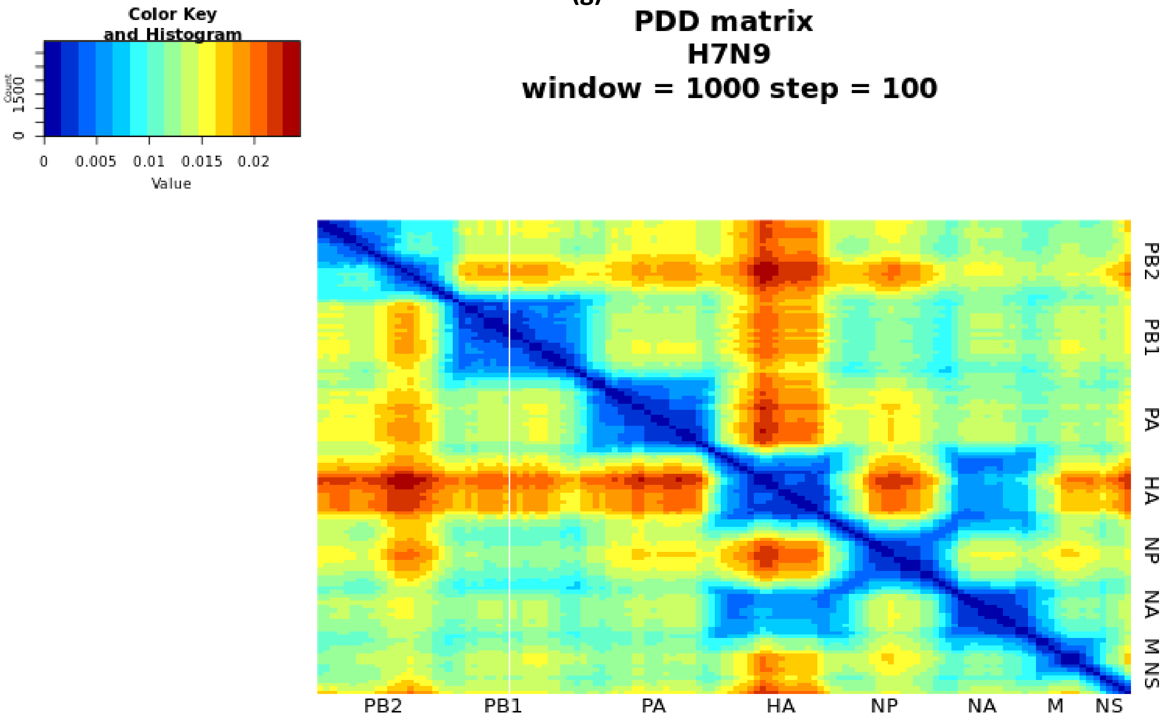
Color Key
and Histogram
Count
0
1500
0
0.005
0.01
0.015
0.02
Value
PDD matrix
H7N9
window = 1000 step = 100
PB2
PB1
PA
HA
NP
NA
M
NS

(h)

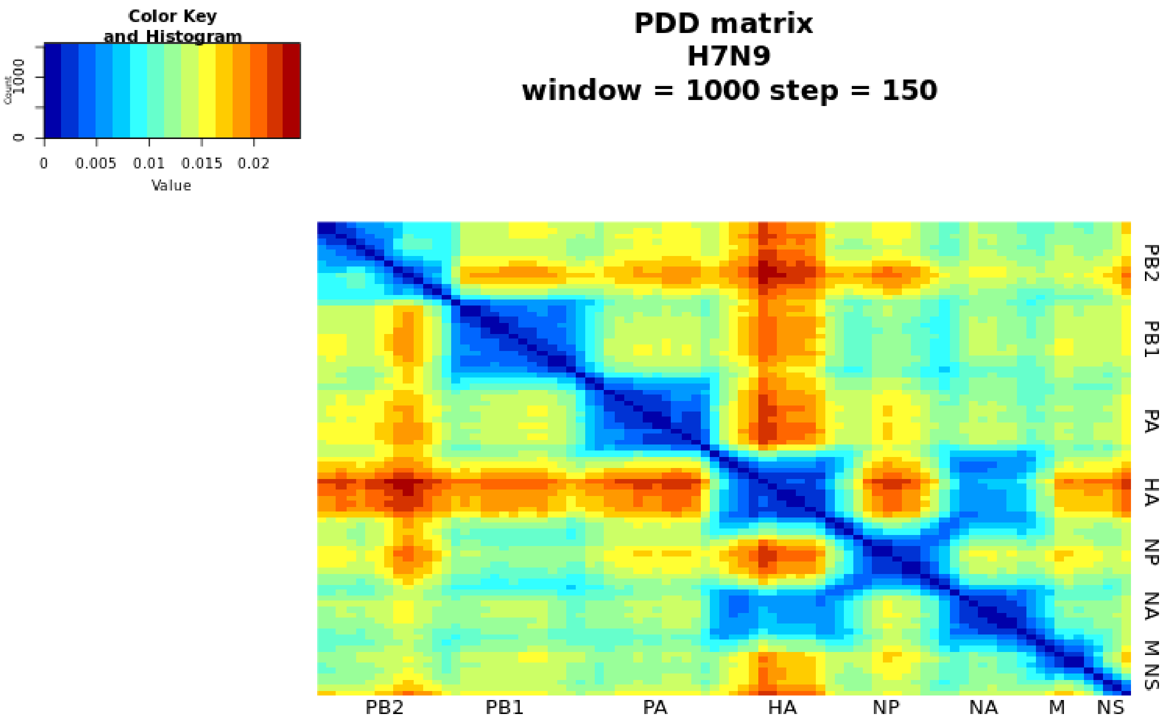

(i)

Supplementary Figure S9. Pairwise distance divergence matrix (PDDM) for H7N9 influenza viruses **(a)** Window = 500 nucleotides, Step = 50 nucleotides **(b)** Window = 500 nucleotides, Step = 100 nucleotides **(c)** Window = 500 nucleotides, Step = 150 nucleotides **(d)** Window = 750 nucleotides, Step = 50 nucleotides **(e)** Window = 750 nucleotides, Step = 100 nucleotides **(f)** Window = 750 nucleotides, Step = 150 nucleotides **(g)** Window = 1000 nucleotides, Step = 50 nucleotides **(h)** Window = 1000 nucleotides, Step = 100 nucleotides **(I)** Window = 1000 nucleotides, Step = 150 nucleotides. The color gradient scale shows the root mean square error (RMSE) values in PDCP is a figure.
