# Supplementary material for: Phylogenetic Insights into H7Nx Influenza Viruses: Uncovering Reassortment Patterns and Geographic Variability
Source: Viruses. 2024 Oct 23;16(11):1656. doi: 10.3390/v16111656 (PMC11598867; doi:10.3390/v16111656)
Supplement: Supplementary file 1 [file viruses-16-01656-s001.zip › Supplementary Table S1.pdf]

Supplementary Table S1. Names of the H7Nx virus strains analyzed in the study (n=3239).

A/mallard/Alberta/34/2001	mallard
A/turkey/Italy/4169/1999	turkey
A/chicken/Rostock/45/1934	chicken
A/mallard duck/Netherlands/31/2012	mallard_duck
A/bean goose/Netherlands/1/2009	bean_goose
A/bewicks swan/Netherlands/7/2008	bewicks_swan
A/mallard duck/Netherlands/44/2011	mallard_duck
A/mallard duck/Netherlands/43/2011	mallard_duck
A/mallard duck/Netherlands/60/2008	mallard_duck
A/mallard duck/Netherlands/77/2008	mallard_duck
A/mallard duck/Netherlands/45/2011	mallard_duck
A/mallard duck/Netherlands/48/2011	mallard_duck
A/mallard duck/Netherlands/40/2011	mallard_duck
A/mallard duck/Netherlands/61/2008	mallard_duck
A/mallard duck/Netherlands/78/2008	mallard_duck
A/mallard duck/Netherlands/86/2008	mallard_duck
A/turkey/Italy/3466/1999	turkey
A/sacred ibis/South Africa/120701/2012	sacred_ibis
A/green-winged teal/Illinois/17OS1208/2017	green-winged_teal
A/common pochard/XiangHai/420/2010	common_pochard
A/turkey/Italy/4042/1999	turkey
A/ostrich/South Africa/OUD/2012	ostrich
A/ostrich/South Africa/FWS/2012	ostrich
A/softbill/CA/33445-158/1992	softbill
A/turkey/Italy/2732/1999	turkey
A/turkey/Italy/3283/1999	turkey
A/turkey/Italy/4482/1999	turkey
A/turkey/Italy/2379/2000	turkey
A/blue-winged teal/Texas/578575/2002	blue-winged_teal
A/blue-winged teal/Texas/578588/2002	blue-winged_teal
A/northern shoveler/Arkansas/12OS158/2012	northern_shoveler
A/blue-winged teal/Texas/AI12-909/2012	blue-winged_teal
A/ring-necked duck/Texas/A00766403/2009	ring-necked_duck
A/American green winged teal/Utah/A00461135/2009	American_green_winged_teal
A/Tadorna tadorna/Belgium/3441-P3/2009	Tadorna_tadorna
A/mallard/Tennessee/AH0039713/2016	mallard
A/turkey/Italy/1084/2000	turkey
A/turkey/Italy/4708/1999	turkey
A/pekin duck/Italy/1848/2000	pekin_duck
A/turkey/Italy/3489/1999	turkey
A/ostrich/Italy/1038/2000	ostrich
A/turkey/Italy/4426/2000	turkey
A/chicken/Italy/322/2001	chicken
A/duck/Italy/551/2000	duck
A/chicken/Italy/1082/1999	chicken

A/chicken/Italy/2335/2000	chicken
A/turkey/Italy/3488/1999	turkey
A/turkey/Italy/4644/1999	turkey
A/quail/Italy/4992/1999	quail
A/chicken/Italy/4789/1999	chicken
A/turkey/Italy/3560/1999	turkey
A/duck/Mongolia/867/2002	duck
A/duck/Bangladesh/24706/2015	duck
A/duck/Bangladesh/24694/2015	duck
A/turkey/Italy/4294/1999	turkey
A/turkey/Italy/2715/1999	turkey
A/turkey/Italy/1265/1999	turkey
A/turkey/Italy/1067/1999	turkey
A/turkey/Italy/1351/2001	turkey
A/turkey/Italy/3185/1999	turkey
A/American green winged teal/Utah/A00461136/2009	American_green_winged_teal
A/ostrich/South Africa/ORD/2012	ostrich
A/blue-winged teal/Ohio/13OS2037/2013	blue-winged_teal
A/chicken/Italy/5086/1999	chicken
A/ruddy shelduck/Mongolia/921C2/2009	ruddy_shelduck
A/common goldeneye/Mongolia/1271/2010	common_goldeneye
A/ruddy shelduck/Mongolia/1189/2010	ruddy_shelduck
A/ruddy shelduck/Mongolia/1209/2010	ruddy_shelduck
A/chicken/Italy/1279/1999	chicken
A/common teal/Hong Kong/MPM1645/2011	common_teal
A/turkey/Italy/32/2001	turkey
A/turkey/Italy/4944/2000	turkey
A/chicken/Italy/797/2000	chicken
A/mallard x black duck/Massachusetts/AH0011261/2015	mallard_x_black_duck
A/aquatic bird/South Korea/sw001/2015	aquatic_bird
A/turkey/Italy/148/2000	turkey
A/turkey/Italy/1085/1999	turkey
A/turkey/Italy/5222/2000	turkey
A/turkey/Italy/1083/1999	turkey
A/turkey/Italy/2732/1999	turkey
A/blue-winged_teal/Texas/578575/2002	blue-winged_teal
A/turkey/Italy/4580/99	turkey
A/turkey/Italy/3675/99	turkey
A/blue-winged_teal/Texas/578588/2002	blue-winged_teal
A/turkey/Italy/977/99	turkey
A/blue-winged_teal/Ohio/13OS2037/2013	blue-winged_teal
A/ostrich/Italy/1038/2000	ostrich
A/turkey/Italy/3185/99	turkey
A/turkey/Italy/4644/99	turkey
A/turkey/Italy/4294/99	turkey
A/turkey/Italy/3489/99	turkey
A/turkey/Italy/3560/99	turkey

A/turkey/Italy/2715/99	turkey
A/turkey/Italy/1265/99	turkey
A/turkey/Italy/4829/1999	turkey
A/mallard/Alberta/34/2001	mallard
A/Rhea/North_Carolina/39482/93	Rhea
A/common_teal/Hong_Kong/MPM1645/2011	common_teal
A/turkey/Italy/4169/99	turkey
A/duck/Mongolia/867/2002	duck
A/chicken/Italy/2335/2000	chicken
A/turkey/Italy/1084/2000	turkey
A/turkey/Italy/4708/1999	turkey
A/northern_shoveler/Arkansas/12OS158/2012	northern_shoveler
A/turkey/Italy/3488/1999	turkey
A/pekin_duck/Italy/1848/2000	pekin_duck
A/ostrich/South_Africa/FWS/2012	ostrich
A/softbill/CA/33445-158/1992	softbill
A/ostrich/South_Africa/ORD/2012	ostrich
A/sacred_ibis/South_Africa/120701/2012	sacred_ibis
A/ostrich/South_Africa/OUD/2012	ostrich
A/turkey/Italy/1351/2001	turkey
A/blue-winged_teal/Texas/AI12-909/2012	blue-winged_teal
A/chicken/Rostock/45/1934	chicken
A/turkey/Italy/4426/2000	turkey
A/chicken/Italy/322/2001	chicken
A/shoveler/Egypt/00597-NAMRU3/2004	shoveler
A/duck/Italy/551/2000	duck
A/duck/Mongolia/47/01	duck
A/chicken/Italy/797/2000	chicken
A/chicken/Italy/1082/1999	chicken
A/quail/Italy/4992/1999	quail
A/chicken/Italy/4789/1999	chicken
A/swine/KU/16/2001	swine
A/American green-winged teal/Texas/AH0057366/2016	American_green-winged_teal
A/feline/New York/WVDL-14/2016	feline
A/feline/New York/WVDL-16/2016	feline
A/feline/New York/WVDL-20/2016	feline
A/feline/New York/WVDL-3/2016	feline
A/feline/New York/WVDL-9/2016	feline
A/mallard/Kentucky/AH00729344/2016	mallard
A/mallard/Utah/AH0020535/2015	mallard
A/feline/New York/16-040082-1/2016	feline
A/duck/Hokkaido/W19/2013	duck
A/New York/107/2003	human
A/chicken/NJ/294508-12/2004	chicken
A/chicken/CT/260413-2/2003	chicken
A/environment/NY/260422-10/2003	environment
A/guinea fowl/NJ/119063-8/2001	guinea_fowl

A/unknown/NY/120485-5/2001	unknown
A/ruddy turnstone/New Jersey/563/2006	ruddy_turnstone
A/bufflehead/Virginia/A00120022/2008	bufflehead
A/American green-winged teal/Texas/AH0058651/2016	American_green-winged_teal
A/environment/New York/16326-1/2005	environment
A/environment/New York/19501-1/2006	environment
A/guinea fowl/New York/22067-10/2005	guinea_fowl
A/chicken/New York/16330/2005	chicken
A/chicken/New York/22067-7/2005	chicken
A/chukar/New York/11653-1/2005	chukar
A/chicken/New York/22067-9/2005	chicken
A/environment/New York/11678-2/2005	environment
A/chicken/New York/16326-5/2005	chicken
A/duck/New York/21211-6/2005	duck
A/chicken/New York/11678-5/2005	chicken
A/chicken/New York/21211-1/2005	chicken
A/chukar/New York/21211-7/2005	chukar
A/environment/New York/22067-3/2005	environment
A/environment/New York/16326-2/2005	environment
A/environment/New York/139052/2005	environment
A/chicken/New York/8391-2/2006	chicken
A/chicken/New York/16326-4/2005	chicken
A/environment/New York/23165-4/2005	environment
A/chicken/New York/31621-9/2005	chicken
A/environment/New York/37982-1/2005	environment
A/guinea fowl/New York/37982-3/2005	guinea_fowl
A/muscovy duck/New York/23165-13/2005	muscovy_duck
A/chicken/New York/30732-9/2005	chicken
A/chicken/New York/30732-10/2005	chicken
A/environment/New York/31621-2/2005	environment
A/duck/New York/22067-11/2005	duck
A/environment/New York/23165-5/2005	environment
A/environment/New York/31621-1/2005	environment
A/mallard/Korea/GJ62/2007	mallard
A/chicken/New York/23165-9/2005	chicken
A/duck/New York/143646-5/2005	duck
A/duck/Italy/4609/2003	duck
A/environment/New York/22067-5/2005	environment
A/guinea fowl/New York/23165-12/2005	guinea_fowl
A/quail/New York/30732-12/2005	quail
A/muscovy duck/New York/30732-13/2005	muscovy_duck
A/environment/New York/37982-2/2005	environment
A/unknown/New York/13479-2/2005	unknown
A/environment/New York/19501-2/2006	environment
A/environment/New York/11678-3/2005	environment
A/environment/New York/11678-4/2005	environment
A/environment/New York/143646-1/2005	environment

A/guinea fowl/New York/143646-3/2005	guinea_fowl
A/pheasant/New York/143646-4/2005	pheasant
A/environment/New York/3181-1/2006	environment
A/chicken/New York/3181-5/2006	chicken
A/environment/New York/3185-2/2006	environment
A/environment/New York/3185-3/2006	environment
A/environment/New York/3185-5/2006	environment
A/guinea fowl/New York/8391-1/2006	guinea_fowl
A/guinea fowl/New York/19501-4/2006	guinea_fowl
A/environment/New York/29047-1/2006	environment
A/environment/New York/29047-2/2006	environment
A/environment/New York/29047-3/2006	environment
A/chicken/New York/29047-4/2006	chicken
A/environment/New York/3185-1/2006	environment
A/environment/New York/22067-4/2005	environment
A/chukar/New York/23164-13/2005	chukar
A/guinea fowl/New York/10196-7/2005	guinea_fowl
A/guinea fowl/New York/22071/2005	guinea_fowl
A/environment/New York/23164-1/2005	environment
A/guinea fowl/New York/23164-3-05/2005	guinea_fowl
A/environment/New York/31621-3/2005	environment
A/chicken/New York/22067-8/2005	chicken
A/chicken/New York/23165-11/2005	chicken
A/environment/New York/31621-5/2005	environment
A/quail/New York/89641/2005	quail
A/environment/New York/3181-2/2006	environment
A/Muscovy duck/New York/11646-4/2005	Muscovy_duck
A/unknown/New York/11675/2005	unknown
A/unknown/New York/13479-3/2005	unknown
A/unknown/New York/13479-5/2005	unknown
A/chicken/New York/19495-2/2006	chicken
A/chicken/New York/19495-3/2006	chicken
A/environment/New York/26792-3/2005	environment
A/unknown/New York/13479-1/2005	unknown
A/Guinea fowl/New York/19495-6/2006	Guinea_fowl
A/chicken/New York/19499-1/2006	chicken
A/environment/New York/30732-1/2005	environment
A/unknown/New York/11646-8/2005	unknown
A/environment/New York/30720-1/2005	environment
A/Northern shoveler/Mississippi/16OS6138/2016	Northern_shoveler
A/quail/Italy/4610/2003	quail
A/environmental/NY/268925-10/2003	environmental
A/chicken/New York/19495-5/2006	chicken
A/environment/New York/23164-2/2005	environment
A/chicken/New York/10508/2005	chicken
A/environment/NY/241365-18/2003	environment
A/environment/New York/30732-3/2005	environment

A/environment/New York/30732-4/2005	environment
A/chicken/New York/10196-5/2005	chicken
A/chicken/New York/23165-10/2005	chicken
A/environment/New York/26792-1/2005	environment
A/environment/New York/26792-4/2005	environment
A/chicken/New York/79672/2005	chicken
A/unknown/New York/11646-9/2005	unknown
A/chicken/New York/23165-6/2005	chicken
A/environment/New York/30720-3/2005	environment
A/environment/New York/30732-2/2005	environment
A/unknown/New York/19501-5/2006	unknown
A/chicken/PA/19241/1997	chicken
A/Guinea fowl/New York/98616-3/2005	Guinea_fowl
A/turkey/New York/122501-2/2005	turkey
A/environment/New York/46545-1/2006	environment
A/environment/New York/10196-1/2005	environment
A/guinea fowl/New York/32084/2006	guinea_fowl
A/environment/New York/10196-3/2005	environment
A/chicken/New York/10196-6/2005	chicken
A/unknown/New York/11646-6/2005	unknown
A/environment/New York/88291-1/2005	environment
A/environment/New York/88291-2/2005	environment
A/chicken/New York/23164-4/2005	chicken
A/chicken/New York/30732-6/2005	chicken
A/duck/New York/98616-5/2005	duck
A/Guinea fowl/New York/101276-1/2005	Guinea_fowl
A/unknown/New York/11646-5/2005	unknown
A/chicken/New York/88291-15/2005	chicken
A/American green-winged teal/Ohio/13OS1769/2013	American_green-winged_teal
A/turkey/New York/30732-7/2005	turkey
A/chicken/New York/10196-4/2005	chicken
A/environment/New York/26792-2/2005	environment
A/environment/New York/19501-3/2006	environment
A/environment/New York/88291-3/2005	environment
A/unknown/New York/13479-4/2005	unknown
A/environment/New York/88291-5/2005	environment
A/duck/New York/88291-13/2005	duck
A/chicken/New York/63806-11/2005	chicken
A/quail/New York/63806-13/2005	quail
A/unknown/New York/88291-17/2005	unknown
A/environment/New York/98616-2/2005	environment
A/environment/New York/81928-2/2005	environment
A/chicken/New York/88291-6/2005	chicken
A/unknown/New York/88291-16/2005	unknown
A/chicken/New York/19499/2005	chicken
A/Guinea fowl/New York/88291-9/2005	Guinea_fowl
A/duck/Korea/A349/09	duck

A/chicken/Jilin/SD020/2014	chicken
A/Anas platyrhynchos/Spain/1877/2009	Anas_platyrhynchos
A/twite/Gangcha/01/2006	twite
A/twite/Gangcha/02/2006	twite
A/tree sparrow/Gangcha/02/2006	tree_sparrow
A/Tibetan snowfinch/Maduo/01/2006	Tibetan_snowfinch
A/tree sparrow/Gangcha/03/2006	tree_sparrow
A/tree sparrow/Gonghe/01/2006	tree_sparrow
A/long-billed calandra/Gonghe/01/2006	long-billed_calandra
A/tree sparrow/Gangcha/01/2006	tree_sparrow
A/ground jay/Maduo/01/2006	ground_jay
A/pika/QH-Maduo/01/2006	pika
A/pika/QH-Maduo/02/2006	pika
A/pika/QH-Maduo/03/2006	pika
A/pika/QH-Maduo/04/2006	pika
A/pika/QH-Gonghe/01/2006	pika
A/pika/QH-Gangcha/01/2006	pika
A/pika/Menyuan/01/2008	pika
A/pika/Maduo/01/2009	pika
A/environment/New_York/31621-1/2005	environment
A/Guinea_fowl/New_York/88291-9/2005	Guinea_fowl
A/chukar/New_York/11653-1/2005	chukar
A/unknown/New_York/88291-16/2005	unknown
A/environment/New_York/11678-2/2005	environment
A/environment/New_York/11678-3/2005	environment
A/unknown/New_York/13479-1/2005	unknown
A/unknown/New_York/13479-2/2005	unknown
A/environment/New_York/11678-4/2005	environment
A/environment/New_York/3185-1/2006	environment
A/duck/Italy/4609/2003	duck
A/environment/New_York/16326-1/2005	environment
A/chicken/New_York/16326-5/2005	chicken
A/environment/New_York/46545-1/2006	environment
A/Guinea_fowl/New_York/19495-6/2006	Guinea_fowl
A/chicken/New_York/16330/2005	chicken
A/environment/New_York/22067-4/2005	environment
A/duck/New_York/21211-6/2005	duck
A/chicken/New_York/19499-1/2006	chicken
A/environment/New_York/22067-5/2005	environment
A/chukar/New_York/23164-13/2005	chukar
A/environment/New_York/19501-3/2006	environment
A/unknown/New_York/19501-5/2006	unknown
A/chicken/New_York/23165-6/2005	chicken
A/environment/New_York/30720-3/2005	environment
A/chicken/NJ/294508-12/2004	chicken
A/environment/New_York/30732-2/2005	environment
A/environment/New_York/30732-3/2005	environment

A/environment/New_York/30732-4/2005	environment
A/chicken/New_York/30732-6/2005	chicken
A/turkey/New_York/30732-7/2005	turkey
A/chicken/CT/260413-2/2003	chicken
A/environment/New_York/23165-4/2005	environment
A/environment/NY/260422-10/2003	environment
A/chicken/PA/19241/1997	chicken
A/guinea_fowl/NJ/119063-8/2001	guinea_fowl
A/unknown/NY/120485-5/2001	unknown
A/Turkey/New_York/4450-5/94	Turkey
A/avian/NY/118353-1/2001	avian
A/swine/KU/16/2001	swine
A/chicken/NJ/118878-5/01	chicken
A/chicken/New_York/11678-5/2005	chicken
A/environment/NY/241365-18/2003	environment
A/chicken/NY/119256-7/01	chicken
A/chicken/New_York/19495-5/2006	chicken
A/chicken/NY/21586-8/99	chicken
A/chicken/Jilin/SD020/2014	chicken
A/chicken/NY/30749-3/00	chicken
A/chicken/New_York/21211-1/2005	chicken
A/chukar/New_York/21211-7/2005	chukar
A/chicken/PA/149092-1/02	chicken
A/environment/New_York/22067-3/2005	environment
A/chicken/New_York/22067-8/2005	chicken
A/chicken/New_York/22067-9/2005	chicken
A/Chicken/NJ/16224-6/99	Chicken
A/muscovy_duck/New_York/23165-13/2005	muscovy_duck
A/environment/New_York/30720-1/2005	environment
A/chicken/New_York/30732-9/2005	chicken
A/chicken/New_York/30732-10/2005	chicken
A/muscovy_duck/New_York/30732-13/2005	muscovy_duck
A/environment/New_York/16326-2/2005	environment
A/environment/New_York/31621-2/2005	environment
A/environment/New_York/139052/2005	environment
A/environment/New_York/143646-1/2005	environment
A/guinea_fowl/New_York/143646-3/2005	guinea_fowl
A/environment/New_York/37982-2/2005	environment
A/pheasant/New_York/143646-4/2005	pheasant
A/quail/Italy/4610/2003	quail
A/duck/New_York/143646-5/2005	duck
A/environment/New_York/3181-1/2006	environment
A/environment/New_York/3181-2/2006	environment
A/chicken/New_York/3181-5/2006	chicken
A/environment/New_York/3185-2/2006	environment
A/environment/New_York/3185-3/2006	environment
A/environment/New_York/3185-5/2006	environment

A/guinea_fowl/New_York/8391-1/2006	guinea_fowl
A/chicken/New_York/8391-2/2006	chicken
A/environment/New_York/19501-1/2006	environment
A/guinea_fowl/New_York/19501-4/2006	guinea_fowl
A/environment/New_York/29047-1/2006	environment
A/environment/New_York/29047-2/2006	environment
A/environment/New_York/29047-3/2006	environment
A/chicken/New_York/29047-4/2006	chicken
A/chicken/Hebei/1/2002	chicken
A/chicken/New_York/22067-7/2005	chicken
A/guinea_fowl/New_York/22071/2005	guinea_fowl
A/environment/New_York/23164-1/2005	environment
A/environment/New_York/23164-2/2005	environment
A/guinea_fowl/New_York/23164-3-05/2005	guinea_fowl
A/guinea_fowl/New_York/23165-12/2005	guinea_fowl
A/quail/New_York/30732-12/2005	quail
A/environment/New_York/31621-3/2005	environment
A/chicken/New_York/31621-9/2005	chicken
A/environment/New_York/37982-1/2005	environment
A/guinea_fowl/New_York/37982-3/2005	guinea_fowl
A/environment/New_York/30732-1/2005	environment
A/environment/New_York/81928-2/2005	environment
A/American_green-winged_teal/Ohio/13OS1769/2013	American_green-winged_teal
A/environment/New_York/88291-3/2005	environment
A/guinea_fowl/New_York/32084/2006	guinea_fowl
A/chicken/New_York/19499/2005	chicken
A/chicken/New_York/10196-4/2005	chicken
A/chicken/New_York/10196-5/2005	chicken
A/guinea_fowl/New_York/22067-10/2005	guinea_fowl
A/duck/New_York/22067-11/2005	duck
A/environment/New_York/23165-5/2005	environment
A/chicken/New_York/23165-10/2005	chicken
A/chicken/New_York/23165-11/2005	chicken
A/chicken/New_York/63806-11/2005	chicken
A/environment/New_York/88291-1/2005	environment
A/environment/New_York/26792-1/2005	environment
A/environment/New_York/26792-2/2005	environment
A/quail/New_York/63806-13/2005	quail
A/environment/New_York/88291-2/2005	environment
A/environment/New_York/31621-5/2005	environment
A/chicken/New_York/88291-15/2005	chicken
A/environment/New_York/88291-5/2005	environment
A/Guinea_fowl/New_York/98616-3/2005	Guinea_fowl
A/chicken/New_York/88291-6/2005	chicken
A/unknown/New_York/11646-8/2005	unknown
A/duck/New_York/88291-13/2005	duck
A/environment/New_York/26792-4/2005	environment

A/quail/New_York/89641/2005	quail
A/environment/New_York/98616-2/2005	environment
A/duck/New_York/98616-5/2005	duck
A/Guinea_fowl/New_York/101276-1/2005	Guinea_fowl
A/chicken/New_York/10508/2005	chicken
A/Muscovy_duck/New_York/11646-4/2005	Muscovy_duck
A/unknown/New_York/11646-6/2005	unknown
A/unknown/New_York/11675/2005	unknown
A/unknown/New_York/13479-3/2005	unknown
A/unknown/New_York/13479-5/2005	unknown
A/turkey/New_York/122501-2/2005	turkey
A/chicken/New_York/19495-2/2006	chicken
A/chicken/New_York/19495-3/2006	chicken
A/environment/New_York/26792-3/2005	environment
A/ruddy_turnstone/New_Jersey/563/2006	ruddy_turnstone
A/chicken/New_York/16326-4/2005	chicken
A/unknown/New_York/13479-4/2005	unknown
A/environment/New_York/19501-2/2006	environment
A/mallard/Alberta/27/2001	mallard
A/ruddy turnstone/NJ/65/1985	ruddy_turnstone
A/blue-winged teal/ALB/295/1977	blue-winged_teal
A/widgeon/ALB/284/1977	widgeon
A/green-winged teal/ALB/228/1985	green-winged_teal
A/Canada/rv504/2004	human
A/turkey/England/1963	turkey
A/American black duck/NB/2538/2007	American_black_duck
A/chicken/SK/HR-00011/2007	chicken
A/green winged teal/California/AKS1370/2008	green_winged_teal
A/northern shoveler/California/HKWF1026/2007	northern_shoveler
A/northern shoveler/California/HKWF2031/2008	northern_shoveler
A/tufted duck/PT/13771/2006	tufted_duck
A/Mexico/InDRE7218/2012	human
A/chicken/Jalisco/CPA1/2012	chicken
A/northern shoveler/California/LDC366/2015	northern_shoveler
A/mallard/California/LDC331/2015	mallard
A/chicken/Jalisco/CPA-01863-16-CENASA-95294/2016	chicken
A/mallard/Kentucky/AH0051748/2016	mallard
A/mallard/Kentucky/AH0051797/2016	mallard
A/mallard/Kentucky/AH0051806/2016	mallard
A/mallard/Kentucky/AH0051811/2016	mallard
A/northern shoveler/Texas/AH0056519/2016	northern_shoveler
A/American green-winged teal/Texas/AH0041269/2016	American_green-winged_teal
A/American green-winged teal/Texas/AH0040242/2016	American_green-winged_teal
A/American green-winged teal/Texas/AH0057336/2016	American_green-winged_teal
A/feces/Minnesota/AH0017578/2015	feces
A/ruddy turnstone/New Jersey/UGAI15-3933/2015	ruddy_turnstone
A/blue winged teal/Texas/UGAI15-4927/2015	blue_winged_teal

A/mallard/Utah/AH0066981/2016	mallard
A/American green-winged teal/Utah/AH0076018/2016	American_green-winged_teal
A/mallard/Utah/AH0076030/2016	mallard
A/American green-winged teal/Utah/AH0076032/2016	American_green-winged_teal
A/mallard/Utah/AH0075860/2016	mallard
A/mallard/Utah/AH0075862/2016	mallard
A/American green-winged teal/Utah/AH0075891/2016	American_green-winged_teal
A/mallard/Utah/AH0075906/2016	mallard
A/mallard/Utah/AH0013275/2016	mallard
A/northern shoveler/Guatemala/CIP049H107-88/2012	northern_shoveler
A/mallard duck/Netherlands/24/2014	mallard_duck
A/mallard duck/Netherlands/4/2010	mallard_duck
A/mallard duck/Georgia/3/2012	mallard_duck
A/mallard duck/Netherlands/23/2013	mallard_duck
A/mallard duck/Netherlands/5/2010	mallard_duck
A/blue winged teal/Texas/A00676566/2009	blue_winged_teal
A/American green-winged teal/Colorado/AH0026688/2015	American_green-winged_teal
A/mallard/Indiana/A00142205/2008	mallard
A/turkey/Minnesota/1138/1980	turkey
A/yellow-billed pintail/Chile/11/2014	yellow-billed_pintail
A/American green-winged teal/Mississippi/AH0076465/2016	American_green-winged_teal
A/mallard/Kansas/AH0044134/2016	mallard
A/mallard duck/Maryland/17OS0122/2017	mallard_duck
A/mallard duck/Maryland/17OS0114/2017	mallard_duck
A/Northern shoveler/Mississippi/16OS6107/2016	Northern_shoveler
A/Northern shoveler/Mississippi/16OS6067/2016	Northern_shoveler
A/lesser scaup/Illinois/17OS1577/2017	lesser_scaup
A/chicken/Jalisco/CPA-01859-16-CENASA-95294/2016	chicken
A/mallard duck/Maryland/17OS0098/2017	mallard_duck
A/chicken/Jalisco/CPA-01655-16-CENASA-95076/2016	chicken
A/chicken/Puebla/CPA-03191-16-CENASA-95076/2016	chicken
A/chicken/Puebla/CPA-03309-16-CENASA-95076/2016	chicken
A/chicken/Jalisco/CPA-01858-16-CENASA-95294/2016	chicken
A/chicken/Jalisco/CPA-04173-16-CENASA-95294/2016	chicken
A/chicken/Puebla/CPA-04451-16-CENASA-95294/2016	chicken
A/chicken/Puebla/CPA-04760-16-CENASA-95294/2016	chicken
A/chicken/Puebla/CPA-04148-16-CENASA-95294/2016	chicken
A/chicken/Puebla/CPA-02457-16-CENASA-95294/2016	chicken
A/chicken/Jalisco/CPA-01861-16-CENASA-95294/2016	chicken
A/chicken/Jalisco/CPA-01864-16-CENASA-95294/2016	chicken
A/green-winged teal/Illinois/17OS1400/2017	green-winged_teal
A/chicken/Guanajuato/CPA-02921-16-CENASA- 95294/2016	chicken
A/mallard/Utah/AH0075852/2016	mallard
A/Northern shoveler/Mississippi/16OS6165/2016	Northern_shoveler
A/mallard duck/Illinois/16OS4580/2016	mallard_duck
A/mallard/Utah/AH0020690/2015	mallard
A/ruddy turnstone/New Jersey/UGAI15-4215/2015	ruddy_turnstone

A/ruddy turnstone/New Jersey/UGAI15-4237/2015	ruddy_turnstone
A/northern shoveler/Louisiana/AH0036364/2016	northern_shoveler
A/duck/Alaska/3111/1993	duck
A/Northern shoveler/Mississippi/16OS6112/2016	Northern_shoveler
A/Northern shoveler/Mississippi/16OS6142/2016	Northern_shoveler
A/yellow-billed teal/Chile/12/2014	yellow-billed_teal
A/chicken/Chakwal/NARC-35/2001	chicken
A/chicken/Karachi/NARC-23/2003	chicken
A/chicken/Chakwal/NARC-46/2003	chicken
A/chicken/Karachi/NARC-100/2004	chicken
A/chicken/Chakwal/NARC-148/2004	chicken
A/laughing gull/Delaware/42/06	laughing_gull
A/northern pintail/Texas/A00466052/2009	northern_pintail
A/mute swan/Rhode Island/A00325136/2008	mute_swan
A/mute swan/Rhode Island/A00325129/2008	mute_swan
A/mute swan/Rhode Island/A00325125/2008	mute_swan
A/mute swan/Rhode Island/A00325117/2008	mute_swan
A/mute swan/Rhode Island/A00325115/2008	mute_swan
A/mute swan/Rhode Island/A00325112/2008	mute_swan
A/mute swan/Rhode Island/A00325108/2008	mute_swan
A/mute swan/Rhode Island/A00325105/2008	mute_swan
A/blue winged teal/Minnesota/A00137660/2009	blue_winged_teal
A/turkey/MN/24552/1982	turkey
A/chicken/Canada/314514-1/2005	chicken
A/blue-winged teal/Ohio/658/2004	blue-winged_teal
A/northern shoveler/California/27820/2007	northern_shoveler
A/northern shoveler/Washington/44249-664/2006	northern_shoveler
A/northern shoveler/Washington/44249-749/2006	northern_shoveler
A/northern shoveler/Washington/44249-752/2006	northern_shoveler
A/northern shoveler/Washington/44249-783/2006	northern_shoveler
A/American green-winged teal/California/44287-084/2007	American_green-winged_teal
A/northern shoveler/California/28327/2007	northern_shoveler
A/northern shoveler/California/44287-364/2007	northern_shoveler
A/American green-winged teal/California/44287-713/2007	American_green-winged_teal
A/environment/Indiana/08OS2857/2008	environment
A/environment/Indiana/08OS2885/2008	environment
A/northern pintail/Interior Alaska/8MP0262R2/2008	northern_pintail
A/northern shoveler/California/HKWF1372C/2007	northern_shoveler
A/mallard/Alberta/195/1989	mallard
A/ruddy turnstone/Delaware Bay/135/1996	ruddy_turnstone
A/mallard/Alberta/22/2001	mallard
A/laughing gull/Delaware Bay/42/2006	laughing_gull
A/ruddy turnstone/Delaware Bay/281/2006	ruddy_turnstone
A/ruddy turnstone/Delaware Bay/282/2006	ruddy_turnstone
A/shorebird/Delaware Bay/332/2006	shorebird
A/shorebird/Delaware Bay/513/2006	shorebird
A/shorebird/Delaware Bay/552/2006	shorebird

A/chicken/Murree/NARC-01/1995	chicken
A/environment/California/7451/2010	environment
A/American black duck/New Brunswick/02493/2007	American_black_duck
A/American black duck/New Brunswick/04388/2007	American_black_duck
A/American black duck/New Brunswick/02490/2007	American_black_duck
A/northern pintail/Interior Alaska/10BM02539R0/2010	northern_pintail
A/northern pintail/Illinois/10OS3959/2010	northern_pintail
A/northern shoveler/Wisconsin/10OS3226/2010	northern_shoveler
A/mallard/Wisconsin/10OS3171/2010	mallard
A/gadwall/Missouri/10OS4731/2010	gadwall
A/northern shoveler/Missouri/10OS4750/2010	northern_shoveler
A/American green-winged teal/Mississippi/11OS250/2011	American_green-winged_teal
A/mallard/Interior Alaska/10BM04564R1/2010	mallard
A/mallard/Minnesota/182761/1998	mallard
A/blue-winged teal/Texas/578585/2002	blue-winged_teal
A/blue-winged Teal/North Dakota/AI09-3760/2009	blue-winged_Teal
A/northern pintail/Interior Alaska/10BM07399R0/2010	northern_pintail
A/mallard/Interior Alaska/10BM07501R0/2010	mallard
A/mallard/Interior Alaska/10CH00016R0/2010	mallard
A/mallard/Interior Alaska/10BM10324R0/2010	mallard
A/mallard/Interior Alaska/10BM10859R0/2010	mallard
A/northern pintail/Interior Alaska/10BM11366R0/2010	northern_pintail
A/northern pintail/Interior Alaska/10BM11038R0/2010	northern_pintail
A/laughing gull/New York/AI00-2455/2000	laughing_gull
A/red knot/Delaware/650665/2002	red_knot
A/ruddy turnstone/Delaware/650635/2002	ruddy_turnstone
A/red knot/New Jersey/AI06-096/2006	red_knot
A/ruddy turnstone/New Jersey/AI06-179/2006	ruddy_turnstone
A/ruddy turnstone/Delaware/AI06-778/2006	ruddy_turnstone
A/northern shoveler/Illinois/11OS4404/2011	northern_shoveler
A/mallard/California/3956/2012	mallard
A/mallard/Wisconsin/143/1981	mallard
A/mallard/Wisconsin/1132/1982	mallard
A/mallard/Wisconsin/1279/1983	mallard
A/mallard/Wisconsin/223/1979	mallard
A/mallard/Wisconsin/438/1979	mallard
A/mallard/Alberta/49/1976	mallard
A/mallard/Alberta/699/1981	mallard
A/green-winged teal/Alberta/228/1985	green-winged_teal
A/mallard/Alberta/93/1996	mallard
A/pintail/Alberta/25/2001	pintail
A/mallard/Alberta/167/2010	mallard
A/mallard/Alberta/192/2010	mallard
A/ruddy turnstone/New Jersey/65/1985	ruddy_turnstone
A/shorebird/Delaware Bay/2696/1988	shorebird
A/ruddy turnstone/Delaware Bay/126/1996	ruddy_turnstone
A/shorebird/Delaware Bay/22/2006	shorebird

A/ruddy turnstone/Delaware Bay/262/2006	ruddy_turnstone
A/mallard/Minnesota/AI07-3433/2007	mallard
A/mallard/Minnesota/AI08-5469/2008	mallard
A/blue-winged teal/North Dakota/AI09-3760/2009	blue-winged_teal
A/ruddy turnstone/New Jersey/AI11-2098/2011	ruddy_turnstone
A/environment/Maryland/06OS1627/2006	environment
A/environment/Maryland/06OS1628/2006	environment
A/environment/Maryland/06OS1634/2006	environment
A/environment/Maryland/06OS1635/2006	environment
A/environment/Maryland/06OS1636/2006	environment
A/environment/Maryland/06OS1656/2006	environment
A/environment/Maryland/06OS1658/2006	environment
A/environment/Maryland/06OS268/2006	environment
A/environment/Maryland/06OS280/2006	environment
A/mute swan/Rhode Island/A00325114/2008	mute_swan
A/mallard/Oklahoma/A00744383/2009	mallard
A/mallard/Oklahoma/A00449455/2009	mallard
A/mallard/Oklahoma/A00449368/2009	mallard
A/mallard/New York/A00723392/2009	mallard
A/mallard/Nebraska/A00709657/2009	mallard
A/mallard/Michigan/A00869519/2009	mallard
A/mallard/Kansas/A00523306/2008	mallard
A/mallard/Iowa/A00558620/2008	mallard
A/mallard/Illinois/A00755320/2009	mallard
A/mallard/Illinois/A00325439/2009	mallard
A/domestic duck/West Virginia/A00140915/2008	domestic_duck
A/domestic duck/West Virginia/A00140913/2008	domestic_duck
A/domestic duck/West Virginia/A00140912/2008	domestic_duck
A/blue winged teal/Texas/A00605473/2009	blue_winged_teal
A/blue winged teal/Texas/A00463679/2010	blue_winged_teal
A/blue winged teal/Missouri/A00624484/2008	blue_winged_teal
A/blue winged teal/Missouri/A00624483/2008	blue_winged_teal
A/blue winged teal/Louisiana/A00637297/2009	blue_winged_teal
A/American green winged teal/Wyoming/A00230796/2008	American_green_winged_teal
A/American green winged teal/Utah/A00833077/2009	American_green_winged_teal
A/American green winged teal/Texas/A00604032/2009	American_green_winged_teal
A/American green winged teal/Texas/A00604029/2009	American_green_winged_teal
A/American green winged teal/Texas/A00604024/2009	American_green_winged_teal
A/American green winged teal/Texas/A00586649/2009	American_green_winged_teal
A/American black duck/Delaware/A00870108/2010	American_black_duck
A/mallard/Interior Alaska/11BM03577/2011	mallard
A/mallard/Interior Alaska/11ML00058/2011	mallard
A/mallard/Maryland/09OS1128/2009	mallard
A/mallard/Maryland/09OS1129/2009	mallard
A/mallard/Maryland/09OS1133/2009	mallard
A/mallard/Maryland/09OS1135/2009	mallard
A/mallard/Maryland/09OS1140/2009	mallard

A/mallard/Maryland/09OS1141/2009	mallard
A/mallard/Maryland/09OS1142/2009	mallard
A/mallard/Maryland/09OS1143/2009	mallard
A/mallard/Maryland/09OS1145/2009	mallard
A/mallard/Maryland/09OS1148/2009	mallard
A/mallard/Maryland/09OS1150/2009	mallard
A/mallard/Maryland/09OS1151/2009	mallard
A/mallard/Maryland/09OS1152/2009	mallard
A/mallard/Maryland/09OS1174/2009	mallard
A/mallard/Southcentral Alaska/15ML00249/2015	mallard
A/American black duck/Ohio/16OS0658/2016	American_black_duck
A/northern shoveler/Mississippi/15OS8011/2015	northern_shoveler
A/American green-winged teal/California/AH0079678/2016	American_green-winged_teal
A/mallard/Kentucky/AH00729354/2016	mallard
A/American green-winged teal/Kansas/AH0068556/2015	American_green-winged_teal
A/mallard/Minnesota/AH0065348/2015	mallard
A/northern shoveler/Louisiana/AH0036366/2016	northern_shoveler
A/American green-winged teal/Louisiana/AH0036368/2016	American_green-winged_teal
A/mallard/New York/AH0049315/2016	mallard
A/mallard/Utah/AH0075857/2016	mallard
A/mallard/Utah/AH0075937/2016	mallard
A/American green-winged teal/California/28855/2007	American_green-winged_teal
A/mallard/Missouri/220/2009	mallard
A/mallard/Wisconsin/A00465618/2008	mallard
A/mallard/South Dakota/A00649542/2008	mallard
A/cinnamon teal/Mexico/2817/2006	cinnamon_teal
A/yellow-billed pintail/Chile/10/2014	yellow-billed_pintail
A/mallard/Ohio/322/1998	mallard
A/mallard/Delaware/418/2005	mallard
A/turkey/Italy/8912/2002	turkey
A/turkey/Italy/2987/2003	turkey
A/turkey/Italy/3337/2004	turkey
A/American green-winged teal/Indiana/AH0029607/2015	American_green-winged_teal
A/American green-winged teal/Texas/AH0040565/2016	American_green-winged_teal
A/turkey/Italy/251/2003	turkey
A/turkey/Italy/3807/2004	turkey
A/turkey/Italy/1010/2003	turkey
A/turkey/Italy/2043/2003	turkey
A/chicken/Queensland/1994	chicken
A/environment/Maryland/261/2006	environment
A/environment/Maryland/267/2006	environment
A/turkey/Italy/8000/2002	turkey
A/chicken/Victoria/224/1992	chicken
A/turkey/Italy/9742/2002	turkey
A/turkey/Italy/3829/2004	turkey
A/turkey/Italy/3477/2004	turkey
A/mallard/Alaska/44300-075/2007	mallard

A/environment/Maryland/1626/2006	environment
A/chicken/Italy/4616/2003	chicken
A/mallard/Republic of Georgia/3/2010	mallard
A/northern shoveler/Oregon/A00654616/2008	northern_shoveler
A/mallard/Southcentral Alaska/15ML00267/2015	mallard
A/turkey/Italy/4479/2004	turkey
A/black duck/Maryland/415/2001	black_duck
A/turkey/Italy/2685/2003	turkey
A/avian/Delaware Bay/226/2006	avian
A/bean goose/Korea/SH20-17/2008	bean_goose
A/chicken/Italy/682/2003	chicken
A/shorebird/Delaware Bay/560/2006	shorebird
A/ruddy turnstone/Delaware Bay/121/2007	ruddy_turnstone
A/American green-winged teal/California/44242-906/2007	American_green-winged_teal
A/green-winged teal/California/11275/2008	green-winged_teal
A/green-winged teal/California/1841/2009	green-winged_teal
A/American green-winged teal/Illinois/10OS4014/2010	American_green-winged_teal
A/northern shoveler/Mississippi/11OS289/2011	northern_shoveler
A/mallard/Interior Alaska/10BM05347R0/2010	mallard
A/mallard/Interior Alaska/10BM05860R0/2010	mallard
A/northern pintail/Interior Alaska/10BM06303R0/2010	northern_pintail
A/northern pintail/Interior Alaska/10BM06306R0/2010	northern_pintail
A/northern pintail/Interior Alaska/10BM06524R0/2010	northern_pintail
A/mallard/Interior Alaska/10BM06838R0/2010	mallard
A/northern pintail/Interior Alaska/10BM06895R0/2010	northern_pintail
A/northern pintail/Interior Alaska/10BM06899R0/2010	northern_pintail
A/mallard/Interior Alaska/10BM06905R0/2010	mallard
A/mallard/Interior Alaska/10BM06909R0/2010	mallard
A/mallard/Interior Alaska/10BM06911R0/2010	mallard
A/northern pintail/Interior Alaska/10BM06720R0/2010	northern_pintail
A/northern pintail/Interior Alaska/10BM07029R0/2010	northern_pintail
A/mallard/Interior Alaska/10BM07072R0/2010	mallard
A/mallard/Interior Alaska/10BM07085R0/2010	mallard
A/mallard/Interior Alaska/10BM07092R0/2010	mallard
A/northern pintail/Interior Alaska/10BM07106R0/2010	northern_pintail
A/northern pintail/Interior Alaska/10BM07114R0/2010	northern_pintail
A/northern pintail/Interior Alaska/10BM07120R0/2010	northern_pintail
A/northern pintail/Interior Alaska/10BM07121R0/2010	northern_pintail
A/northern pintail/Interior Alaska/10BM07469R0/2010	northern_pintail
A/mallard/Interior Alaska/10BM07575R0/2010	mallard
A/mallard/Interior Alaska/10BM08100R0/2010	mallard
A/mallard/Interior Alaska/10BM08120R0/2010	mallard
A/northern pintail/Interior Alaska/10BM08400R0/2010	northern_pintail
A/northern pintail/Interior Alaska/10BM08586R0/2010	northern_pintail
A/northern pintail/Interior Alaska/10BM08705R0/2010	northern_pintail
A/mallard/Interior Alaska/10BM08874R0/2010	mallard
A/mallard/Interior Alaska/10BM08883R0/2010	mallard

A/mallard/Interior Alaska/10BM08884R0/2010	mallard
A/northern pintail/Interior Alaska/10BM09181R0/2010	northern_pintail
A/northern pintail/Interior Alaska/10BM09347R0/2010	northern_pintail
A/northern pintail/Interior Alaska/10BM09348R0/2010	northern_pintail
A/mallard/Interior Alaska/10BM09528R0/2010	mallard
A/mallard/Interior Alaska/10BM09539R0/2010	mallard
A/mallard/Interior Alaska/10BM09563R0/2010	mallard
A/northern pintail/Interior Alaska/10BM09481R0/2010	northern_pintail
A/mallard/Interior Alaska/10BM09818R0/2010	mallard
A/northern pintail/Interior Alaska/10BM10166R0/2010	northern_pintail
A/northern pintail/Interior Alaska/10BM10476R0/2010	northern_pintail
A/northern pintail/Interior Alaska/10BM10790R0/2010	northern_pintail
A/northern pintail/Interior Alaska/10BM10801R0/2010	northern_pintail
A/northern pintail/Interior Alaska/10BM10805R0/2010	northern_pintail
A/northern pintail/Interior Alaska/10BM10807R0/2010	northern_pintail
A/northern pintail/Interior Alaska/10BM10809R0/2010	northern_pintail
A/mallard/Interior Alaska/10BM10811R0/2010	mallard
A/northern pintail/Interior Alaska/10BM10853R0/2010	northern_pintail
A/mallard/Interior Alaska/10BM10861R0/2010	mallard
A/mallard/Interior Alaska/10BM10876R0/2010	mallard
A/northern pintail/Interior Alaska/10BM10906R0/2010	northern_pintail
A/northern pintail/Interior Alaska/10BM11208R0/2010	northern_pintail
A/mallard/Interior Alaska/10BM11434R0/2010	mallard
A/northern pintail/Interior Alaska/10BM11945R0/2010	northern_pintail
A/mallard/Interior Alaska/10BM12534R0/2010	mallard
A/mallard/Interior Alaska/10BM10829R0/2010	mallard
A/American green-winged teal/Ohio/12OS5538/2012	American_green-winged_teal
A/northern shoveler/Ohio/12OS5539/2012	northern_shoveler
A/northern shoveler/Utah/A00831758/2009	northern_shoveler
A/northern shoveler/Utah/A00468766/2009	northern_shoveler
A/northern shoveler/Oklahoma/A00744384/2009	northern_shoveler
A/mallard/Oklahoma/A00749161/2009	mallard
A/mallard/Montana/A00750842/2009	mallard
A/mallard/Delaware/A00456271/2009	mallard
A/American green winged teal/Utah/A00831743/2009	American_green_winged_teal
A/American green winged teal/Utah/A00614935/2009	American_green_winged_teal
A/American green winged teal/Arizona/A00115995/2009	American_green_winged_teal
A/American green winged teal/Arizona/A00115994/2009	American_green_winged_teal
A/northern shoveler/California/AH0076719/2016	northern_shoveler
A/mallard/Kentucky/AH0051787/2016	mallard
A/American green-winged teal/Texas/AH0058646/2016	American_green-winged_teal
A/mallard/Utah/AH0075849/2016	mallard
A/Northern shoveler/Mississippi/16OS6158/2016	Northern_shoveler
A/mallard duck/Ohio/16OS0869/2016	mallard_duck
A/shorebird/Delaware Bay/53/2002	shorebird
A/sanderling/New Jersey/AI06-125/2006	sanderling
A/mallard/Arkansas/15OS7607/2015	mallard

A/mallard/Interior Alaska/10BM07066R0/2010	mallard
A/northern pintail/Interior Alaska/10BM07458R0/2010	northern_pintail
A/northern pintail/Interior Alaska/10BM07737R0/2010	northern_pintail
A/mallard/Interior Alaska/10BM09548R0/2010	mallard
A/northern pintail/Interior Alaska/10BM09832R0/2010	northern_pintail
A/mallard/Interior Alaska/10BM10787R0/2010	mallard
A/northern shoveler/Illinois/12OS4951/2012	northern_shoveler
A/mallard/Maryland/14OS2911/2015	mallard
A/mallard/Maryland/12OS3628/2013	mallard
A/mallard/Maryland/12OS3629/2013	mallard
A/mallard/Maryland/12OS3630/2013	mallard
A/American green-winged teal/Utah/AH0021680/2015	American_green-winged_teal
A/duck/Korea/BC10/2007	duck
A/American green winged teal/Texas/A00604814/2009	American_green_winged_teal
A/American green winged teal/Colorado/A00660616/2008	American_green_winged_teal
A/ruddy turnstone/Delaware Bay/123/2007	ruddy_turnstone
A/ruddy turnstone/New Jersey/UGAI14-1204/2014	ruddy_turnstone
A/ruddy turnstone/New Jersey/UGAI14-1343/2014	ruddy_turnstone
A/blue-winged teal/Louisiana/UGAI14-523/2014	blue-winged_teal
A/blue-winged teal/Louisiana/UGAI14-816/2014	blue-winged_teal
A/mallard/Minnesota/UGAI15-7196/2015	mallard
A/blue-winged teal/Louisiana/UGAI15-2510/2015	blue-winged_teal
A/blue-winged teal/Louisiana/UGAI15-2471/2015	blue-winged_teal
A/blue-winged teal/Texas/UGAI15-2338/2015	blue-winged_teal
A/blue-winged teal/Texas/UGAI15-2283/2015	blue-winged_teal
A/blue-winged teal/Louisiana/UGAI15-1321/2015	blue-winged_teal
A/blue-winged teal/Texas/UGAI15-0701/2015	blue-winged_teal
A/blue-winged teal/Texas/UGAI15-0642/2015	blue-winged_teal
A/blue-winged teal/Texas/UGAI15-0568/2015	blue-winged_teal
A/environment/Maryland/1631/2006	environment
A/shorebird/Delaware Bay/555/2006	shorebird
A/turkey/Italy/8534/2002	turkey
A/American green winged teal/Colorado/A00551331/2007	American_green_winged_teal
A/mallard/Interior Alaska/6MP0984/2006	mallard
A/blue-winged teal/Ohio/13OS1770/2013	blue-winged_teal
A/American green-winged teal/Wisconsin/13OS3468/2013	American_green-winged_teal
A/blue-winged teal/Ohio/13OS1773/2013	blue-winged_teal
A/blue-winged teal/Iowa/13OS2316/2013	blue-winged_teal
A/American green-winged teal/Ohio/13OS1777/2013	American_green-winged_teal
A/turkey/Italy/9739/2002	turkey
A/environment/Maryland/276/2006	environment
A/duck/Zhejiang/12/2011	duck
A/duck/Zhejiang/11/2011	duck
A/duck/Zhejiang/10/2011	duck
A/duck/Zhejiang/2/2011	duck
A/duck/Zhejiang/DK16/2013	duck
A/duck/Zhejiang/DK10/2013	duck

A/duck/Fujian/6380/2010	duck
A/duck/Fujian/6388/2010	duck
A/duck/Jiangxi/7016/2009	duck
A/duck/Jiangxi/9742/2009	duck
A/duck/Jiangxi/13291/2009	duck
A/duck/Jiangxi/23005/2009	duck
A/duck/Jiangxi/16736/2010	duck
A/duck/Jiangxi/22330/2009	duck
A/duck/Jiangxi/16285/2010	duck
A/duck/Fujian/6326/2010	duck
A/duck/Fujian/6390/2010	duck
A/wild duck/Jiangxi/10179/2005	wild_duck
A/duck/Zhejiang/1208/2009	duck
A/chicken/BC/CN-006/2004	chicken
A/duck/Jiangxi/18644/2013	duck
A/duck/Jiangxi/20151/2013	duck
A/duck/Jiangxi/20189/2013	duck
A/duck/Jiangxi/20674/2013	duck
A/duck/Jiangxi/22676/2013	duck
A/duck/Jiangxi/22686/2013	duck
A/duck/Jiangxi/22693/2013	duck
A/duck/Jiangxi/22710/2013	duck
A/duck/Jiangxi/28843/2013	duck
A/duck/Jiangxi/29344/2013	duck
A/duck/Jiangxi/31045/2013	duck
A/duck/Jiangxi/5461/2014	duck
A/duck/Jiangxi/5465/2014	duck
A/duck/Jiangxi/23510/2013	duck
A/turkey/California/8199/2015	turkey
A/pheasant/New Jersey/26996-2/2014	pheasant
A/northern shoveler/California/AH0007019/2014	northern_shoveler
A/American green-winged teal/California/AH0011959/2015	American_green-winged_teal
A/American green-winged teal/California/AH0010595/2015	American_green-winged_teal
A/cinnamon teal/New Mexico/AH0013653/2015	cinnamon_teal
A/mallard/Louisiana/15-004158-3/2014	mallard
A/duck/New Jersey/10-001022-21/2010	duck
A/ruddy turnstone/New Jersey/UGAI15-2958/2015	ruddy_turnstone
A/Northern shoveler/Utah/AH0007941/2014	Northern_shoveler
A/American green-winged teal/AH0003731/2015	American_green-winged_teal
A/duck/Cambodia/b0116502/2017	duck
A/duck/Cambodia/b0120501/2017	duck
A/laughing gull/Delaware Bay/6/2006	laughing_gull
A/turkey/Italy/68819/03	turkey
A/wild bird/Korea/A4/11	wild_bird
A/sanderling/New_Jersey/AI06-125/2006	sanderling
A/ruddy_turnstone/New_Jersey/AI06-179/2006	ruddy_turnstone
A/environment/Maryland/261/2006	environment

A/environment/Maryland/267/2006	environment
A/blue-winged_teal/Texas/578585/2002	blue-winged_teal
A/ruddy_turnstone/Delaware/AI06-778/2006	ruddy_turnstone
A/shorebird/Delaware_Bay/53/2002	shorebird
A/mallard/Alberta/27/2001	mallard
A/mallard/Alberta/22/2001	mallard
A/duck/Jiangxi/20189/2013	duck
A/duck/Jiangxi/20151/2013	duck
A/mallard/Interior_Alaska/10BM07066R0/2010	mallard
A/mallard/Interior_Alaska/10BM07072R0/2010	mallard
A/duck/Jiangxi/20674/2013	duck
A/northern_pintail/Interior_Alaska/10BM07029R0/2010	northern_pintail
A/northern_pintail/Interior_Alaska/10BM07114R0/2010	northern_pintail
A/mallard/Interior_Alaska/10BM07092R0/2010	mallard
A/mallard/Interior_Alaska/10BM07085R0/2010	mallard
A/northern_pintail/Interior_Alaska/10BM07106R0/2010	northern_pintail
A/northern_pintail/Interior_Alaska/10BM07120R0/2010	northern_pintail
A/duck/Jiangxi/22676/2013	duck
A/northern_pintail/Interior_Alaska/10BM07469R0/2010	northern_pintail
A/northern_pintail/Interior_Alaska/10BM07121R0/2010	northern_pintail
A/northern_shoveler/Washington/44249-783/2006	northern_shoveler
A/northern_pintail/Interior_Alaska/10BM07737R0/2010	northern_pintail
A/northern_pintail/Interior_Alaska/10BM07458R0/2010	northern_pintail
A/duck/Jiangxi/22686/2013	duck
A/mallard/Interior_Alaska/10BM07501R0/2010	mallard
A/mallard/Interior_Alaska/10BM07575R0/2010	mallard
A/mallard/Interior_Alaska/10BM08100R0/2010	mallard
A/mallard/Interior_Alaska/10BM08120R0/2010	mallard
A/American_green-winged_teal/California/44287-084/2007	American_green-winged_teal
A/northern_pintail/Interior_Alaska/10BM08705R0/2010	northern_pintail
A/mallard/Interior_Alaska/10BM08874R0/2010	mallard
A/northern_pintail/Interior_Alaska/10BM08400R0/2010	northern_pintail
A/northern_pintail/Interior_Alaska/10BM08586R0/2010	northern_pintail
A/northern_pintail/Interior_Alaska/10BM09181R0/2010	northern_pintail
A/northern_pintail/Interior_Alaska/10BM09347R0/2010	northern_pintail
A/northern_shoveler/California/44287-364/2007	northern_shoveler
A/mallard/Interior_Alaska/10BM08883R0/2010	mallard
A/mallard/Interior_Alaska/10BM08884R0/2010	mallard
A/northern_shoveler/California/28327/2007	northern_shoveler
A/mallard/Interior_Alaska/10BM09539R0/2010	mallard
A/mallard/Interior_Alaska/10BM09548R0/2010	mallard
A/northern_pintail/Interior_Alaska/10BM09348R0/2010	northern_pintail
A/mallard/Interior_Alaska/10BM09528R0/2010	mallard
A/northern_pintail/Interior_Alaska/10BM09481R0/2010	northern_pintail
A/American_green-winged_teal/Ohio/12OS5538/2012	American_green-winged_teal
A/mallard/Interior_Alaska/10BM09818R0/2010	mallard
A/mallard/Interior_Alaska/10BM09563R0/2010	mallard

A/laughing_gull/Delaware/42/06	laughing_gull
A/northern_shoveler/Ohio/12OS5539/2012	northern_shoveler
A/mallard/Interior_Alaska/10CH00016R0/2010	mallard
A/mallard/Interior_Alaska/10BM10324R0/2010	mallard
A/northern_pintail/Interior_Alaska/10BM09832R0/2010	northern_pintail
A/northern_pintail/Interior_Alaska/10BM10166R0/2010	northern_pintail
A/duck/Jiangxi/22693/2013	duck
A/duck/Jiangxi/28843/2013	duck
A/northern_shoveler/California/27820/2007	northern_shoveler
A/ruddy_turnstone/Delaware_Bay/135/1996	ruddy_turnstone
A/northern_shoveler/Washington/44249-664/2006	northern_shoveler
A/environment/Maryland/276/2006	environment
A/American_green-winged_teal/California/44242-906/2007	American_green-winged_teal
A/green_winged_teal/California/AKS1370/2008	green_winged_teal
A/duck/Jiangxi/31045/2013	duck
A/northern_shoveler/Washington/44249-749/2006	northern_shoveler
A/northern_shoveler/Washington/44249-752/2006	northern_shoveler
A/duck/Jiangxi/29344/2013	duck
A/ruddy_turnstone/NJ/65/1985	ruddy_turnstone
A/duck/Jiangxi/5465/2014	duck
A/northern_shoveler/Illinois/12OS4951/2012	northern_shoveler
A/environment/Maryland/06OS1627/2006	environment
A/environment/Maryland/06OS1628/2006	environment
A/mallard/Interior_Alaska/6MP0984/2006	mallard
A/environment/Maryland/06OS1636/2006	environment
A/environment/Maryland/06OS1634/2006	environment
A/environment/Maryland/06OS1658/2006	environment
A/environment/Maryland/06OS1635/2006	environment
A/northern_pintail/Interior_Alaska/10BM02539R0/2010	northern_pintail
A/environment/Maryland/06OS268/2006	environment
A/chicken/Queensland/1994	chicken
A/environment/Maryland/06OS280/2006	environment
A/tufted_duck/PT/13771/2006	tufted_duck
A/environment/Maryland/06OS1656/2006	environment
A/mallard/Alberta/195/1989	mallard
A/widgeon/Alberta/284/1977	widgeon
A/mallard/Missouri/220/2009	mallard
A/blue-winged_teal/ALB/295/1977	blue-winged_teal
A/mallard_duck/Alberta/435/1985	mallard_duck
A/mallard/Interior_Alaska/10BM10829R0/2010	mallard
A/green-winged_teal/ALB/228/1985	green-winged_teal
A/chicken/British_Columbia/04	chicken
A/GSC_chicken_B/British_Columbia/04	GSC_chicken_B
A/GSC_chicken/British_Columbia/04	GSC_chicken
A/mallard/Wisconsin/08OS2844/2008	mallard
A/environment/Indiana/08OS2885/2008	environment
A/pheasant/Minnesota/917/1980	pheasant

A/mallard/Alberta/93/1996	mallard
A/mallard/Alberta/49/1976	mallard
A/green-winged_teal/Alberta/228/1985	green-winged_teal
A/mallard/Alberta/699/1981	mallard
A/chicken/Italy/682/2003	chicken
A/mallard/Alberta/192/2010	mallard
A/pintail/Alberta/25/2001	pintail
A/mallard/Alberta/167/2010	mallard
A/shorebird/Delaware_Bay/2696/1988	shorebird
A/ruddy_turnstone/Delaware_Bay/126/1996	ruddy_turnstone
A/ruddy_turnstone/New_Jersey/65/1985	ruddy_turnstone
A/ruddy_turnstone/Delaware_Bay/262/2006	ruddy_turnstone
A/shorebird/Delaware_Bay/22/2006	shorebird
A/mallard/Minnesota/AI08-5469/2008	mallard
A/mallard/Interior_Alaska/10BM05860R0/2010	mallard
A/blue-winged_teal/Louisiana/AI11-2911/2011	blue-winged_teal
A/northern_pintail/Interior_Alaska/10BM06303R0/2010	northern_pintail
A/American_black_duck/NB/2538/2007	American_black_duck
A/mallard/Minnesota/AI07-3433/2007	mallard
A/northern_pintail/Interior_Alaska/10BM06306R0/2010	northern_pintail
A/ruddy_turnstone/New_Jersey/AI11-2098/2011	ruddy_turnstone
A/chicken/SK/HR-00011/2007	chicken
A/blue-winged_teal/North_Dakota/AI09-3760/2009	blue-winged_teal
A/Canada/rv504/2004	human
A/laughing_gull/New_York/AI00-2455/2000	laughing_gull
A/northern_pintail/Interior_Alaska/10BM06524R0/2010	northern_pintail
A/mallard/Alberta/243/2006	mallard
A/laughing_gull/Delaware_Bay/42/2006	laughing_gull
A/mallard/Interior_Alaska/10BM05347R0/2010	mallard
A/mallard/Interior_Alaska/10BM06838R0/2010	mallard
A/mallard/Interior_Alaska/10BM06905R0/2010	mallard
A/mallard/Interior_Alaska/10BM06909R0/2010	mallard
A/northern_pintail/Interior_Alaska/10BM06895R0/2010	northern_pintail
A/northern_pintail/Interior_Alaska/10BM06899R0/2010	northern_pintail
A/northern_pintail/Interior_Alaska/10BM06720R0/2010	northern_pintail
A/mallard/Interior_Alaska/10BM06911R0/2010	mallard
A/turkey/Italy/4479/2004	turkey
A/turkey/Italy/251/2003	turkey
A/turkey/Italy/3807/2004	turkey
A/turkey/Italy/8912/2002	turkey
A/red_knot/Delaware/650665/2002	red_knot
A/northern_pintail/Illinois/10OS3959/2010	northern_pintail
A/chicken/Chakwal/NARC-35/2001	chicken
A/chicken/Karachi/NARC-23/2003	chicken
A/chicken/Chakwal/NARC-46/2003	chicken
A/ruddy_turnstone/Delaware/650635/2002	ruddy_turnstone
A/chicken/Karachi/NARC-100/2004	chicken

A/chicken/Chakwal/NARC-148/2004	chicken
A/chicken/Murree/NARC-01/1995	chicken
A/northern_pintail/Interior_Alaska/8MP0262R2/2008	northern_pintail
A/mallard/Republic_of_Georgia/3/2010	mallard
A/American_green-winged_teal/Illinois/10OS4014/2010	American_green-winged_teal
A/American_green-winged_teal/Ohio/13OS1777/2013	American_green-winged_teal
A/American_black_duck/New_Brunswick/04388/2007	American_black_duck
A/black_duck/Ohio/415/2001	black_duck
A/American_black_duck/New_Brunswick/02490/2007	American_black_duck
A/northern_shoveler/Wisconsin/10OS3226/2010	northern_shoveler
A/chicken/Canada/314514-1/2005	chicken
A/mallard/Interior_Alaska/10BM10787R0/2010	mallard
A/northern_pintail/Interior_Alaska/10BM10790R0/2010	northern_pintail
A/northern_pintail/Interior_Alaska/10BM10476R0/2010	northern_pintail
A/Mexico/InDRE7218/2012	human
A/northern_pintail/Interior_Alaska/10BM10809R0/2010	northern_pintail
A/northern_pintail/Interior_Alaska/10BM10801R0/2010	northern_pintail
A/northern_pintail/Interior_Alaska/10BM10805R0/2010	northern_pintail
A/mallard/Interior_Alaska/10BM04564R1/2010	mallard
A/northern_pintail/Interior_Alaska/10BM10807R0/2010	northern_pintail
A/northern_shoveler/Illinois/11OS4404/2011	northern_shoveler
A/northern_pintail/Interior_Alaska/10BM10853R0/2010	northern_pintail
A/mallard/Interior_Alaska/10BM10876R0/2010	mallard
A/turkey/California/8199/2015	turkey
A/blue-winged_teal/Ohio/13OS1773/2013	blue-winged_teal
A/duck/Jiangxi/18644/2013	duck
A/mallard/Interior_Alaska/10BM10811R0/2010	mallard
A/American_green-winged_teal/Wisconsin/13OS3468/2013	American_green-winged_teal
A/mallard/Interior_Alaska/10BM10859R0/2010	mallard
A/northern_pintail/Interior_Alaska/10BM11366R0/2010	northern_pintail
A/northern_pintail/Interior_Alaska/10BM11208R0/2010	northern_pintail
A/turkey/Italy/8000/2002	turkey
A/mallard/Interior_Alaska/10BM10861R0/2010	mallard
A/northern_pintail/Interior_Alaska/10BM10906R0/2010	northern_pintail
A/northern_pintail/Interior_Alaska/10BM11038R0/2010	northern_pintail
A/mallard/Interior_Alaska/10BM12534R0/2010	mallard
A/northern_pintail/Interior_Alaska/10BM11945R0/2010	northern_pintail
A/northern_shoveler/California/HKWF1026/2007	northern_shoveler
A/northern_shoveler/California/HKWF2031/2008	northern_shoveler
A/mallard/Interior_Alaska/10BM11434R0/2010	mallard
A/mallard/Wisconsin/10OS3171/2010	mallard
A/gadwall/Missouri/10OS4731/2010	gadwall
A/northern_shoveler/Missouri/10OS4750/2010	northern_shoveler
A/turkey/Italy/1010/2003	turkey
A/Chicken/NY/14714-2/1999	Chicken
A/American_green-winged_teal/California/28855/2007	American_green-winged_teal
A/American_green-winged_teal/California/44287-713/2007	American_green-winged_teal

A/Duck/PA/143585/01	Duck
A/turkey/Italy/2987/2003	turkey
A/turkey/Italy/3337/2004	turkey
A/chicken/Victoria/224/1992	chicken
A/American_green-winged_teal/Mississippi/11OS250/2011	American_green-winged_teal
A/mallard/Delaware/418/2005	mallard
A/turkey/MN/24552/1982	turkey
A/turkey/Italy/9742/2002	turkey
A/turkey/Italy/2685/2003	turkey
A/northern_shoveler/Mississippi/11OS289/2011	northern_shoveler
A/turkey/Italy/3829/2004	turkey
A/turkey/Italy/3477/2004	turkey
A/duck/Jiangxi/22330/2009	duck
A/duck/Jiangxi/16285/2010	duck
A/duck/Fujian/6326/2010	duck
A/duck/Fujian/6390/2010	duck
A/blue-winged_Teal/North_Dakota/AI09-3760/2009	blue-winged_Teal
A/duck/Zhejiang/10/2011	duck
A/duck/Zhejiang/2/2011	duck
A/duck/Zhejiang/12/2011	duck
A/duck/Zhejiang/11/2011	duck
A/wild_duck/Jiangxi/10179/2005	wild_duck
A/blue-winged_teal/Ohio/13OS1770/2013	blue-winged_teal
A/chicken/BC/CN-006/2004	chicken
A/shorebird/Delaware_Bay/560/2006	shorebird
A/ruddy_turnstone/Delaware_Bay/121/2007	ruddy_turnstone
A/ruddy_turnstone/Delaware_Bay/123/2007	ruddy_turnstone
A/duck/Fujian/6388/2010	duck
A/duck/Fujian/6380/2010	duck
A/environment/Maryland/1626/2006	environment
A/ruddy_turnstone/Delaware_Bay/124/2007	ruddy_turnstone
A/duck/Jiangxi/9742/2009	duck
A/laughing_gull/Delaware_Bay/6/2006	laughing_gull
A/duck/Jiangxi/23005/2009	duck
A/ruddy_turnstone/Delaware_Bay/283/2006	ruddy_turnstone
A/duck/Jiangxi/16736/2010	duck
A/blue-winged_teal/Ohio/658/2004	blue-winged_teal
A/green-winged_teal/California/1841/2009	green-winged_teal
A/shorebird/Delaware_Bay/513/2006	shorebird
A/shorebird/Delaware_Bay/552/2006	shorebird
A/shorebird/Delaware_Bay/332/2006	shorebird
A/ruddy_turnstone/Delaware_Bay/281/2006	ruddy_turnstone
A/ruddy_turnstone/Delaware_Bay/282/2006	ruddy_turnstone
A/mallard/Minnesota/182761/1998	mallard
A/blue-winged_teal/Iowa/13OS2316/2013	blue-winged_teal
A/green-winged_teal/California/11275/2008	green-winged_teal
A/environment/California/7451/2010	environment

A/turkey/Italy/2043/2003	turkey
A/red_knot/New_Jersey/AI06-096/2006	red_knot
A/cinnamon_teal/Mexico/2817/2006	cinnamon_teal
A/northern_shoveler/California/HKWF1372C/2007	northern_shoveler
A/northern shoveler/California/LDC351/2015	northern_shoveler
A/Eurasian wigeon/Netherlands/2/2008	Eurasian_wigeon
A/mallard/Utah/AH0020452/2015	mallard
A/long-tailed duck/Wisconsin/16OS5291/2016	long-tailed_duck
A/long-tailed duck/Wisconsin/16OS4707/2016	long-tailed_duck
A/chicken/Jiangsu/1/2018	chicken
A/Jiangsu/1/2018	human
A/mallard/Nova Scotia/02286/2007	mallard
A/mallard/Missouri/10MO053/2010	mallard
A/mallard/Wisconsin/1169/1983	mallard
A/mallard/Wisconsin/1171/1983	mallard
A/mallard/Wisconsin/1177/1983	mallard
A/mallard/Wisconsin/1111/1982	mallard
A/mallard/Wisconsin/1168/1983	mallard
A/mallard/Wisconsin/1319/1983	mallard
A/mallard/Wisconsin/1360/1983	mallard
A/mallard/Wisconsin/1391/1983	mallard
A/mallard/New York/A00723400/2009	mallard
A/emu/New South Wales/775/1997	emu
A/mallard/Maryland/06OS101/2006	mallard
A/chicken/New South Wales/327/1997	chicken
A/chicken/New South Wales/2/1997	chicken
A/northern pintail/Alaska/471/2014	northern_pintail
A/northern pintail/Alaska/619/2014	northern_pintail
A/duck/Thailand/CU-9754C/2010	duck
A/duck/Thailand/CU-9744C /2010	duck
A/environment/Korea/W266/2007	environment
A/environment/Korea/W267/2007	environment
A/mallard/Idaho/AH0008589/2015	mallard
A/duck/Thailand/CU-10507T/2011	duck
A/duck/Thailand/CU-10510C/2011	duck
A/emu/New_South_Wales/775/1997	emu
A/mallard/Missouri/10MO053/2010	mallard
A/mallard/Nova_Scotia/02286/2007	mallard
A/chicken/New_South_Wales/2/1997	chicken
A/chicken/New_South_Wales/327/1997	chicken
A/unknown/Chelyabinsk/206/2023	unknown
CTTATTTAATGATAAA/mallard duck/ALB/224/1977	mallard_duck
A/mallard duck/Netherlands/8/2014	mallard_duck
A/shorebird/Delaware Bay/274/1994	shorebird
A/mallard/California/1390/2010	mallard
A/mallard/California/1390/2010	mallard
A/mallard_duck/ALB/224/1977	mallard_duck

A/shorebird/Delaware_Bay/274/1994	shorebird
A/yellow-billed teal/Chile/8/2013	yellow-billed_teal
A/scoter/Maryland/16OS2531/2016	scoter
A/yellow-billed teal/Chile/9/2013	yellow-billed_teal
A/duck/Thailand/CU-LM7285C/2010	duck
A/Northern shoveler/NC/6412-052/2005	Northern_shoveler
A/American green-winged teal/California/28228/2007	American_green-winged_teal
A/northern shoveler/California/27985/2007	northern_shoveler
A/northern shoveler/California/44287-179/2007	northern_shoveler
A/American green-winged teal/California/44287-305/2007	American_green-winged_teal
A/black scoter/New Brunswick/00010/2009	black_scoter
A/black scoter/New Brunswick/00014/2009	black_scoter
A/black scoter/New Brunswick/00003/2009	black_scoter
A/common goldeneye/Wisconsin/10OS4202/2010	common_goldeneye
A/northern pintail/Interior Alaska/10BM09015R0/2010	northern_pintail
A/American green winged teal/Mississippi/A00630203/2009	American_green_winged_teal
A/American green-winged teal/Illinois/15OS6133/2015	American_green-winged_teal
A/duck/Thailand/CU-LM7288C/2010	duck
A/duck/Thailand/CU-LM7279T/2010	duck
A/northern shoveler/Nevada/A00505416/2008	northern_shoveler
A/duck/Thailand/CU-LM7283C/2010	duck
A/duck/Thailand/CU-LM7280C/2010	duck
A/duck/Thailand/CU-LM7284C/2010	duck
A/duck/Thailand/CU-LM7288T/2010	duck
A/wild duck/Korea/CSM27-12/2009	wild_duck
A/duck/Jiangxi/25134/2009	duck
A/duck/Jiangxi/25186/2009	duck
A/duck/Yunnan/87/2007	duck
A/wild waterfowl/Hong Kong/MPM3375/2011	wild_waterfowl
A/duck/Jiangxi/5453/2014	duck
A/chicken/Jiangxi/14501/2014	chicken
A/chicken/Jiangxi/14535/2014	chicken
A/chicken/Zhejiang/233/2016	chicken
A/duck/Korea/A117/10	duck
A/duck/Thailand/CU-LM7283T/2010	duck
A/black_scoter/New_Brunswick/00003/2009	black_scoter
A/American_green-winged_teal/California/28228/2007	American_green-winged_teal
A/wild_waterfowl/Hong_Kong/MPM3375/2011	wild_waterfowl
A/common_goldeneye/Wisconsin/10OS4202/2010	common_goldeneye
A/chicken/Jiangxi/14535/2014	chicken
A/black_scoter/New_Brunswick/00010/2009	black_scoter
A/northern_pintail/Interior_Alaska/10BM09015R0/2010	northern_pintail
A/black_scoter/New_Brunswick/00014/2009	black_scoter
A/northern_shoveler/California/44287-179/2007	northern_shoveler
A/northern_shoveler/California/27985/2007	northern_shoveler
A/American_green-winged_teal/California/44287-305/2007	American_green-winged_teal
A/duck/Jiangxi/5453/2014	duck

A/Northern_shoveler/NC/6412-052/2005	Northern_shoveler
A/duck/Jiangxi/25134/2009	duck
A/duck/Jiangxi/25186/2009	duck
A/chicken/Jiangxi/14501/2014	chicken
A/goose/Leipzig/187-7/1979	goose
A/mallard/Korea/GH170/2007	mallard
A/magpie/Korea/YJD174/2007	magpie
A/mallard/Korea/GH171/2007	mallard
A/chicken/Japan/1925	chicken
A/turkey/England/647/1977	turkey
A/duck/Pennsylvania/15-030188-1/2015	duck
A/mallard/Idaho/AH0011522/2015	mallard
A/mallard/Pennsylvania/AH0038929/2015	mallard
A/mallard/Sweden/102/2002	mallard
A/duck/Taiwan/4201/99	duck
A/equine/Prague/2/1956	equine
A/FPV/Dutch/1927	chicken
A/mallard/Missouri/10MO0551/2010	mallard
A/duck/Wisconsin/3034/1986	duck
A/mallard/Sweden/1985/2003	mallard
A/mallard/Sweden/1997/2003	mallard
A/mallard/Sweden/2001/2003	mallard
A/mallard/Sweden/2003/2003	mallard
A/mallard/Sweden/3329/2003	mallard
A/mallard/Sweden/3331/2003	mallard
A/mallard/Sweden/7206/2004	mallard
A/mallard/Sweden/124987/2010	mallard
A/mallard/Sweden/885/2002	mallard
A/mallard/Sweden/1337/2002	mallard
A/mallard/Sweden/1645/2002	mallard
A/mallard/Sweden/1689/2002	mallard
A/mallard/Sweden/1690/2002	mallard
A/mallard/Sweden/1978/2003	mallard
A/mallard/Sweden/1983/2003	mallard
A/mallard/Sweden/2027/2003	mallard
A/mallard/Sweden/2051/2003	mallard
A/mallard/Sweden/3269/2003	mallard
A/mallard/Sweden/1628/2002	mallard
A/mallard/Sweden/1671/2002	mallard
A/mallard/Sweden/1678/2002	mallard
A/mallard/Sweden/1681/2002	mallard
A/mallard/Sweden/1682/2002	mallard
A/mallard/Sweden/1962/2003	mallard
A/mallard/Sweden/1965/2003	mallard
A/mallard/Sweden/1967/2003	mallard
A/mallard/Sweden/1970/2003	mallard
A/mallard/Sweden/1972/2003	mallard

A/mallard/Sweden/1975/2003	mallard
A/mallard/Sweden/1981/2003	mallard
A/mallard/Sweden/1448/2002	mallard
A/shorebird/Delaware	shorebird
A/mallard/Minnesota/19033/1999	mallard
A/gadwall/Arizona/A00663934/2009	gadwall
A/mallard/Kentucky/AH0069753/2016	mallard
A/mallard/Sweden/S90597/2005	mallard
A/duck/Hokkaido/Vac-2/04	duck
A/starling/Victoria/1985	starling
A/chicken/Victoria/1976	chicken
A/chicken/Netherlands/2586/2003	chicken
A/equine/Lexington/1/1966	equine
A/swan/Slovenia/53/2009	swan
A/mallard/Korea/GG3/2007	mallard
A/mallard/Korea/NHG187/2008	mallard
A/Italy/3/2013	human
A/mallard/Nizhnegorsk/3-06-10/2010	mallard
A/mallard/Novomyhalivka/10-23-12/2010	mallard
A/mallard/Askania-Nova/39-15-02/2011	mallard
A/duck/Victoria/1976	duck
A/mallard/Sweden/95/2005	mallard
A/mallard/Korea/GG2/2007	mallard
A/mallard/Sweden/5994/2005	mallard
A/equine/Uruguay/1063/1976	equine
A/chicken/Wenzhou/334b/2013	chicken
A/equine/Argentina/1/1977	equine
A/equine/Kentucky/1a/1975	equine
A/mallard/Sweden/5944/2005	mallard
A/goose/Guangdong/7472/2012	goose
A/duck/Fujian/5408/2008	duck
A/duck/Fujian/5476/2008	duck
A/duck/Fujian/5728/2008	duck
A/duck/Jiangxi/21669/2009	duck
A/duck/Jiangxi/23008/2009	duck
A/chicken/Jiangxi/2369/2010	chicken
A/duck/Jiangxi/16309/2010	duck
A/duck/Jiangxi/16326/2010	duck
A/duck/Jiangxi/16769/2010	duck
A/duck/Jiangxi/21980/2010	duck
A/duck/Fujian/5448/2008	duck
A/duck/Jiangxi/1717/2003	duck
A/duck/Jiangxi/1775/2003	duck
A/chicken/Wenzhou/89/2013	chicken
A/chicken/Wenzhou/189/2013	chicken
A/pigeon/Wenzhou/397/2013	pigeon
A/chicken/Wenzhou/610/2013	chicken

A/chicken/Wenzhou/626/2013	chicken
A/chicken/Wenzhou/269/2013	chicken
A/chicken/Wenzhou/275/2013	chicken
A/chicken/Wenzhou/279/2013	chicken
A/chicken/Wenzhou/305/2013	chicken
A/chicken/Wenzhou/323/2013	chicken
A/chicken/Wenzhou/331/2013	chicken
A/pigeon/Wenzhou/497/2013	pigeon
A/chicken/Wenzhou/602/2013	chicken
A/chicken/Wenzhou/624/2013	chicken
A/chicken/Wenzhou/634/2013	chicken
A/chicken/Wenzhou/640/2013	chicken
A/chicken/Wenzhou/645/2013	chicken
A/chicken/Wenzhou/658/2013	chicken
A/chicken/Wenzhou/662/2013	chicken
A/chicken/Wenzhou/666/2013	chicken
A/chicken/Wenzhou/678/2013	chicken
A/chicken/Wenzhou/685/2013	chicken
A/chicken/Wenzhou/690/2013	chicken
A/chicken/Wenzhou/698/2013	chicken
A/chicken/Wenzhou/278/2013	chicken
A/chicken/Wenzhou/299/2013	chicken
A/chicken/Delaware/10851/2014	chicken
A/chicken/Jiangxi/C25/2014	chicken
A/duck/Huzhou/4227/2013	duck
A/duck/Huzhou/4268/2013	duck
A/chicken/Jiangxi/10784/2014	chicken
A/duck/Hunan/S1161/2010	duck
A/duck/Jiangxi/S4770/2013	duck
A/duck/Zhejiang/S3158/2012	duck
A/duck/Zhejiang/S4235/2011	duck
A/environment/Fujian/S3307/2012	environment
A/environment/Zhejiang/S4051/2013	environment
A/environment/Korea/W109/2006	environment
A/environment/Korea/W133/2006	environment
A/environment/Korea/W136/2006	environment
A/environment/Korea/W143/2006	environment
A/environment/Korea/W148/2006	environment
A/environment/Korea/W152/2006	environment
A/environment/Korea/W156/2006	environment
A/environment/Korea/W169/2007	environment
A/environment/Korea/W178/2007	environment
A/environment/Korea/W437/2012	environment
A/environment/Korea/W478/2014	environment
A/duck/Korea/A75/2010	duck
A/pigeon/Wenzhou/559/2013	pigeon
A/duck/Wenzhou/47/2013	duck

A/FPV/Dutch/1927	chicken
A/American_black_duck/New_Brunswick/00344/2010	American_black_duck
A/mallard/Sweden/1645/2002	mallard
A/chicken/Wenzhou/89/2013	chicken
A/mallard/Nova_Scotia/00372/2010	mallard
A/mallard/Sweden/1690/2002	mallard
A/chicken/Wenzhou/626/2013	chicken
A/mallard/Sweden/1978/2003	mallard
A/chicken/Germany/AR915-L01268/2015	chicken
A/chicken/Wenzhou/189/2013	chicken
A/mallard/Sweden/1337/2002	mallard
A/mallard/Sweden/1689/2002	mallard
A/green-winged_teal/New_Brunswick/00392/2010	green-winged_teal
A/mallard/Sweden/2051/2003	mallard
A/chicken/Wenzhou/305/2013	chicken
A/mallard/Republic_of_Georgia/2/2010	mallard
A/mallard/Sweden/3269/2003	mallard
A/chicken/Wenzhou/323/2013	chicken
A/chicken/Germany/AR942-L01269/2015	chicken
A/chicken/Wenzhou/275/2013	chicken
A/mallard/Sweden/1983/2003	mallard
A/chicken/Wenzhou/279/2013	chicken
A/American_green-winged_teal/Illinois/10OS3329/2010	American_green-winged_teal
A/mallard/Sweden/2027/2003	mallard
A/American_green-winged_teal/Illinois/10OS3368/2010	American_green-winged_teal
A/mallard/Sweden/5944/2005	mallard
A/chicken/Wenzhou/331/2013	chicken
A/chicken/Wenzhou/658/2013	chicken
A/chicken/Wenzhou/640/2013	chicken
A/chicken/Wenzhou/645/2013	chicken
A/chicken/Wenzhou/685/2013	chicken
A/turkey/England/647/1977	turkey
A/mallard/Sweden/885/2002	mallard
A/chicken/Wenzhou/666/2013	chicken
A/chicken/Wenzhou/678/2013	chicken
A/pigeon/Wenzhou/397/2013	pigeon
A/chicken/Wenzhou/698/2013	chicken
A/chicken/Wenzhou/278/2013	chicken
A/pigeon/Wenzhou/497/2013	pigeon
A/silkie_chicken/Wenzhou/792/2013	silkie_chicken
A/silkie_chicken/Wenzhou/816/2013	silkie_chicken
A/northern_shoveler/California/44287-164/2007	northern_shoveler
A/Italy/3/2013	human
A/duck/Hokkaido/Vac-2/04	duck
A/northern_shoveler/Missouri/10OS4632/2010	northern_shoveler
A/mallard/Netherlands/9/2005	mallard
A/mallard/Sweden/95/2005	mallard

A/equine/Detroit/3/1964	equine
A/mallard/Sweden/1681/2002	mallard
A/mallard/Sweden/1682/2002	mallard
A/mallard/Sweden/1628/2002	mallard
A/mallard/Sweden/1678/2002	mallard
A/mallard/Sweden/1967/2003	mallard
A/mallard/Sweden/1970/2003	mallard
A/mallard/Sweden/1962/2003	mallard
A/starling/Victoria/1985	starling
A/mallard/Sweden/1965/2003	mallard
A/mallard/Sweden/1981/2003	mallard
A/mallard/Sweden/1972/2003	mallard
A/mallard/Sweden/1975/2003	mallard
A/chicken/Victoria/1/1985	chicken
A/northern_shoveler/Mississippi/12OS347/2012	northern_shoveler
A/American_green-winged_teal/Mississippi/11OS255/2011	American_green-winged_teal
A/teal/Egypt/00835-NAMRU3/2004	teal
A/wild_waterfowl/Hong_Kong/MPM2121/2011	wild_waterfowl
A/mallard/Sweden/2003/2003	mallard
A/American_green-winged_teal/Mississippi/12OS397/2012	American_green-winged_teal
A/SC35M	SC35M
A/wild_waterfowl/Hong_Kong/MPL1006/2011	wild_waterfowl
A/mallard/Sweden/1985/2003	mallard
A/mallard/Sweden/2001/2003	mallard
A/northern_shoveler/Mississippi/12OS362/2012	northern_shoveler
A/northern_shoveler/Mississippi/11OS202/2011	northern_shoveler
A/Egyptian_goose/Egypt/05588-NAMRU3/2006	Egyptian_goose
A/mallard/Sweden/3329/2003	mallard
A/mallard/Sweden/3331/2003	mallard
A/mallard/Sweden/5994/2005	mallard
A/mallard/Sweden/7206/2004	mallard
A/mallard/Sweden/1671/2002	mallard
A/mallard/Sweden/124987/2010	mallard
A/chicken/NL-Barneveld/15004745-006-010/2015	chicken
A/Chicken/NL-Barneveld/15004745-001-005/2015	Chicken
A/duck/Fujian/5448/2008	duck
A/wild_goose/Dongting/PC0360/2012	wild_goose
A/duck/Huzhou/4227/2013	duck
A/chicken/Jiangxi/10784/2014	chicken
A/ruddy_turnstone/DE/2378/1988	ruddy_turnstone
A/duck/Huzhou/4268/2013	duck
A/red_knot/NJ/325/1989	red_knot
A/chicken/Netherlands/2586/2003	chicken
A/northern_shoveler/Mississippi/09OS643/2009	northern_shoveler
A/chicken/Wenzhou/334b/2013	chicken
A/common_teal/Hong_Kong/MPM1740/2011	common_teal
A/wild_waterfowl/Hong_Kong/MPL705/2011	wild_waterfowl

A/common_teal/Hong_Kong/MPM1670/2011	common_teal
A/common_teal/Hong_Kong/MPL634/2011	common_teal
A/mallard/Sweden/1997/2003	mallard
A/chicken/Germany/AR1386-L01302/2015	chicken
A/chicken/Germany/AR1385-L01301/2015	chicken
A/duck/Fujian/5408/2008	duck
A/duck/Fujian/5476/2008	duck
A/goose/Guangdong/7472/2012	goose
A/duck/Fujian/5728/2008	duck
A/chicken/Netherlands/1/03	chicken
A/Netherlands/219/03	human
A/duck/Jiangxi/23008/2009	duck
A/chicken/Brescia/1902	chicken
A/duck/Jiangxi/16326/2010	duck
A/chicken/Jiangxi/2369/2010	chicken
A/ostrich/South_Africa/KRB/2013	ostrich
A/equine/Kentucky/1a/1975	equine
A/mallard/Missouri/10MO0551/2010	mallard
A/duck/Jiangxi/16769/2010	duck
A/equine/Sao_Paulo/4/1976	equine
A/duck/Jiangxi/21980/2010	duck
A/red_knot/Delaware_Bay/242/1994	red_knot
A/equine/Uruguay/1063/1976	equine
A/equine/Argentina/1/1977	equine
A/ruddy_turnstone/New_Jersey/AI11-1678/2011	ruddy_turnstone
A/mallard/Minnesota/19033/1999	mallard
A/chicken/Victoria/1976	chicken
A/chicken/Italy/13VIR4541-9/2013	chicken
A/ruddy_turnstone/Delaware/752/2006	ruddy_turnstone
A/equine/Lexington/1/1966	equine
A/chicken/Italy/13VIR4774/2013	chicken
A/chicken/Italy/13VIR5091/2013	chicken
A/turkey/Indiana/16-001571-6/2016	turkey
A/turkey/Indiana/16-001574-9/2016	turkey
A/pintail/Alberta/121/1979	pintail
A/turkey/Indiana/16-001574-8/2016	turkey
A/shelduck/South Africa/DLH/2012	shelduck
A/mallard/New Jersey/A00122457/2008	mallard
A/northern shoveler/Washington/44249-690/2006	northern_shoveler
A/mallard/Ohio/11OS2033/2011	mallard
A/mallard/Ohio/11OS2010/2011	mallard
A/American black duck/Wisconsin/10OS3949/2010	American_black_duck
A/mallard/Alberta/590/2010	mallard
A/mallard/Ohio/421/1987	mallard
A/lesser scaup/Kentucky/AH0012935/2015	lesser_scaup
A/turkey/Indiana/16-001403-2/2016	turkey
A/turkey/Indiana/16-001571-1/2016	turkey

A/turkey/Indiana/16-001571-8/2016	turkey
A/turkey/Indiana/16-001573-3/2016	turkey
A/turkey/Indiana/16-001574-2/2016	turkey
A/turkey/Indiana/16-001403-1/2016	turkey
A/turkey/Indiana/16-001574-7/2016	turkey
A/turkey/Indiana/16-001573-2/2016	turkey
A/duck/Korea/GJ56/2007	duck
A/wild duck/Jiangxi/19615/2009	wild_duck
A/duck/Jiangxi/9631/2009	duck
A/wild duck/Jiangxi/9157/2005	wild_duck
A/northern shoveler/Nevada/AH0009328/2015	northern_shoveler
A/American_black_duck/Wisconsin/10OS3949/2010	American_black_duck
A/wild_duck/Jiangxi/19615/2009	wild_duck
A/mallard/Ohio/11OS2033/2011	mallard
A/mallard/Ohio/421/1987	mallard
A/mallard/Ohio/11OS2010/2011	mallard
A/wild_duck/Jiangxi/9157/2005	wild_duck
A/duck/Jiangxi/9631/2009	duck
A/mallard/Alberta/590/2010	mallard
A/shellduck/South_Africa/DLH/2012	shellduck
A/mallard/Netherlands/33/2006	mallard
A/turkey/Minnesota/1/1988	turkey
A/Zhejiang/DTID-ZJU01/2013	human
A/Anhui/DEWH72-01/2013	human
A/Anhui/DEWH72-02/2013	human
A/Anhui/DEWH72-03/2013	human
A/Anhui/DEWH72-04/2013	human
A/Anhui/DEWH72-05/2013	human
A/Anhui/DEWH72-07/2013	human
A/Anhui/DEWH72-08/2013	human
A/Anhui/DEWH72-09/2013	human
A/Anhui/1-JCVI1_RG1/2013	human
A/Anhui/1-JCVI1_RG2/2013	human
A/Anhui/1-JCVI1_RG3/2013	human
A/Anhui/1-BALF_RG6/2013	human
A/Anhui/1-BALF_RG7/2013	human
A/Anhui/1-BALF_RG8/2013	human
A/Anhui/1-BALF_RG9/2013	human
A/Anhui/1-BALF_RG17/2013	human
A/Anhui/1-BALF_RG18/2013	human
A/Anhui/1-BALF_RG19/2013	human
A/Anhui/1-BALF_RG20/2013	human
A/Anhui/1-BALF_RG21/2013	human
A/Anhui/1-BALF_RG29/2013	human
A/Anhui/1-BALF_RG31/2013	human
A/Anhui/1-BALF_RG32/2013	human
A/Anhui/1-BALF_RG40/2013	human

A/Anhui/1-BALF_RG41/2013	human
A/Anhui/1-BALF_RG42/2013	human
A/Anhui/1-BALF_RG43/2013	human
A/Anhui/1-BALF_RG44/2013	human
A/Anhui/1-BALF_RG45/2013	human
A/Anhui/1-BALF_RG1/2013	human
A/Anhui/1-BALF_RG2/2013	human
A/Anhui/1-BALF_RG3/2013	human
A/Anhui/1-BALF_RG4/2013	human
A/Anhui/1-BALF_RG5/2013	human
A/pigeon/Wuxi/0405007G/2013	pigeon
A/duck/Sunan/040802G/2013	duck
A/chicken/Hangzhou/174/2013	chicken
A/goose/Jiangsu/1027/2013	goose
A/chicken/Wuxi/0405005/2013	chicken
A/duck/Wuxi/0405006/2013	duck
A/mallard/Alberta/177/2004	mallard
A/Qingyuan/GIRD01/2017	human
A/Guangdong/HP001/2017	human
A/Guangdong/SP440/2017	human
A/chicken/Guangdong/J1/2017	chicken
A/chicken/Guangdong/J2/2017	chicken
A/Guangdong/GZ8H002/2017	human
A/Kunming/KMCDC-YHY/2017	Kunming
A/Anhui/DEWH72-06/2013	human
A/Anhui/1-DEWH730/2013	human
A/Minhang/S01/2013	human
A/cinnamon teal/California/AH0079011/2016	cinnamon_teal
A/Changsha/2/2013	human
A/Changsha/1/2013	human
A/ruddy turnstone/Delaware Bay/220/1995	ruddy_turnstone
A/northern shoverl/Mississippi/11OS145/2011	northern_shoverl
A/mallard/Minnesota/AI09-3770/2009	mallard
A/Anhui/1-YK_RG01/2013	human
A/Anhui/1-YK_RG02/2013	human
A/Anhui/1-YK_RG03/2013	human
A/Anhui/1-YK_RG04/2013	human
A/Anhui/1-YK_RG05/2013	human
A/Anhui/1-YK_RG06/2013	human
A/Anhui/1-YK_RG07/2013	human
A/Anhui/1-YK_RG08/2013	human
A/Anhui/1-YK_RG09/2013	human
A/Anhui/1-YK_RG10/2013	human
A/Anhui/1-YK_RG12/2013	human
A/Anhui/1-YK_RG13/2013	human
A/Anhui/1-YK_RG14/2013	human
A/Anhui/1-YK_RG15/2013	human

A/Anhui/1-YK_RG16/2013	human
A/Anhui/1-YK_RG17/2013	human
A/Anhui/1-YK_RG18/2013	human
A/Anhui/1-YK_RG19/2013	human
A/Anhui/1-YK_RG20/2013	human
A/Anhui/1-YK_RG21/2013	human
A/Anhui/1-YK_RG22/2013	human
A/Anhui/1-YK_RG23/2013	human
A/Anhui/1-YK_RG24/2013	human
A/Anhui/1-YK_RG25/2013	human
A/Anhui/1-YK_RG26/2013	human
A/Anhui/1-YK_RG27/2013	human
A/Anhui/1-YK_RG28/2013	human
A/Anhui/1-YK_RG29/2013	human
A/Anhui/1-YK_RG30/2013	human
A/Anhui/1-YK_RG31/2013	human
A/Anhui/1-YK_RG32/2013	human
A/Anhui/1-YK_RG33/2013	human
A/Anhui/1-YK_RG34/2013	human
A/Anhui/1-YK_RG35/2013	human
A/Anhui/1-YK_RG36/2013	human
A/Anhui/1-YK_RG37/2013	human
A/Anhui/1-YK_RG38/2013	human
A/Anhui/1-YK_RG39/2013	human
A/Anhui/1-YK_RG40/2013	human
A/Anhui/1-YK_RG41/2013	human
A/Anhui/1-YK_RG42/2013	human
A/Anhui/1-YK_RG43/2013	human
A/Anhui/1-YK_RG44/2013	human
A/Anhui/1-YK_RG45/2013	human
A/Anhui/1-YK_RG46/2013	human
A/Anhui/1-YK_RG47/2013	human
A/Anhui/1-YK_RG48/2013	human
A/Anhui/1-YK_RG49/2013	human
A/Anhui/1-YK_RG50/2013	human
A/Anhui/1-YK_RG51/2013	human
A/Anhui/1-YK_RG52/2013	human
A/Anhui/1-YK_RG53/2013	human
A/Anhui/1-YK_RG54/2013	human
A/Anhui/1-YK_RG55/2013	human
A/Anhui/1-YK_RG56/2013	human
A/Anhui/1-YK_RG57/2013	human
A/Anhui/1-YK_RG58/2013	human
A/Anhui/1-YK_RG59/2013	human
A/Anhui/1-YK_RG60/2013	human
A/Anhui/1-YK_RG61/2013	human
A/Anhui/1-YK_RG62/2013	human

A/Anhui/1-YK_RG63/2013	human
A/Anhui/1-YK_RG64/2013	human
A/Anhui/1-YK_RG65/2013	human
A/Anhui/1-YK_RG66/2013	human
A/Anhui/1-YK_RG67/2013	human
A/Anhui/1-YK_RG68/2013	human
A/Anhui/1-YK_RG69/2013	human
A/Anhui/1-YK_RG70/2013	human
A/Anhui/1-YK_RG71/2013	human
A/Anhui/1-YK_RG72/2013	human
A/Anhui/1-YK_RG73/2013	human
A/Anhui/1-YK_RG74/2013	human
A/Anhui/1-YK_RG75/2013	human
A/Anhui/1-YK_RG76/2013	human
A/Anhui/1-YK_RG77/2013	human
A/Anhui/1-YK_RG78/2013	human
A/Anhui/1-YK_RG79/2013	human
A/Anhui/1-YK_RG80/2013	human
A/Anhui/1-YK_RG81/2013	human
A/Anhui/1-YK_RG82/2013	human
A/Anhui/1-YK_RG83/2013	human
A/Anhui/1-YK_RG84/2013	human
A/Anhui/1-YK_RG85/2013	human
A/Anhui/1-YK_RG86/2013	human
A/Anhui/1-YK_RG87/2013	human
A/Anhui/1-YK_RG88/2013	human
A/Anhui/1-YK_RG89/2013	human
A/Anhui/1-YK_RG90/2013	human
A/Anhui/1-YK_RG91/2013	human
A/Anhui/1-YK_RG92/2013	human
A/Anhui/1-YK_RG93/2013	human
A/Anhui/1-YK_RG94/2013	human
A/Anhui/1-YK_RG95/2013	human
A/Anhui/1-YK_RG96/2013	human
A/Anhui/1-YK_RG97/2013	human
A/Anhui/1-YK_RG98/2013	human
A/Anhui/1-YK_RG99/2013	human
A/Anhui/1-YK_RG100/2013	human
A/Anhui/1-YK_RG101/2013	human
A/Anhui/1-YK_RG102/2013	human
A/Anhui/1-YK_RG103/2013	human
A/Anhui/1-YK_RG104/2013	human
A/Anhui/1-YK_RG105/2013	human
A/Anhui/1-YK_RG106/2013	human
A/Anhui/1-YK_RG107/2013	human
A/Anhui/1-YK_RG108/2013	human
A/Anhui/1-YK_RG109/2013	human

A/Anhui/1-YK_RG110/2013	human
A/Anhui/1-YK_RG111/2013	human
A/Anhui/1-YK_RG112/2013	human
A/Anhui/1-YK_RG113/2013	human
A/Anhui/1-YK_RG114/2013	human
A/Anhui/1-YK_RG115/2013	human
A/Anhui/1-YK_RG116/2013	human
A/Anhui/1-YK_RG117/2013	human
A/Anhui/1-YK_RG118/2013	human
A/Anhui/1-YK_RG119/2013	human
A/Anhui/1-YK_RG120/2013	human
A/Anhui/1-YK_RG121/2013	human
A/Anhui/1-YK_RG122/2013	human
A/Anhui/1-YK_RG123/2013	human
A/Anhui/1-YK_RG124/2013	human
A/Anhui/1-YK_RG125/2013	human
A/Anhui/1-YK_RG126/2013	human
A/Anhui/1-YK_RG127/2013	human
A/Anhui/1-YK_RG128/2013	human
A/Anhui/1-YK_RG129/2013	human
A/Anhui/1-YK_RG130/2013	human
A/Anhui/1-YK_RG131/2013	human
A/Anhui/1-YK_RG132/2013	human
A/Anhui/1-YK_RG133/2013	human
A/Anhui/1-YK_RG134/2013	human
A/Anhui/1-YK_RG135/2013	human
A/Anhui/1-YK_RG136/2013	human
A/Anhui/1-YK_RG137/2013	human
A/Anhui/1-YK_RG138/2013	human
A/Anhui/1-YK_RG139/2013	human
A/Anhui/1-YK_RG140/2013	human
A/Anhui/1-YK_RG141/2013	human
A/Anhui/1-YK_RG142/2013	human
A/Anhui/1-YK_RG143/2013	human
A/Anhui/1-YK_RG144/2013	human
A/Anhui/1-YK_RG145/2013	human
A/Anhui/1-YK_RG146/2013	human
A/Anhui/1-YK_RG147/2013	human
A/Anhui/1-YK_RG148/2013	human
A/Anhui/1-YK_RG149/2013	human
A/Anhui/1-YK_RG150/2013	human
A/Anhui/1-YK_RG151/2013	human
A/Anhui/1-YK_RG152/2013	human
A/Anhui/1-YK_RG153/2013	human
A/Anhui/1-YK_RG154/2013	human
A/Anhui/1-YK_RG155/2013	human
A/Anhui/1-YK_RG156/2013	human

A/Anhui/1-YK_RG157/2013	human
A/Anhui/1-YK_RG158/2013	human
A/Anhui/1-YK_RG159/2013	human
A/Anhui/1-YK_RG160/2013	human
A/Anhui/1-YK_RG161/2013	human
A/Anhui/1-YK_RG162/2013	human
A/Anhui/1-YK_RG163/2013	human
A/Anhui/1-YK_RG164/2013	human
A/Anhui/1-YK_RG165/2013	human
A/Anhui/1-YK_RG166/2013	human
A/Anhui/1-YK_RG167/2013	human
A/Anhui/1-YK_RG168/2013	human
A/Anhui/1-YK_RG169/2013	human
A/Anhui/1-YK_RG170/2013	human
A/Anhui/1-YK_RG171/2013	human
A/Anhui/1-YK_RG172/2013	human
A/Anhui/1-YK_RG173/2013	human
A/Anhui/1-YK_RG174/2013	human
A/Anhui/1-YK_RG176/2013	human
A/Anhui/1-YK_RG177/2013	human
A/Anhui/1-YK_RG178/2013	human
A/Anhui/1-YK_RG179/2013	human
A/Anhui/1-YK_RG180/2013	human
A/Anhui/1-YK_RG181/2013	human
A/Anhui/1-YK_RG182/2013	human
A/Anhui/1-YK_RG183/2013	human
A/Anhui/1-YK_RG184/2013	human
A/Anhui/1-YK_RG185/2013	human
A/Anhui/1-YK_RG186/2013	human
A/Anhui/1-YK_RG187/2013	human
A/Anhui/1-YK_RG188/2013	human
A/Anhui/1-YK_RG189/2013	human
A/Anhui/1-YK_RG190/2013	human
A/Anhui/1-YK_RG191/2013	human
A/Anhui/1-YK_RG192/2013	human
A/Anhui/1-YK_RG193/2013	human
A/Anhui/1-YK_RG194/2013	human
A/Anhui/1-YK_RG195/2013	human
A/Anhui/1-YK_RG196/2013	human
A/Anhui/1-YK_RG197/2013	human
A/Anhui/1-YK_RG198/2013	human
A/Anhui/1-YK_RG199/2013	human
A/Anhui/1-YK_RG200/2013	human
A/Anhui/1-YK_RG201/2013	human
A/Anhui/1-YK_RG202/2013	human
A/duck/Bangladesh/26980/2015	duck
A/duck/Bangladesh/27042/2015	duck

A/duck/Bangladesh/26992/2015	duck
A/chicken/Tennessee/17-007147-2/2017	chicken
A/chicken/Tennessee/17-007431-3/2017	chicken
A/guinea fowl/Alabama/17-008272-2/2017	guinea_fowl
A/chicken/Tennessee/17-008279-4/2017	chicken
A/chicken/Alabama/17-008899-10/2017	chicken
A/chicken/Alabama/17-008899-11/2017	chicken
A/chicken/Alabama/17-008901-1/2017	chicken
A/chicken/Tennessee/17-007147-1/2017	chicken
A/chicken/Tennessee/17-007147-3/2017	chicken
A/chicken/Tennessee/17-007147-4/2017	chicken
A/chicken/Tennessee/17-007147-5/2017	chicken
A/chicken/Tennessee/17-007147-6/2017	chicken
A/chicken/Tennessee/17-007147-7/2017	chicken
A/chicken/Tennessee/17-007429-3/2017	chicken
A/chicken/Tennessee/17-007429-7/2017	chicken
A/chicken/Tennessee/17-008152-1/2017	chicken
A/chicken/Tennessee/17-008279-2/2017	chicken
A/chicken/Tennessee/17-008279-5/2017	chicken
A/chicken/Tennessee/17-008279-6/2017	chicken
A/duck/Alabama/17-008643-2/2017	duck
A/guinea fowl/Alabama/17-008645-2/2017	guinea_fowl
A/guinea fowl/Alabama/17-008646-1/2017	guinea_fowl
A/blue-winged teal/Wyoming/AH0099021/2016	blue-winged_teal
A/Fujian/1/2013	human
A/Guangdong/05/2013	human
A/blue-winged teal/Ohio/566/2006	blue-winged_teal
A/wild duck/Korea/CSM42-1/2011	wild_duck
A/wild duck/Korea/MHC39-26/2011	wild_duck
A/wild duck/Korea/MHC39-13/2011	wild_duck
A/wild duck/Korea/MHC35-25/2011	wild_duck
A/wild duck/Korea/MHC35-41/2011	wild_duck
A/wild duck/Korea/SH19-47/2010	wild_duck
A/wild duck/Korea/SH19-27/2010	wild_duck
A/wild duck/Korea/SH19-50/2010	wild_duck
A/wild duck/Korea/SH20-27/2008	wild_duck
A/mallard/Sweden/1621/2002	mallard
A/wild duck/Korea/SH19-44/2010	wild_duck
A/duck/Japan/AQ-HE29-22/2017	duck
A/northern shoveler/California/AH0076755/2016	northern_shoveler
A/blue-winged teal/Louisiana/UGAI15-1692/2015	blue-winged_teal
A/blue-winged teal/Louisiana/UGAI15-1367/2015	blue-winged_teal
A/Huizhou/01/2013	human
A/Shanghai/4664T/2013	human
A/duck/Wenzhou/YJYF24/2015	duck
A/Shanghai/5190T/2013	human
A/Shanghai/01/2014	human

A/Shanghai/CN02/2013	human
A/chicken/Zhejiang/DTID-ZJU01/2013	chicken
A/Nanjing/1/2013	human
A/environment/Nanjing/2913/2013	environment
A/Hangzhou/1/2013	human
A/Zhejiang/HZ1/2013	human
A/chicken/Zhejiang/C483/2013	chicken
A/chicken/Zhejiang/C481/2013	chicken
A/pigeon/Zhejiang/P2/2013	pigeon
A/pigeon/Zhejiang/P1/2013	pigeon
A/Wuxi/2/2013	human
A/Wuxi/1/2013	human
A/environment/Suzhou/14/2013	environment
A/Xuzhou/1/2013	human
A/environment/Wuxi/1/2013	environment
A/Nanchang/1/2013	Nanchang
A/duck/Jiangxi/3096/2009	duck
A/duck/Jiangxi/3141/2009	duck
A/duck/Jiangxi/3169/2009	duck
A/duck/Jiangxi/3190/2009	duck
A/duck/Jiangxi/3230/2009	duck
A/duck/Jiangxi/3286/2009	duck
A/duck/Jiangxi/3283/2009	duck
A/duck/Jiangxi/3152/2009	duck
A/chicken/Rizhao/515/2013	chicken
A/chicken/Rizhao/867/2013	chicken
A/chicken/Rizhao/871/2013	chicken
A/chicken/Guangdong/SD641/2013	chicken
A/chicken/Jiangsu/S002/2013	chicken
A/chicken/Jiangsu/SC035/2013	chicken
A/chicken/Jiangsu/SC099/2013	chicken
A/chicken/Jiangsu/SC537/2013	chicken
A/chicken/Jiangxi/SD001/2013	chicken
A/chicken/Shanghai/S1053/2013	chicken
A/chicken/Shanghai/S1055/2013	chicken
A/chicken/Shanghai/S1076/2013	chicken
A/chicken/Shanghai/S1077/2013	chicken
A/chicken/Shanghai/S1078/2013	chicken
A/chicken/Shanghai/S1079/2013	chicken
A/chicken/Shanghai/S1358/2013	chicken
A/chicken/Shanghai/S1410/2013	chicken
A/chicken/Shanghai/S1413/2013	chicken
A/chicken/Zhejiang/SD007/2013	chicken
A/chicken/Zhejiang/SD019/2013	chicken
A/chicken/Zhejiang/SD033/2013	chicken
A/duck/Anhui/SC702/2013	duck
A/duck/Zhejiang/SC410/2013	duck

A/environment/Fujian/SC337/2013	environment
A/environment/Henan/SC232/2013	environment
A/environment/Henan/SD429/2013	environment
A/environment/Shandong/SD038/2013	environment
A/environment/Shandong/SD039/2013	environment
A/environment/Shandong/SD049/2013	environment
A/environment/Shanghai/S1088/2013	environment
A/environment/Shanghai/S1436/2013	environment
A/environment/Shanghai/S1438/2013	environment
A/environment/Shanghai/S1439/2013	environment
A/homing pigeon/Jiangsu/SD184/2013	homing_pigeon
A/pigeon/Shanghai/S1069/2013	pigeon
A/pigeon/Shanghai/S1421/2013	pigeon
A/pigeon/Shanghai/S1423/2013	pigeon
A/wild pigeon/Jiangsu/SD001/2013	wild_pigeon
A/Nanjing/7/2013	human
A/Suzhou/5/2013	human
A/Nanjing/6/2013	human
A/Suzhou/3/2013	human
A/Wuxi/4/2013	human
A/Wuxi/3/2013	human
A/Zhenjiang/1/2013	human
A/Nanjing/4/2013	human
A/Nanjing/2/2013	human
A/environment/Zhenjiang/4/2013	environment
A/environment/Suzhou/8/2013	environment
A/chicken/Shanghai/019/2013	chicken
A/chicken/Shanghai/017/2013	chicken
A/Shanghai/JS01/2013	human
A/Shanghai/MH01/2013	human
A/Guangdong/1/2013	human
A/guinea fowl/Nebraska/17096/2011	guinea_fowl
A/turkey/Minnesota/32710/2011	turkey
A/turkey/Minnesota/31676/2009	turkey
A/turkey/Minnesota/14135-2/2009	turkey
A/environment/Shanghai/TY01/2013	environment
A/tree sparrow/Shanghai/01/2013	tree_sparrow
A/chicken/Guangdong/3135/2013	chicken
A/chicken/Guangdong/3640/2013	chicken
A/chicken/Guangdong/G1/2013	chicken
A/chicken/Guangdong/G2/2013	chicken
A/chicken/Guangdong/G3/2013	chicken
A/chicken/Guangdong/SD1/2013	chicken
A/Beijing/3/2013	human
A/Beijing/2/2013	human
A/Beijing/1/2013	human
A/Shanghai/PD-01/2014	human

A/environment/Shanghai/PD-JZ-01/2014	environment
A/chicken/Shanghai/PD-CN-02/2014	chicken
A/ruddy turnstone/Delaware/AI00-1538/2000	ruddy_turnstone
A/Taiwan/4-CGMH2/2014	human
A/Taiwan/4-CGMH1/2014	human
A/Zhejiang/LS01/2014	human
A/chicken/Zhejiang/DTID-ZJU06/2013	chicken
A/chicken/Henan/114/2013	chicken
A/chicken/Henan/141/2013	chicken
A/chicken/Shenzhen/727/2013	chicken
A/chicken/Shenzhen/742/2013	chicken
A/chicken/Shenzhen/898/2013	chicken
A/silkie chicken/Shenzhen/918/2013	silkie_chicken
A/silkie chicken/Shenzhen/919/2013	silkie_chicken
A/chicken/Shenzhen/1665/2013	chicken
A/chicken/Shenzhen/2110/2013	chicken
A/silkie chicken/Shenzhen/2134/2013	silkie_chicken
A/silkie chicken/Shenzhen/2139/2013	silkie_chicken
A/chicken/Shenzhen/2201/2013	chicken
A/chicken/Shenzhen/2293/2013	chicken
A/silkie chicken/Dongguan/3049/2013	silkie_chicken
A/silkie chicken/Dongguan/3166/2013	silkie_chicken
A/chicken/Dongguan/3219/2013	chicken
A/silkie chicken/Dongguan/3275/2013	silkie_chicken
A/silkie chicken/Dongguan/3281/2013	silkie_chicken
A/silkie chicken/Dongguan/3284/2013	silkie_chicken
A/chicken/Dongguan/3418/2013	chicken
A/chicken/Dongguan/3464/2013	chicken
A/silkie chicken/Dongguan/3528/2013	silkie_chicken
A/chicken/Dongguan/3544/2013	chicken
A/chicken/Dongguan/3582/2013	chicken
A/silkie chicken/Dongguan/3605/2013	silkie_chicken
A/silkie chicken/Dongguan/3606/2013	silkie_chicken
A/chicken/Shenzhen/3734/2013	chicken
A/chicken/Shenzhen/3780/2013	chicken
A/chicken/Dongguan/3894/2013	chicken
A/chicken/Dongguan/3935/2013	chicken
A/chicken/Dongguan/3945/2013	chicken
A/chicken/Dongguan/3972/2013	chicken
A/silkie chicken/Dongguan/3990/2013	silkie_chicken
A/chicken/Dongguan/4037/2013	chicken
A/chicken/Dongguan/4063/2013	chicken
A/chicken/Dongguan/4114/2013	chicken
A/silkie chicken/Dongguan/4126/2013	silkie_chicken
A/silkie chicken/Dongguan/4129/2013	silkie_chicken
A/chicken/Dongguan/4251/2013	chicken
A/chicken/Huzhou/3765/2013	chicken

A/chicken/Huzhou/3791/2013	chicken
A/chicken/Huzhou/3802/2013	chicken
A/chicken/Huzhou/4045/2013	chicken
A/chicken/Huzhou/4067/2013	chicken
A/chicken/Huzhou/4073/2013	chicken
A/chicken/Huzhou/4076/2013	chicken
A/chicken/Huzhou/4083/2013	chicken
A/chicken/Huzhou/4141/2013	chicken
A/silkie chicken/Huzhou/4213/2013	silkie_chicken
A/chicken/Jiaxing/4490/2013	chicken
A/chicken/Shaoxing/5087/2013	chicken
A/silkie chicken/Shaoxing/5130/2013	silkie_chicken
A/chicken/Shaoxing/5136/2013	chicken
A/chicken/Shaoxing/5186/2013	chicken
A/chicken/Shaoxing/5201/2013	chicken
A/chicken/Shaoxing/5224/2013	chicken
A/silkie chicken/Shaoxing/5235/2013	silkie_chicken
A/chicken/Shaoxing/5479/2013	chicken
A/chicken/Jiangxi/12486/2013	chicken
A/chicken/Jiangxi/12492/2013	chicken
A/chicken/Jiangxi/12544/2013	chicken
A/chicken/Jiangxi/12554/2013	chicken
A/Shenzhen/SP-Z93/2014	human
A/Shenzhen/SP17/2014	human
A/Shenzhen/SP-W1/2014	human
A/Shenzhen/SP16/2014	human
A/Shenzhen/SP38/2014	human
A/Shenzhen/SP48/2014	human
A/Shenzhen/SP49/2014	human
A/chicken/Dongguan/4119/2013	chicken
A/chicken/Shenzhen/801/2013	chicken
A/chicken/Dongguan/4040/2013	chicken
A/silkie chicken/Jiangxi/9472/2014	silkie_chicken
A/silkie chicken/Jiangxi/9476/2014	silkie_chicken
A/chicken/Jiangxi/9534/2014	chicken
A/chicken/Jiangxi/9558/2014	chicken
A/chicken/Jiangxi/10552/2014	chicken
A/chicken/Jiangxi/10870/2014	chicken
A/chicken/Jiangxi/10871/2014	chicken
A/chicken/Jiangxi/10873/2014	chicken
A/chicken/Jiangxi/10874/2014	chicken
A/chicken/Jiangxi/10877/2014	chicken
A/chicken/Jiangxi/10882/2014	chicken
A/chicken/Jiangxi/10885/2014	chicken
A/chicken/Jiangxi/10894/2014	chicken
A/chicken/Jiangxi/10896/2014	chicken
A/chicken/Jiangxi/10897/2014	chicken

A/chicken/Jiangxi/10939/2014	chicken
A/chicken/Jiangxi/10943/2014	chicken
A/chicken/Jiangxi/10945/2014	chicken
A/chicken/Jiangxi/10946/2014	chicken
A/chicken/Jiangxi/10947/2014	chicken
A/chicken/Jiangxi/10948/2014	chicken
A/chicken/Jiangxi/10950/2014	chicken
A/chicken/Jiangxi/10953/2014	chicken
A/chicken/Jiangxi/10954/2014	chicken
A/chicken/Jiangxi/10958/2014	chicken
A/chicken/Jiangxi/10959/2014	chicken
A/chicken/Jiangxi/10962/2014	chicken
A/chicken/Jiangxi/10965/2014	chicken
A/silkie chicken/Dongguan/157/2014	silkie_chicken
A/chicken/Dongguan/169/2014	chicken
A/chicken/Dongguan/173/2014	chicken
A/chicken/Dongguan/189/2014	chicken
A/chicken/Dongguan/449/2014	chicken
A/chicken/Dongguan/518/2014	chicken
A/chicken/Dongguan/536/2014	chicken
A/chicken/Dongguan/568/2014	chicken
A/chicken/Dongguan/584/2014	chicken
A/silkie chicken/Dongguan/635/2014	silkie_chicken
A/silkie chicken/Dongguan/656/2014	silkie_chicken
A/chicken/Dongguan/695/2014	chicken
A/chicken/Dongguan/856/2014	chicken
A/silkie chicken/Dongguan/963/2014	silkie_chicken
A/silkie chicken/Dongguan/988/2014	silkie_chicken
A/silkie chicken/Dongguan/991/2014	silkie_chicken
A/chicken/Dongguan/1057/2014	chicken
A/chicken/Dongguan/1075/2014	chicken
A/chicken/Dongguan/1143/2014	chicken
A/chicken/Dongguan/1177/2014	chicken
A/silkie chicken/Dongguan/1264/2014	silkie_chicken
A/silkie chicken/Dongguan/1268/2014	silkie_chicken
A/silkie chicken/Dongguan/1274/2014	silkie_chicken
A/chicken/Dongguan/1297/2014	chicken
A/chicken/Dongguan/1312/2014	chicken
A/chicken/Dongguan/1374/2014	chicken
A/silkie chicken/Dongguan/1448/2014	silkie_chicken
A/silkie chicken/Dongguan/1450/2014	silkie_chicken
A/silkie chicken/Dongguan/1451/2014	silkie_chicken
A/chicken/Dongguan/1456/2014	chicken
A/chicken/Dongguan/1494/2014	chicken
A/chicken/Dongguan/1505/2014	chicken
A/chicken/Dongguan/1506/2014	chicken
A/silkie chicken/Dongguan/1516/2014	silkie_chicken

A/chicken/Dongguan/1526/2014	chicken
A/chicken/Dongguan/1619/2014	chicken
A/chicken/Dongguan/1666/2014	chicken
A/chicken/Dongguan/1673/2014	chicken
A/chicken/Dongguan/1091/2014	chicken
A/chicken/Dongguan/1108/2014	chicken
A/chicken/Dongguan/178/2014	chicken
A/chicken/Dongguan/191/2014	chicken
A/chicken/Dongguan/210/2014	chicken
A/chicken/Dongguan/213/2014	chicken
A/chicken/Dongguan/237/2014	chicken
A/chicken/Dongguan/248/2014	chicken
A/chicken/Dongguan/262/2014	chicken
A/chicken/Dongguan/709/2014	chicken
A/chicken/Dongguan/711/2014	chicken
A/chicken/Dongguan/748/2014	chicken
A/chicken/Dongguan/749/2014	chicken
A/chicken/Dongguan/803/2014	chicken
A/chicken/Dongguan/815/2014	chicken
A/chicken/Dongguan/835/2014	chicken
A/chicken/Dongguan/836/2014	chicken
A/chicken/Dongguan/843/2014	chicken
A/chicken/Dongguan/850/2014	chicken
A/chicken/Dongguan/851/2014	chicken
A/chicken/Dongguan/874/2014	chicken
A/chicken/Dongguan/899/2014	chicken
A/chicken/Dongguan/934/2014	chicken
A/silkie chicken/Dongguan/969/2014	silkie_chicken
A/silkie chicken/Dongguan/979/2014	silkie_chicken
A/silkie chicken/Dongguan/981/2014	silkie_chicken
A/silkie chicken/Dongguan/986/2014	silkie_chicken
A/chicken/Dongguan/1009/2014	chicken
A/chicken/Dongguan/1022/2014	chicken
A/chicken/Dongguan/1124/2014	chicken
A/chicken/Dongguan/1188/2014	chicken
A/chicken/Dongguan/1230/2014	chicken
A/silkie chicken/Dongguan/1271/2014	silkie_chicken
A/chicken/Dongguan/1303/2014	chicken
A/chicken/Dongguan/1314/2014	chicken
A/chicken/Dongguan/1318/2014	chicken
A/chicken/Dongguan/1382/2014	chicken
A/chicken/Dongguan/1393/2014	chicken
A/chicken/Dongguan/1401/2014	chicken
A/chicken/Dongguan/1421/2014	chicken
A/chicken/Dongguan/1527/2014	chicken
A/chicken/Dongguan/1533/2014	chicken
A/silkie chicken/Dongguan/1641/2014	silkie_chicken

A/chicken/Dongguan/1690/2014	chicken
A/silkie chicken/Dongguan/523/2014	silkie_chicken
A/chicken/Dongguan/575/2014	chicken
A/chicken/Shenzhen/138/2014	chicken
A/silkie chicken/Shantou/1406/2014	silkie_chicken
A/chicken/Shantou/1552/2014	chicken
A/chicken/Shantou/1554/2014	chicken
A/chicken/Shantou/1556/2014	chicken
A/chicken/Dongguan/397/2014	chicken
A/silkie chicken/Dongguan/953/2014	silkie_chicken
A/silkie chicken/Dongguan/997/2014	silkie_chicken
A/chicken/Dongguan/1100/2014	chicken
A/silkie chicken/Dongguan/1519/2014	silkie_chicken
A/Shenzhen/SP58/2014	human
A/Shenzhen/SP60/2014	human
A/Shenzhen/SP75/2014	human
A/Shenzhen/SP62/2014	human
A/Shenzhen/SP113/2014	human
A/Shenzhen/SP116/2014	human
A/Shenzhen/SP118/2014	human
A/chicken/Jiangxi/12206/2014	chicken
A/chicken/Jiangxi/12210/2014	chicken
A/chicken/Jiangxi/12216/2014	chicken
A/chicken/Jiangxi/12221/2014	chicken
A/chicken/Jiangxi/12245/2014	chicken
A/chicken/Jiangxi/12249/2014	chicken
A/chicken/Jiangxi/12254/2014	chicken
A/chicken/Jiangxi/12261/2014	chicken
A/chicken/Jiangxi/12274/2014	chicken
A/chicken/Jiangxi/12768/2014	chicken
A/chicken/Jiangxi/13207/2014	chicken
A/chicken/Jiangxi/13209/2014	chicken
A/chicken/Jiangxi/12240/2014	chicken
A/chicken/Jiangxi/13220/2014	chicken
A/chicken/Jiangxi/13230/2014	chicken
A/chicken/Jiangxi/13250/2014	chicken
A/chicken/Jiangxi/13268/2014	chicken
A/chicken/Jiangxi/12200/2014	chicken
A/chicken/Jiangxi/9513/2014	chicken
A/Shenzhen/SP126/2014	human
A/Shenzhen/SP139/2014	human
A/chicken/Dongguan/1096/2014	chicken
A/chicken/Dongguan/1358/2014	chicken
A/chicken/Jiangxi/13510/2014	chicken
A/chicken/Jiangxi/13513/2014	chicken
A/chicken/Jiangxi/13518/2014	chicken
A/chicken/Jiangxi/13519/2014	chicken

A/chicken/Jiangxi/13538/2014	chicken
A/chicken/Jiangxi/13546/2014	chicken
A/chicken/Jiangxi/9497/2014	chicken
A/chicken/Jiangxi/9530/2014	chicken
A/chicken/Jiangxi/12201/2014	chicken
A/chicken/Jiangxi/12208/2014	chicken
A/chicken/Jiangxi/12217/2014	chicken
A/chicken/Jiangxi/12219/2014	chicken
A/chicken/Jiangxi/12222/2014	chicken
A/chicken/Jiangxi/12232/2014	chicken
A/chicken/Jiangxi/12239/2014	chicken
A/chicken/Jiangxi/12243/2014	chicken
A/chicken/Jiangxi/12247/2014	chicken
A/chicken/Jiangxi/12248/2014	chicken
A/chicken/Jiangxi/12251/2014	chicken
A/chicken/Jiangxi/12256/2014	chicken
A/chicken/Jiangxi/12260/2014	chicken
A/chicken/Jiangxi/12264/2014	chicken
A/chicken/Jiangxi/12265/2014	chicken
A/chicken/Jiangxi/12273/2014	chicken
A/chicken/Jiangxi/13210/2014	chicken
A/chicken/Jiangxi/13252/2014	chicken
A/chicken/Jiangxi/13493/2014	chicken
A/chicken/Jiangxi/13507/2014	chicken
A/chicken/Jiangxi/13512/2014	chicken
A/chicken/Jiangxi/13524/2014	chicken
A/chicken/Jiangxi/13530/2014	chicken
A/chicken/Jiangxi/13537/2014	chicken
A/chicken/Jiangxi/13543/2014	chicken
A/chicken/Jiangxi/13544/2014	chicken
A/chicken/Jiangxi/13548/2014	chicken
A/chicken/Jiangxi/13551/2014	chicken
A/chicken/Jiangxi/13556/2014	chicken
A/chicken/Jiangxi/13564/2014	chicken
A/chicken/Jiangxi/14023/2014	chicken
A/chicken/Jiangxi/14033/2014	chicken
A/chicken/Jiangxi/14479/2014	chicken
A/chicken/Jiangxi/14482/2014	chicken
A/chicken/Jiangxi/14513/2014	chicken
A/chicken/Jiangxi/14515/2014	chicken
A/chicken/Jiangxi/14517/2014	chicken
A/chicken/Jiangxi/14518/2014	chicken
A/chicken/Jiangxi/14530/2014	chicken
A/chicken/Jiangxi/14554/2014	chicken
A/chicken/Shantou/2537/2014	chicken
A/chicken/Shantou/2538/2014	chicken
A/chicken/Shantou/2539/2014	chicken

A/chicken/Shantou/2546/2014	chicken
A/chicken/Shantou/2550/2014	chicken
A/chicken/Shantou/2556/2014	chicken
A/chicken/Shantou/2562/2014	chicken
A/chicken/Jiangxi/15044/2014	chicken
A/chicken/Jiangxi/15524/2014	chicken
A/silkie chicken/Shantou/2050/2014	silkie_chicken
A/silkie chicken/Shantou/2056/2014	silkie_chicken
A/chicken/Shantou/3057/2014	chicken
A/chicken/Shantou/4325/2014	chicken
A/chicken/Shantou/4816/2014	chicken
A/chicken/Shantou/4824/2014	chicken
A/chicken/Shantou/4832/2014	chicken
A/chicken/Shantou/4833/2014	chicken
A/chicken/Jiangxi/18008/2014	chicken
A/chicken/Jiangxi/18449/2014	chicken
A/chicken/Jiangxi/18482/2014	chicken
A/chicken/Jiangxi/18513/2014	chicken
A/chicken/Jiangxi/18515/2014	chicken
A/chicken/Zhangzhou/8585/2014	chicken
A/duck/Zhejiang/LS02/2014	duck
A/Shanghai/ION/2013	human
A/Quzhou/1/2015	human
A/Quzhou/2/2015	human
A/chicken/Wenzhou/WZTSLG02/2015	chicken
A/chicken/Wenzhou/HATSLG01/2015	chicken
A/chicken/China/028/2014	chicken
A/Huai'an/083/2014	human
A/chicken/Huai'an/003/2015	chicken
A/chicken/Huai'an/007/2014	chicken
A/Huai'an/001/2015	human
A/Huai'an/002/2015	human
A/Huai'an/062/2014	human
A/Huai'an/065/2014	human
A/Huai'an/074/2014	human
A/environment/Korea/W410/2011	environment
A/Guangdong/6/2013	human
A/Guangdong/7/2013	human
A/magpie robin/Hubei/SZY300/2015	magpie_robin
A/American green-winged teal/Idaho/AH0006121/2015	American_green-winged_teal
A/Yunnan/0129/2017	human
A/chicken/Jiangsu/JT114/2015	chicken
A/chicken/Jiangsu/JT115/2015	chicken
A/chicken/Jiangsu/JT119/2015	chicken
A/chicken/Jiangsu/JT123/2015	chicken
A/chicken/Anhui/AH271/2015	chicken
A/chicken/Jiangsu/XZ250/2015	chicken

A/chicken/Anhui/AH284/2015	chicken
A/chicken/Jiangsu/XZ252/2015	chicken
A/chicken/Shandong/SD69/2015	chicken
A/chicken/Jiangsu/LY79/2015	chicken
A/chicken/Jiangsu/RG126/2015	chicken
A/chicken/Shandong/SD183/2016	chicken
A/chicken/Jiangsu/JS11/2016	chicken
A/chicken/Shandong/SD216/2016	chicken
A/chicken/Jiangsu/JT156/2016	chicken
A/chicken/Jiangsu/YZ30/2017	chicken
A/chicken/Liaoning/LN1/2016	chicken
A/chicken/Hebei/HB13/2016	chicken
A/chicken/Zhejiang/ZJ14/2017	chicken
A/chicken/Guangdong/GD15/2016	chicken
A/chicken/Zhejiang/ZJ19/2017	chicken
A/chicken/Guangdong/GD20/2017	chicken
A/chicken/Jiangsu/LY246/2017	chicken
A/chicken/Jiangsu/0116/2017	chicken
A/chicken/Jiangsu/JT186/2017	chicken
A/chicken/Jiangsu/TZ45/2017	chicken
A/chicken/Zhejiang/JH16/2017	chicken
A/chicken/Guangdong/30/2017	chicken
A/chicken/Guangdong/Q1/2016	chicken
A/chicken/Guangdong/Q26/2017	chicken
A/chicken/Guangdong/Q39/2017	chicken
A/chicken/Heinan/ZZ01/2017	chicken
A/chicken/Fujian/S1017/2014	chicken
A/chicken/Fujian/S1085/2014	chicken
A/chicken/Fujian/S1096/2015	chicken
A/chicken/Fujian/S1322/2014	chicken
A/chicken/Fujian/S4215/2014	chicken
A/chicken/Fujian/S4219/2014	chicken
A/chicken/Fujian/S4248/2014	chicken
A/chicken/Fujian/S4518/2015	chicken
A/chicken/Fujian/SD005/2015	chicken
A/chicken/Guangdong/S1200/2015	chicken
A/chicken/Guangdong/SC086/2013	chicken
A/chicken/Guangdong/SC092/2013	chicken
A/chicken/Guangdong/SD002/2014	chicken
A/chicken/Guangdong/SD007/2015	chicken
A/chicken/Guangdong/SD010/2017	chicken
A/chicken/Guangdong/SD014/2014	chicken
A/chicken/Guangdong/SD015/2013	chicken
A/chicken/Guangdong/SD018/2015	chicken
A/chicken/Guangdong/SD021/2014	chicken
A/chicken/Guangdong/SD023/2014	chicken
A/chicken/Guangdong/SD025/2014	chicken

A/chicken/Guangdong/SD027/2015	chicken
A/chicken/Guangdong/SD027/2017	chicken
A/chicken/Guangdong/SD028/2017	chicken
A/chicken/Guangdong/SD029/2014	chicken
A/chicken/Guangdong/SD031/2017	chicken
A/chicken/Guangdong/SD032/2017	chicken
A/chicken/Guangdong/SD034/2017	chicken
A/chicken/Guangdong/SD1433/2016	chicken
A/chicken/Guangxi/SD004/2014	chicken
A/chicken/Guangxi/SD005/2014	chicken
A/chicken/Guangxi/SD006/2014	chicken
A/chicken/Hebei/S1257/2016	chicken
A/chicken/Henan/S1425/2014	chicken
A/chicken/Hunan/S12753/2016	chicken
A/chicken/Hunan/SD010/2014	chicken
A/chicken/Hunan/SD017/2014	chicken
A/chicken/Hunan/SD019/2014	chicken
A/chicken/Jiangsu/S1045/2016	chicken
A/chicken/Jiangsu/S1361/2015	chicken
A/chicken/Jiangsu/S1441/2016	chicken
A/chicken/Jiangsu/S1460/2016	chicken
A/chicken/Jiangsu/S4519/2015	chicken
A/chicken/Jiangsu/SD012/2015	chicken
A/chicken/Jiangxi/S10017/2015	chicken
A/chicken/Jiangxi/S10844/2015	chicken
A/chicken/Jiangxi/S11228/2015	chicken
A/chicken/Jiangxi/S40993/2015	chicken
A/chicken/Jilin/SD009/2016	chicken
A/chicken/Ningxia/S1152/2014	chicken
A/chicken/Shanghai/S4100/2015	chicken
A/chicken/Xinjiang/SD033/2014	chicken
A/chicken/Xinjiang/SD036/2014	chicken
A/chicken/Zhejiang/S1074/2016	chicken
A/chicken/Zhejiang/S1190/2015	chicken
A/chicken/Zhejiang/S4008/2013	chicken
A/chicken/Zhejiang/S4017/2013	chicken
A/chicken/Zhejiang/S4060/2014	chicken
A/chicken/Zhejiang/S4071/2013	chicken
A/chicken/Zhejiang/S4079/2013	chicken
A/chicken/Zhejiang/S4135/2013	chicken
A/chicken/Zhejiang/S4168/2013	chicken
A/chicken/Zhejiang/SD001/2016	chicken
A/duck/Fujian/S4170/2014	duck
A/duck/Guangxi/SDY129/2014	duck
A/duck/Hunan/S11682/2015	duck
A/duck/Hunan/S40506/2015	duck
A/duck/Hunan/S40685/2015	duck

A/duck/Jiangsu/S1181/2014	duck
A/duck/Jiangsu/S1220/2016	duck
A/duck/Jiangsu/S1700/2016	duck
A/duck/Shanghai/SD015/2015	duck
A/duck/Shanghai/SD016/2015	duck
A/duck/Zhejiang/S1375/2016	duck
A/duck/Zhejiang/S4488/2014	duck
A/duck/Zhejiang/S4489/2014	duck
A/environment/Fujian/SD003/2014	environment
A/environment/Hebei/SC069/2013	environment
A/environment/Hunan/S40858/2015	environment
A/environment/Hunan/SD009/2015	environment
A/environment/Qinghai/S3032/2014	environment
A/duck/Anhui/S702/2013	duck
A/WuXi/0213/2016	human
A/WuXi/0409/2014	human
A/chicken/Heilongjiang/BQC01/2017	chicken
A/goose/Guangdong/Q38/2017	goose
A/goose/Guangdong/Q70/2017	goose
A/Changsha/44/2017	human
A/chicken/Heilongjiang/LJS15/2017	chicken
A/chicken/Anhui/AH388/2017	chicken
A/chicken/Anhui/AH395/2017	chicken
A/chicken/Jiangsu/TM261/2017	chicken
A/chicken/Jiangsu/JS/02/71/2017	chicken
A/chicken/Jiangsu/YZLH37/2017	chicken
A/chicken/Guangxi/GX110/2017	chicken
A/chicken/Henan/HNXY1/2017	chicken
A/chicken/Guangxi/GX102/2017	chicken
A/chicken/Hebei/SJ27/2017	chicken
A/chicken/Guangdong/HZLH1/2017	chicken
A/chicken/Guangdong/SD008/2017	chicken
A/duck/Jiangxi/3292/2009	duck
A/chicken/Henan/117/2013	chicken
A/chicken/Henan/97/2013	chicken
A/silkie chicken/Dongguan/3522/2013	silkie_chicken
A/wild bird/Korea/A3/11	wild_bird
A/wild bird/Korea/A9/11	wild_bird
A/spot-billed duck/Korea/447/11	spot-billed_duck
A/Zhejiang/DTID-ZJU10/2013	human
A/Taiwan/S02076/2013	human
A/chicken/Jiangxi/10962/2014	chicken
A/Shanghai/5190T/2013	human
A/Hong_Kong/8113530/2014	human
A/chicken/Jiangxi/10965/2014	chicken
A/Guizhou/01502/2014	human
A/Environment/Yangzhou/c13/2013	Environment

A/Nanjing/105/2014	human
A/Hangzhou/30/2014	human
A/Hangzhou/40/2014	human
A/Hangzhou/112/2014	human
A/Wuxi/419/2014	human
A/Hangzhou/118/2014	human
A/Changzhou/416/2014	human
A/Environment/Wuxi/wx02/2014	Environment
A/Environment/Suzhou/sz21/2014	Environment
A/silkie_chicken/Dongguan/969/2014	silkie_chicken
A/silkie_chicken/Dongguan/979/2014	silkie_chicken
A/Environment/Suzhou/sz10/2014	Environment
A/silkie_chicken/Dongguan/981/2014	silkie_chicken
A/silkie_chicken/Dongguan/986/2014	silkie_chicken
A/Quzhou/1/2015	human
A/Quzhou/2/2015	human
A/Hangzhou/2/2013	human
A/Hangzhou/3/2013	human
A/environment/Hangzhou/34-1/2013	environment
A/silkie_chicken/Dongguan/1271/2014	silkie_chicken
A/Wuxi/1/2013	human
A/environment/Wuxi/1/2013	environment
A/Wuxi/2/2013	human
A/Environment/Changzhou/cz95/2014	Environment
A/Yancheng/1121/2014	human
A/chicken/Hunan/02.07__YYFQHJ19Y-O/2015	chicken
A/Nanjing/M2/2013	human
A/Nanjing/f0874/2013	human
A/Nanjing/3/2013	human
A/chicken/Dongguan/1100/2014	chicken
A/Nanjing/5/2013	human
A/chicken/Wenzhou/HATSLG01/2015	chicken
A/duck/Wenzhou/YJYF24/2015	duck
A/ruddy_turnstone/Delaware_Bay/220/1995	ruddy_turnstone
A/chicken/Wenzhou/WZTSLG02/2015	chicken
A/Shanghai/4/2013	human
A/Human/Shenzhen/1/2013	human
A/Human/Shenzhen/2/2014	human
A/Zhejiang/01/2013	human
A/Shanghai/3/2013	human
A/Human/Shenzhen/5/2014	human
A/Human/Shenzhen/6/2014	human
A/Human/Shenzhen/3/2014	human
A/Human/Shenzhen/4/2014	human
A/Human/Shenzhen/9/2014	human
A/Human/Shenzhen/16/2014	human
A/Human/Shenzhen/7/2014	human

A/Human/Shenzhen/8/2014	human
A/Human/Shenzhen/4/2015	human
A/Human/Shenzhen/17/2014	human
A/Human/Shenzhen/1/2015	human
A/environment/Shenzhen/3/2013	environment
A/Human/Shenzhen/5/2015	human
A/Human/Shenzhen/6/2015	human
A/environment/Shenzhen/4/2013	environment
A/chicken/Hangzhou/174/2013	chicken
A/Taiwan/3/2013	human
A/chicken/Yunnan/03.16_DQXYL0034-Z-O/2015	chicken
A/Hangzhou/254/2013	human
A/Hangzhou/327/2013	human
A/Zhejiang/KLED-XS01/2014	human
A/Zhejiang/KLED-XS03/2014	human
A/Zhejiang/KLED-XS02T/2014	human
A/Zhejiang/KLED-XS02S/2014	human
A/Anhui/DEWH72-01/2013	human
A/Anhui/DEWH72-04/2013	human
A/Anhui/DEWH72-05/2013	human
A/Anhui/1-DEWH730/2013	human
A/Anhui/DEWH72-02/2013	human
A/Anhui/DEWH72-03/2013	human
A/Anhui/DEWH72-08/2013	human
A/Anhui/DEWH72-09/2013	human
A/Anhui/DEWH72-06/2013	human
A/Anhui/DEWH72-07/2013	human
A/Shanghai/MH01/2013	human
A/Hangzhou/169/2013	human
A/chicken/Hangzhou/48-1/2013	chicken
A/chicken/Hangzhou/50-1/2013	chicken
A/Hangzhou/173/2013	human
A/environment/Hangzhou/37/2013	environment
A/environment/Hangzhou/109-1/2013	environment
A/Shanghai/01/2014	human
A/Duck/Guangdong/DG527/2014	Duck
A/Taiwan/1/2014	human
A/Chicken/Guangdong/DG592/2014	Chicken
A/Chicken/Guangdong/DG478/2014	Chicken
A/Chicken/Guangdong/DG479/2014	Chicken
A/Chicken/Guangdong/DG593/2014	Chicken
A/Taiwan/2/2014	human
A/Chicken/Guangdong/HZ098/2015	Chicken
A/Environment/Guangdong/DG104/2015	Environment
A/Duck/Guangdong/DG103/2015	Duck
A/Chicken/Guangdong/GZ068/2015	Chicken
A/Chicken/Guangdong/CZ145/2015	Chicken

A/Chicken/Guangdong/DG120/2015	Chicken
A/Environment/Guangdong/DG127/2015	Environment
A/Chicken/Guangdong/SW153/2015	Chicken
A/Chicken/Guangdong/SW154/2015	Chicken
A/Jiangsu/1/2013	human
A/chicken/Rizhao/867/2013	chicken
A/Environment/Xinjiang/98679/2014	Environment
A/Guangdong/15SF018/2015	human
A/Guizhou/03240/2015	human
A/Guangdong/02496/2014	human
A/Xinjiang/05845/2015	human
A/Jiangsu/98342/2014	human
A/XinjiangBintuan/99117/2014	human
A/Hong_Kong/3263/2014	human
A/Shantou/1002/2014	human
A/tree_sparrow/Shanghai/01/2013	tree_sparrow
A/duck/Jiangxi/3283/2009	duck
A/duck/Jiangxi/3152/2009	duck
A/Hong_Kong/2212982/2014	human
A/duck/Jiangxi/3141/2009	duck
A/duck/Jiangxi/3169/2009	duck
A/duck/Jiangxi/3096/2009	duck
A/Guangdong/1/2013	human
A/duck/Jiangxi/3230/2009	duck
A/duck/Jiangxi/3286/2009	duck
A/duck/Jiangxi/3190/2009	duck
A/environment/Nanjing/2913/2013	environment
A/Nanjing/1/2013	human
A/Hong_Kong/2550/2015	human
A/Hong_Kong/8130773/2015	human
A/turkey/Minnesota/14135-2/2009	turkey
A/turkey/Minnesota/32710/2011	turkey
A/turkey/Minnesota/31676/2009	turkey
A/guinea_fowl/Nebraska/17096/2011	guinea_fowl
A/Taiwan/1/2013	human
A/mallard/Sweden/1621/2002	mallard
A/Environment/Zhejiang/5/2014	Environment
A/Huizhou/01/2013	human
A/Changsha/1/2013	human
A/Changsha/2/2013	human
A/Taiwan/01/2013	human
A/mallard/Minnesota/AI09-3770/2009	mallard
A/ruddy_turnstone/Delaware/AI00-1538/2000	ruddy_turnstone
A/Zhejiang/22/2013	human
A/duck/Sunan/040802G/2013	duck
A/blue-winged_teal/Ohio/566/2006	blue-winged_teal
A/Chicken/Suzhou/097-1/2013	Chicken

A/shoveler/Egypt/00215-NAMRU3/2007	shoveler
A/Nanjing/2/2013	human
A/environment/Zhenjiang/4/2013	environment
A/Zhenjiang/1/2013	human
A/Nanjing/4/2013	human
A/environment/Suzhou/8/2013	environment
A/Suzhou/3/2013	human
A/Nanjing/6/2013	human
A/Wuxi/3/2013	human
A/Wuxi/4/2013	human
A/Suzhou/5/2013	human
A/Nanjing/7/2013	human
A/chicken/Shanghai/017/2013	chicken
A/chicken/Shanghai/019/2013	chicken
A/Hong_Kong/8194273/2014	human
A/Shanghai/2/2013	human
A/Anhui/1/2013	human
A/Shanghai/1/2013	human
A/chicken/Guangdong/G1/2013	chicken
A/Xuzhou/1/2013	human
A/chicken/Guangdong/G2/2013	chicken
A/chicken/Guangdong/3135/2013	chicken
A/environment/Suzhou/14/2013	environment
A/chicken/Guangdong/3640/2013	chicken
A/chicken/Guangdong/G3/2013	chicken
A/chicken/Guangdong/SD1/2013	chicken
A/Shandong/0068A/2013	human
A/Hunan/0070A/2013	human
A/silkie_chicken/Shenzhen/918/2013	silkie_chicken
A/silkie_chicken/Shenzhen/919/2013	silkie_chicken
A/pigeon/Shanghai/S1423/2013	pigeon
A/pigeon/Shanghai/S1421/2013	pigeon
A/wild_pigeon/Jiangsu/SD001/2013	wild_pigeon
A/environment/Shanghai/S1438/2013	environment
A/homing_pigeon/Jiangsu/SD184/2013	homing_pigeon
A/environment/Shanghai/S1439/2013	environment
A/environment/Shandong/SD039/2013	environment
A/environment/Shandong/SD038/2013	environment
A/environment/Shanghai/S1436/2013	environment
A/environment/Shandong/SD049/2013	environment
A/environment/Fujian/SC337/2013	environment
A/silkie_chicken/Dongguan/3528/2013	silkie_chicken
A/duck/Zhejiang/SC410/2013	duck
A/environment/Henan/SD429/2013	environment
A/environment/Henan/SC232/2013	environment
A/chicken/Zhejiang/SD019/2013	chicken
A/chicken/Zhejiang/SD007/2013	chicken

A/duck/Anhui/SC702/2013	duck
A/chicken/Zhejiang/SD033/2013	chicken
A/chicken/Shanghai/S1358/2013	chicken
A/chicken/Shanghai/S1080/2013	chicken
A/chicken/Shanghai/S1413/2013	chicken
A/chicken/Shanghai/S1410/2013	chicken
A/chicken/Shanghai/S1077/2013	chicken
A/chicken/Shanghai/S1076/2013	chicken
A/chicken/Shanghai/S1079/2013	chicken
A/chicken/Shanghai/S1078/2013	chicken
A/chicken/Jiangsu/SC537/2013	chicken
A/chicken/Jiangsu/SC099/2013	chicken
A/chicken/Shanghai/S1055/2013	chicken
A/chicken/Jiangxi/SD001/2013	chicken
A/chicken/Guangdong/SD641/2013	chicken
A/chicken/Jiangsu/SC035/2013	chicken
A/chicken/Jiangsu/S002/2013	chicken
A/silkie_chicken/Dongguan/3606/2013	silkie_chicken
A/silkie_chicken/Dongguan/3605/2013	silkie_chicken
A/Beijing/02/2013	human
A/Anhui/04/2013	human
A/Henan/02/2013	human
A/Hunan/0070/2013	human
A/silkie_chicken/Dongguan/3990/2013	silkie_chicken
A/Fujian/04/2013	human
A/Fujian/02/2013	human
A/Fujian/03/2013	human
A/Guangxi/08970/2014	human
A/Fujian/05/2013	human
A/Guangxi/08309/2014	human
A/silkie_chicken/Dongguan/4126/2013	silkie_chicken
A/silkie_chicken/Dongguan/4129/2013	silkie_chicken
A/chicken/Huzhou/3791/2013	chicken
A/chicken/Huzhou/3802/2013	chicken
A/chicken/Huzhou/3765/2013	chicken
A/chicken/Jiangxi/12206/2014	chicken
A/chicken/Jiangxi/12221/2014	chicken
A/chicken/Jiangxi/12210/2014	chicken
A/chicken/Jiangxi/12216/2014	chicken
A/chicken/Jiangxi/12254/2014	chicken
A/chicken/Jiangxi/12261/2014	chicken
A/chicken/Jiangxi/12245/2014	chicken
A/chicken/Huzhou/4073/2013	chicken
A/chicken/Huzhou/4045/2013	chicken
A/chicken/Huzhou/4067/2013	chicken
A/chicken/Huzhou/4076/2013	chicken
A/chicken/Huzhou/4083/2013	chicken

A/chicken/Huzhou/4141/2013	chicken
A/chicken/Jiaxing/4490/2013	chicken
A/chicken/Shaoxing/5136/2013	chicken
A/chicken/Shaoxing/5087/2013	chicken
A/silkie_chicken/Shaoxing/5130/2013	silkie_chicken
A/chicken/Shaoxing/5186/2013	chicken
A/chicken/Jiangxi/13207/2014	chicken
A/chicken/Jiangxi/13209/2014	chicken
A/chicken/Jiangxi/12274/2014	chicken
A/chicken/Jiangxi/12768/2014	chicken
A/chicken/Jiangxi/13220/2014	chicken
A/chicken/Jiangxi/13230/2014	chicken
A/chicken/Jiangxi/12240/2014	chicken
A/chicken/Jiangxi/13268/2014	chicken
A/chicken/Jiangxi/13250/2014	chicken
A/chicken/Jiangxi/9513/2014	chicken
A/chicken/Shaoxing/5224/2013	chicken
A/chicken/Jiangxi/13518/2014	chicken
A/Anhui/1-YK_RG01/2013	human
A/Anhui/1-YK_RG03/2013	human
A/chicken/Jiangxi/13519/2014	chicken
A/chicken/Shaoxing/5201/2013	chicken
A/chicken/Jiangxi/13513/2014	chicken
A/Anhui/1-YK_RG06/2013	human
A/chicken/Shaoxing/5479/2013	chicken
A/Anhui/1-YK_RG07/2013	human
A/chicken/Jiangxi/13546/2014	chicken
A/Anhui/1-YK_RG02/2013	human
A/silkie_chicken/Shaoxing/5235/2013	silkie_chicken
A/Anhui/1-YK_RG04/2013	human
A/Anhui/1-YK_RG08/2013	human
A/chicken/Jiangxi/12544/2013	chicken
A/Anhui/1-YK_RG12/2013	human
A/Anhui/1-YK_RG05/2013	human
A/chicken/Jiangxi/12486/2013	chicken
A/Anhui/1-YK_RG09/2013	human
A/chicken/Jiangxi/12492/2013	chicken
A/Anhui/1-YK_RG15/2013	human
A/turkey/Minnesota/1/1988	turkey
A/Anhui/1-YK_RG14/2013	human
A/chicken/Jiangxi/12554/2013	chicken
A/Anhui/1-YK_RG10/2013	human
A/Anhui/1-YK_RG13/2013	human
A/Anhui/1-YK_RG18/2013	human
A/Anhui/1-YK_RG20/2013	human
A/Anhui/1-YK_RG16/2013	human
A/Anhui/1-YK_RG17/2013	human

A/Anhui/1-YK_RG23/2013	human
A/Anhui/1-YK_RG24/2013	human
A/Anhui/1-YK_RG21/2013	human
A/Anhui/1-YK_RG22/2013	human
A/Anhui/1-YK_RG26/2013	human
A/Anhui/1-YK_RG27/2013	human
A/Anhui/1-YK_RG19/2013	human
A/Hangzhou/1/2013	human
A/Anhui/1-YK_RG25/2013	human
A/Anhui/1-YK_RG31/2013	human
A/Anhui/1-YK_RG28/2013	human
A/Chicken/Shanghai/S1053/2013	Chicken
A/Anhui/1-YK_RG29/2013	human
A/Anhui/1-YK_RG30/2013	human
A/Anhui/1-YK_RG34/2013	human
A/Anhui/1-YK_RG36/2013	human
A/Environment/Shanghai/S1088/2013	Environment
A/Anhui/1-YK_RG32/2013	human
A/Pigeon/Shanghai/S1069/2013	Pigeon
A/Anhui/1-YK_RG33/2013	human
A/Anhui/1-YK_RG39/2013	human
A/Anhui/1-YK_RG40/2013	human
A/Anhui/1-YK_RG35/2013	human
A/Anhui/1-YK_RG38/2013	human
A/Anhui/1-YK_RG43/2013	human
A/Anhui/1-YK_RG44/2013	human
A/Anhui/1-YK_RG41/2013	human
A/Anhui/1-YK_RG42/2013	human
A/Anhui/1-YK_RG46/2013	human
A/chicken/Jiangxi/12201/2014	chicken
A/chicken/Jiangxi/12217/2014	chicken
A/Anhui/1-YK_RG48/2013	human
A/chicken/Jiangxi/9497/2014	chicken
A/Anhui/1-YK_RG37/2013	human
A/chicken/Jiangxi/9530/2014	chicken
A/Anhui/1-YK_RG45/2013	human
A/chicken/Jiangxi/12222/2014	chicken
A/Anhui/1-YK_RG51/2013	human
A/Hong_Kong/734/2014	human
A/Anhui/1-YK_RG52/2013	human
A/chicken/Jiangxi/12232/2014	chicken
A/chicken/Jiangxi/12208/2014	chicken
A/Anhui/1-YK_RG49/2013	human
A/chicken/Jiangxi/12219/2014	chicken
A/Anhui/1-YK_RG50/2013	human
A/chicken/Jiangxi/12247/2014	chicken
A/Anhui/1-YK_RG47/2013	human

A/chicken/Jiangxi/12248/2014	chicken
A/Anhui/1-YK_RG55/2013	human
A/chicken/Jiangxi/12239/2014	chicken
A/Anhui/1-YK_RG53/2013	human
A/Anhui/1-YK_RG54/2013	human
A/chicken/Jiangxi/12243/2014	chicken
A/Anhui/1-YK_RG59/2013	human
A/chicken/Jiangxi/12260/2014	chicken
A/Anhui/1-YK_RG60/2013	human
A/chicken/Jiangxi/12264/2014	chicken
A/chicken/Jiangxi/12251/2014	chicken
A/Anhui/1-YK_RG57/2013	human
A/chicken/Jiangxi/12256/2014	chicken
A/Anhui/1-YK_RG58/2013	human
A/chicken/Jiangxi/13210/2014	chicken
A/Anhui/1-YK_RG63/2013	human
A/chicken/Jiangxi/13252/2014	chicken
A/Anhui/1-YK_RG64/2013	human
A/chicken/Jiangxi/12265/2014	chicken
A/Anhui/1-YK_RG61/2013	human
A/Anhui/1-YK_RG62/2013	human
A/chicken/Jiangxi/12273/2014	chicken
A/Anhui/1-YK_RG67/2013	human
A/Anhui/1-YK_RG68/2013	human
A/chicken/Jiangxi/13507/2014	chicken
A/Anhui/1-YK_RG65/2013	human
A/chicken/Jiangxi/13493/2014	chicken
A/Anhui/1-YK_RG66/2013	human
A/Anhui/1-YK_RG71/2013	human
A/Anhui/1-YK_RG72/2013	human
A/Anhui/1-YK_RG69/2013	human
A/chicken/Jiangxi/13512/2014	chicken
A/Anhui/1-YK_RG70/2013	human
A/Anhui/1-YK_RG73/2013	human
A/Jiangsu/22184/2015	human
A/Guangdong/15SF082/2015	human
A/Anhui/1-YK_RG75/2013	human
A/Anhui/1-YK_RG56/2013	human
A/chicken/Jiangxi/13524/2014	chicken
A/Anhui/1-YK_RG74/2013	human
A/Anhui/1-YK_RG79/2013	human
A/Jiangsu/18828/2014	human
A/Anhui/1-YK_RG80/2013	human
A/Guangdong/15SF080/2015	human
A/Anhui/1-YK_RG77/2013	human
A/Jiangsu/19757/2015	human
A/Anhui/1-YK_RG78/2013	human

A/Anhui/1-YK_RG83/2013	human
A/Guangdong/15SF081/2015	human
A/Anhui/1-YK_RG84/2013	human
A/Zhejiang/6/2015	human
A/Anhui/1-YK_RG81/2013	human
A/Anhui/1-YK_RG82/2013	human
A/Fujian/6/2015	human
A/Anhui/1-YK_RG87/2013	human
A/Hunan/19762/2015	human
A/Anhui/1-YK_RG88/2013	human
A/Hunan/19763/2015	human
A/Anhui/1-YK_RG85/2013	human
A/Anhui/1-YK_RG86/2013	human
A/Anhui/1-YK_RG91/2013	human
A/Anhui/1-YK_RG92/2013	human
A/Anhui/1-YK_RG89/2013	human
A/Anhui/1-YK_RG90/2013	human
A/Fujian/17/2015	human
A/Zhejiang/35/2014	human
A/Anhui/1-YK_RG95/2013	human
A/Anhui/1-YK_RG96/2013	human
A/Zhejiang/34/2014	human
A/Zhejiang/36/2014	human
A/Anhui/1-YK_RG93/2013	human
A/Anhui/1-YK_RG94/2013	human
A/Anhui/1-YK_RG99/2013	human
A/Zhejiang/5/2015	human
A/Anhui/1-YK_RG100/2013	human
A/Zhejiang/3/2015	human
A/Anhui/1-YK_RG97/2013	human
A/Zhejiang/4/2015	human
A/Anhui/1-YK_RG98/2013	human
A/Anhui/1-YK_RG103/2013	human
A/Anhui/1-YK_RG104/2013	human
A/Guangdong/15SF020/2015	human
A/Anhui/1-YK_RG101/2013	human
A/Anhui/1-YK_RG102/2013	human
A/Guangdong/15SF017/2015	human
A/Anhui/1-YK_RG107/2013	human
A/Fujian/21/2015	human
A/chicken/Jiangxi/13537/2014	chicken
A/Anhui/1-YK_RG108/2013	human
A/Fujian/14/2015	human
A/Anhui/1-YK_RG76/2013	human
A/chicken/Jiangxi/13530/2014	chicken
A/Fujian/24/2015	human
A/Anhui/1-YK_RG106/2013	human

A/Fujian/23/2015	human
A/Guangdong/15SF001/2015	human
A/chicken/Jiangxi/13548/2014	chicken
A/Anhui/1-YK_RG111/2013	human
A/chicken/Jiangxi/13551/2014	chicken
A/Anhui/1-YK_RG112/2013	human
A/Fujian/7/2015	human
A/chicken/Jiangxi/13543/2014	chicken
A/Anhui/1-YK_RG109/2013	human
A/chicken/Jiangxi/13544/2014	chicken
A/Anhui/1-YK_RG110/2013	human
A/Anhui/1-YK_RG115/2013	human
A/chicken/Jiangxi/13564/2014	chicken
A/Fujian/11/2015	human
A/Fujian/8/2015	human
A/chicken/Jiangxi/14023/2014	chicken
A/Anhui/1-YK_RG116/2013	human
A/Anhui/1-YK_RG113/2013	human
A/Fujian/19/2015	human
A/chicken/Jiangxi/13556/2014	chicken
A/Anhui/1-YK_RG114/2013	human
A/Anhui/1-YK_RG119/2013	human
A/chicken/Jiangxi/14482/2014	chicken
A/Environment/Xinjiang/00344/2014	Environment
A/Anhui/1-YK_RG120/2013	human
A/Xinjiang/05914/2015	human
A/chicken/Jiangxi/14033/2014	chicken
A/Anhui/1-YK_RG117/2013	human
A/chicken/Jiangxi/14479/2014	chicken
A/Xinjiang/05915/2014	human
A/Anhui/1-YK_RG118/2013	human
A/Fujian/22/2014	human
A/Anhui/1-YK_RG123/2013	human
A/chicken/Jiangxi/14513/2014	chicken
A/Anhui/1-YK_RG124/2013	human
A/Anhui/1-YK_RG121/2013	human
A/Environment/Xinjiang/00347/2014	Environment
A/Fujian/5/2015	human
A/Anhui/1-YK_RG122/2013	human
A/Anhui/1-YK_RG127/2013	human
A/Anhui/01876/2014	human
A/Anhui/1-YK_RG128/2013	human
A/Anhui/1-YK_RG105/2013	human
A/chicken/Jiangxi/14517/2014	chicken
A/Guangdong/02497/2014	human
A/chicken/Jiangxi/14518/2014	chicken
A/Anhui/1-YK_RG125/2013	human

A/Anhui/01872/2014	human
A/Anhui/01883/2014	human
A/Anhui/1-YK_RG131/2013	human
A/Anhui/1-YK_RG132/2013	human
A/Anhui/01887/2014	human
A/Anhui/1-YK_RG129/2013	human
A/Anhui/1-YK_RG130/2013	human
A/Anhui/1-YK_RG135/2013	human
A/Fujian/2/2015	human
A/Anhui/1-YK_RG134/2013	human
A/Fujian/1/2015	human
A/Anhui/1-YK_RG133/2013	human
A/Anhui/1-YK_RG126/2013	human
A/Anhui/1-YK_RG139/2013	human
A/Fujian/20/2014	human
A/Anhui/1-YK_RG140/2013	human
A/Anhui/1-YK_RG137/2013	human
A/Anhui/1-YK_RG138/2013	human
A/Anhui/1-YK_RG136/2013	human
A/Zhejiang/24/2014	human
A/Zhejiang/23/2014	human
A/Anhui/1-YK_RG144/2013	human
A/Anhui/1-YK_RG141/2013	human
A/Anhui/1-YK_RG142/2013	human
A/fujian/16/2014	human
A/Anhui/1-YK_RG147/2013	human
A/Anhui/1-YK_RG148/2013	human
A/Anhui/1-YK_RG145/2013	human
A/Zhejiang/22/2014	human
A/Anhui/1-YK_RG146/2013	human
A/Zhejiang/21/2014	human
A/Anhui/1-YK_RG151/2013	human
A/Anhui/1-YK_RG152/2013	human
A/Zhejiang/20/2014	human
A/Anhui/1-YK_RG149/2013	human
A/Anhui/1-YK_RG150/2013	human
A/Zhejiang/15/2014	human
A/Anhui/1-YK_RG155/2013	human
A/Anhui/1-YK_RG156/2013	human
A/Anhui/1-YK_RG153/2013	human
A/Anhui/1-YK_RG154/2013	human
A/Environment/Shandong/ZZ001/2014	Environment
A/Anhui/1-YK_RG159/2013	human
A/shanghai/05/2013	human
A/Anhui/1-YK_RG160/2013	human
A/chicken/Jiangxi/14530/2014	chicken
A/Anhui/1-YK_RG157/2013	human

A/Guangdong-Guangzhou/XN09086/2014	human
A/Anhui/1-YK_RG158/2013	human
A/Jiangsu/02/2013	human
A/Anhui/1-YK_RG163/2013	human
A/jiangsu/03/2013	human
A/Anhui/1-YK_RG164/2013	human
A/Guangdong-Guangzhou/XN02778/2014	human
A/Shanghai/07/2013	human
A/Anhui/1-YK_RG161/2013	human
A/Anhui/1-YK_RG162/2013	human
A/Anhui/1-YK_RG167/2013	human
A/Shanghai/06-A/2013	human
A/Zhejiang/5/2013	human
A/Anhui/1-YK_RG168/2013	human
A/Guangdong/0024/2014	human
A/Guangdong-Guangzhou/XN08763/2014	human
A/Jiangsu/04/2013	human
A/Zhejiang/3/2013	human
A/Anhui/1-YK_RG165/2013	human
A/Beijing/01-A/2013	human
A/Zhejiang/4/2013	human
A/Anhui/1-YK_RG166/2013	human
A/Shanghai/13/2013	human
A/Anhui/1-YK_RG171/2013	human
A/Guangdong-Guangzhou/XN00457/2014	human
A/Anhui/1-YK_RG172/2013	human
A/Shanghai/14/2013	human
A/Anhui/1-YK_RG169/2013	human
A/Anhui/1-YK_RG170/2013	human
A/duck/Jiangxi/15044/2014	duck
A/Zhejiang/13/2013	human
A/Anhui/1-YK_RG176/2013	human
A/Zhejiang/14/2013	human
A/Zhejiang/02/2013	human
A/Anhui/1-YK_RG177/2013	human
A/Anhui/1-YK_RG173/2013	human
A/Zhejiang/11/2013	human
A/Zhejiang/12/2013	human
A/Anhui/1-YK_RG174/2013	human
A/chicken/Jiangxi/15524/2014	chicken
A/Guangdong-Guangzhou/XN05586/2014	human
A/Shanghai/8/2013	human
A/Henan/01/2013	human
A/Anhui/1-YK_RG180/2013	human
A/Environment/Shandong/YT003/2014	Environment
A/Zhejiang/18/2013	human
A/Anhui/1-YK_RG143/2013	human

A/Environment/Shandong/YT002/2014	Environment
A/Shandong/01/2013	human
A/Shanghai/15/2013	human
A/Anhui/1-YK_RG178/2013	human
A/Zhejiang/15/2013	human
A/Guangdong-Guangzhou/XN06839/2014	human
A/Hong_Kong/5942/2013	human
A/Anhui/1-YK_RG179/2013	human
A/Shandong/01/2014	human
A/Anhui/1-YK_RG184/2013	human
A/Anhui/1-YK_RG185/2013	human
A/Anhui/1-YK_RG181/2013	human
A/northern_shoverl/Mississippi/11OS145/2011	northern_shoverl
A/Fujian/01/2013	human
A/Jiangxi/01/2013	human
A/Anhui/1-YK_RG183/2013	human
A/Zhejiang/21/2013	human
A/Anhui/1-YK_RG188/2013	human
A/Jiangsu/07/2013	human
A/Zhejiang/1/2014	human
A/Environment/Shandong/YT007/2014	Environment
A/Zhejiang/2/2014	human
A/Anhui/1-YK_RG189/2013	human
A/Anhui/1-YK_RG186/2013	human
A/Anhui/1-YK_RG187/2013	human
A/Jilin/10117/2014	human
A/Chicken/Jilin/05/2014	Chicken
A/Environment/Xinjiang/73033/2014	Environment
A/Anhui/02/2013	human
A/Anhui/1-YK_RG192/2013	human
A/Anhui/1-YK_RG193/2013	human
A/chicken/Jiangxi/18449/2014	chicken
A/Xinjiang/73030/2014	human
A/Anhui/03/2013	human
A/chicken/Jiangxi/18008/2014	chicken
A/Zhejiang/3/2014	human
A/Anhui/1-YK_RG190/2013	human
A/Anhui/1-YK_RG191/2013	human
A/Anhui/1-YK_RG196/2013	human
A/Zhejiang/xs03/2014	human
A/Anhui/1-YK_RG197/2013	human
A/Anhui/1-YK_RG194/2013	human
A/Zhejiang/xs01/2014	human
A/Anhui/1-YK_RG195/2013	human
A/Chicken/Jilin/13202/2014	Chicken
A/Zhejiang/xs02/2014	human
A/Anhui/1-YK_RG200/2013	human

A/Guangdong/0081/2014	human
A/Environmental/Guangdong/00329/2013	Environmental
A/Anhui/1-YK_RG201/2013	human
A/Anhui/1-YK_RG198/2013	human
A/Zhejiang/10/2014	human
A/Anhui/1-YK_RG199/2013	human
A/Guangdong/0074/2014	human
A/Guangdong/0048/2014	human
A/Guangdong/0075/2014	human
A/Anhui/1-YK_RG202/2013	human
A/Guangdong/0065/2014	human
A/Anhui/1-YK_RG182/2013	human
A/Anhui/09214/2014	human
A/Anhui/09212/2014	human
A/Anhui/09186/2014	human
A/Anhui/09188/2014	human
A/Jiangsu/09387/2014	human
A/Hunan/09191/2014	human
A/Guangdong/0019/2014	human
A/Guangdong/0026/2014	human
A/Beijing/09149/2014	human
A/Hunan/12172/2014	human
A/Hunan/12173/2014	human
A/chicken/Jiangxi/9534/2014	chicken
A/Guangdong/0029/2014	human
A/silkie_chicken/Jiangxi/9472/2014	silkie_chicken
A/Guangdong/0031/2014	human
A/silkie_chicken/Jiangxi/9476/2014	silkie_chicken
A/Fujian/12/2014	human
A/chicken/Jiangxi/9558/2014	chicken
A/Fujian/14/2014	human
A/Fujian/13/2014	human
A/chicken/Jiangxi/10552/2014	chicken
A/chicken/Jiangxi/10873/2014	chicken
A/Fujian/9/2014	human
A/Fujian/8/2014	human
A/chicken/Jiangxi/10874/2014	chicken
A/Hunan/09193/2014	human
A/chicken/Jiangxi/10870/2014	chicken
A/chicken/Jiangxi/10871/2014	chicken
A/Jiangsu/09389/2014	human
A/chicken/Jiangxi/10882/2014	chicken
A/chicken/Jiangxi/10885/2014	chicken
A/Jiangsu/09043/2014	human
A/Fujian/7/2014	human
A/chicken/Jiangxi/10877/2014	chicken
A/chicken/Jiangxi/18482/2014	chicken

A/chicken/Jiangxi/10896/2014	chicken
A/environment/Bangladesh/1008/2010	environment
A/Fujian/3/2014	human
A/chicken/Jiangxi/10897/2014	chicken
A/chicken/Jiangxi/10894/2014	chicken
A/Jiangsu/09041/2014	human
A/Zhejiang/07807/2014	human
A/environment/Bangladesh/917/2012	environment
A/chicken/Jiangxi/18513/2014	chicken
A/Hunan/07833/2014	human
A/chicken/Jiangxi/18515/2014	chicken
A/Anhui/1-JCVI1_RG3/2013	human
A/Zhejiang/07800/2014	human
A/Anhui/1-BALF_RG6/2013	human
A/Anhui/1-JCVI1_RG1/2013	human
A/Anhui/1-JCVI1_RG2/2013	human
A/Anhui/1-BALF_RG9/2013	human
A/Anhui/1-BALF_RG17/2013	human
A/Zhejiang/6/2014	human
A/Anhui/1-BALF_RG7/2013	human
A/Anhui/1-BALF_RG8/2013	human
A/Fujian/2/2014	human
A/Anhui/1-BALF_RG21/2013	human
A/Anhui/1-BALF_RG20/2013	human
A/Anhui/1-BALF_RG18/2013	human
A/Anhui/1-BALF_RG19/2013	human
A/Guangdong/037/2014	human
A/Anhui/1-BALF_RG32/2013	human
A/Anhui/1-BALF_RG40/2013	human
A/Shanghai/02619/2014	human
A/environment/Jiangsu/03174/2013	environment
A/Anhui/1-BALF_RG29/2013	human
A/Anhui/1-BALF_RG31/2013	human
A/environment/Jiangsu/03137/2013	environment
A/Anhui/1-BALF_RG42/2013	human
A/Guangdong/034/2014	human
A/Anhui/1-BALF_RG44/2013	human
A/Anhui/1-BALF_RG43/2013	human
A/environment/Guangdong/02621/2013	environment
A/Anhui/1-BALF_RG45/2013	human
A/Anhui/1-BALF_RG1/2013	human
A/Anhui/1-BALF_RG41/2013	human
A/Anhui/1-BALF_RG2/2013	human
A/Guangdong/035/2014	human
A/Guangdong/033/2014	human
A/Anhui/1-BALF_RG3/2013	human
A/Guangdong/24997/2013	human

A/environment/Guangdong/25003/2013	environment
A/Anhui/1-BALF_RG4/2013	human
A/Anhui/1-BALF_RG5/2013	human
A/Guangdong/24999/2013	human
A/Environment/Hunan/39719/2015	Environment
A/Anhui/40094/2015	human
A/Environment/Anhui/33240/2015	Environment
A/Environment/Guangdong/21231/2015	Environment
A/Environment/Fujian/23611/2014	Environment
A/Environment/Anhui/39280/2015	Environment
A/Environment/Hunan/20986/2015	Environment
A/Environment/Guangdong/21275/2015	Environment
A/Environment/Guangdong/21286/2015	Environment
A/Environment/Shandong-Taian/EV02/2015	Environment
A/Environment/Shandong-Taian/EV01/2015	Environment
A/Environment/Guangdong/22351/2015	Environment
A/Environment/Guangxi/06891/2014	Environment
A/Environment/Hunan/07626/2015	Environment
A/Anhui/33228/2015	human
A/Anhui/33223/2015	human
A/Zhejiang/7/2015	human
A/chicken/Jiangxi/10939/2014	chicken
A/chicken/Jiangxi/10943/2014	chicken
A/Shandong-Taian/01/2015	human
A/Environment/Shandong-Linyi/EV01/2015	Environment
A/Zhejiang/8/2015	human
A/chicken/Jiangxi/10947/2014	chicken
A/Fujian/27/2015	human
A/chicken/Jiangxi/10948/2014	chicken
A/Zhejiang/11/2015	human
A/chicken/Jiangxi/10945/2014	chicken
A/Environment/Shandong-Taian/EV03/2015	Environment
A/chicken/Jiangxi/10946/2014	chicken
A/chicken/Jiangxi/10953/2014	chicken
A/chicken/Jiangxi/10950/2014	chicken
A/Zhejiang/9/2015	human
A/chicken/Jiangxi/10958/2014	chicken
A/chicken/Jiangxi/10959/2014	chicken
>A/ruddy_turnstone/DE/2378/1988	ruddy_turnstone
>A/red_knot/NJ/325/1989	red_knot
>A/king eider/Alaska/44060-250/2006	king_eider
>A/wild_goose/Dongting/PC0360/2012	wild_goose
A/American green winged teal/Mississippi/A00468514/2009	American_green_winged_teal
A/mallard duck/Netherlands/2/2009	mallard_duck
A/mallard duck/Netherlands/2/2015	mallard_duck
A/mallard duck/Netherlands/82/2008	mallard_duck
A/mallard duck/Netherlands/1/2011	mallard_duck

A/mallard duck/Netherlands/1/2013	mallard_duck
A/mallard duck/Netherlands/7/2009	mallard_duck
A/mallard duck/Netherlands/13/2013	mallard_duck
A/mallard duck/Netherlands/3/2015	mallard_duck
A/mallard/South Dakota/AH0038061/2016	mallard
A/mallard/South Dakota/AH0038067/2016	mallard
A/mallard/South Dakota/AH0037340/2016	mallard
A/American wigeon/Utah/AH0067123/2015	American_wigeon
A/mallard duck/Netherlands/3/2009	mallard_duck
A/mallard duck/Netherlands/4/2009	mallard_duck
A/mallard A/mallard duck/Netherlands/6/2009	mallard_A
A/mallard duck/Netherlands/11/2013	mallard_duck
A/mallard duck/Georgia/10/2016	mallard_duck
A/mallard duck/Georgia/2/2016	mallard_duck
A/mallard duck/Netherlands/18/2015	mallard_duck
>A/ostrich/South_Africa/KRB/2013	ostrich
A/cinnamon teal/Utah/AH0067154/2015	cinnamon_teal
A/northern pintail/California/AH0011948/2015	northern_pintail
>A/ruddy_turnstone/Delaware/752/2006	ruddy_turnstone
A/ruddy turnstone/Delaware/892/2006	ruddy_turnstone
>A/northern_shoveler/California/44287-164/2007	northern_shoveler
A/American green-winged teal/Mississippi/09OS046/2009	American_green-winged_teal
>A/northern_shoveler/Mississippi/09OS643/2009	northern_shoveler
>A/American_green-winged_teal/Illinois/10OS3329/2010	American_green-winged_teal
>A/American_green-winged_teal/Illinois/10OS3368/2010	American_green-winged_teal
>A/northern_shoveler/Missouri/10OS4632/2010	northern_shoveler
>A/American_green-winged_teal/Mississippi/11OS255/2011	American_green-winged_teal
>A/American_black_duck/New_Brunswick/00344/2010	American_black_duck
>A/mallard/Nova_Scotia/00372/2010	mallard
>A/green-winged_teal/New_Brunswick/00392/2010	green-winged_teal
>A/northern_shoveler/Mississippi/12OS362/2012	northern_shoveler
>A/American_green-winged_teal/Mississippi/12OS397/2012	American_green-winged_teal
>A/red_knot/Delaware_Bay/242/1994	red_knot
>A/ruddy_turnstone/New_Jersey/AI11-1678/2011	ruddy_turnstone
A/northern shoveler/Mississippi/A00682947/2008	northern_shoveler
A/blue winged teal/South Dakota/A00772794/2009	blue_winged_teal
A/blue winged teal/Louisiana/A00557206/2009	blue_winged_teal
A/mallard duck/Ohio/15OS7675/2015	mallard_duck
A/northern shoveler/California/AH0079568/2016	northern_shoveler
A/mallard/New York/AH0081570/2016	mallard
A/mallard/South Dakota/AH0038060/2016	mallard
A/American green winged teal/Utah/A00468772/2009	American_green_winged_teal
A/swan/Czech Republic/5416/2011	swan
A/wild duck/Korea/MHC40-28/2010	wild_duck
A/common teal/Korea/MHC5-8/2009	common_teal
>A/mallard/Republic_of_Georgia/2/2010	mallard
A/blue-winged teal/Texas/UGAI14-1210TX/2014	blue-winged_teal

A/blue-winged teal/Louisiana/UGAI14-209/2014	blue-winged_teal
>A/northern_shoveler/Mississippi/11OS202/2011	northern_shoveler
>A/northern_shoveler/Mississippi/12OS347/2012	northern_shoveler
A/mallard/West Virginia/AH0077733/2016	mallard
>A/equine/Sao_Paulo/4/1976	equine
A/blue-winged teal/Louisiana/UGAI14-191/2014	blue-winged_teal
A/blue-winged teal/Louisiana/UGAI14-261/2014	blue-winged_teal
A/blue-winged teal/Louisiana/UGAI14-845/2014	blue-winged_teal
A/blue-winged teal/Lousiana/UGAI14-0192/2014	blue-winged_teal
A/blue-winged teal/Lousiana/UGAI14-0203/2014	blue-winged_teal
A/blue-winged teal/Lousiana/UGAI14-0204/2014	blue-winged_teal
A/blue-winged teal/Lousiana/UGAI14-0205/2014	blue-winged_teal
A/blue-winged teal/Lousiana/UGAI14-0206/2014	blue-winged_teal
A/blue-winged teal/Lousiana/UGAI14-0207/2014	blue-winged_teal
A/blue-winged teal/Lousiana/UGAI14-0208/2014	blue-winged_teal
A/blue-winged teal/Lousiana/UGAI14-0210/2014	blue-winged_teal
A/blue-winged teal/Lousiana/UGAI14-0211/2014	blue-winged_teal
A/blue-winged teal/Lousiana/UGAI14-0217/2014	blue-winged_teal
A/blue-winged teal/Lousiana/UGAI14-0218/2014	blue-winged_teal
A/blue-winged teal/Lousiana/UGAI14-0224/2014	blue-winged_teal
A/blue-winged teal/Lousiana/UGAI14-0236/2014	blue-winged_teal
A/blue-winged teal/Lousiana/UGAI14-0322/2014	blue-winged_teal
A/blue-winged teal/Lousiana/UGAI14-0345/2014	blue-winged_teal
A/blue-winged teal/Lousiana/UGAI14-0373/2014	blue-winged_teal
A/blue-winged teal/Lousiana/UGAI14-0747/2014	blue-winged_teal
A/blue-winged teal/Lousiana/UGAI14-0751/2014	blue-winged_teal
A/blue-winged teal/Lousiana/UGAI14-0817/2014	blue-winged_teal
A/mute swan/Hungary/5973/2007	mute_swan
A/wild bird/Korea/A330/2009	wild_bird
A/wild duck/Jiangxi/19831/2009	wild_duck
>A/common_teal/Hong_Kong/MPM1670/2011	common_teal
>A/common_teal/Hong_Kong/MPL634/2011	common_teal
>A/common_teal/Hong_Kong/MPM1740/2011	common_teal
A/wild_waterfowl/Hong_Kong/MPL705/2011	wild_waterfowl
>A/wild_waterfowl/Hong_Kong/MPM2121/2011	wild_waterfowl
>A/wild_waterfowl/Hong_Kong/MPL1006/2011	wild_waterfowl
>A/silkie_chicken/Wenzhou/792/2013	silkie_chicken
>A/silkie_chicken/Wenzhou/816/2013	silkie_chicken
A/lesser white-fronted goose/HuNan/412-3Y/2010	lesser_white-fronted_goose
A/wild bird/Jiangxi/34458/2013	wild_bird
A/wild bird/Jiangxi/34503/2013	wild_bird
A/wild bird/Jiangxi/35982/2013	wild_bird
A/wild bird/Korea/A331/2009	wild_bird
A/silkie chicken/Wenzhou/793/2013	silkie_chicken
A/Anas platyrhynchos/Belgium/25576cls41/2012	Anas_platyrhynchos
